# *In silico* Identification and Mechanism Exploration of Hepatotoxic Ingredients in Traditional Chinese Medicine

**DOI:** 10.3389/fphar.2019.00458

**Published:** 2019-05-03

**Authors:** Qihui Wu, Chuipu Cai, Pengfei Guo, Meiling Chen, Xiaoqin Wu, Jingwei Zhou, Yunxia Luo, Yidan Zou, Ai-lin Liu, Qi Wang, Zaoyuan Kuang, Jiansong Fang

**Affiliations:** ^1^Institute of Clinical Pharmacology, Guangzhou University of Chinese Medicine, Guangzhou, China; ^2^School of Basic Medical Sciences, Guangzhou University of Chinese Medicine, Guangzhou, China; ^3^Clinical Research Laboratory, Hainan Province Hospital of Traditional Chinese Medicine, Haikou, China; ^4^Lerner Research Institute, Cleveland Clinic, Cleveland, OH, United States; ^5^Institute of Materia Medica, Chinese Academy of Medical Sciences and Peking Union Medical College, Beijing, China

**Keywords:** herb induced liver injury, consensus model, traditional chinese medicine, hepatotoxicity mechanism, *in silico*

## Abstract

**Backgrounds and Aims:**

Recently, a growing number of hepatotoxicity cases aroused by Traditional Chinese Medicine (TCM) have been reported, causing increasing concern. To date, the reported predictive models for drug induced liver injury show low prediction accuracy and there are still no related reports for hepatotoxicity evaluation of TCM systematically. Additionally, the mechanism of herb induced liver injury (HILI) still remains unknown. The aim of the study was to identify potential hepatotoxic ingredients in TCM and explore the molecular mechanism of TCM against HILI.

**Materials and Methods:**

In this study, we developed consensus models for HILI prediction by integrating the best single classifiers. The consensus model with best performance was applied to identify the potential hepatotoxic ingredients from the Traditional Chinese Medicine Systems Pharmacology database (TCMSP). Systems pharmacology analyses, including multiple network construction and KEGG pathway enrichment, were performed to further explore the hepatotoxicity mechanism of TCM.

**Results:**

16 single classifiers were built by combining four machine learning methods with four different sets of fingerprints. After systematic evaluation, the best four single classifiers were selected, which achieved a Matthews correlation coefficient (MCC) value of 0.702, 0.691, 0.659, and 0.717, respectively. To improve the predictive capacity of single models, consensus prediction method was used to integrate the best four single classifiers. Results showed that the consensus model C-3 (MCC = 0.78) outperformed the four single classifiers and other consensus models. Subsequently, 5,666 potential hepatotoxic compounds were identified by C-3 model. We integrated the top 10 hepatotoxic herbs and discussed the hepatotoxicity mechanism of TCM via systems pharmacology approach. Finally, *Chaihu* was selected as the case study for exploring the molecular mechanism of hepatotoxicity.

**Conclusion:**

Overall, this study provides a high accurate approach to predict HILI and an *in silico* perspective into understanding the hepatotoxicity mechanism of TCM, which might facilitate the discovery and development of new drugs.

## Introduction

Liver injury induced by drug, novel foods or phytotherapy, also known as hepatotoxicity, is still a major clinical and pharmaceutical concern (Amadi and Orisakwe, 2018; Hammann et al., 2018; Kyawzaw et al., 2018; Real et al., 2018). According to the data from United States National Institute of Diabetes and Digestive and Kidney Diseases (NIDDK), hepatotoxicity accounts for 50% of all liver failure cases in the United States (Tujios and Fontana, 2011). Additionally, it is one of the leading causes of drug failure in trials and withdrawal from the market (Segall and Barber, 2014).

Over the last decades, Traditional Chinese Medicine (TCM), regarded as safe and natural, has received growing attention (Vikas and Singh, 2012). Dating back to 2,500 years ago, TCM has played an irreplaceable role in Chinese health care system to fight against various diseases and keep health for Chinese people (Cheung, 2011). Moreover, TCM is a gorgeous cradle of new active compounds in the course of drug discovery. For example, artemisinin (*Qinghaosu*) (Tu, 2011) is an effective anti-malaria drug, which is extracted from Chinese herb *Artemisia annua* L. (*Qinghao*). Despite its long clinical success, the most annoying problem in the herbal TCM area is the lack of proven efficacy in large-scale clinical studies and the unknown adverse reactions (ADRs). Side effects (SE) aroused by TCM, especially herb induced liver injury (HILI), have been reported widely (Teschke et al., 2015; Amadi and Orisakwe, 2018; Jing and Teschke, 2018), which also severely restricts the application of TCM (Lee et al., 2015; Kaplowitz, 2018). Thus, there is an urgent need to identify the potential hepatotoxic ingredients in TCM and explore the molecular mechanism of these compounds against HILI.

Generally, it is difficult to detect hepatotoxicity in the early phase of drug development due to its complex mechanism and lacking of scientific basis (Chatila, 1999; Williams, 2006). The traditional approach with experimental validation of drug induced hepatotoxicity on animals is costly, time-consuming and labor-intensive (Merz et al., 2014). Nowadays, due to the increasing number of hepatotoxic drugs identified in clinical or experimental studies, *in silico* prediction, such as machine learning (ML) approach based on ligand characteristic, provides the possibility of making predictions for HILI without knowing their underlying mechanisms. In this study, we try to identify hepatotoxic ingredients of TCM from a ligand-based ML perspective, and explore the hepatotoxicity mechanism via system pharmacology approach.

Quantitative structure-activity relationship (QSAR) are the most widely used *in silico* approach in absorption, distribution, metabolism, excretion and toxicity (ADMET) prediction (Cheng et al., 2013). Thus far, multiples of QSAR models have been generated for hepatotoxicity study of chemicals (Rodgers et al., 2010; Xu et al., 2015; Zhang et al., 2016; Cronin et al., 2017). For instance, Rodgers et al. (2010) reported a QSAR model with approximately 200 compounds by using the *k*-nearest neighbor (*k*NN) method, which achieved the sensitivity and specificity values of more than 73.7% and 94.4% for the prediction of liver adverse effects of drugs. Xu et al. (2015) developed hepatotoxicity prediction models by utilizing deep learning architectures, and the best model trained on 475 drugs predicted an external validation set of 198 drugs with an accuracy of 86.9%, sensitivity of 82.5%, specificity of 92.9%, and the area under the receiver operating characteristic (ROC) curve (AUC) of 0.955. Besides, Zhang et al. (2016) developed classification models using five machine learning methods based on MACCS Keys fingerprint and FingerPrint4 (FP4). However, the AUC values for the best model are only 0.656, 0.552, and 0.607 for training set, test set and external validation set, respectively. Notably, Cronin et al. (2017) proposed a new paradigm for *in silico* modeling by incorporating adverse outcome pathways, providing new insights into the QSAR models. Taken together, the predictive accuracies of current published QSAR models for hepatotoxicity remains to be improved due to incomplete data source. In addition, there are few consensus models reported to integrate single classifier for hepatotoxicity prediction. Furthermore, the hepatotoxicity models generated have not been applied to predict herb ingredients from TCM database yet.

**FIGURE 1:**
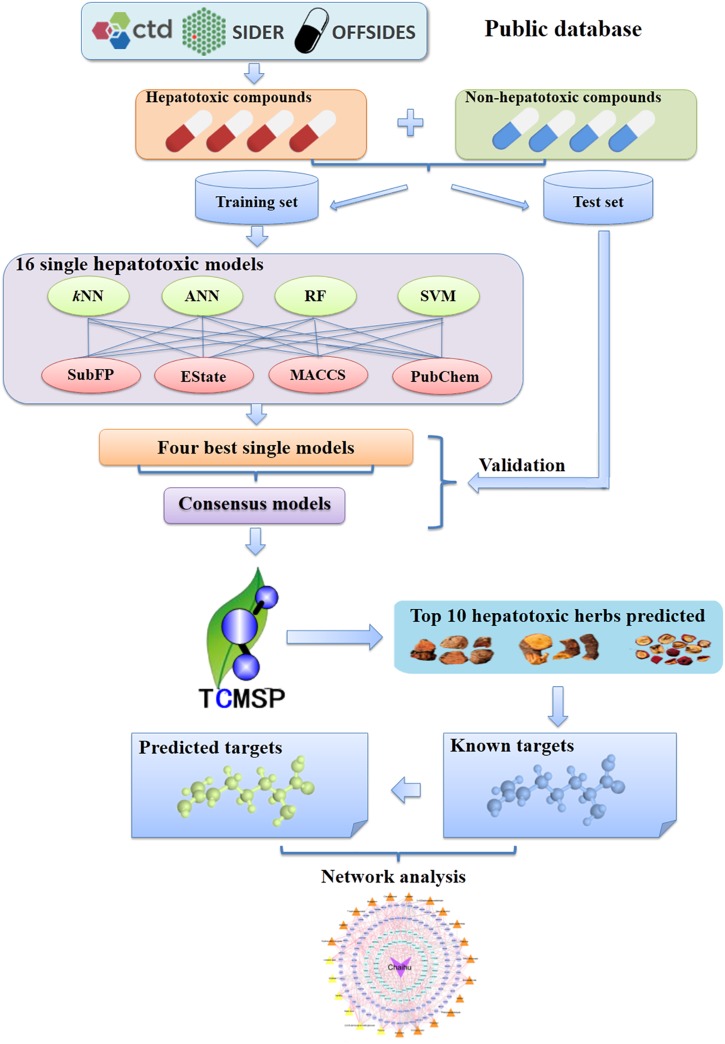
Schematic diagram of models building, identification of potential hepatotoxic ingredients and unraveling the hepatotoxicity mechanisms based on systems pharmacology approaches. CTD, Comparative Toxicogenomics Database; *k*NN, *k*-nearest neighbor; ANN, artificial neural network; RF, random forest; SVM, support vector machine; SubFP, Substructure fingerprint; EState, EState fingerprint; MACCS, MACCS Keys fingerprint; Pubchem, PubChem fingerprint; TCMSP, the Traditional Chinese Medicine systems pharmacology database and analysis platform.

In this work, we constructed a high-quality data set involving 619 hepatotoxic and 1,857 non-hepatotoxic compounds. All the hepatotoxic compounds were collected by integrating available adverse reactions databases (e.g., SIDER). Consensus models were generated to screen the Traditional Chinese Medicine systems pharmacology database and analysis platform (TCMSP) database. After identifying hepatotoxic ingredients in TCM, the molecular mechanisms of hepatotoxicity were explored. The detailed workflow could be seen in Figure 1. Firstly, data set containing hepatotoxic and non-hepatotoxic compounds were randomly assigned into training set and test set. Subsequently, four machine learning methods including artificial neural network (ANN), support vector machine (SVM), random forest (RF) and *k*-nearest neighbors (*k*NN) together with four different sets of fingerprints (EState, MACCS, PubChem, and SubFP) were utilized to develop 16 classifiers. Moreover, the consensus models by integrating the best four single classifiers were applied to screen the TCMSP database after systematic evaluation. Finally, to decipher the hepatotoxicity mechanism of TCM, systems pharmacology analyses of top 10 herbs with the largest number of potential hepatotoxic ingredients were carried out via integrating known and predicted targets.

## Materials and Methods

### Data Preparation

In this study, all the liver toxic compounds were collected from three public databases, including side effect resource (SIDER) (Kuhn et al., 2010), OFFSIDES (Tatonetti et al., 2012) and Comparative Toxicogenomics Database (CTD) (Davis et al., 2019). Specifically, we firstly collected all the adverse SE of liver according to the criteria on “Common Terminology Criteria for Adverse Events” (CTCAE, version 4.03, 2010) released by the United States Department of Health and Human Services. Then all the liver related SE terms were annotated using Medical Subject Headings (MeSH) or Unified Medical Language System (UMLS) vocabularies (Bodenreider, 2004). Compounds that corresponded with these MeSH or UMLS IDs were extracted from the three databases. Only side effect information labeled with “frequent” or “direct evidence” were preserved. Additionally, the proteins, organic metals, compounds with molecular weight larger than 800 or smaller than 100, and duplicate structures were removed for avoiding potential noise (Wang et al., 1995). Finally, 619 liver toxic compounds were obtained for model construction.

After that, triple corresponding decoys were generated in RApid DEcoy Retriever (RADER) online database with a similarity threshold value of 0.75 between liver toxic compounds and decoys (Ling et al., 2016). Only molecules with molecular weight between 100 and 800 were preserved. All the compounds were randomly separated into training set and test set with ratio of 3:1. We considered the liver toxic compounds as hepatotoxic (labeled as “1”) during the calculation. Since decoys were randomly selected from a massive “decoy pool” containing 70,030,298 compounds (Ling et al., 2016) after eliminating hepatotoxic compounds, decoys were supposed as non-hepatotoxic (labeled as “0”) in this study. Eventually, the training set contained 453 hepatotoxic compounds and 1,359 non-hepatotoxic compounds, while the test set was consisted of 166 hepatotoxic compounds and 498 non-hepatotoxic compounds. Detailed information about the two data sets can be found in Supplementary Tables S1, S2. Meanwhile, herb ingredients covering 499 traditional Chinese herbs registered in the Chinese pharmacopeia were downloaded from TCMSP (Ru et al., 2014). Then 13,139 unique compounds were finally collected after removing duplicates.

All the data sets above were processed as the following three steps: Firstly, inorganic compounds were removed and hydrogen atoms were added. Secondly, strong acids were deprotonated and strong bases were protonated. Thirdly, three-dimensional (3D) conformers were generated by using washing and energy minimizing in Molecular Operating Environment (MOE) software (MOE, version 2010.10, Chemical Computing Group Inc., Montreal, QC, Canada, 2010).

As the chemical diversity of the datasets plays a vital role in the application domain of predictive model, we further employed the chemical space analysis to evaluate the chemical diversity within the data sets. As shown in Supplementary Figure S1, diverse chemical space distributions for all compounds as well as overlaps among the compounds within these data sets can be observed. In addition, we also proposed the chemical space analysis for TCMSP since the predictive models were applied to identify the potential hepatotoxic compounds in TCMSP. As presented in Supplementary Figure S2, the applicability domain of the predictive models covers 90.6% (11,902/13,139) chemicals of TCMSP.

### Molecular Representation

Molecular fingerprints are often utilized for describing chemical structures. The main idea of fingerprints is to describe molecules based on molecular fragment. Through the segmentation of the molecular structure, a series of fragments as the characterization of the molecular structure can be obtained, and suitable fingerprints can enhance the performance of models (Fang et al., 2015a). Here, four common types of molecular fingerprints were calculated with PaDEL-Descriptor software (Yap, 2011) to represent molecular substructures and fragments information for each molecule. The four types of fingerprints are EState fingerprint (EState), MACCS Keys fingerprint (MACCS), PubChem fingerprint (PubChem) and Substructure fingerprint (SubFP).

### Machine Learning Methods and Consensus Prediction for Model Building

In this part, four different machine learning methods including ANN, SVM, RF and *k*NN, were applied to develop predictive models and then consensus prediction was adopted to generate combined models. All the algorithms except SVM were performed using Orange Canvas (version, 2.7).

#### Artificial Neural Network (ANN)

Artificial Neural Network is a powerful computational algorithm with sufficient accuracy which mimics the complex networks of neural connections in the neural brain (Dan, 1996). The network was made up of three layers: one input layer, one hidden layer and one output layer. ANN could identify complex non-linear relationship between input and output sets (Basheer and Hajmeer, 2000).

#### Support Vector Machine (SVM)

The purpose of SVM is to find a hyperplane which could discriminate molecules from different categories. Kernel function makes SVM deal with high-dimensional data effectively. The SVM algorithm was provided by the LibSVM package (Chang and Lin, 2011). And the commonly used kernel function radial bias function (RBF) was utilized to develop models after seeking the penalty parameter “C” and different kernel parameter “γ” with grid search strategy based on a 5-fold cross-validation in LibSVM.

#### Random Forest (RF)

Random Forest is also a widely used machine learning method (Biau et al., 2008). The core idea of this classification algorithm is to train the input sample vectors through constructing multitudes of decision trees in the forest. Each tree gives a classification, which means the tree “vector” for the class. Finally, the forest can select the classification which has the most vectors in all the trees of the forest.

#### *k*-Nearest Neighbors (*k*NN)

*k*-Nearest Neighbors is a non-parametric method to categorize objects based on closest training examples in the feature space (Larose, 2004). The distance or similarity between each training sample was calculated by the algorithm to choose the list of its nearest neighbor, which can be classified according to the majority of the nearest neighbors (Cover and Hart, 1967). In this study, *k* (the number of nearest neighbors value) was set to the default (*k* = 5) and Hamming distance was selected for distance metric.

#### Consensus Models and Prediction

The main purpose of the consensus model is to combine the predicted results from various single classifiers for improving the predictive accuracy. It is generally considered that the consensus model is in a position to optimize the performance of the single classifier by improving predictive reliability (Cheng et al., 2011; Mansouri et al., 2013). Various kinds of noise from a single model can be reduced by consensus modeling (Fang et al., 2016b). In this study, four consensus models based on the best four single classifiers were generated through a “consensus prediction” procedure (Fang et al., 2015b). First, the training set and test set were screened with four single classifiers, and the compounds were considered as “hepatotoxicity” if predicted as “+1” by one of the four single classifiers. The procedure is defined as consensus prediction C1. Similarly, we obtained consensus prediction C2 (predicted as “+1” by two of the four single classifiers), C3 (predicted as “+1” by three of the four single classifiers), and C4 (predicted as “+1” by all the four single classifiers).

### Performance Evaluation of Models

All classification models were evaluated by counting the numbers of true positives (TP), true negatives (TN), false positives (FP), and false negatives (FN) compounds. Additionally, sensitivity (SE), specificity (SP), overall predictive accuracy (Q) and Matthews correlation coefficient (MCC) were calculated with equations (1)–(4). Among them, SE and SP represent predictive accuracy of hepatotoxic and non-hepatotoxic compounds, respectively. Q represents predictive accuracy of total compounds, while MCC is the most significant indicator to measure the prediction performance of all the models. Usually, the higher the MCC value is, the better the model is.

(1) $$\text{SE}=\frac{\text{TP}}{\text{TP}+\text{FN}}$$

(2) $$\text{SP}=\frac{\text{TN}}{\text{TN}+\text{FP}}$$

(3) $$\text{Q}=\frac{\text{TP}+\text{TN}}{\text{TP}+\text{FN}+\text{FP}+\text{TN}}$$

(4) $$\text{MCC}=\frac{\text{TP}\times \text{TN}-\text{FN}\times \text{FP}}{\sqrt{\left(\text{TP}+\text{FN})(TP+\text{FP})(\text{TN}+\text{FN})(\text{TN}+\text{FP}\right)}}$$

Furthermore, the area under the receiver operating characteristic curve (AUC) value was also calculated. A perfect classifier can be found as AUC = 1.0 while the classifier has no discriminative power as AUC = 0.5.

### Collection of Hepatotoxicity-Related Genes

The genes associated with liver diseases were collected from the previous public reference (Lu et al., 2015), which integrated the data from four public databases, including the Online Mendelian Inheritance in Man (OMIM) database (Hamosh et al., 2005), HuGE Navigator (Yu et al., 2008), PharmGKB (Hernandezboussard et al., 2008) and CTD (Davis et al., 2019). In this study, all collected genes were annotated using gene Entrez ID and official gene symbols based on the NCBI database^1^ and then mapped to corresponding Uniprot ID^2^. After removing duplicates, 627 unique genes related with liver disease were finally obtained.

### Integration of Known and Predicted Hepatotoxic Target Proteins

The known hepatotoxic targets for compounds were acquired by two steps: (1) collecting known targets of compounds from our previous integrated natural products database (Fang et al., 2017a); (2) overlapping known targets and hepatotoxicity- related genes. The putative targets of compounds were predicted via a balanced substructure-drug-target network-based inference (bSDTNBI) (Wu et al., 2016; Fang et al., 2017c) approach, which prioritizes potential targets utilizing resource-diffusion processes for both known drugs and new chemical entities (NCEs) via substructure-drug-target network (Wu et al., 2016). In this study, the top 20 putative targets for each compound with known targets were selected. Similarly, the predicted hepatotoxic targets were acquired through overlapping predicted targets and hepatotoxicity-related genes. All the target names were subsequently normalized to the official gene name using the UniProt database (see text footnote 2).

### Network Construction and Analysis

To decipher the complex hepatotoxicity mechanisms of TCM, two types of network including herb-herb network (H-H network) and compound-target network (C-T network) were generated by Cytoscape (version 3.2.1). In the graphical network, the compounds or targets were presented by nodes, and edges encoded the interactions. The degree of nodes was calculated to measure its topological property as it characterizes the most important nodes in a network.

## Results and Discussion

### Model Building and Evaluation

#### Single Model Building and Evaluation

In this study, 16 single models were developed by four different algorithms (ANN, SVM, RF, and *k*NN) using four common types of fingerprints (EState, MACCS, PubChem and SubFP). The performance of each model was measured with the internal 5-fold cross validation in training set. Then, in order to further validate the predictive capability of our models, they were applied to predict the test set consisting of 664 compounds. The comprehensive performances of all the 16 classifiers are provided in Table 1.

**Table 1:** Performance of 16 single classifiers using four sets of fingerprints and four modeling methods on 5-fold cross validation and test set.

Model name	Training set (5-fold cross validation)					Test set				
	Q	SE	SP	MCC	AUC	Q	SE	SP	MCC	AUC
EState-ANN	0.848	0.755	0.888	0.609	0.902	0.859	0.680	0.911	0.607	0.899
EState-RF	0.820	0.789	0.824	0.460	0.882	0.821	0.737	0.835	0.471	0.861
EState-SVM	0.837	0.751	0.855	0.528	0.878	0.839	0.627	0.910	0.556	0.867
EState-*k*NN	0.834	0.713	0.864	0.532	0.855	0.839	0.602	0.918	0.551	0.854
MACCS-ANN	0.898	0.829	0.919	0.722	0.943	0.887	0.789	0.920	0.702	0.925
MACCS-RF	0.866	0.769	0.892	0.628	0.926	0.851	0.729	0.892	0.610	0.900
MACCS-SVM	0.902	0.861	0.913	0.730	0.944	0.886	0.753	0.930	0.691	0.920
MACCS-*k*NN	0.884	0.801	0.908	0.681	0.915	0.875	0.711	0.930	0.659	0.886
PubChem-ANN	0.876	0.779	0.904	0.659	0.915	0.852	0.681	0.910	0.600	0.877
PubChem-RF	0.848	0.714	0.888	0.585	0.915	0.816	0.639	0.876	0.512	0.843
PubChem-SVM	0.876	0.799	0.897	0.656	0.932	0.852	0.669	0.914	0.597	0.881
PubChem-*k*NN	0.870	0.772	0.897	0.641	0.888	0.827	0.657	0.884	0.539	0.830
SubFP-ANN	0.896	0.822	0.917	0.714	0.935	0.893	0.795	0.926	0.717	0.911
SubFP-RF	0.847	0.747	0.872	0.568	0.904	0.813	0.663	0.863	0.514	0.873
SubFP-SVM	0.869	0.802	0.886	0.632	0.929	0.857	0.753	0.892	0.629	0.904
SubFP-*k*NN	0.877	0.795	0.900	0.661	0.898	0.852	0.705	0.902	0.606	0.885

*Q: overall predictive accuracy; SE: sensitivity; SP: specificity; MCC: Matthews correlation coefficient; AUC: the area under the receiver operating characteristic curve; ANN: artificial neural network; SVM: support vector machine; RF: random forest; kNN: k-nearest neighbors; EState: EState fingerprint; MACCS: MACCS keys fingerprint; PubChem: PubChem fingerprint; SubFP: Substructure fingerprint.*

As illustrated in Table 1, the overall predictive accuracies for 16 classifiers are acceptable. The *Q*-values of all single models are higher than 0.8 and the highest one is MACCS-SVM model reaching up to 0.902. The AUC value for each model is greater than 0.8, ranging from 0.855 to 0.944 on 5-fold cross validation as well as 0.830 to 0.920 on test set validation. MCC values, the most significant index to evaluate models, also have acceptable performances. The highest one (MACCS-ANN) reaches up to 0.722 on 5-fold cross validation and 0.702 on test set validation. Given the balance between SE and SP, we consider MACCS-ANN, MACCS-SVM, MACCS-*k*NN, and SubFP-ANN as the best four single models, which satisfy the condition that all of the MCC, SE as well as SP values are greater than 0.65 on the test set.

#### Performance of the Consensus Models

**FIGURE 2:**
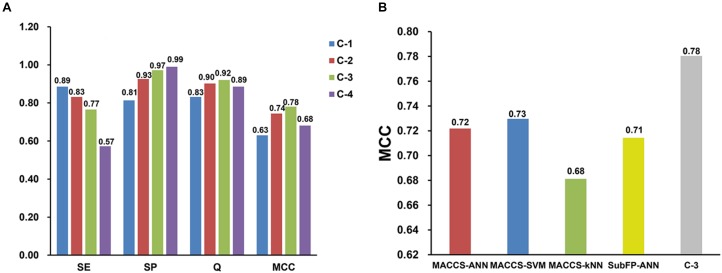
Performance comparisons of four consensus models **(A)** and four best single models with consensus model 3 (C-3) **(B)** on test set validation. SE, sensitivity; SP, specificity; Q, accuracy; MCC, Matthews correlation coefficient.

Consensus predictions were adopted to integrate the best four single classifiers discussed above, then four consensus models named C-1, C-2, C-3, C-4 were generated. The SE, SP, Q, and MCC values for consensus models on test set are presented in Figure 2A. The SE values of C-1 and C-2 are 0.89 and 0.83, which are higher than that of C-3 (SE = 0.77) and C-4 (SE = 0.57). Nevertheless, the SP values were opposite. The SP values for C-3 and C-4 are 0.97 and 0.99, greater than C-1 (SP = 0.81) and C-2 (SP = 0.93). The possible reason is that strict criterion for hepatotoxic prediction leads to higher SP and lower SE, and vice versa. Overall, C-3 model performs best among the four models, which keeps an equilibrium between specificity (SP = 0.97) and sensitivity (SE = 0.77), and has the highest MCC (MCC = 0.78) and Q (Q = 0.92) values.

Moreover, the performance between the best four single models and C-3 model was compared on test set. As shown in Figure 2B, the C-3 model has a MCC value of 0.78, which is much greater than that of any other single models. This suggests that the C-3 model does enhance the predictive capabilities.

#### The Comparison of Performance Between C-3 Model and the Hepatotoxic Prediction by Discovery Studio (DS)

The ADME/T Descriptors is a mature protocol of commercial software Discovery Studio (version 4.0, Accelrys Inc., San Diego, CA, 2013) to estimate various pharmacokinetics features of ligands, including aqueous solubility, blood brain barrier penetration, CYP2D6 binding, intestinal absorption and hepatotoxicity. In order to further validate the predictive capability of C-3 model in practice, we compared the accuracy between C-3 model and the hepatotoxic prediction in DS software. First, 184 withdrawn drugs were downloaded from DrugBank database (Wishart et al., 2006). After browsing the detailed information about the withdrawal causes, we found that 11 of these drugs were withdrawn from the market due to clearly labeled hepatotoxicity. To evaluate if both of C-3 and DS can recognize these hepatotoxic drugs, C-3 model and DS were applied to predict these 11 drugs.

Table 2 shows that 9 out of 11 hepatotoxic compounds are predicted correctly by C-3 model, while only 7 compounds are considered hepatotoxic by DS software. However, once the positive threshold value is improved by means of setting the probability to 0.8, the correct number of DS descends to only 5, while the number of consensus model is 8. This also demonstrates that our model has higher accuracy to recognize hepatotoxic compounds.

**Table 2:** Comparison of the hepatotoxic prediction results between consensus model 3 (C-3) and Discovery Studio (DS) for 11 withdrawn drugs due to hepatotoxicity.

Drugbank ID	Name	Probability (*p*)					Prediction		*p* = 0.8	
		M1	M2	M3	M4	DS	C-3	DS	C-3	DS
DB00197	Troglitazone	0.999	0.997	0.792	0.999	0.768	1	1	1	0
DB00323	Tolcapone	1	0.986	1	1	0.96	1	1	1	1
DB01149	Nefazodone	0.992	0.947	0.866	0.795	0.198	1	0	1	0
DB04743	Nimesulide	1	0.943	0.756	0.951	0.827	1	1	1	1
DB04831	Ticrynafen	1	0.978	0	0.822	0.973	1	1	1	1
DB04898	Ximelagatran	1	1	0.848	0.297	0.172	1	0	1	0
DB06268	Sitaxentan	0.986	0.945	0.997	0.986	0.9	1	1	1	1
DB00685	Trovafloxacin	0.98	0.945	1	0.981	0.536	1	1	1	0
DB08986	Etifoxine	0.001	0.005	0	0.024	0.92	0	1	0	1
DB09247	Iproclozide	0.313	0.3	0.281	0.019	0.205	0	0	0	0
DB09251	Phenoxypropazine	0.974	0.362	0.528	0.583	0.072	1	0	0	0

*M1: SUB-ANN model; M2: MACCS-kNN model; M3: MACCS-ANN model; M4: MACCS-SVM model; C-3: consensus model 3.*

### Identification of Potential Hepatotoxic Ingredients Based on TCMSP

The TCMSP database was built based on the framework of systems pharmacology which is aimed at accelerating the development of herbal medicines and drug discovery. It is a widely recognized database with comprehensive and authoritative data sources about the TCM (Ru et al., 2014). Therefore, the TCMSP database was selected to screen the potential hepatotoxic ingredients of TCM.

**FIGURE 3:**
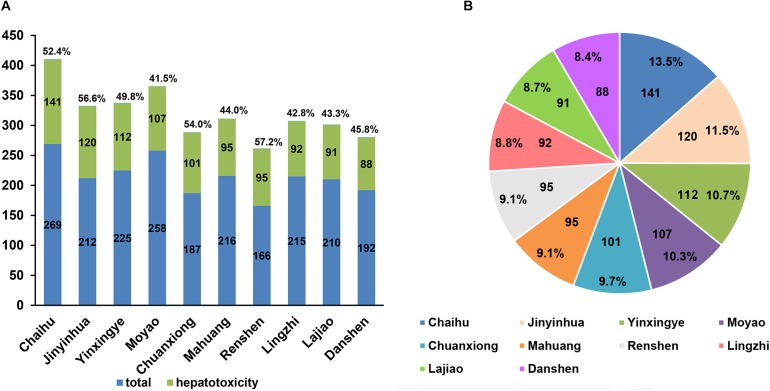
The top 10 herbs with the highest number of hepatotoxic compounds as well as the corresponding proportions of hepatotoxic ingredient numbers to the number of compounds in each herb **(A)** and the corresponding proportions of the predicted hepatotoxic compounds for each herb to the total 1,042 hepatotoxic compounds of top 10 herbs **(B)**.

As discussed above, the C-3 model, which performed best, was finally chosen to identify the hepatotoxic ingredients among 13,139 unique compounds from TCMSP. Eventually, 5,666 out of them were predicted as hepatotoxic (Supplementary Table S3). To further explore the distribution of hepatotoxic compounds in herbs, the total number of compounds in each herb were obtained from TCMSP, while the number of predicted hepatotoxic compounds in each herb were also calculated. After that, each herb with specific number of hepatotoxic compounds was sorted in a descending order. Figure 3A shows the top 10 herbs possessing the highest number of hepatotoxic compounds, as well as the corresponding proportions of hepatotoxic ingredients numbers to the number of compounds in each herb. They are *Bupleurum L.* (*Chaihu*), *Lonicera japonica* (*Jinyinhua*), *G. folium* (*the leaves of Ginkgo, Yinxingye*)*, Commiphora myrrha* (*Moyao*)*, Ligusticum chuanxiong (Chuanxiong)*, *Ephedra sinica* (*Mahuang*)*, Panax ginseng (Renshen)*, *Micromeria biflora* (*Lingzhi*)*, Capsicum annuum* (*Lajiao*)*, Salvia miltiorrhiza* (*Danshen*), respectively. The top 10 herbs contain 1,042 unique hepatotoxic compounds predicted (Supplementary Table S4). Figure 3B gives the corresponding proportions of the predicted hepatotoxic compounds for each herb to the total 1,042 hepatotoxic compounds of top 10 herbs.

Among the top 10 herbs, *Bupleurum L.* (*Chaihu*) has the largest number (141) of potential hepatotoxic compounds, and 52% (141/269) of compounds in *Chaihu* were predicted to have hepatotoxicity. Interestingly, Lee et al. (2011) reported the risk of hospitalization for liver injury by using *radix bupleuri* (*Chaihu*), referring to 61 hepatotoxicity cases in 639,779 patients with chronic hepatitis B virus infection. Besides, both of *Ephedra sinica* (*Mahuang*) and *Micromeria biflora* (*Lingzhi*) also have been emphasized to be alerted for their hepatotoxicity. Estes et al. (2003) reported two cases of *Ephedra sinica* (*Mahuang*) linked acute liver failure resulting in orthotopic liver transplantation. And Yuen et al. (2004) referred to a case that in taking a formulation of *Ganoderma lucidum* (*lingzhi*) caused a significant hepatotoxicity in 2004. Collectively, these clinical reports are consistent with our predictions. It is worth noting that the hepatotoxicity level of herb are not only related to the number of potential hepatotoxic compounds in each herb, but also depended on the doses of the hepatotoxic compounds contained.

### Systems Pharmacology Analysis of Hepatotoxic Compounds in Top 10 Herbs

In recent years, systems pharmacology as an emerging new field, has made great contribution to unravel the nature of TCM and the actions of prescriptions (Fang et al., 2016a, 2017b; Cai et al., 2018). In our study, the systems pharmacology approach was applied to explore the hepatotoxicity mechanism of TCM.

#### Herb-Herb Network

**FIGURE 4:**
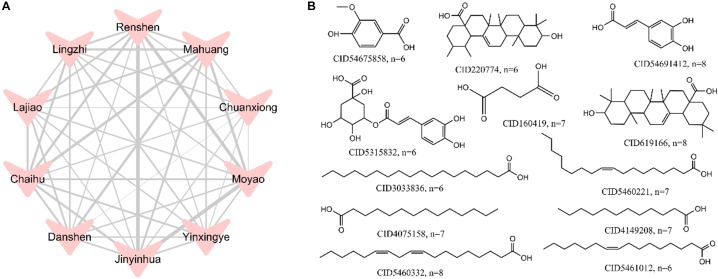
A herb-herb (H-H) network of the top 10 herbs **(A)** and the structures of 1,042 hepatotoxic compounds with frequency greater than 6 **(B)**. The width of lines was based on the number of duplicate compounds between two herbs, which was proportional to degree.

To examine whether the top 10 herbs discussed above have similar compounds in terms of chemical structures, we explored the relationships among the top 10 herbs and constructed a herb-herb (H-H) network (Figure 4A). This network represents the relationship of different herbs based on the number of duplicate compounds between two herbs. The width of connected lines between two herbs is proportional to degree. As seen in Figure 4A, the top 10 herbs share lots of duplicate molecules in terms of structures. For example, network analysis indicates that *Lonicera japonica* (*Jinyinhua*) and *Commiphora myrrha* (*Moyao*) have the largest number of duplicate compounds (*n* = 29), followed by *Lonicera japonica (Jinyinhua)* and *Panax ginseng* (*Renshen*) (*n* = 27), *Commiphora myrrha* (*Moyao*) and *Panax ginseng* (*Renshen*) (*n* = 25). *Ligusticum chuanxiong* (*Chuanxiong*) and *Salvia miltiorrhiza* (*Danshen*), with the least number of common structures, also have 8 duplicate compounds.

Meanwhile, to reveal the most common hepatotoxic ingredients in top 10 herbs, frequency analysis of 1,042 potential hepatotoxic compounds was performed. Figure 4B displays the structures of compounds which have frequency greater than 6. Among them, CID5460332 (Linoleic acid), CID54691412 (*trans-*caffeic acid) and CID619166 (Oleanoic acid) show the highest frequency (*n* = 8), which indicates these compounds should be highly alerted for hepatotoxicity.

#### Compound-Target (C-T) Network and KEGG Analysis

**FIGURE 5:**
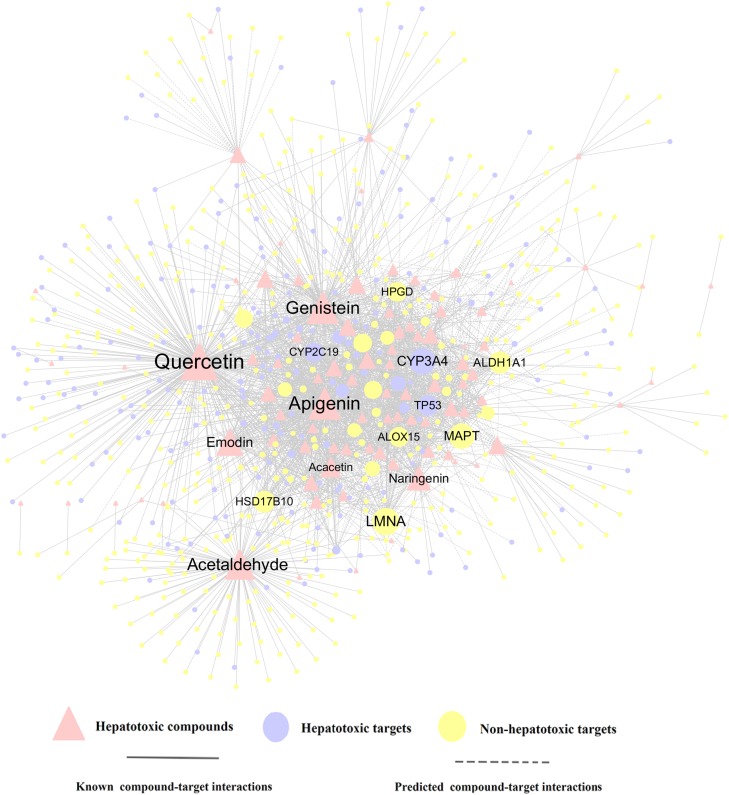
A compound-target (C-T) network consists of 1,056 known interactions as well as 1,245 predicted interactions, including 91 compounds, 211 hepatotoxic targets and 464 non-hepatotoxic targets. The label font size and node size are proportional to degree.

To further decipher the hepatotoxicity mechanism of action between herb ingredients and targets, a compound-target (C-T) network of the top 10 herbs was constructed, which contains 1,056 known compound-target interactions (CTIs) and 1,245 predicted CTIs. This network connects 91 hepatotoxic compounds to 675 target nodes (211 hepatotoxic targets and 464 non-hepatotoxic proteins). Figure 5 suggests that most compounds are connected to multiple targets with the average degree of 7.4 for each compound. Among the 91 compounds, quercetin (CID5280343, *D* = 240) has the highest number of target connections (D), followed by apigenin (CID5280443, *D* = 134) and genistein (CID5280961, *D* = 131). For the 675 targets, 211 out of them are associated with liver toxicity. Network analysis shows 9 targets with the degree (K) larger than 40, including LMNA, CYP3A4, MAPT, ALDH1A1, HSD17B10, TP53, ALOX15, HPGD, and CYP2C19. Among them, LMNA (*K* = 59) exhibits the largest number of connected compounds, followed by CYP3A4 (*K* = 56) and MAPT (*K* = 55). According to *in vitro* studies, CYP3A4 may play an important role in liver toxicity (Kostrubsky et al., 1995, 1997a,b; Zhang et al., 2010; Zhou et al., 2017, Li et al., 2018). For instance, Dictamnine (DTN), the main alkaloid from a herb called Dictamni Cortex (DC), has been reported to induce liver injury through the regulation of CYP3A4 (Li et al., 2018). The inhibition of CYP3A4 could alleviate the toxicity both *in vitro* and *in vivo* while induction of CYP3A4 was able to aggravate the toxicity effects. Accordingly, it is likely that the hepatotoxic compounds take effects through the regulation of these targets with higher degree, which deserves to be validated by *in vivo* or *in vitro* experiments. The detailed information for the C-T network is provided in Supplementary Table S5.

**Table 3:** Enriched KEGG pathways of the top 20 targets.

GOID	Pathway	*P*-value	Nr. Genes
GO:0005230	Central carbon metabolism in cancer	0.00052147	3.00
GO:0000590	Arachidonic acid metabolism	0.000453637	3.00
GO:0000591	Linoleic acid metabolism	6.26843E-07	4.00
GO:0000830	Retinol metabolism	0.00052147	3.00
GO:0000980	Metabolism of xenobiotics by cytochrome P450	0.000763064	3.00
GO:0000982	Drug metabolism-cytochrome P450 pathway	2.26974E-05	4.00
GO:0004726	Serotonergic synapse	6.36867E-06	5.00

*Nr. denotes the number of associated genes found in specific pathway.*

In addition, KEGG pathway enrichment analysis by utilizing Cluego (a plugin in Cytoscape) was proposed. Here, we choose the top 20 targets (*D* ≥ 28) in the C-T network. As shown in Table 3, the top 20 targets are enriched in 7 pathways. Among them, 5 out of 7 pathways have been experimentally validated involved with liver injury, including arachidonic acid metabolism pathway (Holownia et al., 2014) (*P* = 4.5 × 10^−4^), linoleic acid metabolism pathway (Lu et al., 2014) (*P* = 6.3 × 10^−7^), retinol metabolism pathway (Freund and Gotthardt, 2017) (*P* = 5.2 × 10^−4^), metabolism of xenobiotics by cytochrome P450 pathway (Gonzalez, 2005) (*P* = 7.6 × 10^−4^), and drug metabolism-cytochrome P450 pathway (Gabbia et al., 2017) (*P* = 2.3 × 10^−7^). Taking retinol metabolism pathway as an example, the pathway has been demonstrated to regulate hepatic immunological response to cholestatic injury and alleviate hepatic fibrosis in different rodent models (Freund and Gotthardt, 2017). Interestingly, there are two pathways (metabolism of xenobiotics by cytochrome P450 pathway and drug metabolism-cytochrome P450 pathway) both relevant with cytochrome P450 (CYP450), which indicates CYP450 may play a key role in HILI. According to experimental studies, lots of CYP450 related enzymes have been validated to involve with the hepatotoxicity of TCM, such as the CYP2E1 enzyme (Gonzalez, 2005), CYP2A6 enzyme (Yamaguchi et al., 2013) and CYP2A8 enzyme (Albassam et al., 2015). Therefore, these pathways are more likely to be regulated by hepatotoxic ingredients of TCM and thus induce liver injury, which merits further investigation by experimental assays.

#### Case Study:Exploring the Molecular Mechanism of *Bupleurum L.* (*Chaihu*) Against HILI

**FIGURE 6:**
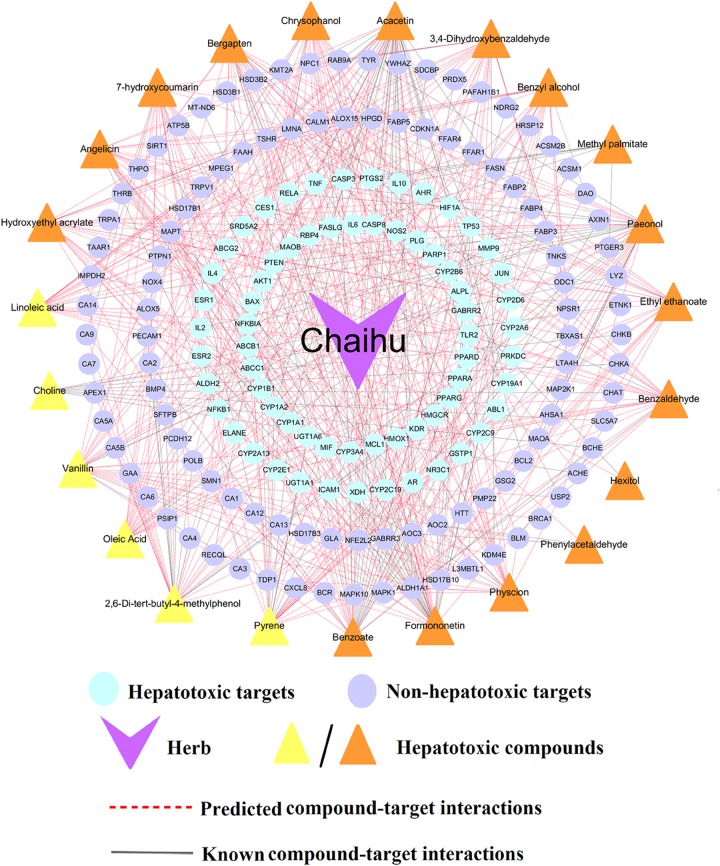
A compound-target (C-T) network of *Bupleurum L.* (*Chaihu*) contains 133 known and 380 predicted C-T interactions connecting 23 compounds and 172 targets (including 66 hepatotoxic targets and 106 non-hepatotoxic targets). The yellow triangles represent the predicted hepatotoxic compounds validated by literatures, while the orange triangles stand for the predicted hepatotoxic compounds without literature evidences.

*Bupleurum L.* (*Chaihu*) was selected as the case study for exploring the molecular mechanism of hepatotoxicity. Through integration of the known and putative targets of the potential hepatotoxic compounds, we found that 23 compounds of *Bupleurum L.* possessed compound-target interactions and selected them to construct the compound-target (C-T) network of *Bupleurum L*. Figure 6 shows a specific compound-target (C-T) network of *Bupleurum L.*, which contains 133 known and 380 predicted C-T interactions connecting 23 compounds and 172 targets. Among the 23 compounds, the average number of non-hepatotoxic and hepatotoxic targets for each compound is 4.6 and 2.8, respectively. The network indicates that CID5280442 (Acacetin, *D* = 44) has the largest number of target connections, followed by CID5280378 (Formononetin, *D* = 36) and CID11092 (Paeonol, *D* = 35).

Further literature analyses suggested that 6 out of 23 compounds, including Linoleic acid, Vanillin, Oleic acid, 2,6-Di-tert-butyl-4-methylphenol, Pyrene and Choline, had been reported with confirmed hepatotoxicity (Supplementary Table S6). For instance, Linoleic acid (CID5460332), a polyunsaturated omega-6 fatty acid, could significantly increase the protein levels of hepatic ALOX15 in Hepa-1c1c7 cells, which contributed to the pathogenesis of alcohol-induced liver injury (Zhang et al., 2017). Additionally, Linoleic acid has been reported to cause oxidative damage (Tong et al., 2016; Li et al., 2019; Qu et al., 2019; Zhang et al., 2019) and mediate selective loss of intrahepatic CD4(+) T lymphocytes, which leads to accelerated hepatocarcinogenesis (Ma et al., 2016). Several clinical case reports also indicate that linoleic acid may induce hepatic disease, such as acute hepatitis (Ramos et al., 2009; Rita and José, 2012; Mohammad et al., 2015). These results were also consistent with its highest frequency (*n* = 8) in top 10 herbs aforementioned. Another study showed high choline (CID305) caused oxidative damage, significant dyslipidemia and liver injury in mice (Ren et al., 2016). Additionally, 2,6-Di-tert-butyl-4-methylphenol (Butylated hydroxytoluene, CID31404), a lung toxicant, was reported to produce liver injury in mice with depressed hepatic GSH levels (Mizutani et al., 1987) and a report indicated that a single dose of BHT (1000 mg/kg) to rats could induce a hepatic injury accompanying centrilobular necrosis (Nakagawa et al., 1984). Taken together, these investigations demonstrate the high prediction accuracy of our consensus model C-3, showing promise for the identification of hepatotoxic compounds.

**FIGURE 7:**
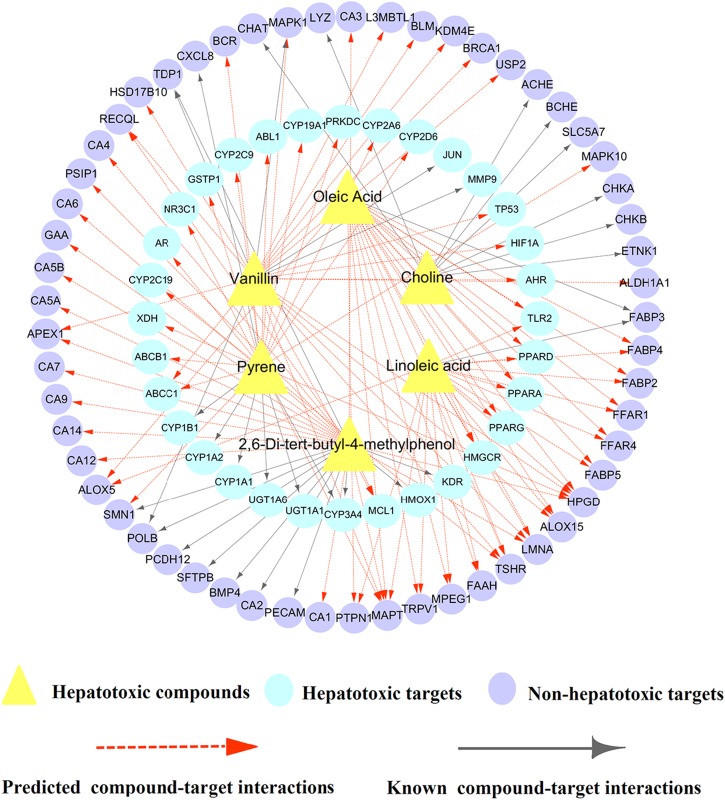
A biqartite compound-target (C-T) network for 6 hepatotoxic compounds including 33 known and 97 predicted CTIs connecting 6 hepatotoxic compounds and 89 targets (including 32 hepatotoxic targets and 57 non-hepatotoxic targets).

To further clarify the detailed molecular mechanism of 6 validated hepatotoxic compounds against HILI, we constructed a specific C-T network for these 6 compounds. As illustrated in Figure 7, the specific C-T network is composed of 33 known CTIs and 97 predicted CTIs connecting 6 compounds and 89 targets (including 32 hepatotoxic targets and 57 non-hepatotoxic targets). For instance, Linoleic acid (LA) interacts with 1 known targets and 20 computationally predicted targets. Among the 21 targets, 5 out of them (TLR2, PPARD, PPARA, PPARG, HMGCR) are hepatotoxic targets, providing potential hepatotoxicity mechanism of LA. Similarly, Pyrene binds with 27 targets, including 7 known targets and 20 predicted targets. Among the 27 targets, 19 out of them are associated with liver toxicity, including 13 hepatotoxic targets predicted as well as 6 known hepatotoxic targets. For example, CYP2C19, one of the 13 hepatotoxic targets predicted, has been reported to mediate the hepatotoxicity of rhein as well as be implicated in the mechanism of clopidogrel-induced hepatotoxicity (He et al., 2015; Zhai et al., 2016). It is likely that Pyrene induces liver injury through regulation of these targets that deserves to be validated by experiments, which indicates new liver toxicity mechanism of Pyrene.

Overall, aforementioned examples indicate that systems pharmacology-based network analyses provide a new perspective for exploring the hepatotoxicity mechanisms of TCM. In the future, experimental validation combined with epidemiologic studies will be introduced to further validate our discovery of HILI and its underlining mechanisms.

## Conclusion

In this study, consensus models were developed by integrating the best four single classifiers to improve the predictive capability of HILI. Based on the evaluated results of withdrawn drugs, we found that C-3 exhibited excellent performance in contrast to the hepatotoxic prediction of ADME/T Descriptor module in DS. Subsequently, C-3 model was utilized to identify potential hepatotoxic ingredients from TCMSP. Furthermore, systems pharmacology analyses and KEGG pathway enrichment were proposed to decipher the hepatotoxicity mechanisms of hepatotoxic ingredients among the top 10 herbs. Finally, we exemplified molecular mechanism of HILI by a case study of *Bupleurum L.* (*Chaihu*).

However, several shortcomings should be recognized in the presented study. First, due to lacking of sufficient quantitative data from clinical and non-clinical (*in vitro* and *in vivo*) studies, the current predictive models are qualitative rather than quantitative models and mainly aimed at the prediction of intrinsic HILI type. As the increasing number of hepatotoxic compounds with quantitative pharmacological data are reported, we intend to develop the quantitative predictive models in the future. Second, as the intrinsic interactions on different ingredients are complicated and unobtainable from public resources, thus it is difficult to explore the specific mixture activity and toxicity of plant ingredients just through this research. Moreover, integration of biological descriptors from drug-target networks (Cheng and Zhao, 2014) and clinical data from multiple sources, such as Drug Induced Liver Injury Rank (DILIrank) dataset (Chen et al., 2016) and Roussel Uclaf Causality Assessment Method (RUCAM) (Danan and Teschke, 2015), will be utilized to further analyze the causal relationship of HILI and improve the application domain of current study. Last, current study have not considered antagonistic or agonistic effects of compound–targets pairs. In the future, specific biological functions (upregulation or downregulation) will be integrated into current network by fetching specific information (upregulation or downregulation) from Connectivity Map (CMap) and LINCs databases.

In summary, this study provides a high accurate approach to predict the herb-induced liver toxicity and systematically explore the hepatotoxicity mechanism of TCM, which facilitates the discovery and development of new drugs.

## Author Contributions

JF and ZK conceived and designed the experiments. QWu and CC wrote the manuscript. PG, MC, JZ, YL, and YZ data acquired and interpreted. XW, A-lL, QWa, and JF reviewed and revised the manuscript.

## Conflict of Interest Statement

The authors declare that the research was conducted in the absence of any commercial or financial relationships that could be construed as a potential conflict of interest.

## Footnotes

1 http://www.ncbi.nlm.nih.gov/

2 https://www.uniprot.org
