# Supplementary material for: In silico Identification and Mechanism Exploration of Hepatotoxic Ingredients in Traditional Chinese Medicine
Source: Front Pharmacol. 2019 May 3;10:458. doi: 10.3389/fphar.2019.00458 (PMC6509242; doi:10.3389/fphar.2019.00458)
Supplement: Supplementary file 1 [file Presentation_1.zip › Supporting Information.doc]

*In silico* identification and mechanism exploration of hepatotoxic ingredients in Traditional Chinese Medicine

Qihui Wu^1,2,3#^, Chuipu Cai^1,2#^, Pengfei Guo^1^, Meiling Chen^2^, Xiaoqin Wu^4^, Jingwei Zhou^1^, Yunxia Luo^1^, Yidan Zou,^2^ Ai-lin Liu^5^, Qi Wang^1^, Zaoyuan Kuang^2^* and Jiansong Fang^1,4^*

**Table of Contents**

**Table S1.** Structures of the 1812 compounds (in SMILE format) used for the training set together with their activities (0 or 1).

**Table S2.** Structures of the 664 compounds (in SMILE format) used for the test set together with their activities (0 or 1).

**Table S3.** Detailed prediction results of 13,139 compounds from TCMSP by the consensus model 3 (C-3) with different threshold value of predicted hepatotoxic probability. (.xlsx)

**Table S4.** Structures of 1,042 hepatotoxic compounds (in SMILE format) in top 10 herbs predicted by consensus model 3 (C-3).

**Table S5.** Detailed information for C-T network.

**Table S6.** Detailed information for the 6 hepatotoxic compounds validated by literatures.

**Figure S1.** Chemical space analysis of training set and test set.

**Figure S2.** Chemical space analysis of compounds in TCMSP.

**Table S1.** Structures of the 1812 compounds (in SMILE format) used for the training set together with their activities (0 or 1).

| **mol** | **Activity** |
| --- | --- |
| ClCCN(P1(=O)OCCC(O)N1)CCCl | 1 |
| O1C(CCc2c1c(c(c(O)c2C)C)C)(C)C | 1 |
| O1CC(CCCCc2nc(oc2C)c2ccc(cc2)C)COC1(C)C(=O)[O-] | 1 |
| ClC(Cl)(CCCCCCCCCCc1ccc(Cl)cc1)C(=O)[O-] | 1 |
| ClC1=C[C@@H]2[C@@H]([C@]3([C@@H]4[C@@H](C4)C(=O)C=C13)C)CC[C@]1([C@H]2CC[C@]1(OC(=O)C)C(=O)C)C | 1 |
| Clc1sc(C(=O)NC[C@@H]2OC(=O)N(C2)c2ccc(N3CCOCC3=O)cc2)cc1 | 1 |
| O=CCCN(N=O)C | 1 |
| Clc1ccc(N2[C@H](OC(=O)N3CC[N@H+](CC3)C)c3nccnc3C2=O)nc1 | 1 |
| O1[C@H]2[C@]3(c4c1c(OC)ccc4C[N@@H+](CC3)C)C=C[C@@H](O)C2 | 1 |
| S(=O)(Cc1ncc(c(OC)c1C)C)c1[nH]c2c(n1)ccc(OC)c2 | 1 |
| O(CCCCCC)C(=O)/N=C(/N)\c1ccc(NCc2n(c3c(n2)cc(cc3)C(=O)N(CCC(=O)OCC)c2ncccc2)C)cc1 | 1 |
| S(=O)(Cc1nccc(OCC(F)(F)F)c1C)c1[nH]c2c(n1)cccc2 | 1 |
| S(=O)(Cc1ncc(c(OC)c1C)C)c1nc2c([n-]1)ccc(OC)c2 | 1 |
| Brc1c(Oc2c(Br)cc(Br)cc2)ccc(Br)c1 | 1 |
| O1[C@@H]([C@@H](O)[C@@H](O)[C@@H]1n1c(=O)nc(nc1)N)CO | 1 |
| O([C@@H]1[C@@]2([C@H]([C@H]3[C@@H]([C@@]4(C(=CC(=O)CC4)CC3)C)CC2)CC1)C)C(=O)CCCCCC | 1 |
| ClC(Cl)C(F)(F)F | 1 |
| P(=O)(OC1C(O)C(OC1n1c2ncnc(N)c2nc1)COP(=O)(OP(=O)(OCC1OC([n+]2cc(ccc2)C(=O)N)C(O)C1O)[O-])[O-])([O-])[O-] | 1 |
| o1c2c(cccc2)cc1 | 1 |
| O(c1c(cccc1)C(=O)[O-])C(=O)C | 1 |
| BrC(COP(=O)(OCC(Br)CBr)[O-])CBr | 1 |
| c12c3c4c(c1cccc2c1c(c3)cccc1)cccc4 | 1 |
| [nH]1c2c(c3c1ccc1c3cccc1)c1c(cc2)cccc1 | 1 |
| Oc1c(cc(NC(=O)C)cc1C)C | 1 |
| Clc1c2c(c3c(cc2)cccc3)cc2c1cccc2 | 1 |
| O(Cc1ccccc1)C(=O)C | 1 |
| S(c1ccc(N)cc1)c1ccc(N)cc1 | 1 |
| [O-]C(=O)[C@@H]([NH3+])CCCC[NH3+] | 1 |
| Clc1c(n(nc1CC)C)C(=O)NCc1ccc(C(C)(C)C)cc1 | 1 |
| Ic1cc(C[C@H]([NH3+])C(=O)[O-])cc(I)c1Oc1cc(I)c(O)c(I)c1 | 1 |
| OC(C[N+](C)(C)C)CC(=O)[O-] | 1 |
| Clc1c(N)ccc([N+](=O)[O-])c1 | 1 |
| [O-][N+](=O)c1c(ccc([N+](=O)[O-])c1)C | 1 |
| O([C@H]1[C@@H]([C@H](O[C@H]2O[C@@H]([C@@H](O)[C@@](OC)(C2)C)C)C(C(=O)O[C@@H]([C@@](O)([C@@H](O)[C@@H](C(=O)[C@H](C[C@]1(OC)C)C)C)C)CC)C)C)[C@H]1O[C@@H](C[C@@H]([NH+](C)C)[C@H]1O)C | 1 |
| OCCN(N=O)C(=O)N | 1 |
| Oc1cc(C[C@H]([NH3+])C(=O)[O-])ccc1O | 1 |
| O=C1c2c(C(=O)c3c1cccc3)ccc(N)c2 | 1 |
| [Ge](=O)(O[Ge](=O)CCC(=O)[O-])CCC(=O)[O-] | 1 |
| [O-]C(=O)c1ccc(C(=C)c2cc3C(CCC(c3cc2C)(C)C)(C)C)cc1 | 1 |
| O([C@H]1[C@H](O)[C@H](O[C@H]2O[C@@H]([C@@H](O)[C@@H](O)[C@H]2O)C[NH3+])[C@H]([NH3+])C[C@H]1[NH3+])[C@H]1O[C@@H]([C@@H](O)[C@@H]([NH3+])[C@H]1O)CO | 1 |
| O=C(CCC(=O)C)C | 1 |
| Brc1ccccc1 | 1 |
| ClCC(OC(CCl)C)C | 1 |
| Brc1cc(Br)ccc1 | 1 |
| BrCCC | 1 |
| S1/C(=C/c2cc(OC)c(Oc3c(cc(cc3)C#N)C(F)(F)F)cc2)/C(=O)[N-]C1=O | 1 |
| S1C(Cc2ccc(OCCN(c3ncccc3)C)cc2)C(=O)[N-]C1=O | 1 |
| Nc1ccc(Cc2ccc(N)cc2)cc1 | 1 |
| Clc1ccc(C2CCC(CC2)C2=C([O-])c3c(C(=O)C2=O)cccc3)cc1 | 1 |
| [O-][N+](=O)c1ccc(cc1)C | 1 |
| [O-][N+](=O)c1cc(ccc1)C | 1 |
| P(=O)([O-])([O-])[C@H]1O[C@H]1C | 1 |
| N(=N\c1c(cccc1)C)/c1cc(c(N)cc1)C | 1 |
| ClCC(O)CCl | 1 |
| ClC1C(Cl)C(Cl)C(Cl)C(Cl)C1Cl | 1 |
| Nc1c(ccc(N)c1)C | 1 |
| c1(c(cccc1)C)C | 1 |
| O=C(N1[C@H]2[C@@H](C[C@H]1C(=O)[O-])CCCC2)[C@H]([NH2+][C@@H](CCC)C(=O)[O-])C | 1 |
| Clc1c(Nc2c(CC(=O)OCC(=O)[O-])cccc2)c(Cl)ccc1 | 1 |
| Fc1c([C@](O)([C@@H](C)c2ncncc2F)Cn2ncnc2)ccc(F)c1 | 1 |
| Clc1c(OCCN(CCC)C(=O)n2ccnc2)c(Cl)cc(Cl)c1 | 1 |
| S(=O)(=O)([O-])CCCNC(=O)C | 1 |
| Nc1ccc(c2ccccc2)cc1 | 1 |
| Clc1cc([N+](=O)[O-])c(N)cc1 | 1 |
| [O-][N+](=O)c1c(cccc1)C | 1 |
| O[C@@]12C[C@]3([NH2+]CC(=O)N4[C@@H](CCC4)C#N)C[C@@H](C1)C[C@@H](C3)C2 | 1 |
| O(C([C@H](Oc1nc(cc(n1)C)C)C(=O)[O-])(c1ccccc1)c1ccccc1)C | 1 |
| s1c(N(CC(=O)[O-])CC(=O)[O-])c(c(c1C(=O)[O-])CC(=O)[O-])C#N | 1 |
| S1[C@H]2N(C(=O)[C@H]2NC(=O)/C(=N\O)/c2nc(sc2)N)C(=C(C1)C=C)C(=O)[O-] | 1 |
| Nc1c(cccc1C)C | 1 |
| O1O[C@]2(O[C@H]3OC(=O)[C@@H]([C@H]4[C@@]13[C@H]([C@@H](CC4)C)CC2)C)C | 1 |
| [O-][N+](=O)c1c2c(ccc1)cccc2 | 1 |
| Oc1cc(CC[NH3+])ccc1O | 1 |
| P(=O)(O[C@H](OC(=O)CC)C(C)C)(CC(=O)N1C[C@H](C2CCCCC2)C[C@H]1C(=O)[O-])CCCCc1ccccc1 | 1 |
| O=C1c2c(C(=O)c3c1cccc3)ccc(c2N)C | 1 |
| Brc1c2c(C(=O)c3c(C2=O)cccc3)c(N)c(Br)c1 | 1 |
| O=C=Nc1c2c(ccc1)cccc2 | 1 |
| Oc1ccc(C(c2ccc(O)cc2)(C)C)cc1 | 1 |
| [O-]C(=O)c1c(c2ccc(Cn3c4c(nc3CCC)c(cc(c4)c3n(c4c(n3)cccc4)C)C)cc2)cccc1 | 1 |
| ClC(Cl)C(Cl)Cl | 1 |
| O(C(C[N@H+](CCC(c1ccccc1)c1ccccc1)C)(C)C)C(=O)C1=C(NC(=C([C@H]1c1cc([N+](=O)[O-])ccc1)C(=O)OC)C)C | 1 |
| ClC(Cl)CCl | 1 |
| Fc1nc2n([C@@H]3O[C@@H]([C@@H](O)[C@@H]3O)CO)cnc2c(n1)N | 1 |
| O=C(N)c1cc(C)cnc1 | 1 |
| O([C@H]1[C@@H](NC(=O)C)[C@H]([NH3+])CC(=C1)C(=O)OCC)C(CC)CC | 1 |
| Clc1ccc(C(=O)c2ccc(OC(C)(C)C(=O)[O-])cc2)cc1 | 1 |
| S(c1cc2[nH]nc(c2cc1)/C=C/c1ncccc1)c1c(cccc1)C(=O)NC | 1 |
| O=C(CCCCC)/C=C/C=O | 1 |
| FC(F)(F)c1cc(n2cc(nc2)C)cc(NC(=O)c2cc(Nc3nc(c4cccnc4)ccn3)c(cc2)C)c1 | 1 |
| O1[C@H]2[C@]34[C@@]5(C[C@H]([C@]2(OC)CC5)[C@](O)(C(C)(C)C)C)C([N@H+](CC3)CC2CC2)Cc2c4c1c(O)cc2 | 1 |
| Clc1cc([C@@H](O)C[NH+](CCCC)CCCC)c2c(/C(=C\c3ccc(Cl)cc3)/c3c2ccc(Cl)c3)c1 | 1 |
| Clc1cc2[C@@](OC(=O)Nc2cc1)(C#CC1CC1)C(F)(F)F | 1 |
| BrC(Cl)(Cl)Cl | 1 |
| O(c1ccc(cc1)/C=C/C)C | 1 |
| O(c1c(cc(OC)c(OC)c1)/C=C/C)C | 1 |
| BrC(Cl)Cl | 1 |
| S1[C@H]2N(C(=O)[C@H]2NC(=O)/C(=N\OC)/c2nc(sc2)N)C(=C(C1)COC)C(=O)[O-] | 1 |
| S1[C@H]2N(C(=O)[C@H]2NC(=O)/C(=N/OCC(=O)[O-])/c2nc(sc2)N)C(=C(C1)C=C)C(=O)[O-] | 1 |
| Clc1c(c2c(Cl)c(Cl)ccc2)ccc(Cl)c1 | 1 |
| Clc1cc(c2cc(Cl)c(Cl)cc2)cc(Cl)c1Cl | 1 |
| Clc1cc([C@H]2CC[C@H]([NH2+]C)c3c2cccc3)ccc1Cl | 1 |
| Fc1c(N2C[C@H]3[C@@H]([C@H]3[NH3+])C2)nc2n(cc(c(=O)c2c1)C(=O)[O-])c1c(F)cc(F)cc1 | 1 |
| [nH]1c2c(c3c1nc(N)cc3)cccc2 | 1 |
| ClC(Cl)(Cl)C | 1 |
| Ic1cc(C(=O)c2c3c(oc2CC)cccc3)cc(I)c1O | 1 |
| n1(c2c(nc1N)c1nc(cnc1cc2)C)C | 1 |
| [nH]1c2c(c3c1nc(N)c(c3)C)cccc2 | 1 |
| [O-][N+](=O)c1c2c3c4c(c1)cccc4ccc3ccc2 | 1 |
| Br/C=C(/OP(=O)(OCC)OCC)\c1c(Cl)cc(Cl)cc1 | 1 |
| O[C@H]1[C@]23[C@H](N(c4c3cccc4)C)C3[N@H+]4[C@@H]([C@H]1[C@@H](C3)[C@@H]([C@H]4O)CC)C2 | 1 |
| O1[C@@H](n2c3ncnc(N)c3nc2)[C@H](O)[C@H](O)[C@H]1CO | 1 |
| O=C(NC(C)(C)C)[C@H]1[N@H+](C[C@@H]2[C@@H](C1)CCCC2)C[C@H](O)[C@H](NC(=O)[C@H](NC(=O)c1nc2c(cc1)cccc2)CC(=O)N)Cc1ccccc1 | 1 |
| S1C[C@H](O[C@H]1CO)n1cc(F)c(nc1=O)N | 1 |
| [O-]C(=O)[C@@H](N(Cc1ccc(cc1)c1c(cccc1)c1n[n-]nn1)C(=O)CCCC)C(C)C | 1 |
| Fc1ccc(c2n(c(C(C)C)c(c2c2ccccc2)C(=O)Nc2ccccc2)CC[C@@H](O)C[C@@H](O)CC(=O)[O-])cc1 | 1 |
| O(C(=O)[C@@H]([NH3+])C(C)C)CCOCn1c2[nH]c(nc(=O)c2nc1)N | 1 |
| FC1(F)[C@H](O)[C@H](O[C@H]1n1ccc(nc1=O)N)CO | 1 |
| Brc1ccc(C(=O)c2c(N)c(CC(=O)[O-])ccc2)cc1 | 1 |
| s1c(C(=CCC[N@@H+]2C[C@@H](CCC2)C(=O)[O-])c2sccc2C)c(cc1)C | 1 |
| Clc1c([C@H]([N@@H+]2CCc3sccc3C2)C(=O)OC)cccc1 | 1 |
| s1c(C(N([O-])C(=O)N)C)cc2c1cccc2 | 1 |
| Fc1c2n(C3CC3)cc(c(=O)c2c(N)c(F)c1N1C[C@H]([NH2+][C@@H](C1)C)C)C(=O)[O-] | 1 |
| OC(C(O)C(O)C(=O)[O-])C(O)CO | 1 |
| O=C1[C@@]2([C@H]([C@H]3[C@H](CC2)[C@@]2(C(=CC(=O)C=C2)C(=C)C3)C)CC1)C | 1 |
| O=C(N1[C@H]2[C@@H](C[C@H]1C(=O)[O-])CCCC2)[C@H]([NH2+][C@@H](CCC)C(=O)OCC)C | 1 |
| Clc1cc(C2SC(=O)C(=O)N2)cc(Cl)c1 | 1 |
| O[C@@H]1[C@@]2([C@H]([C@H]3[C@@H]([C@@]4(C(=CC(=O)CC4)CC3)C)CC2)CC1)C | 1 |
| O=C([C@@H]1[C@@]2([C@H]([C@H]3[C@@H]([C@@]4(C(=CC(=O)CC4)CC3)C)CC2)CC1)C)C | 1 |
| c12c(cc3c(c2)cccc3)ccc2c1cccc2 | 1 |
| c12c(cc3c(c2)ccc2c3cccc2)ccc2c1cccc2 | 1 |
| O=C1/C(=N\Nc2ccccc2)/c2c(C=C1)cccc2 | 1 |
| O[C@@H]1[C@H]2[C@H]([C@H]3[C@](C1)([C@H](CC3)C(=O)CO)C=O)CCC1=CC(=O)CC[C@]21C | 1 |
| SCC(NC(=O)C)C(=O)[O-] | 1 |
| O=C1C(=O)[N-]C(=O)[N-]C1=O | 1 |
| O[C@@H]1[C@@]2([C@H]([C@H]3[C@H](CC2)c2c(CC3)cc(O)cc2)CC1)C | 1 |
| O1[C@H]2[C@]34[C@@H](C([N@@H+](CC3)C)Cc3c4c1c(OC)cc3)C=C[C@H]2O | 1 |
| n1(CC(C)C)c2c3c(nc(N)c2nc1)cccc3 | 1 |
| S1[C@H]2N(C(=O)[C@H]2NC(=O)/C(=N\OC)/c2nc(sc2)N)C(=C(C1)COC(=O)C)C(=O)[O-] | 1 |
| O=C(N(c1cc(c2n3ncc(c3ncc2)C#N)ccc1)CC)C | 1 |
| OC1([C@@H](C[NH+](C)C)c2ccc(OC)cc2)CCCCC1 | 1 |
| [O-]C(=O)CCCCC[NH3+] | 1 |
| P(=O)(OCN1C(=O)C(NC1=O)(c1ccccc1)c1ccccc1)([O-])[O-] | 1 |
| S(=O)(=O)(Nc1cc2cc([nH]c2cc1)C(=O)N1CCN(CC1)c1ncccc1NC(C)C)C | 1 |
| O(CCc1c([nH+]c(n2ncnc12)N)c1ccccc1)CC | 1 |
| O=C(N(CC)CC)[C@]1([C@H](C1)C[NH3+])c1ccccc1 | 1 |
| Clc1c([C@@]2(O[C@H](CO2)COc2ccc(N3CCN(CC3)c3ccc(n4c(=O)n(nc4)C(CC)C)cc3)cc2)Cn2ncnc2)ccc(Cl)c1 | 1 |
| O[C@@H]([C@@H]([NH3+])C[C@@H](C(C)C)Cc1cc(OCCCOC)c(OC)cc1)C[C@@H](C(C)C)C(=O)NCC(C)(C)C(=O)N | 1 |
| O=C1/C(=c\2/n([nH]/c(=C/3\C(=O)C=CC=C3)/[nH]2)c2ccc(cc2)C(=O)[O-])/C=CC=C1 | 1 |
| Clc1cn2c(CC(=O)N(CCC)CCC)c(nc2cc1)c1ccc(Cl)cc1 | 1 |
| OCc1cc2c(N=C/C/2=C\NN/C(=[NH+]/CCCCC)/N)cc1 | 1 |
| Clc1c(NC2=[NH+]CCN2)c2nsnc2cc1 | 1 |
| [O-]C(=O)C[C@H](CC(C)C)C[NH3+] | 1 |
| O([C@@H](CC[NH2+]C)c1ccccc1)c1c(cccc1)C | 1 |
| P(=O)(OCOC(=O)OC(C)C)(OCOC(=O)OC(C)C)CO[C@@H](Cn1c2ncnc(N)c2nc1)C | 1 |
| S1[C@H]2N(C(=O)[C@H]2NC(=O)/C(=N\OC)/c2occc2)C(=C(C1)COC(=O)N)C(=O)[O-] | 1 |
| O=C(N(CCC[NH+](C)C)C(=O)NCC)[C@H]1C[C@@H]2[C@H]([N@@H+](C1)CC=C)Cc1c3c2cccc3[nH]c1 | 1 |
| O([C@@H]1[C@@H]2[C@H]([C@H](C=CC2=C[C@@H](O)C1)C)CC[C@@H](O)C[C@@H](O)CC(=O)[O-])C(=O)[C@H](CC)C | 1 |
| O[C@@]12[C@@H](C[C@H]3C(=C([O-])c4c(C3)c(N(C)C)cc(NC(=O)C[NH2+]C(C)(C)C)c4O)C1=O)[C@H]([NH+](C)C)C(=O)C(=C2[O-])C(=O)N | 1 |
| FC(F)(F)c1ccc(NC(=O)/C(=C(\[O-])/C)/C#N)cc1 | 1 |
| o1c(=O)c(C(CC(=O)C)c2ccc([N+](=O)[O-])cc2)c([O-])c2c1cccc2 | 1 |
| O[C@H]1[C@H]2[C@](O)(C(=O)C3=C([O-])c4c([C@H]([C@H]13)C)cccc4O)C(=C(C(=O)[C@H]2[NH+](C)C)C(=O)N)[O-] | 1 |
| O1[C@H]([C@@H](O)CO)C(=C(O)C1=O)[O-] | 1 |
| O=C(N1[C@H]2[C@@H](C[C@H]1C(=O)[O-])CCCC2)[C@H]([NH2+][C@@H](CCc1ccccc1)C(=O)[O-])C | 1 |
| Oc1c(cc(N/N=C\2/C=C(C(=O)C=C2)C(=O)[O-])cc1)C(=O)[O-] | 1 |
| S1c2c(N(CC[C@H]3[N@H+](CCCC3)C)c3c1cccc3)cc(SC)cc2 | 1 |
| O([C@@H]1[C@@H]2[C@H]([C@H](C=CC2=C[C@@H](C1)C)C)CC[C@H]1OC(=O)C[C@H](O)C1)C(=O)C(CC)(C)C | 1 |
| O=C1NC(=O)CCC1N1C(=O)c2c(C1=O)cccc2 | 1 |
| O=c1n2c(nnn1C)c(nc2)C(=O)N | 1 |
| Fc1c(N2C[C@H]([NH2+]CC2)C)c(OC)c2n(C3CC3)cc(c(=O)c2c1)C(=O)[O-] | 1 |
| ClC1=C(C(OC1=O)O)C(Cl)Cl | 1 |
| O[C@@H](C[N@@H+]1[C@@H](C[N@@H+](CC1)Cc1cccnc1)C(=O)NC(C)(C)C)C[C@@H](C(=O)N[C@H]1[C@H](O)Cc2c1cccc2)Cc1ccccc1 | 1 |
| O=C(N1[C@H]2[C@@H](C[C@H]1C(=O)[O-])CCC2)[C@H]([NH2+][C@@H](CCc1ccccc1)C(=O)OCC)C | 1 |
| O=C(NCCC(=O)[O-])c1ccc(N/N=C\2/C=C(C(=O)C=C2)C(=O)[O-])cc1 | 1 |
| S1[C@H]2N(C(=O)[C@H]2NC(=O)/C(=N/OC(C)(C)C(=O)[O-])/c2nc(sc2)N)C(=C(C1)C[n+]1ccccc1)C(=O)[O-] | 1 |
| O1[C@H]2[C@]34[C@](O)(C([N@H+](CC3)CC3CC3)Cc3c4c1c(O)cc3)CCC2=O | 1 |
| S(=O)(=O)(Nc1ncccc1)c1ccc(N/N=C\2/C=C(C(=O)C=C2)C(=O)[O-])cc1 | 1 |
| S(=O)(=O)(NC)Cc1cc2c(CC[NH+](C)C)c[nH]c2cc1 | 1 |
| n1(c2c(nc1N)c1c(nccc1)cc2)C | 1 |
| O1[C@H]2[C@H]3O[C@H]4[C@H]5O[C@@H](CC4)CC(=O)C[C@H]4[C@H](O[C@@H]([C@H]4OC)C[C@H](O)C[NH3+])C[C@@H]4O[C@@H](CC[C@H]6O[C@@H](CC[C@]7(O[C@@H]3[C@H]1C7)O[C@@H]25)CC6=C)C[C@@H](C4=C)C | 1 |
| FC(F)(F)c1ccc(/C(=N/OCC[NH3+])/CCCCOC)cc1 | 1 |
| [O-][N+](=O)c1c2c3c4c(ccc3c([N+](=O)[O-])c1)cccc4cc2 | 1 |
| FC(F)(F)COc1c(OCC[NH2+][C@@H](Cc2cc3c(N(CC3)CCCO)c(c2)C(=O)N)C)cccc1 | 1 |
| O[C@H]1[C@@H]([C@H]([C@@H](O)C1)C/C=C\CCCC(=O)OC(C)C)CC[C@@H](O)CCc1ccccc1 | 1 |
| [nH]1c([C@H](c2c(c(ccc2)C)C)C)cnc1 | 1 |
| O=C(Nc1cc(Nc2nc(c3cccnc3)ccn2)c(cc1)C)c1ccc(C[N@@H+]2CC[N@H+](CC2)C)cc1 | 1 |
| S(=O)(=O)(OC[C@]12OC[C@H]3OC(O[C@H]3[C@@H]2OC(O1)(C)C)(C)C)N | 1 |
| O=C1N(CCC1)[C@@H](CC)C(=O)N | 1 |
| ClC1=C[C@@H]2[C@@H]([C@]3([C@@H]4[C@@H](C4)C(=O)C=C13)C)CC[C@]1([C@H]2CC[C@]1(O)C(=O)C)C | 1 |
| O(c1c(c(c(c(c1)C)/C=C/C(=C/C=C/C(=C/C(=O)[O-])/C)/C)C)C)C | 1 |
| [nH]1c2c(c3c1cccc3)c(nc(N)c2C)C | 1 |
| O[C@@H]1[C@@H]([C@H](C(=O)C1)CCC(=O)CCCCC)C/C=C\CCCC(=O)[O-] | 1 |
| Fc1ccc(c2c(c(nc3c2cccc3)C2CC2)/C=C/[C@@H](O)C[C@@H](O)CC(=O)[O-])cc1 | 1 |
| [O-]C(=O)/C=C(\C=C\C=C(\C=C\C1=C(CCCC1(C)C)C)/C)/C | 1 |
| O[C@@H]1[C@H]([C@H]([C@H](O)C1)/C=C/[C@@H](O)CCCCC)C/C=C\CCCC(=O)[O-] | 1 |
| Fc1c(COC(=O)C2C(C2/C=C/C)(C)C)c(F)c(F)c(c1F)COC | 1 |
| O=C(NCCO)CCC/C=C\C/C=C\C/C=C\C/C=C\CCCCC | 1 |
| O=C(N(CC)CC)/C(=C/c1cc([N+](=O)[O-])c(O)c(O)c1)/C#N | 1 |
| Clc1cc2nc(/C=C/c3cc([C@H](SCC4(CC4)CC(=O)[O-])CCc4c(C(O)(C)C)cccc4)ccc3)ccc2cc1 | 1 |
| O1[C@]2([C@@]3([C@H]([C@H]4[C@H]([C@@H](O)C3)[C@@]3(C(=CC(=O)C=C3)CC4)C)C[C@H]2OC1CCC)C)C(=O)CO | 1 |
| o1c2c(c(=O)cc1c1ccc(O)cc1)c(O)cc(O)c2 | 1 |
| [O-]C(=O)CCc1c([nH]c(c1C)/C=C/1\NC(=O)C(=C1C)C=C)Cc1[nH]c(c(c1CCC(=O)[O-])C)/C=C/1\NC(=O)C(=C1C=C)C | 1 |
| FC(F)(F)C(OCF)C(F)(F)F | 1 |
| [O-]/[N+](=N\CC)/CC | 1 |
| ClC1=C(N2[C@H](SC1)[C@H](NC(=O)[C@H]([NH3+])c1ccccc1)C2=O)C(=O)[O-] | 1 |
| O=C1Nc2c(c(CC[NH+](CCC)CCC)ccc2)C1 | 1 |
| s1c2c(nc1N)ccc(OC(F)(F)F)c2 | 1 |
| O1[C@@H](n2c3[nH]cnc(=O)c3nc2)CC[C@H]1CO | 1 |
| S(=O)(Cc1nccc(OCCCOC)c1C)c1[nH]c2c(n1)cccc2 | 1 |
| n12c(nc3c1nc(N)cc3)c(ccc2)C | 1 |
| O(C[C@@H](O)C[NH2+]CCC)c1c(C(=O)CCc2ccccc2)cccc1 | 1 |
| S1C(Cc2ccc(OCCc3ncc(CC)cc3)cc2)C(=O)[N-]C1=O | 1 |
| S(C[C@H]1C[C@@H]2[C@H]([N@@H+](C1)CCC)Cc1c3c2cccc3[nH]c1)C | 1 |
| O([N+](=O)[O-])CCNC(=O)c1cccnc1 | 1 |
| O=C(CCCN(N=O)C)c1cccnc1 | 1 |
| OCC(CCn1c2[nH]c(nc(=O)c2nc1)N)CO | 1 |
| Oc1c(ccc(N)c1)C(=O)[O-] | 1 |
| P(=O)([O-])([O-])CO[C@@H](Cn1c2ncnc(N)c2nc1)C | 1 |
| s1c(N[C@H]2[C@H](O)CCCC2)nc2c1cc(Oc1cc(ncc1)C(=O)NC)cc2 | 1 |
| [NH-]C1C([NH-])CCCC1 | 1 |
| ClCCN(c1ccc(C[C@H]([NH3+])C(=O)[O-])cc1)CCCl | 1 |
| Clc1c([C@@]2(O[C@H](CO2)COc2ccc(N3CCN(CC3)C(=O)C)cc2)Cn2ccnc2)ccc(Cl)c1 | 1 |
| Fc1c(N2CC[NH2+]CC2)cc2n(CC)cc(c(=O)c2c1)C(=O)[O-] | 1 |
| OC(C(O)C(O)CO)C(O)CO | 1 |
| s1c([C@H]2O[C@H]3[C@H](O)[C@@H](O)[C@H](O[C@H]4[C@@H]5[C@@H]([C@@H](c6c4cc4OCOc4c6)c4cc(OC)c(O)c(OC)c4)C(=O)OC5)O[C@@H]3CO2)ccc1 | 1 |
| O(C(=O)C1=C(NC(=C(C1c1cc([N+](=O)[O-])ccc1)C(=O)OCCOC)C)C)C(C)C | 1 |
| [O-]C(=O)/C=C(/C=C/C=C(\C=C\C1=C(CCCC1(C)C)C)/C)\C | 1 |
| O([C@H]1[C@@H]([C@H](O[C@H]2O[C@@H]([C@@H](O)[C@@](OC)(C2)C)C)[C@@H](C(=O)O[C@@H]([C@@](O)([C@@H](O)[C@H]([N@@H+](C[C@H](C[C@]1(O)C)C)C)C)C)CC)C)C)[C@H]1O[C@@H](C[C@@H]([NH+](C)C)[C@H]1O)C | 1 |
| O1Cc2c(c(O)c(c(OC)c2C)C/C=C(/CCC(=O)[O-])\C)C1=O | 1 |
| O=c1[nH]c2c(n(C3CC3)c3ncccc13)nccc2C | 1 |
| Fc1ccc(c2c(n(C(C)C)c3c2cccc3)/C=C/[C@@H](O)C[C@@H](O)CC(=O)[O-])cc1 | 1 |
| [O-]C(=O)/C=C(/C=C/C=C(/C=C/C1=C(CCCC1(C)C)C)\C)\C | 1 |
| O([C@H]1[C@H](O)[C@H](O)[C@@H](O[C@@H]1CO)O[C@H]1[C@H](O)[C@H](O)[C@@H](O[C@@H]1CO)O)[C@H]1O[C@@H]([C@@H]([NH2+][C@H]2[C@H](O)[C@H](O)[C@H](O)C(=C2)CO)[C@H](O)[C@@H]1O)C | 1 |
| S(=O)(=O)(NC)CCc1cc2c([C@H]3CC[N@H+](CC3)C)c[nH]c2cc1 | 1 |
| [O-][N+](=O)c1c2c3c4c(ccc3c3c1cccc3)cccc4cc2 | 1 |
| Fc1c(N2CCOCC2)ccc(N2C[C@@H](OC2=O)CNC(=O)C)c1 | 1 |
| OC[C@H]1C[C@@H](n2c3nc(nc(NC4CC4)c3nc2)N)C=C1 | 1 |
| O1[C@@H](n2c3NC=[NH+]C[C@@H](O)c3nc2)C[C@H](O)[C@H]1CO | 1 |
| O[C@@H]1C2(C(C3C(C4([C@H](C[C@H]3O)C[C@H](O)CC4)C)C1)CCC2C(CCC(=O)[O-])C)C | 1 |
| Fc1ccc([C@H]2[C@@H](C[NH2+]CC2)COc2cc3OCOc3cc2)cc1 | 1 |
| O(C(c1ccc(OC)cc1)C=C)C(=O)C | 1 |
| S1[C@H]2N([C@H](C1(C)C)C(=O)[O-])C(=O)[C@H]2NC(=O)[C@H](NC(=O)N1CCN(C(=O)C1=O)CC)c1ccccc1 | 1 |
| Brc1c(c2c(Br)cc(Br)c(Br)c2)cc(Br)c(Br)c1 | 1 |
| [O-]C(=O)[C@@H]([NH3+])CC(=O)[O-] | 1 |
| Clc1c2oc3c(c2cc(Cl)c1Cl)cc(Cl)c(Cl)c3 | 1 |
| FC(OC(F)F)C(F)(F)F | 1 |
| N12[C@@H](C[N@H+](CC1)C)c1c(Cc3c2nccc3)cccc1 | 1 |
| O(C[C@@H](O)C[NH2+]C(C)C)c1ccc(CCOC)cc1 | 1 |
| O(C(=O)[C@@H]([C@H]1[NH2+]CCCC1)c1ccccc1)C | 1 |
| N(C)(C)C(=[NH2+])[NH+]=C(N)N | 1 |
| Oc1c(cc(N)cc1)C(=O)[O-] | 1 |
| FC(F)(F)c1nc2c(c([C@@H](O)[C@H]3[NH2+]CCCC3)c1)cccc2C(F)(F)F | 1 |
| S1(=O)(=O)N(C(=C([O-])c2c1cccc2)C(=O)Nc1sc(cn1)C)C | 1 |
| OC(c1ccc(OC)cc1)C=C | 1 |
| Clc1cc2c3c(oc2cc1Cl)cc(Cl)c(Cl)c3 | 1 |
| Clc1nc(n(Cc2ccc(cc2)c2c(cccc2)c2n[n-]nn2)c1CO)CCCC | 1 |
| Fc1c(N2C[C@H]([NH2+]CC2)C)c(F)cc2c1n(CC)cc(c2=O)C(=O)[O-] | 1 |
| [O-][N+](=O)c1c2c3c4c(cc2)c([N+](=O)[O-])ccc4ccc3cc1 | 1 |
| Clc1ccc(C(=O)NCCc2ccc(OC(C)(C)C(=O)[O-])cc2)cc1 | 1 |
| n1(ncnc1)C(c1ccc(cc1)C#N)c1ccc(cc1)C#N | 1 |
| O=C(N[C@@H](CC(C)C)B(O)O)[C@@H](NC(=O)c1nccnc1)Cc1ccccc1 | 1 |
| [O-]C(=O)C1CCn2c1ccc2C(=O)c1ccccc1 | 1 |
| Clc1cc(c2cc(Cl)ccc2)cc(Cl)c1 | 1 |
| Clc1c(c2c(Cl)c(Cl)c(Cl)cc2)ccc(Cl)c1Cl | 1 |
| S(=O)(=O)(O[C@H]1[C@H](O[C@@H]2OC[C@@H](O)[C@H](OS(=O)(=O)[O-])[C@H]2OS(=O)(=O)[O-])CO[C@@H](O)[C@@H]1OS(=O)(=O)[O-])[O-] | 1 |
| ClC(OC(F)F)C(F)(F)F | 1 |
| O1[C@@H]([C@@H](O)[C@@H](O)[C@@H]1n1nc(nc1)C(=O)N)CO | 1 |
| O=C1N(C(=NC21CCCC2)CCCC)Cc1ccc(cc1)c1c(cccc1)c1n[n-]nn1 | 1 |
| [O-][N+](=O)c1c(c(N)cc([N+](=O)[O-])c1)C | 1 |
| Clc1c(c2c(Cl)cc(Cl)c(Cl)c2)cc(Cl)c(Cl)c1 | 1 |
| ClCCN1P(=O)(OCCC1)NCCCl | 1 |
| O1c2cc(C(OC(=O)C)C=C)ccc2OC1 | 1 |
| O([C@H]1[C@@H]2[C@@H]([C@@H](c3c1cc1OCOc1c3)c1cc(OC)c(O)c(OC)c1)C(=O)OC2)[C@@H]1O[C@H]2[C@@H](O[C@@H](OC2)C)[C@H](O)[C@H]1O | 1 |
| N(N)c1nncc2c1cccc2 | 1 |
| Brc1c(Oc2c(Br)cc(Br)cc2)cc(Br)c(Br)c1 | 1 |
| O(c1cc(Cc2c(nc(nc2)N)N)cc(OC)c1OC)C | 1 |
| Clc1c(c2cc(Cl)c(Cl)cc2)cc(Cl)c(Cl)c1 | 1 |
| Clc1ccc([C@]2(O)CC[N@H+](CC2)CCCC(=O)c2ccc(F)cc2)cc1 | 1 |
| O1[C@@H](n2cc(c(=O)[nH]c2=O)C)C[C@H](N=[N+]=[N-])[C@H]1CO | 1 |
| Clc1c(CC(=O)N=C(N)N)c(Cl)ccc1 | 1 |
| [O-]C(=O)CCCCCC[NH2+]C1c2c(CCc3c1cccc3)cccc2 | 1 |
| S(=O)(=O)([N-]C(=O)NC1CCCCC1)c1ccc(CCNC(=O)c2ncc(nc2)C)cc1 | 1 |
| [S@+](C[C@H]1O[C@@H](n2c3ncnc(N)c3nc2)[C@@H](O)[C@H]1O)(CC[C@H]([NH3+])C(=O)[O-])C | 1 |
| [O-]C(=O)CC1(CCCCC1)C[NH3+] | 1 |
| O=C1Cc2c(N(c3c1cccc3)C(=O)N)cccc2 | 1 |
| Oc1cc(ccc1)C | 1 |
| FC(F)(F)c1ccc(O[C@@H](CC[NH2+]C)c2ccccc2)cc1 | 1 |
| O[C@]1([C@@H](CCCC1)C[NH+](C)C)c1cc(OC)ccc1 | 1 |
| S1[C@H]2N([C@@H](C1(C)C)C(=O)[O-])C(=O)[C@H]2NC(=O)[C@H]([NH3+])c1ccc(O)cc1 | 1 |
| FC(F)(F)COc1c(C(=O)[NH2+]CC2NCCCC2)cc(OCC(F)(F)F)cc1 | 1 |
| Clc1ccc(C(=O)c2ccc(OC(C)(C)C(=O)OC(C)C)cc2)cc1 | 1 |
| S1[C@H]2N(C(=O)[C@H]2NC(=O)Cn2nnnc2)C(=C(C1)CSc1sc(nn1)C)C(=O)[O-] | 1 |
| O(CC(CCn1c2nc(ncc2nc1)N)COC(=O)C)C(=O)C | 1 |
| O1C(C(=O)N2CCN(CC2)c2nc3c(c(n2)N)cc(OC)c(OC)c3)COc2c1cccc2 | 1 |
| Oc1c(C(C)(C)C)cc(cc1C(C)(C)C)C | 1 |
| N(Nc1ccccc1)c1ccccc1 | 1 |
| Brc1[nH]c2c3c(C4=C[C@@H](C(=O)N[C@@]5(O[C@@]6(O)N([C@@H](C(=O)N7[C@@H]6CCC7)CC(C)C)C5=O)C(C)C)C[N@H+]([C@@H]4Cc13)C)ccc2 | 1 |
| O=C(c1ccccc1)c1ccccc1 | 1 |
| O=C(Nc1c(cc(/N=N/c2c(cccc2)C)cc1)C)c1n(ncc1)C | 1 |
| Fc1c(N2C[C@H]3[C@@H]([C@H]3NC(=O)[C@H](NC(=O)[C@H]([NH3+])C)C)C2)nc2n(cc(c(=O)c2c1)C(=O)[O-])c1c(F)cc(F)cc1 | 1 |
| [O-]C(=O)[C@@H]([NH3+])Cc1ccc(O)cc1 | 1 |
| Clc1c(NC(=O)c2sc(Nc3nc(nc(N4CC[NH+](CC4)CCO)c3)C)nc2)c(ccc1)C | 1 |
| O1C(n2c3[nH]cnc(=O)c3nc2)CCC1CO | 1 |
| O1[C@H]([C@H](CCCCCC)C1=O)C[C@@H](OC(=O)[C@@H](NC=O)CC(C)C)CCCCCCCCCCC | 1 |
| [O-][N+](=O)c1c2c(c3c(cc2)cccc3)cc2c1cccc2 | 1 |
| [NH3+][C@@H](Cc1ccccc1)C | 1 |
| ClC(Cl)C(=O)NC(C(O)c1ccc([N+](=O)[O-])cc1)CO | 1 |
| Oc1c(n(ccc1=O)C)C | 1 |
| [O-][N+](=O)c1c(c([N+](=O)[O-])cc(N)c1)C | 1 |
| O(c1ccc(C(O)C#C)cc1)C | 1 |
| O1[C@@H]([C@@H](O)[C@H](O)[C@@H](NC(=O)N(N=O)C)[C@H]1O)CO | 1 |
| ClCCN(P1(=O)OCCCN1)CCCl | 1 |
| Clc1cc2NC(=c3c(=Nc2cc1)cccc3)N1CC[N@H+](CC1)C | 1 |
| Clc1cc2N(CCC[NH+](C)C)c3c(CCc2cc1)cccc3 | 1 |
| Fc1c(N2CC[NH2+]CC2)cc2n(C3CC3)cc(c(=O)c2c1)C(=O)[O-] | 1 |
| O(c1ccc(/C(=C(/CC)\c2ccccc2)/c2ccccc2)cc1)CC[NH+](C)C | 1 |
| ClCCN(c1ccc(CCCC(=O)[O-])cc1)CCCl | 1 |
| Clc1ccc([C@H]([N@@H+]2CC[N@H+](CC2)CCOCC(=O)[O-])c2ccccc2)cc1 | 1 |
| [N@H+]([C@@H](Cc1ccccc1)C)(CC#C)C | 1 |
| O[C@@]12[C@H]([C@H]([NH+](C)C)C(=O)/C(=C(/[O-])\N)/C2=O)C[C@H]2C(=C([O-])c3c(C2)c(N(C)C)ccc3O)C1=O | 1 |
| Nc1c(cc(c2c(cccc2)C)cc1)C | 1 |
| ClCCN(N=O)C(=O)NCCCl | 1 |
| [O-]C(=O)C(NN)(Cc1cc(O)c(O)cc1)C | 1 |
| o1c(c(c2c1cccc2)C(=O)c1ccc(O)cc1)CC | 1 |
| O=C(NCc1cc(OC)c(O)cc1)CCCC/C=C/C(C)C | 1 |
| S1C(C2NC(=O)NC2C1)CCCCC(=O)[O-] | 1 |
| O=c1n(c(=O)n(c2ncn(c12)C)C)C | 1 |
| O=C1NC(=O)NC(=O)C1(CC(C)C)CC=C | 1 |
| ClC/C=C/Cl | 1 |
| O(c1c(C(C)(C)C)cc(O)cc1)C | 1 |
| O1C2(C3(C(C4C(C(O)C3)C3(C(=CC(=O)C=C3)CC4)C)CC2OC1CCC)C)C(=O)CO | 1 |
| Brc1c(NC2=[NH+]CCN2)ccc2nccnc12 | 1 |
| [S-]C1OC(C(OC(=O)C)C(OC(=O)C)C1OC(=O)C)COC(=O)C | 1 |
| [O-][N+](=O)c1c2c(c3c(c4c(cc3)cccc4)c1)cccc2 | 1 |
| O(C[C@@H](O)C[NH2+]C(C)C)c1ccc(COCCOC(C)C)cc1 | 1 |
| O(c1ccc(C(c2ccc(OC(=O)C)cc2)c2ncccc2)cc1)C(=O)C | 1 |
| S(=O)(=O)(CC(O)(C)C(=O)Nc1cc(c(cc1)C#N)C(F)(F)F)c1ccc(F)cc1 | 1 |
| o1c(ccc1C=O)CO | 1 |
| Fc1ccc(CNC(=O)c2nc(n(c(=O)c2[O-])C)C(NC(=O)c2oc(nn2)C)(C)C)cc1 | 1 |
| o1c2c(c3c(cc2)cccc3)c(=O)cc1c1ccccc1 | 1 |
| O([C@H]1C[C@H]2[N@H+](C(CC2)C1)C)C(c1ccccc1)c1ccccc1 | 1 |
| S1(=O)(=O)NC(Nc2c1cc(S(=O)(=O)N)c(c2)C(F)(F)F)Cc1ccccc1 | 1 |
| Clc1ccc([C@@H](CC(=O)[O-])C[NH3+])cc1 | 1 |
| Clc1ccc(Cc2nn([C@H]3CCC[N@@H+](CC3)C)c(=O)c3c2cccc3)cc1 | 1 |
| Fc1ccc(c2n(c(C(C)C)c(c2c2ccccc2)C(=O)Nc2ccccc2)CCC(O)CC(O)CC(=O)[O-])cc1 | 1 |
| O(C(=O)C1=CCC[N@H+](C1)C)C | 1 |
| O=Cc1c2=c3c(=Nc2cc2=c4c(=Nc12)cccc4)cccc3 | 1 |
| Oc1c2c3c4[C@H]([N@H+](CCc4ccc3)C)Cc2ccc1O | 1 |
| s1sc(=S)cc1c1ccc(OC)cc1 | 1 |
| S1[C@H]2N([C@@H](C1(C)C)C(=O)[O-])C(=O)[C@H]2NC(=O)[C@H]([NH3+])c1ccccc1 | 1 |
| [O-][N+](=O)c1c2c3c4c(cc2)cccc4ccc3cc1 | 1 |
| O(CC[NH+]1CCCC1)c1ccc([C@H]2[C@@H](CCc3c2ccc(O)c3)c2ccccc2)cc1 | 1 |
| O=C1N(C2CCC(=O)NC2=O)Cc2c1cccc2N | 1 |
| Clc1c(onc1C)[N-]S(=O)(=O)c1c(scc1)C(=O)Cc1c(cc2OCOc2c1)C | 1 |
| Ic1c(OCC[NH+](CC)CC)c(I)cc(C(=O)c2c(oc3c2cccc3)CCCC)c1 | 1 |
| O([C@H]1[C@H](NC(=O)[C@H](O)CC[NH3+])C[C@@H]([NH3+])[C@@H](O[C@H]2O[C@@H]([C@@H](O)[C@@H](O)[C@H]2O)C[NH3+])[C@H]1O)[C@H]1O[C@@H]([C@@H](O)[C@@H]([NH3+])[C@H]1O)CO | 1 |
| Clc1c(c2noc(c2C(=O)N[C@H]2[C@H]3SC([C@@H](N3C2=O)C(=O)[O-])(C)C)C)c(F)ccc1 | 1 |
| O1c2c(OC1)ccc(C(O)C=C)c2 | 1 |
| O1C(CCCC(CCCC(CCCC(C)C)C)C)(CCc2c1c(c(c(O)c2C)C)C)C | 1 |
| [S@@](=O)(=N)(CC[C@H]([NH3+])C(=O)[O-])CCCC | 1 |
| O=C1N(CCc2n(c3c(c12)cccc3)C)Cc1nc[nH]c1C | 1 |
| Clc1cc(Nc2ncnc3c2cc(c2oc(C[NH2+]CCS(=O)(=O)C)cc2)cc3)ccc1OCc1cc(F)ccc1 | 1 |
| ClCC(=O)N(c1c(CC)cccc1CC)COC | 1 |
| O(C[C@H]1[C@@H](CC[NH2+]C1)c1ccccc1)c1cc2OCOc2cc1 | 1 |
| O1C(C(O)C(O)C(O)C1O)CO | 1 |
| Clc1nc2n([C@@H]3O[C@@H]([C@@H](O)C3)CO)cnc2c(n1)N | 1 |
| O(Cn1c2[nH]c(nc(=O)c2nc1)N)CCO | 1 |
| S(=O)(=O)([N-]C(=O)NC1CCCCC1)c1ccc(cc1)C(=O)C | 1 |
| O(c1cc(C(O)C=C)ccc1OC)C | 1 |
| OCC1CC(n2c3nc(nc(NC4CC4)c3nc2)N)C=C1 | 1 |
| OCCCCN(N=O)CCCC | 1 |
| c12c(c3c(cc2C)cccc3)ccc2c1cccc2 | 1 |
| Brc1c(Oc2c(cc(cc2C)C#N)C)nc(Nc2ccc(cc2)C#N)nc1N | 1 |
| O1C(n2c3ncnc(N)c3nc2)C(O)C(O)C1CO | 1 |
| OC1(C2(C(C3C(C(O)C2)C2(C(=CC(=O)C=C2)C(C3)C)C)CC1)C)C(=O)COC(=O)CCC(=O)[O-] | 1 |
| BrCC(CO)(CO)CBr | 1 |
| O1[C@H]2OC=C[C@H]2c2c3oc(=O)c4c(c3c(OC)cc12)CCC4=O | 1 |
| FC(F)(F)Oc1c(C(=O)C[N+](=O)[O-])cccc1 | 1 |
| O1[C@@H](n2cc(c(=O)[nH]c2=O)C)C=C[C@H]1CO | 1 |
| O=C1NC(=O)NC1(c1ccccc1)c1ccccc1 | 1 |
| O=C(Nc1c(cccc1C)C)[C@H]1[N@@H+](CCCC1)CCC | 1 |
| O([C@H]1C[C@H]2[N@H+](C(CC2)C1)C)C(=O)[C@@H](c1ccccc1)CO | 1 |
| [NH2+]1CC2C[C@@H](c3c2cc2nccnc2c3)C1 | 1 |
| O([C@]1([C@@]2([C@H]([C@H]3[C@@H]([C@@]4(C(=CC(=O)CC4)CC3)C)CC2)CC1)C)C(=O)C)C(=O)CCCCC | 1 |
| [O-]C(=O)[C@@H](NC(=O)c1ccc(NCc2nc3c(nc2)nc(nc3N)N)cc1)CCC(=O)[O-] | 1 |
| c12c3c4ccc1c(ccc2ccc3ccc4)C | 1 |
| O(C1CC(O)(Cc2c1c(O)c1c(c2O)C(=O)c2c(C1=O)c(OC)ccc2)C(=O)CO)C1OC(C(O)C([NH3+])C1)C | 1 |
| Nc1c2c(c(N)ccc2)ccc1 | 1 |
| O=C(N)c1cc(N)ccc1 | 1 |
| S(=O)(=O)([N-]c1cc([C@H](C2=C(O[C@@](CC2=O)(CCc2ccccc2)CCC)[O-])CC)ccc1)c1ncc(cc1)C(F)(F)F | 1 |
| Clc1nc(c(nc1N)N)C(=O)N=C(N)N | 1 |
| O1c2cc(C[C@H]([NH2+]C)C)ccc2OC1 | 1 |
| O1C2C(C(C(O)C2)/C=C/C(O)CCCCC)C/C/1=C/CCCC(=O)[O-] | 1 |
| [NH3+][C@H]1CC[C@H](C[C@H]2CC[C@H]([NH3+])CC2)CC1 | 1 |
| O1CC[NH+](CC1)CCOc1c2c(c(NC(=O)Nc3n(nc(C(C)(C)C)c3)c3ccc(cc3)C)cc1)cccc2 | 1 |
| O(c1cc2c(cc([C@H](C)C(=O)[O-])cc2)cc1)C | 1 |
| Clc1ccc(c2[nH]nc(N3CC[NH+](CC3)C)c2c2ccncc2)cc1 | 1 |
| [NH+](Cc1c2c(ccc1)cccc2)(C/C=C/C#CC(C)(C)C)C | 1 |
| Cl/C(=C(/c1ccc(OCC[NH+](CC)CC)cc1)\c1ccccc1)/c1ccccc1 | 1 |
| S(=O)(=O)(OCc1oc(cc1)C=O)[O-] | 1 |
| n1(c2c(c3c1cncc3)cccc2)c1ccc(N)cc1 | 1 |
| O([C@H]1[C@H]2CC[N@@H+](C1)CC2)C(=O)N1[C@@H](c2c(CC1)cccc2)c1ccccc1 | 1 |
| Nc1cc2Cc3c(c2cc1)cccc3 | 1 |
| Cl[C@H]1[C@@H]2N(C[C@H]1Cl)C(=O)[C@@H](NC(=O)C[C@@H](NC(=O)[C@@H](NC(=O)[C@@H](NC2=O)[C@@H](O)C)CO)c1ccccc1)CC | 1 |
| S(c1ccc(C[C@H]([NH3+])C)cc1)C | 1 |
| Fc1ccc(N2[C@@H]([C@@H](CC[C@H](O)c3ccc(F)cc3)C2=O)c2ccc(O)cc2)cc1 | 1 |
| Fc1c(N2CC[N@H+](CC2)C)c2OC[C@H](n3c2c(c1)c(=O)c(c3)C(=O)[O-])C | 1 |
| O1CCN(N=O)C1=O | 1 |
| O[C@H]([C@@H](NC(=O)[C@@H](NC(=O)OC)C(C)(C)C)Cc1ccccc1)CN(NC(=O)[C@@H](NC(=O)OC)C(C)(C)C)Cc1ccc(cc1)c1ncccc1 | 1 |
| Fc1ccc([C@]2(OCc3c2ccc(c3)C#N)CCC[NH+](C)C)cc1 | 1 |
| O([C@H]1C[C@H]2[N@@H+]3[C@@H](C[C@H](C2)C(=O)C3)C1)C(=O)c1c2c([nH]c1)cccc2 | 1 |
| n1(nnc2c1cccc2)N | 1 |
| N1N=CC=Cc2c1cccc2 | 1 |
| s1c(nc(c1C(=O)[O-])C)c1cc(c(OCC(C)C)cc1)C#N | 1 |
| O[C@@H]1[C@@]2([C@H]([C@H]3[C@H](CC2)[C@@]2(C(=CC(=O)C=C2)CC3)C)CC1)C | 1 |
| O=c1n(c(=O)n(c2nc([nH]c12)C1CCCC1)CCC)CCC | 1 |
| S(c1nc(NC(C)C)nc(NCC)n1)C | 1 |
| O(c1cc2c(cc(C(C)C(=O)[O-])cc2)cc1)C | 1 |
| Clc1c(Cl)ccc(Cl)c1 | 1 |
| Clc1ccc(COc2ccc(COC(=O)c3cccnc3)cc2)cc1 | 1 |
| O(C(=O)C1=C(NC(=C(C1C)C(=O)OCC)C)C)CC | 1 |
| Clc1cc2c(C(=C3CC[NH2+]CC3)c3ncccc3CC2)cc1 | 1 |
| Clc1cc(c2ccc(S(=O)(=O)C)cc2)c(nc1)c1ccc(nc1)C | 1 |
| Cl[C@]12[C@@H]3[C@H]([C@](Cl)(C1(Cl)Cl)C(=C2Cl)Cl)[C@@H]1C[C@H]3C=C1 | 1 |
| Clc1cc([N+](=O)[O-])cc(Cl)c1O | 1 |
| [NH2+]([C@@H](Cc1ccccc1)C)C | 1 |
| SC[C@H](NC(=O)C)C(=O)[O-] | 1 |
| Brc1c(Br)ccc(Br)c1 | 1 |
| S(C[C@H](NC(=O)CC[C@H]([NH3+])C(=O)[O-])C(=O)NCC(=O)[O-])c1c(O)ccc(O)c1 | 1 |
| [O-][N+](=O)c1cc2Cc3c(c2cc1)cccc3 | 1 |
| [O-][N+](=O)c1c(c([N+](=O)[O-])ccc1)C | 1 |
| o1c2c3c(ccc2c(=O)cc1c1ccccc1)cccc3 | 1 |
| [O-][N+](=O)c1c2c3c(CCc3ccc2)cc1 | 1 |
| Clc1c([C@H](Oc2cc(c3cn(nc3)C3CC[NH2+]CC3)cnc2N)C)c(Cl)ccc1F | 1 |
| O=C(N[C@@H](Cc1ccccc1)C)[C@@H]([NH3+])CCCC[NH3+] | 1 |
| Oc1c(Cc2c3c(cc(c2O)C(=O)[O-])cccc3)c2c(cc1C(=O)[O-])cccc2 | 1 |
| O=c1n(c(N2C[C@H]([NH3+])CCC2)cc(=O)n1C)Cc1c(cccc1)C#N | 1 |
| Brc1c(Br)cccc1 | 1 |
| O1C(n2cc(c(=O)[nH]c2=O)C)CC(O)C1CO | 1 |
| OC(CC)C(=O)[O-] | 1 |
| Clc1c2c(c(Cl)c(Cl)c1Cl)ccc(Cl)c2Cl | 1 |
| S1[C@H]2[C@H]3[N@H+]([C@H](O)[C@@H]4[N@@H+]([C@@H]3c3c(C4)cc(c(OC)c3O)C)C)[C@@H](c3c2c(OC(=O)C)c(c2OCOc32)C)COC(=O)[C@@]2([NH2+]CCc3c2cc(OC)c(O)c3)C1 | 1 |
| S(C[C@H]([NH3+])C(=O)[O-])CC(=O)[O-] | 1 |
| o1c2c(c3oc(=O)ccc3cc2)cc1 | 1 |
| O[C@@H]([C@H]1[N@@H+]2C[C@H]([C@H](C1)CC2)C=C)c1c2c(ncc1)ccc(OC)c2 | 1 |
| Oc1c(c(CO)cnc1C)C=O | 1 |
| OCc1c2c3c4c(cc2)cccc4ccc3cc1 | 1 |
| [O-]C(=O)C(CCC)CC=C | 1 |
| S(=O)(=O)(Nc1nc(nc(OCCO)c1Oc1c(OC)cccc1)c1ncccn1)c1ccc(C(C)(C)C)cc1 | 1 |
| Clc1c(S(=O)(=O)Nc2c(Oc3c(OC)cc(cc3)CC(=O)[O-])ccc(c2)C(=O)NCCCC)ccc(Cl)c1 | 1 |
| O=Nc1c2c3c4c(cc2)cccc4ccc3cc1 | 1 |
| S(=O)(CCCCCCCCC[C@H]1[C@H]2[C@H]3[C@](CC[C@@H]2c2c(C1)cc(O)cc2)([C@@H](O)CC3)C)CCCC(F)(F)C(F)(F)F | 1 |
| O=C1N(CCc2c1n(nc2C(=O)N)c1ccc(OC)cc1)c1ccc(N2CCCCC2=O)cc1 | 1 |
| Fc1c(N2C[C@@H]3[C@H]([NH2+]CCC3)C2)c(OC)c2n(C3CC3)cc(c(=O)c2c1)C(=O)[O-] | 1 |
| O=C1N[C@H]2C[C@@H]1[C@H]([C@H]2O)O | 0 |
| OC[C@H]1O[C@@H]([C@H]([C@@H]1O)O)n1c(SC)nc2c1cc(C)c(c2)C | 0 |
| OCc1nc2cccc3c2c([nH]1)ccc3 | 0 |
| CCc1c(C)nc[nH]c1=O | 0 |
| Cc1c(cc2n1c1ccccc1n2C)c1ccccc1 | 0 |
| Clc1ccc(cc1)C1c2c(cc(oc2=O)C)Oc2c1c(=O)oc(c2)C | 0 |
| Clc1ccc2n(c1)c(NC(=O)c1ccccc1O)c(n2)c1ccc(cc1)C | 0 |
| O=C1N[C@H]2[C@@H]3CCCC[C@H]3/C(=C\c3ccccc3)/N2[C@H]2[C@H]1[C@H]1CCC[C@H]1S2 | 0 |
| COc1ccc(c(c1I)F)F | 0 |
| CCOc1ccc(cc1C)C | 0 |
| C[C@@H]1C[C@H](C)CN(C1)c1cc(C)c2c(n1)cc(cc2)Cl | 0 |
| Fc1cccc(c1)NCc1ccccc1O | 0 |
| Fc1ccc(cc1)C1=C(C(=O)Nc2ccccc2)[C@@H](C)Nc2n1c1ccccc1n2 | 0 |
| N#Cc1c(SCc2nc3c([nH]2)cccc3)nc(cc1C(F)(F)F)c1cccs1 | 0 |
| Brc1ccc(c(c1)C(=O)NCc1ccc2c(c1)OCO2)O | 0 |
| O=c1[nH]c([nH+]c(c1Cc1ccccc1)[O-])N1CCc2c1cccc2 | 0 |
| Oc1ccc2c(c1)nc(o2)c1ccncc1 | 0 |
| N#Cc1cc(c2cccs2)c(=O)[nH]c1SCC(=O)c1ccc(cc1)Br | 0 |
| Fc1ccc(cc1)c1cccn1C | 0 |
| O=C1NC(=C([C@H](N1)c1ccccc1OC1CCCC1)C(=O)Nc1cccc(c1)Cl)C | 0 |
| Ic1ccc(cc1)C(=O)C[C@H]1Sc2c(S1)ccc(c2)C | 0 |
| O[C@@H]1c2ccccc2N(c2c([C@@H]1O)cccc2)C(=O)N | 0 |
| CC(C#Cc1ccc(s1)Br)(O)C | 0 |
| Cc1ccc(cc1)c1nc(O)c(c(=O)o1)C | 0 |
| Cc1ccc(cc1)S(=O)(=O)NC(=O)n1cnc2c1[nH]c(=O)[nH]c2=O | 0 |
| Brc1ccc(cc1)n1c(/C=C\c2c[nH]c3c2cccc3)nc2c(c1=O)cccc2 | 0 |
| Oc1ccccc1C(c1c(O)cc(n(c1=O)C)C)c1c(O)cc(n(c1=O)C)C | 0 |
| CC(/C=C/1\[C@H](C)NN(C1=C)c1ccccc1)C | 0 |
| CO[C@@H]1C[C@@H]2CCC(C1)[C@@H]2C(=O)N1c2ccccc2C=Cc2c1cccc2 | 0 |
| Nc1cc(Br)c(cc1N)F | 0 |
| COC(=O)c1cc(F)c(c(c1F)F)F | 0 |
| Fc1ccc(cc1C)OC(F)(F)F | 0 |
| CNc1cccc(c1)C(F)(F)F | 0 |
| [O-]C(=O)C1=[C-]N2C(=[NH+]1)C=C[NH2+]2 | 0 |
| Cc1c(C)cccc1N1CCCC1 | 0 |
| Brc1ccc(c(c1)Cl)S(=O)(=O)Oc1ccccc1Cl | 0 |
| N#Cc1cccc(c1)C[NH2+][C@@H]1CCN(C[C@@H]1C(=O)[O-])C(=O)OC(C)(C)C | 0 |
| COC1=C(OC)C(C1(F)F)(F)F | 0 |
| O=C(c1cc(=O)[nH]c2c1cccc2)Nc1cc(ccc1N1CCOCC1)C(F)(F)F | 0 |
| O=C(Nc1ccc(cc1)NC(=O)C)CSc1nc(N)cc(=O)[nH]1 | 0 |
| N#C/C(=c/1\s/c(=C\c2ccc(cc2)OC(F)F)/c(=O)n1c1ccc(cc1)C(C)C)/C(=O)C(C)(C)C | 0 |
| OCCCc1cccnc1 | 0 |
| Fc1ccc(cc1)Nc1cc(N)nc(n1)N | 0 |
| OCCn1ncc2c1[nH]c(SCC(=O)Nc1ccc3c(c1)OCO3)nc2=O | 0 |
| Fc1ccccc1CSc1[nH]c(=O)c2c(n1)nc(cc2C(F)(F)F)c1cccs1 | 0 |
| COc1ccc(cc1)n1ccn(c1=O)CC(=O)Nc1ccccc1OC | 0 |
| COc1ccccc1[C@H](c1oc2c(c1NC(=O)c1ccccc1)cccc2)N1CCOCC1 | 0 |
| Fc1cc(F)cc(c1)S(=O)(=O)c1cc2cc(Br)ccc2oc1=O | 0 |
| CC[C@H]1O[C@@H]([C@@H](C1)C)C | 0 |
| Clc1ccc(cc1)[C@@H]1CCC[C@@H]2N1CCn1c2ccc1 | 0 |
| Cc1ccc(c(c1)Cl)NC(=O)N1CCN(CC1)Cc1nc(oc1C)c1ccccc1 | 0 |
| c1ccc(cc1)C=N1CCCCC1 | 0 |
| C1CC[C@H](OC1)C#Cc1ccccc1 | 0 |
| C[C@@H]1C2N[C@]3([C@]([C@H]1C(=C3)c1ccccc1)(O2)c1ccccc1)c1ccccc1 | 0 |
| CCC(/N=C\1/[C@H](O)C(=O)C1=O)(C)C | 0 |
| OC1=C([O-])/C(=[NH+]/C2CC2)/C1=O | 0 |
| O=C1[C@@H](O)/C(=N\Cc2ccccc2Cl)/C1=O | 0 |
| Clc1ccnc2c1ccc1c2c(=O)cc[nH]1 | 0 |
| CCOC(=O)N1CCN(CC1)c1ccc(cc1F)C(=O)c1ccc(c(c1)Br)OC | 0 |
| CC(NC(=O)C[C@@H]1CC[NH2+]C[C@@H]1Cc1noc(c1)C(=O)[O-])C | 0 |
| O=c1nc2ccccc2c([nH]1)c1ccccc1 | 0 |
| FC(c1ccccc1NCc1cccc2c1cc[nH]2)(F)F | 0 |
| O=C([C@H](c1ccccc1)Sc1nc2sc3c(c2c(=O)[nH]1)CCCC3)c1ccccc1 | 0 |
| CC([C@]1(O)CC[C@@]2([C@@H](C1)[C@](C)(O)CC[C@@H]2O)C)C | 0 |
| OC[C@@H]1OC=C[C@@H]([C@H]1O)O | 0 |
| OC[C@@]1(O)C(=C[C@@H]([C@H]2[C@@]1(C)CCCC2(C)C)O)C | 0 |
| O=C(Nc1ccccc1Cl)CNc1cc(Cl)cc(c1)Cl | 0 |
| CN(Cc1ccncc1)C[C@@H]1CCC[N@@H+](C1)CCc1cccc(c1)C(F)(F)F | 0 |
| Brc1ccc2c(c1)CCCCO2 | 0 |
| Brc1cccc(c1)N1CC=CC1 | 0 |
| Brc1cccc(c1)[C@]1(C)CCO1 | 0 |
| Brc1ccc2c(c1)c(c[nH]2)Cc1ccc(cc1)C(F)(F)F | 0 |
| COc1ccccc1CC(=O)N1CC[C@@H]2[C@@H](C1)CCCN2C(=O)COc1ccc(cc1C)Cl | 0 |
| Nc1ccc2c(c1)[nH]c1c2CCCC1=O | 0 |
| COc1cccc(c1)C[C@H](N(C(=O)c1ccco1)C)[C@@H]1CCN(CC1)C(=O)[C@H]1C[C@@H]2C[C@H]1C=C2 | 0 |
| Cc1ccc(cc1)Nc1cc(I)c2c3c1C(=O)c1ccccc1c3on2 | 0 |
| Cn1nc(c(c1)C(=O)Nc1ccc2c(c1)CCC2)c1ccc2c(c1)OCCCO2 | 0 |
| COc1cccc(c1)C[C@H](N(C(=O)c1cccnc1)C)C1CCN(CC1)C(=O)c1ccccc1F | 0 |
| COc1ccc(cc1)CNC(=O)c1nn(c2c1CN(CC2)C(=O)c1ccoc1C)Cc1ccccn1 | 0 |
| C=CCn1cccc1 | 0 |
| CCCn1cccc1 | 0 |
| FC(Oc1ccccc1C)F | 0 |
| COc1cccc2c1nc(Nc1nc(CSc3nnnn3c3ccccc3)cc(=O)[nH]1)nc2C | 0 |
| Clc1cccc(c1)C(=O)Oc1ccc(c2c1cccc2)Br | 0 |
| CC(Oc1ccc2c(c1)[C@@H]1C=CC[C@@H]1[C@@H](N2)c1ccc(cc1)F)C | 0 |
| COc1ccc2c(c1[C@H]1Nc3ccccc3[C@@H]3[C@H]1CC=C3)cccc2 | 0 |
| [O-]C(=O)[C@@H]1CCC(=[NH+]1)N | 0 |
| O[C@H]1CCC[NH2+][C@@H]1C(=O)[O-] | 0 |
| CCOc1ccc2c(c1)[C@@H]1C=CC[C@@H]1[C@@H](N2)c1ccc(cc1Cl)Cl | 0 |
| CCCC(=O)Nc1ccc2c(c1)[C@@H]1C=CC[C@@H]1[C@@H](N2)c1ccc(cc1)C | 0 |
| CC[C@H](NC(=O)c1ccc2c(c1)[C@@H]1C=CC[C@@H]1[C@@H](N2)c1ccc(cc1)C)C | 0 |
| CCCCNC(=O)c1ccc2c(c1)[C@@H]1C=CC[C@@H]1[C@@H](N2)c1ccc(cc1)C | 0 |
| CC(CC(=O)Nc1ccc2c(c1)[C@H]1C=CC[C@H]1[C@H](N2)c1ccc(cc1)C)C | 0 |
| CCN(c1ccc(cc1)NC(=O)c1cn(nc1c1cccnc1)c1ccccc1)CC | 0 |
| CC(=O)c1cc2c(I)c[nH]c2nc1 | 0 |
| O=C(C1CCN(CC1)C(=O)C1CCN(CC1)S(=O)(=O)C)Nc1ccc(cc1)S(=O)(=O)N | 0 |
| CSc1nc2n(n1)c(C)c(c(n2)C)CCC(=O)Nc1ccc2c(c1)[nH]c(=O)[nH]2 | 0 |
| CCCn1c(n[nH]c1=O)S[C@H](C(=O)Nc1cccc(c1)S(=O)(=O)N1CCOCC1)C | 0 |
| O=C1CNc2c1c(=O)[nH]c(=O)n2C | 0 |
| O=C([C@H](Sc1nnnn1c1cccc(c1)F)C)Nc1ccc2c(c1)[nH]c(=O)[nH]2 | 0 |
| Ic1ccc2c(c1)[C@@H]1C=CC[C@@H]1[C@@H](N2)c1ccc2c3c1cccc3CC2 | 0 |
| O=C([C@H](Sc1nnnn1C1CCCC1)C)Nc1ccc2c(c1)[nH]c(=O)[nH]2 | 0 |
| O=C(N1c2ccccc2NC(=O)C1(C)C)[C@@H](Sc1n[nH]c(n1)N)C | 0 |
| CN(C(=O)c1cc(nn1c1ccccc1)C1CC1)Cc1ccc(cc1)N1CCCCC1 | 0 |
| COc1ccc(cc1)CCNC(=O)C[C@@H]1CSc2n1c(=O)c1c(n2)n(nc1)c1ccc(cc1C)C | 0 |
| O=C(c1ccc(c(c1)I)C)OCCc1ccccc1 | 0 |
| c1csc(c1)c1[nH]c2c(c1)cccn2 | 0 |
| Brc1ccc(s1)C1=C(C(=O)OC1)c1ccc(c(c1)Cl)Cl | 0 |
| O=C(Cc1cccc2c1cccc2)Oc1ccc(cc1Br)Cl | 0 |
| Clc1ccc(cc1)CNC1=[NH+][C@@H](n2c(=N1)nc(C)cc2[O-])c1ccc(cc1)Cl | 0 |
| Cc1ccc(cc1)c1[nH]c2c(c1[C@@H]1N(C[C@@H]3CCCO3)C(=O)c3c1cccc3)cccc2 | 0 |
| Nc1ccc(cc1)c1nc2c([nH]1)cccc2C(=O)N | 0 |
| O=C1OC(C)(C)OC(=O)C21Cc1ccccc1N1[C@@H]2C[C@H](CC1)c1ccccc1 | 0 |
| N#Cc1c2CCCc2c(n2c1nc1c2cccc1)N1C[C@H](C)O[C@@H](C1)C | 0 |
| COc1ccc(cc1)[C@@]1(O)OC(=O)C(=C1Cc1cc(OC)c(c(c1)OC)OC)c1ccc2c(c1)OCO2 | 0 |
| CCCC[C@@H]1CC[C@H](CC1)C(=O)N1CCN(CC1)c1nccn1c1ccc(cc1)C | 0 |
| O=C(C[C@@H]1C(=O)N=c2n1c1ccccc1[nH]2)Nc1ccc(c(c1)C(F)(F)F)Cl | 0 |
| CCn1c2ccc(cc2c2c1cccc2)[C@H]1Nc2ccc(cc2[C@@H]2[C@H]1CC=C2)I | 0 |
| CCn1c2ccc(cc2c2c1cccc2)[C@H]1Nc2ccccc2[C@@H]2[C@H]1CC=C2 | 0 |
| COc1c(OC)cc(cc1OC)[C@H]1Nc2c(cccc2[C@@H]2[C@H]1CC=C2)OC[C@@H]1CCCO1 | 0 |
| COc1ccc(cc1)[C@H]1Nc2c(C)ccc(c2[C@@H]2[C@@H]1CC=C2)Cl | 0 |
| COc1ccc(cc1NC(=O)[C@H]1C[C@@H](N[C@]21C(=O)Nc1c2cc(F)cc1)C(C)C)Cl | 0 |
| CC[N@H+]1CCc2c([C@@H]1c1c([O-])n(C)c(=O)n(c1=O)c1ccc(cc1)Cl)[nH]c1c2cccc1 | 0 |
| O[C@H]1[C@H](O)C=C[C@H]([C@@H]1O)O | 0 |
| OC[C@H]1O[C@@H]([C@@H]([C@H]1O)O)Oc1ccc2c(c1)oc(=O)cc2C | 0 |
| OC[C@H]1O[C@H](C[C@H]1O)O | 0 |
| O=C1NC(=O)N=C2[C@@H]1C=NN2 | 0 |
| COc1ccc(cc1NC(=O)CSc1n[nH]c(n1)C(C)C)S(=O)(=O)N1CCOCC1 | 0 |
| O=c1[nH]c(=O)[nH]c2c1CC=N2 | 0 |
| O=C1NCC[C@H](c2c1[nH]c(c2Br)Br)c1c[nH]c(n1)N | 0 |
| CC1(C)CCc2c(O1)c1ccccc1cc2 | 0 |
| CC(C[C@@H]1NC(=O)[C@]23[C@H]1[C@@H](C)C(=C[C@@H]2/C=C(/C)\CC[C@@H]([C@@H]([C@@H](CC3=O)O)O)O)C)C | 0 |
| COc1cc(OC)c(cc1NC(=O)c1ccc2c(n1)c(O)ccc2)Cl | 0 |
| COC(=O)[C@H]1[C@@H](O)C=Cc2c1c(=O)c1c(o2)cc(cc1O)CO | 0 |
| O=C(Nc1c(=O)oc2c(c1NC1CCCCC1)cccc2)Nc1cc(Cl)ccc1C | 0 |
| COc1ccc(cc1)[C@@H]1CC(=O)c2c(C1)[nH]c(=O)c(c2)C(=O)Nc1c(C)cccc1C | 0 |
| COc1ccc(cc1)[C@@H]1CC(=O)c2c(C1)[nH]c(=O)c(c2)C(=O)Nc1cccc(c1)Cl | 0 |
| C[NH+](Cc1ccccc1CNS(=O)(=O)c1scc(c1)C(=O)[O-])C | 0 |
| Cc1ccccc1[C@H]1[C@@H]2[C@@](C2(Cl)Cl)(O[C@]2([C@@H]1C2(Cl)Cl)c1ccccc1)c1ccccc1 | 0 |
| [O-]C(=O)c1c[nH+]c([nH]1)C | 0 |
| O=C(c1cccc(c1Cl)Cl)Nc1ccc(c(n1)C)I | 0 |
| Oc1ccc(cc1)c1nc2c(N)nc(nc2nc1N)N | 0 |
| [O-]C(=O)c1c(C)[nH]c[nH+]1 | 0 |
| Cc1cnc(c2c1c(N)ccc2)C1OCCO1 | 0 |
| Cc1ccc(cc1)c1nn(cc1C(=O)Nc1ccc(cc1)N1CCOCC1)c1ccccc1 | 0 |
| CCCN(c1c(=O)[nH]c(=O)n(c1N)Cc1ccccc1)C(=O)c1sc2c(c1C)c(=O)n1c(n2)CCC1 | 0 |
| O=C(N1CCN(CC1)Cc1ccccc1)CCc1nc2ccccc2n(c1=O)CCc1ccccc1 | 0 |
| O=C1C=C/C(=C\2/NCC[C@@H](N2)c2ccc(cc2)Cl)/C=C1 | 0 |
| O=C(Nc1cc(nn1c1ccccc1)C)CSc1nc(N)cc(=O)[nH]1 | 0 |
| O=C(N(c1c(=O)[nH]c(=O)n(c1N)Cc1ccccc1)C)c1cccc(c1)S(=O)(=O)N1CCOCC1 | 0 |
| N#Cc1c2NC(=C([C@@H](n2nc1N1CCOCC1)c1ccccc1)C(=O)Nc1ccc(cc1)Cl)C | 0 |
| Brc1ccc(cc1)OCCN(C(=O)c1cc(nn1c1ccccc1)C1CC1)C | 0 |
| O=C(C1CCN(CC1)c1ccc(cn1)C(F)(F)F)OCC(=O)N1C[C@]2(CC1CC(C2)(C)C)C | 0 |
| O=C(N(c1c(=O)[nH]c(=O)n(c1N)Cc1ccccc1)Cc1ccco1)c1ccc(=O)n(n1)C | 0 |
| CCCc1ccc(cc1)S(=O)(=O)N1CCN(CC1)CC(=O)N1CCC(CC1)Cc1ccccc1 | 0 |
| CN(c1ccccc1)C(=O)CN1CCN(CC1)S(=O)(=O)c1cc(ccc1Cl)C(F)(F)F | 0 |
| CCCCn1c(=O)[nH]c(=O)c(c1N)N(C(=O)c1cccc(c1)S(=O)(=O)N1CCN(CC1)C)CC | 0 |
| Cc1ccc(cc1)[C@H](c1ccccc1)N[C@H](c1ccccc1)C(=O)N | 0 |
| O=C(c1ccc(c(c1)Cl)O)Nc1cnc2c(c1)c(C)nn2C | 0 |
| Clc1ccc(cc1)OCCN(C(=O)c1nn(c(cc1=O)C)c1cccc(c1)C(F)(F)F)C | 0 |
| O=C(Cn1nc(oc1=O)c1cccs1)Nc1ccc2c(c1)[nH]c(=O)[nH]2 | 0 |
| Brc1ccc(c(c1)S(=O)(=O)NCC#Cc1ccccc1)Br | 0 |
| CC[C@@H]([C@@H](C(=O)N1CC[C@@H](CC1)C(=O)N[C@@H](C(=O)[O-])C(C)C)[NH3+])C | 0 |
| O=C(c1ccc(o1)c1cc(Cl)ccc1Cl)N[C@@]12C[C@H]3C[C@@H](C1)C[C@@H](C2)C3 | 0 |
| Fc1ccc(cc1)OCCCC(=O)N1CCN(CC1)S(=O)(=O)c1ccc(cc1)Br | 0 |
| N#Cc1c(cn2c1sc1ccccc1c2=O)c1ccc(cc1)Br | 0 |
| COc1ccc(cc1)C(=O)[C@@H]1CCN(CC1)C(=O)[C@H]1CC(=O)N(C1)c1cccc(c1)C(F)(F)F | 0 |
| Cc1ccc(cc1)n1ncc2c1CC(C)(C)C[C@@H]2NC(=O)c1cn(C)c2c(c1=O)cccc2 | 0 |
| COc1ccc(cc1)c1nc2ccccc2c(c1)C(=O)Nc1ccccc1N1CCOCC1 | 0 |
| COc1cc(OC)cc(c1)C(=O)Nn1c(=O)cc2c(c1CC)C(=O)N([NH2+]2)c1nc2c(s1)cccc2 | 0 |
| COc1cc(cc(c1OC)OC)C(=O)N(Cc1cc2cc3OCCOc3cc2[nH]c1=O)Cc1cccnc1 | 0 |
| C#CCn1/c(=N\C(=O)CCCS(=O)(=O)c2ccc(cc2)F)/sc2c1ccc(c2)Br | 0 |
| O=C1NC(=C([C@H](N1)c1ccccc1OC(F)F)C(=O)Nc1ccc(cc1)Br)C | 0 |
| CC(Oc1cccc(c1)C(=O)[C@H]1CCCN(C1)Cc1c(C)nn(c1C)C(C)C)C | 0 |
| NC(=O)[C@@H](Cc1c[nH]c2c1cccc2)NC(=O)[C@H]1CNCCN1S(=O)(=O)C | 0 |
| N#C[C@H](c1nc2ccccc2nc1N1C[C@H](C)O[C@@H](C1)C)C(=O)NCCc1ccc(c(c1)OC)OC | 0 |
| CN(C(=O)c1c(C)nn(c1C)Cc1ccccc1)Cc1ccccc1N1CCCCC1 | 0 |
| CCOc1ccc(cc1)[C@@H]1CCCN1C(=O)c1cc(nn1c1ccccc1)C1CC1 | 0 |
| O=c1[nH]c2nc(cc(c2c2c1cccc2)c1ccccc1)C(F)(F)F | 0 |
| C1CCC(C1)[N@H+]1CCO[C@H](C1)CCc1ccccc1 | 0 |
| COCCCn1c(SCC(=O)Nc2oc(c3c2c(=O)[nH]nc3C)C)nc2c(c1=O)cccc2 | 0 |
| O=C(c1ccc2c(c1)C(=O)N=NC2=O)Nc1ccncc1 | 0 |
| NC1=Nc2nc3c(n2[C@@H](N1)c1ccc(cc1)OCc1c(F)cccc1Cl)cc1c(c3)OCCCO1 | 0 |
| O=C(c1ccccc1Nc1cccc(c1C)C)NCc1cccnc1 | 0 |
| O=C(Cn1c(COc2ccccc2)nc2c1cccc2)Nc1ccc(cc1)N1CCCCC1 | 0 |
| COC(=O)c1cccc2c1cccn2 | 0 |
| N#C/C(=c/1\s/c(=C/c2ccc(cc2)N(c2ccccc2)C)/c(=O)n1Cc1ccco1)/C(=O)C(C)(C)C | 0 |
| COc1ccc(c2c1N[C@H](c1ccccc1F)[C@@H]1[C@H]2C=CC1)Cl | 0 |
| Fc1ccc(cc1)C1=CCO1 | 0 |
| COc1cc(OC)cc(c1)C(=O)NCCNC(=O)c1nonc1N | 0 |
| C[C@@H]1NC(=O)N([C@@](C1)(C)O)c1ccccc1 | 0 |
| C1=C[C@H]2O[C@@H]1c1c2cccc1 | 0 |
| Fc1ccccc1c1c[nH]nc1N | 0 |
| Fc1ccc(cc1)c1cnc([nH]1)N | 0 |
| Ic1ccc(cc1)C(=O)Cn1cnc(=O)c(c1N)N | 0 |
| O=C1C[C@@H](c2ccccc2C(F)(F)F)c2c(N1)n(nc2)Cc1ccc2c(c1)OCO2 | 0 |
| Fc1ccc(cc1)Cn1c(=O)c2[C@@H](CC(=O)Nc2n2c1nc1c2cccc1)c1ccccc1F | 0 |
| Clc1cc2[nH]c(=O)oc2c(c1C)Cl | 0 |
| Brc1ccc2c(c1)CCN2C | 0 |
| N#Cc1ncccc1N1CCCCC1 | 0 |
| OCc1ncccc1F | 0 |
| Cc1cc(no1)C#N | 0 |
| CC#CCNc1ccccc1 | 0 |
| N#CCn1cncc1 | 0 |
| N#Cc1ccnnc1 | 0 |
| OCCN1CC2=C(C1=O)[C@H](NC(=O)N2)c1cccs1 | 0 |
| OCc1ccc(nc1)C | 0 |
| Brc1ccc(c(c1)C(=O)N=c1[nH]c2c([nH]1)cccc2)n1cnnn1 | 0 |
| Clc1ccc(c(c1)Cl)[C@@H](/C(=C/c1c(C)nn(c1C)C)/n1ncnc1)O | 0 |
| COc1ccc2c(c1)c1CN(CCc1[nH]2)C(=O)c1[nH]c2c(c1)c(OC)ccc2OC | 0 |
| CC(Cn1cc(C(=O)N2CCC(CC2)c2ccccc2)c(=O)c(c1)C(=O)N1CCCCCC1)C | 0 |
| COc1ccc(cc1CC(=O)N1CC[C@H]2[C@@H](C1)CCC(=O)N2Cc1ccc(cc1)C(F)(F)F)OC | 0 |
| O=C([C@@H]1C[C@H]2CC[C@@H]1C[C@@]12NC(=O)c2c(N1)cccc2)NCCc1c[nH]c2c1cccc2 | 0 |
| O=C(c1[nH]nc2c1CCCC2)N1CCc2c(C1)nc([nH]c2=O)N1CCCCC1 | 0 |
| Clc1cc2C[C@H](Oc2c(c1)c1ccc2c(c1)cc[nH]2)CNC(=O)c1cccc(c1)N1CCNC1=O | 0 |
| O=C(N1CCc2c([C@@H]1c1cccc(c1)C(F)(F)F)[nH]c1c2cccc1)c1cccnc1 | 0 |
| NC(=O)Cn1c(=O)cc(c2c1cccc2)C | 0 |
| O=C(C1CC1)Nc1ccnn1C1CCN(CC1)C(=O)c1ccccc1N(C)C | 0 |
| O=C(C1CCN(CC1)C(=O)CSc1nccn1c1cc(C)cc(c1)C)Nc1ccc2c(c1)OCO2 | 0 |
| Fc1cc2C[C@H](Oc2c(c1)c1nc(C)cnc1C)CNC(=O)c1ccc2c(c1)[nH]cc2 | 0 |
| Fc1ccc(c(c1)F)C[C@H](N(C(=O)C1CC1)C)C1CCN(CC1)C(=O)c1ccc(cc1)n1cccn1 | 0 |
| Cc1cccc(c1)n1nnc(c1N)C(=O)Nc1c(C)cccc1C | 0 |
| O=C(c1nnn(c1N)c1ccccc1)Nc1ccc(c(c1)C)Cl | 0 |
| CCc1cccc(c1NC(=O)c1nnn(c1N)c1ccccc1)C | 0 |
| Brc1ccc2c(c1)c(c1ccccc1)c(c(=O)[nH]2)C(=O)C | 0 |
| O=c1c2c(c(oc2n(c(=O)n1C)C)NCc1ccccc1)c1cccc2c1cccc2 | 0 |
| O=C(Nc1cccc2c1cccc2)NCc1cccc(c1)C(F)(F)F | 0 |
| COc1cc(ccc1OC(C)C)C(=O)N(Cc1ccc(cc1)N1CCCCC1)C | 0 |
| O=C(CSc1n[nH]c(n1)N)NCC(=O)Nc1c(C)cc(cc1C)C | 0 |
| O=C1Nc2ccccc2[C@@H](C1)C(=O)Nc1ccc(cc1)N1CCCCC1 | 0 |
| O=C(Oc1cccc(c1)C(=O)OC[C@@H]1CCCO1)COc1ccc(cc1Cl)Br | 0 |
| O=C(Nc1cc(Cl)ccc1N1CCOCC1)CSc1nc(N)cc(=O)[nH]1 | 0 |
| CCCCC[NH2+]Cc1cn(c2c1cccc2)Cc1ccc(cc1Cl)Cl | 0 |
| Clc1ccc(cc1)c1[nH]c(=O)nc2c1cc(Br)cc2 | 0 |
| COc1c(OC)cc(cc1OC)NC(=O)CCc1c(C)nn(c1C)C | 0 |
| CSc1cccc2c1nc(s2)Nc1nc2c(s1)cc(cc2)Br | 0 |
| O=C(C1CCN(CC1)C(=O)Cn1c(=O)oc2c1cccc2)Nc1ccc(cc1)S(=O)(=O)N | 0 |
| CC(=O)N[C@@]1(C)[C@](C)(O)O[C@@]2([C@@]([C@@]1(C)O)(C)O[C@H](OC2)c1ccccc1)C | 0 |
| OC[C@@]1(C)O[C@@]([C@@H]([C@@H]1O)O)(C)Oc1ccc2c(c1)oc(=O)cc2C | 0 |
| CC(=O)Nc1cccc(c1)C1=C[C@@H](n2c(N1)nc(n2)C(F)(F)F)c1ccc2c(c1)OCCO2 | 0 |
| O=C(N1c2ccccc2Sc2c1cccc2)CSc1nnc([nH]1)c1ccccc1F | 0 |
| O=C(c1ccc(cc1)N1C(=O)NC2C[C@]1(C)Oc1c2cccc1)NCCc1c[nH]c2c1cccc2 | 0 |
| O=C(CCc1nnc2n1c1ccccc1c(=O)n2Cc1ccc(cc1)C)NCc1ccc2c(c1)OCO2 | 0 |
| COc1cc(ccc1OC)NC(=O)CCc1nnc2n1c1ccccc1c(=O)n2Cc1ccccc1 | 0 |
| OCc1cnc(n1CC(=O)N)SCC(=O)NCc1ccc2c(c1)OCO2 | 0 |
| O=C(c1c(=O)[nH]c(=O)n(c1N)C)CSc1nnc(n1C1CC1)COc1ccccc1Cl | 0 |
| O=C(Nc1ccc2c(c1)OCCCO2)CSc1nnc(n1C[C@@H]1CCCO1)c1ccc(cc1)F | 0 |
| O=C(N1CCN(CC1)S(=O)(=O)c1ccc2c(c1)cccc2)CNC(=O)c1ccc2c(c1)OCO2 | 0 |
| O=C(C1CCN(CC1)S(=O)(=O)c1ccc2c(c1)OCCO2)NCCc1ccc2c(c1)OCCO2 | 0 |
| O=C(Cn1cc(ccc1=O)S(=O)(=O)N(C)C)Nc1ccc2c(c1)[nH]c(=O)[nH]2 | 0 |
| COc1ccc(cc1S(=O)(=O)N1CCOCC1)NC(=O)[CH-]N1[NH2+]C(=O)c2c(C1=O)cccc2 | 0 |
| Fc1ccc(cc1)c1nnc(n1C[C@@H]1CCCO1)SCC(=O)c1c(=O)[nH]c(=O)n(c1N)C1CC1 | 0 |
| COc1ccc(cc1C(=O)Nc1cc(OC)c(c(c1)OC)OC)S(=O)(=O)N1CCc2c1cccc2 | 0 |
| COc1ccc(c(c1)OC)n1c(=O)n(CC(=O)Nc2n[nH]c(c2)C)c2c(c1=O)c1CCCCc1s2 | 0 |
| O=C(NCc1ccc2c(c1)OCO2)COc1ccc(cc1C(=O)N)S(=O)(=O)N1CCCC1 | 0 |
| COc1ccc(cc1)n1c(SCC(=O)Nc2ccc(cc2OC)OC)nc2c(c1=O)cn[nH]2 | 0 |
| COc1ccc(cc1)n1c(SCC(=O)Nc2ccc3c(c2)OCCO3)nc2c(c1=O)cn[nH]2 | 0 |
| OC[C@H]([C@H]([C@H](c1oc(c(c1)C(=O)OCc1ccccc1C#N)C)O)O)O | 0 |
| Brc1ccc(cc1)OCCN(C(=O)c1cccc(c1)S(=O)(=O)N1CCCC1)C | 0 |
| O=C1NC(=C([C@H](N1)c1c(C)nn(c1Cl)c1ccccc1)C(=O)Nc1ccc(cc1)F)C | 0 |
| O=C(CN(S(=O)(=O)c1ccc2c(c1)NC(=O)CS2)C)NCc1ccc2c(c1)OCO2 | 0 |
| CC[C@@H]1CCCCN1C(=O)c1cnn2c1N[C@@H](C[C@@H]2C(F)(F)F)c1ccc(cc1)OC | 0 |
| O=C1N(C[C@@H]2CCCO2)[C@@H](Nc2c1cccc2)c1cc(Cl)c2c(c1)OCO2 | 0 |
| C#CCSc1nc2NC(=O)C[C@H](c2c(=O)[nH]1)c1cc(OC)c(c(c1)OC)O | 0 |
| Clc1ccc(cc1)[C@@H]1c2c(C(=O)N1c1ccccc1)n[nH]c2c1ccccc1 | 0 |
| Clc1ccc2c(c1)C1NC(=O)N([C@@](O2)(C1)C)c1cccc(c1)C(=O)Nc1ccc2c(c1)OCCO2 | 0 |
| N#Cc1c(=O)[nH]c(cc1c1ccc(c(c1)Cl)Cl)c1ccccc1 | 0 |
| COc1ccc(cc1)Cn1c(SCC(=O)Nc2ccc(cc2F)F)nc2c(c1=O)nn(c2)CC | 0 |
| Clc1ccc2c(c1)N(C(=O)CSc1nc(C)cc(=O)[nH]1)c1c(S2)cccc1 | 0 |
| CCOc1cccc2c1Oc1nc([nH]c(=O)c1C2)c1cc(Br)ccc1O | 0 |
| COc1cc(ccc1O)[C@@H]1C(=C(C)Nc2n1c1ccccc1n2)C(=O)Nc1ccc(cc1)C | 0 |
| O=C(c1ccccc1)N[C@@H]1C(=O)Nc2c([C@H]1c1cc(Cl)c3c(c1)OCCCO3)c(C)nn2C | 0 |
| O=C(c1ccccc1C(F)(F)F)Nc1ccc2c(c1)C(=O)NCCO2 | 0 |
| O=C1Nc2cc(ccc2N2[C@@H]1CCC2)C(=O)Nc1ccc(cc1)Cl | 0 |
| O=C(Cn1c2ccccc2nc1C(F)(F)F)OCC(=O)N1CCC(CC1)Cc1ccccc1 | 0 |
| O=C(CNC(=O)c1cccs1)NCC(=O)Nc1nc2c([nH]1)cccc2 | 0 |
| COc1ccc(cc1C(=O)Nc1ccc2c(c1)C(=O)N(C)c1c(O2)ccc(c1)C)Cl | 0 |
| Cc1ccc2c(c1)NC(=O)c1c(O2)ccc(c1)NC(=O)c1c(F)cccc1F | 0 |
| Cc1ccc2c(c1)NC(=O)c1c(O2)ccc(c1)NC(=O)c1ccccc1C | 0 |
| OC1CCN(CC1)C(=O)Cc1c(=O)oc2c(c1C)c(O)cc(c2)O | 0 |
| CCCn1c(=O)[nH]c(=O)c(c1N)C(=O)COC(=O)c1nn(c2ccccc2)c(=O)c2c1cccc2 | 0 |
| O=C(Nc1ccc(c(c1)F)F)CSc1nnc(n1N)c1n[nH]c(c1)C | 0 |
| O=C(Nc1cc(ccc1N1CCCC1)S(=O)(=O)N1CCOCC1)COC(=O)c1ccccc1C | 0 |
| O=C1N[C@@H](c2ccc(cc2)N2CCOCC2)C(=C(N1)c1ccccc1)c1nc2c([nH]1)cccc2 | 0 |
| O=C(Nc1ccc(cc1F)F)CSc1nnc(n1N)c1[nH]nc2c1CCC2 | 0 |
| CCc1nn2c(c1c1ccccc1)[nH]c(c(c2=O)Cc1ccc(cc1)Br)C | 0 |
| O=C(c1ccc2c(c1)OCO2)NCCn1ncc2c1ncn(c2=O)Cc1cccc(c1)C(F)(F)F | 0 |
| C=CCSc1nc2N=C(O)C[C@H](c2c(=O)[nH]1)c1cc(OC)c(c(c1)OC)O | 0 |
| Oc1ccc(cc1)[C@H]1NC(=Nc2n1c1ccccc1n2)N | 0 |
| COc1ccc(cc1)S(=O)(=O)N1CCC[C@H](C1)C(=O)NCC(=O)Nc1ncc(s1)C | 0 |
| CCc1nn(CC(=O)NCCc2ccc(cc2)S(=O)(=O)N)c(=O)c2n1c1ccoc1c2 | 0 |
| Ic1ccccc1C(=O)/N=c/1\sc2c(n1C)c(F)ccc2 | 0 |
| Fc1cc(F)c2c(c1)s/c(=N/C(=O)c1ccc3c(c1)CCCC3)/n2C | 0 |
| COC(=O)C[C@H]1c2cc(OC)c(cc2CCN1C(=O)c1cc(Br)ccc1Cl)OC | 0 |
| Brc1cc(C)c2c(c1)c(cc(n2)c1ccccc1)C(=O)NC1CCCCC1 | 0 |
| Brc1ccc(cc1)C(=O)CSc1nc(=O)c2c([nH]1)sc1c2CCCC1 | 0 |
| Cc1ccc(c(c1)O)c1n[nH]c2c1[C@@]1(C(=O)N(c3c1cccc3)C)N(C2=O)C | 0 |
| Brc1cccc(c1)C1=Nc2ccccc2N=C(C1)c1cc(Cl)ccc1O | 0 |
| Clc1ccc2c(c1)c(=O)c(c[nH]2)c1ncc(cc1Cl)C(F)(F)F | 0 |
| COc1ccc2c(c1)nc([nH]2)SCC(=O)Nc1nonc1NC(=O)C | 0 |
| CCSc1nc(N2CCCC2)c2c(n1)n(CCNC(=O)C1c3ccccc3Oc3c1cccc3)nc2 | 0 |
| O=C1COc2c(N1)c(C)c(c(c2C)C)N | 0 |
| C[C@@H]1CCN2[C@@H](C1)C1(Cc3c2cc(Cl)cc3)C(=O)OC(OC1=O)(C)C | 0 |
| CC(=O)Nc1ccc(cc1)NC(=O)[C@H](OC(=O)c1sc2c(c1C)c(=O)n1c(n2)CCC1)C | 0 |
| O=C(Nc1ccc(c(c1F)F)F)CNC(=O)[C@H](Sc1n[nH]c(n1)N)C | 0 |
| N#CC1(C#N)[C@H]([C@H]1c1ccc(cc1F)Br)C(=O)[C@@]12C[C@H]3C[C@@H](C1)C[C@@H](C2)C3 | 0 |
| COc1cc(Br)c(cc1O)[C@H]1NC(=O)c2c(N1C)cccc2 | 0 |
| Fc1ccc(cc1)Cn1c(C)ccc1C | 0 |
| CC(n1c(C)ccc1C)C | 0 |
| OCc1sccc1C | 0 |
| CCCCn1nc(C(=O)N(Cc2cc(Br)ccc2OC)C)c2c(c1=O)cccc2 | 0 |
| CSc1ccc2c(c1)c(C)cc(=O)[nH]2 | 0 |
| O=c1[nH]c(nc2c1c(c1ccccc1)c(s2)C)CS(=O)(=O)c1ccccc1 | 0 |
| Cc1ccc(cc1)[C@@H]1C[C@@H](n2c(N1)c(Cl)c(n2)C(=O)Nc1ccc(cc1)[C@H](O)C)C(F)(F)F | 0 |
| CCCCN1C[C@H](CC1=O)c1nc2c(n1CCCOc1ccc(cc1)C)cccc2 | 0 |
| [O-]C(=O)[C@@H]1CN(CC[C@@H]1[NH2+]Cc1cccs1)C(=O)OC(C)(C)C | 0 |
| CCc1cc(=O)[nH]c(n1)C | 0 |
| COc1cc(Br)ccn1 | 0 |
| COc1cc(cc(c1O)OC)[C@@H]1[C@H]2C(=O)OC[C@@H]2[C@H](c2c1cc1OCOc1c2)Nc1ccc(cc1)F | 0 |
| [O-]c1n[nH]c(=O)c[nH+]1 | 0 |
| Fc1cnc[nH]c1=O | 0 |
| N#CC[C@@H]1CC(=O)c2c(C1)c1ccccc1[nH]2 | 0 |
| CO[C@H]1C[C@@H](C)CC[C@@H]1C(C)C | 0 |
| COc1ccccc1CNC(=O)CN1C(=O)COc2c1cc(cc2)S(=O)(=O)N1CCC(CC1)C | 0 |
| COc1ccc(cc1)c1nc(SCC(=O)NCc2[nH+]c3c(n2C)cccc3)n2c(n1)cc(n2)[O-] | 0 |
| CCOc1ccccc1NC(=O)CN1C(=O)COc2c1cc(cc2)S(=O)(=O)N1CCCCCC1 | 0 |
| Nc1cc2[nH]c(=O)c(c(c2cc1C)C)C | 0 |
| COc1ccc(c(c1)O)c1n[nH]c(c1)c1cc(Br)cc2c1OCOC2 | 0 |
| O=C(Nc1ccc2c(c1)OCCO2)CSc1nc2n[nH]cc2c(=O)[nH]1 | 0 |
| O=C(c1c(F)c(F)c(c(c1F)F)F)Oc1ccc(cc1)c1ccccc1 | 0 |
| N#Cc1cccc(c1)CSc1nnc(s1)Nc1cccc(c1)Br | 0 |
| Ic1ccc2c(c1)[C@@H]1C=CC[C@@H]1[C@@H](N2)c1ccccc1Br | 0 |
| Ic1ccc2c(c1)[C@@H]1[C@H]2c2c3c1cccc3ccc2 | 0 |
| Cc1ccc(cc1)c1cc(cc(n1)c1ccc(cc1)I)c1ccccc1 | 0 |
| O=C(c1cnn2c1N[C@@H](C[C@@H]2C(F)(F)F)c1ccccc1)Nc1cccc(c1)[C@@H](O)C | 0 |
| Clc1cc(ccc1C)n1c(C)ccc1C | 0 |
| CC(c1cc2CCc3ccc(CCc1cc2)c(c3)Br)C | 0 |
| c1ccc(cc1)C#C/C=C\c1ccc2c(c1)c1ccccc1cc2 | 0 |
| Cc1cc(C)cc(c1)C#C/C=C\c1cccc2c1cccc2 | 0 |
| Cc1cc(/C=C\C#Cc2ccccc2)cc(c1)C | 0 |
| C[C@]12C[C@@H]3C([C@@]3(C[C@H]1C2(Cl)Cl)C)(Cl)Cl | 0 |
| C1=CC=CC=CC=C1 | 0 |
| CC(NC(=O)N[C@H]1CCN2[C@@H](C1)C(=O)Nc1c(C2=O)cc(cc1)c1cccc(c1C)C)C | 0 |
| O=C(N[C@H]1CCN2[C@@H]1C(=O)Nc1c(C2=O)cc(cc1)c1ccccc1)NC1CCCCC1 | 0 |
| CC(NC(=O)N[C@H]1CCN2[C@@H]1C(=O)Nc1c(C2=O)cc(cc1)c1ccc(cc1F)F)C | 0 |
| CC1=CC[C@@H](C=C1)C(C)C | 0 |
| CC1=CC[C@]2([C@@]3([C@H]1C3)C(C)(C)CCC2)C | 0 |
| CC1=C[C@@H]2C(=C(C)CC[C@H]2C(C)C)CC1 | 0 |
| COc1c(ccc(c1OC)OC)C(=O)NCCCn1cnc2c1cccc2 | 0 |
| CN(C(=O)c1ccc(o1)COc1ccccc1)Cc1ccccc1N1CCCCC1 | 0 |
| C=C1C[C@@H]2C[C@H]1C=C2 | 0 |
| C1=C[C@@H]2[C@@H](C1)[C@@H]1C=C[C@H]2C1 | 0 |
| C[C@@H]1[C@@H](C1(Cl)Cl)C | 0 |
| FC(c1ccccc1)(F)F | 0 |
| O=C(c1c[nH]c(=O)[nH]c1=O)Nc1ccc2c(c1)sc(n2)N1CCOCC1 | 0 |
| O=C(c1cc(nc2c1cnn2C(C)C)c1ccccc1)Nc1ccccc1N1CCCC1 | 0 |
| Ic1cccc(c1)Oc1ncnc2c1ccs2 | 0 |
| COc1cc(CNn2nnnc2N)cc(c1OCC(=O)N)Br | 0 |
| CCOC(=O)C1=C(N)Oc2c([C@@H]1c1ccc3c(c1)OCO3)c(=O)[nH]c(=O)[nH]2 | 0 |
| O=c1[nH]c(nc2c1cccc2)N1CCCc2c1cccc2 | 0 |
| ClC1(Cl)C[C@@H]1Cn1cncc1 | 0 |
| O=C([C@@H](Nc1ccc(cc1)C)C)Nc1cc(Cl)ccc1Cl | 0 |
| CC(n1c(C)cc2c1cccc2)C | 0 |
| CCOc1ccccc1C(c1c[nH]c2c1cccc2)c1c[nH]c2c1cccc2 | 0 |
| COc1ccc(cc1Cl)NC(=O)c1cnn(c1n1cccc1)c1ccc(cc1)C | 0 |
| O=C(Nc1ccc2c(c1)[nH]c(=O)[nH]2)CSc1nnc(n1C)c1ccncc1 | 0 |
| Brc1cccc(c1)NC(=O)c1[nH]cc(c1)S(=O)(=O)N1CCCCC1 | 0 |
| Oc1ccccc1CNc1c(C)cccc1C | 0 |
| Brc1ccc(c(c1)/C=C\c1nc2ccccc2c(=O)[nH]1)F | 0 |
| Clc1ccc2c(c1)nc1n(c2=O)CC/C/1=C/c1ccc2c(c1)OCCO2 | 0 |
| OCCn1nc(c(c1N)[C@]1(O)c2ccccc2N(C1=O)C)C | 0 |
| CC#CCOc1cccc2c1cccc2 | 0 |
| COc1ccc(c(c1)CNC(=O)Cn1ncc2c1c1ccccc1OC2)OC | 0 |
| OC[C@]12CC[C@@H](C[C@]1(O)CC[C@H]1[C@@H]2CC[C@@]2([C@@]31CC[C@@H]2[C@H]1[C@@H](O3)OC(=O)C1)C)O | 0 |
| CCc1ccc(cc1)NC(=O)N1CCC(=CC1)c1c[nH]c2c1cccc2 | 0 |
| O=C(N(Cc1ccc(cc1)N(C)C)C[C@@H]1CCCO1)COc1cc(C)c(c(c1)C)Cl | 0 |
| Cc1ccc(c(c1)S(=O)(=O)c1cn(C)c2c(c1=O)cc(c(c2)F)F)C | 0 |
| O=c1cc(C)[nH]c2c([nH]1)cc(C)c(c2)C | 0 |
| Clc1ccc(cc1)[C@H](Cc1ccccc1Cl)CC[NH2+]Cc1ccc(cc1)N(C)C | 0 |
| Fc1ccc(cc1)[C@@H](c1ccc(cc1)Cl)CC[NH2+]Cc1ccc(cc1)N(C)C | 0 |
| O=C(C(n1c(=O)[nH]c2c(c1=O)cccc2)Cc1c[nH]c2c1cccc2)N1CCCCC1 | 0 |
| c1ccc2c(c1)OCC2 | 0 |
| COc1ccc(cc1)[C@@H]1Cc2ccccc2c2c1cccc2 | 0 |
| C#CCOc1ccc2c(c1)cccc2 | 0 |
| CCN(c1ccc(cc1)n1nc2c(n1)cc(c(c2)C)NC(=O)c1cccc(c1)C)CC | 0 |
| CCCn1c(C)cc2c1cccc2 | 0 |
| O=C(Nc1ccccc1C(=O)NC1CC1)CSc1n[nH]c(n1)N | 0 |
| Cc1n[nH]cc1c1nc2c([nH]1)cccc2 | 0 |
| O=C(Nc1ccc2c(c1)[nH]c(=O)[nH]2)CSc1nnc(n1C)c1ccoc1C | 0 |
| CCSc1nc(cc(c1C#N)C(F)(F)F)c1ccc(cc1)Br | 0 |
| Nc1c(C)[nH]nc1c1ccccc1 | 0 |
| O=c1cc([nH]c2c([nH]1)cccc2)C(C)(C)C | 0 |
| Cc1nn(c2c1CC1([C@@H]3N2CCC3)C(=O)c2c(C1=O)cccc2)c1ccccc1 | 0 |
| O=C(c1cc[nH]n1)N1CCOCC1 | 0 |
| [O-]c1ccc(cc1C(=O)c1cc(C(=O)NCCOC)c(=[NH2+])oc1)Cl | 0 |
| COc1cccnn1 | 0 |
| Oc1c2c(ccc3c2c(O)c(Br)c(=O)[nH]3)[nH]c(=O)c1Br | 0 |
| CC[NH+]([C@H](COC(c1ccccc1)(c1ccccc1)c1ccccc1)C=C)CC | 0 |
| O=C1N=C(N([C@H]1[C@@]1(O)C(=O)Nc2c1cccc2)C)N | 0 |
| Cc1ccc(c(c1)Br)OCC(=O)c1ccc(s1)Br | 0 |
| OC(c1ccccc1)(c1ccccc1)Cc1cc2c([nH]1)cccc2 | 0 |
| CCCN(c1ncnc2c1c(cn2c1ccc(cc1)OCC)c1ccccc1)CCC | 0 |
| Cc1c(C)ccn1C=C | 0 |
| Fc1cc(F)c(c(c1)Br)S(=O)(=O)Nc1ccccc1C(C)C | 0 |
| Oc1ccccc1NC1CCCCC1 | 0 |
| C[C@H]1CC[C@@H](CC1)NC(=O)Nc1cccc(c1)C(F)(F)F | 0 |
| CCn1ccc2c1ccc(c2)Br | 0 |
| Cc1cc2n(c1)cccc2 | 0 |
| C1CC[C@H]2O[C@@H](C1)CCCC2 | 0 |
| c1ccc(cc1)[C@@H]1OCc2c(C1)cccc2 | 0 |
| O=C(c1c[nH]c2c1cccc2)[C@H](c1ccccc1)Sc1ncnc2c1ccs2 | 0 |
| O=S(=O)(C(Br)(Br)Br)c1ccccn1 | 0 |
| Ic1cccc(c1)OC(=O)C1(CCCC1)c1ccccc1 | 0 |
| CC(Cn1c(N)c(C(=O)COC(=O)c2ccc(cc2O)O)c(=O)n(c1=O)C)C | 0 |
| O=C1N=NC(=O)N1C | 0 |
| CC(Oc1ccccc1C)C | 0 |
| Brc1cc(C)c(c(c1)Cl)OC(=O)c1cc2c(s1)CCCC2 | 0 |
| Fc1cccc(c1/C=C\1/CCCc2c1[nH+]c1ccccc1c2C(=O)[O-])F | 0 |
| COC1CCCC1 | 0 |
| C[C@H]1CCc2c(O1)cccc2 | 0 |
| Cc1cccn2c1cc(c2)/C=C\c1cccc2c1cccc2 | 0 |
| O=C(Nc1ccc2c(c1)[nH]c(=O)[nH]2)CSc1nnnn1c1cccc(c1)C | 0 |
| Clc1ccc(cc1)N1CCC[C@H]1C | 0 |
| Cc1ccc(cc1)c1c[nH]c(n1)N | 0 |
| C=CCn1cccc1C | 0 |
| CCO[C@@H]1C[C@H](C)CC(C1)(C)C | 0 |
| c1ccc2c(c1)Oc1ccccc1c1c2cccc1 | 0 |
| Brc1ccc(cc1)COc1ccccc1C(=O)c1ccccc1 | 0 |
| c1ccc(cc1)C=C1c2ccccc2Oc2c1cccc2 | 0 |
| OC[C@@]1(O)[C@@](C)(O)CC[C@@H]2[C@]1(C)CCCC2(C)C | 0 |
| Cc1ccc2c(c1)[C@@](C)(O)CCS2 | 0 |
| C[C@H]1CNc2c(N1C(=O)c1cccnc1)c(=O)[nH]c(n2)N | 0 |
| Cc1sc(c(n1)C)c1cc2c([nH]1)cccc2 | 0 |
| COC12c3ccccc3C(=C)c3c1c(C(=C)c1c2cccc1)ccc3 | 0 |
| CC[C@H]1Oc2ncc(cc2NC1=O)Cl | 0 |
| C=Cn1ccc(c1c1ccccc1)C | 0 |
| O=c1[nH]oc(=O)[nH]1 | 0 |
| CC1(C)OC2C[C@@H]1C[C@@H]1[C@@]2(C)C1(Cl)Cl | 0 |
| O=C(Nc1ccc(c(c1)F)F)COc1ccc(cc1)Nc1ccccc1 | 0 |
| COC1=CCCCC1 | 0 |
| CCn1c(C)ccc1C | 0 |
| C1CO[C@@H](C1)/C=C\c1ccccc1 | 0 |
| N#CC(c1ncc(cc1Cl)C(F)(F)F)(Cc1ccc(cc1Cl)Cl)C#N | 0 |
| C[N@H+](C(c1ccccc1)(c1ccccc1)c1ccccc1)CCOc1ccccc1 | 0 |
| c1ccn2c(c1)c1ccccn1cc2 | 0 |
| O=c1c2CCCCc2[nH]c2n1nc(c2c1cccc2c1cccc2)Cc1ccccc1 | 0 |
| O[C@@H]1OC[C@@H]([C@H](C1)O)O | 0 |
| CC([C@@H](c1nc2c([nH]1)cc(cc2)C)NC(=O)c1cccc(c1)Cl)C | 0 |
| N#Cc1c(Br)nc(c(c1c1ccccc1)Br)SC | 0 |
| N#CCC1(OCCO1)c1ccccc1 | 0 |
| c1ccc2c(c1)c1ccccc1c1c3n2c2ccccc2c3ccc1 | 0 |
| c1ccc2c(c1)N1CCc3c1c(c1c2cccc1)ccc3 | 0 |
| c1ccc(cc1)N1c2ccccc2Cc2c1cccc2 | 0 |
| Cc1cn(c(c1)C)c1ccccc1 | 0 |
| COc1ccccc1Nc1ccccc1C(=O)Nc1ccccc1Cl | 0 |
| CCn1c2ccccc2n2c3c1cccc3C(c1c2cccc1)(C)C | 0 |
| Brc1ccc2c(c1)s/c(=N/C(=O)[C@@]13C[C@H]4C[C@@H](C1)C[C@@H](C3)C4)/n2C | 0 |
| N#CCC(=O)N1CCCCCCC1 | 0 |
| COc1ccccc1NC(=O)C1=C(C)NC(=O)N[C@H]1c1ccc(cc1)c1ccccc1 | 0 |
| O=C1CC(C)(C)C[C@H]2[C@H]1[C@@H](c1ccc3c(c1)OCO3)[C@@H]1[C@@H](O2)CC(CC1=O)(C)C | 0 |
| CCOC(Cn1c(COc2ccc(cc2)Cl)nc2c1cccc2)OCC | 0 |
| COc1ccccc1OCc1nc2c(n1Cc1ccccc1OC)cccc2 | 0 |
| CN1Cc2c3c1cccc3ccc2 | 0 |
| Cc1ccc(c(c1)C)c1cc2n(c1)cc(cc2)C | 0 |
| Clc1cccc(c1)/C=c\1/[nH]c(=O)/c(=C\2/Nc3c(C2=O)cccc3)/[nH]c1=O | 0 |
| C=Cn1ccc2c1[C@H](C)CCC2 | 0 |
| [O-]C(=O)[C@@H]1[NH2+][C@H](/C=C\c2ccccc2)c2c(C1)c1ccccc1[nH]2 | 0 |
| Cc1cnc2c(c1)c(=O)cc([nH]2)c1ccccc1 | 0 |
| c1ccc(cc1)[C@H]1CCOc2c1cccc2 | 0 |
| CN1c2ccccc2[C@@H]2[C@@H]1CCCC2 | 0 |
| CC1(C)c2ccccc2N(c2c1cccc2)c1ccccc1 | 0 |
| C=C1c2ccccc2C2(c3c1cccc3)OC(c1c2cccc1)(C)C | 0 |
| CC1(C)c2ccccc2n2c3c1cccc3c1c2cccc1 | 0 |
| C/C=C\n1cccc1 | 0 |
| O=c1c2ccccc2[nH]c2c1[nH]c1c2cccc1 | 0 |
| CC1CCOCC1 | 0 |
| COc1c(OC)cccc1C(=O)Nc1ccc(c(c1)N1CCCC1=O)OC | 0 |
| Cc1cc(C)cc(c1)n1c(C)nc2c(c1=O)c(=O)c1c(n2C)cccc1 | 0 |
| COCCCn1c(=N)c2c(c1=O)c(N)c1c(c2N)c(=O)c2c(c1=O)cccc2 | 0 |
| CCC/C=C(\[C@H]1[NH2+][C@H](Cc2c1[nH]c1c2cccc1)C(=O)[O-])/CC | 0 |
| CC1=CC[C@@H]2C[C@@H]1[C@H](OC2)c1ccccc1 | 0 |
| O=C([C@H]1CN(CCN1C(=O)c1cccs1)C(=O)c1cccs1)N[C@H]1CCCNC1=O | 0 |
| O=C([C@H]1CN(CCN1C(=O)c1cccs1)S(=O)(=O)c1ccc(cc1)F)N[C@H]1CCCNC1=O | 0 |
| COc1ccc(cc1)c1ccc2c(c1)C(=O)N1CCN(C[C@H]1C(=O)N2)C(=O)Nc1ccccc1 | 0 |
| Cc1c2ccccc2n2c1[C@](C)(CCC2)c1ccccc1 | 0 |
| C[C@H]1CCn2c1cc(c2)c1ccccc1 | 0 |
| C[C@@H]1C[C@@]2(C)OC[C@@H]1[C@@H](C2)C | 0 |
| CC1=CC[C@@H](CC1)[C@H]1OCC2[C@H](C1C(=C[C@H]2C)C)C | 0 |
| COc1ccc(cc1)[C@@H](C(=O)c1ccccc1)c1ccc(cc1)Br | 0 |
| CC1=CC[C@@H]([C@H](C1)C)[C@H]1O[C@@H](C)CC(=C)C1 | 0 |
| Cn1c(C#Cc2ccccc2)cc2c1cccc2 | 0 |
| c1ccc(cc1)[C@H]1CCCn2c1cc1c2cccc1 | 0 |
| c1ccc(cc1)[C@H]1CN2CCCc3c2c(C1)ccc3 | 0 |
| c1ccc(cc1)N1C[C@@H]2CCC[C@@H]2c2c1cccc2 | 0 |
| c1ccc(cc1)NC1=C(CN2c3c1cccc3CCC2)c1ccccc1 | 0 |
| c1ccc(cc1)C1(CCn2c3c1cccc3c1c2cccc1)c1ccccc1 | 0 |
| Cn1c2ccccc2c2c1C(CC2)(c1ccccc1)c1ccccc1 | 0 |
| Cn1c2CCc3c(c2c2c1cccc2)cccc3 | 0 |
| Cc1cccc(c1)N1CCCCC1 | 0 |
| Cc1ccc2c(c1)[C@@H](CCN2C)n1c2ccccc2c2c1cccc2 | 0 |
| Cc1ccc(cc1)[C@@H]1CCn2c3c1cccc3c1c2cccc1 | 0 |
| Cc1ccc(cc1)[C@@H]1CCc2c1n(C)c1c2cccc1 | 0 |
| Cc1ccc(cc1)[C@@H]([C@H]1Cc2c(N1)cccc2)O | 0 |
| Cc1ccc(cc1)N1C2=C(CCc3c2cccc3)CC2=C1c1ccccc1CC2 | 0 |
| OCc1cc(C)cc(n1)C | 0 |
| C[C@@H]1Nc2ccccc2[C@@H](C1)n1c2ccccc2c2c1cccc2 | 0 |
| C[C@H]1CCc2c(N1C)cccc2 | 0 |
| C[C@H]1CCc2c3N1CCCc3ccc2 | 0 |
| C[C@H]1CC(c2ccccc2)(c2ccccc2)c2c3n1c1ccccc1c3ccc2 | 0 |
| Cn1c2ccccc2c2c1[C@](C)(CC2)c1ccccc1 | 0 |
| C[C@H]1[C@H](CN(c2c1cccc2)C)c1ccccc1 | 0 |
| C[C@H]1c2c([C@H](c3c1c1ccccc1n3C)C)c1c(n2C)cccc1 | 0 |
| C[C@@H]1C[C@](c2c1c1ccccc1n2C)(C)c1ccccc1 | 0 |
| C[C@@H]1CN2CCCc3c2c([C@H]1c1ccccc1)ccc3 | 0 |
| CN1C[C@H](Cc2c1cccc2)c1ccccc1 | 0 |
| CN1C[C@@H]2CCC[C@@H]2c2c1cccc2 | 0 |
| CN1C[C@@H]2CN(C)c3c([C@H]2c2c1cccc2)cccc3 | 0 |
| CC[C@H]1[C@H](C)Cc2c(N1C)cccc2 | 0 |
| CC[C@H]1[C@H](C)Cc2c3N1CCCc3ccc2 | 0 |
| CC[C@H]1CCN(c2c1cc(C)cc2)C | 0 |
| CO[C@H]1CCCc2c1cccc2 | 0 |
| CCC[C@H]1CN(C)c2c(C1)cccc2 | 0 |
| CCCCC#C[C@@H]1CCCO1 | 0 |
| CCCCC#C[C@@H]1CCCCO1 | 0 |
| CN1c2ccccc2C(=C([C@H]1c1ccccc1)C)c1ccccc1 | 0 |
| Cn1c(cc2c1cccc2)c1ccc2c3c1cccc3C(C2(C)C)(C)C | 0 |
| C1COC(=O)[C@@H]1C#N | 0 |
| CCn1c(N)nc2c1ccc(c2)O | 0 |
| CN1[C@H](C[C@@](c2c1cccc2)(C)C(=C)C)c1ccccc1 | 0 |
| [O-]C(=O)C1(O)CC[NH2+]CC1 | 0 |
| C1=CN2[C@@H](C=C1)C=CC=C2 | 0 |
| C/C=C/Oc1ccccc1 | 0 |
| N#C[C@H](C(=O)c1ccc(cc1)c1ccccc1)c1[nH]c(=O)c2c(n1)sc(c2C)C | 0 |
| N#Cc1ccccc1NC(=O)C[C@@H](C(=O)[O-])[NH2+]Cc1cccnc1 | 0 |
| Clc1cccc(c1)N(C(=O)Nc1ccccc1)Cc1nnc2n1CCCCC2 | 0 |
| O=C(c1ccccc1)N[C@]1(Nc2n(C1=O)c1c(n2)cccc1)C(F)(F)F | 0 |
| Clc1ccc2c(c1)c(=O)c1c(o2)nc(n2c1nc1c2cccc1)C(C)C | 0 |
| CCc1nc2oc3c4ccccc4ccc3c(=O)c2c2n1c1ccccc1n2 | 0 |
| Fc1ccccc1c1nc2oc3ccccc3c(=O)c2c2n1c1ccccc1n2 | 0 |
| Cc1cccc(c1)c1nc2n(C)c3ccccc3c(=O)c2c2n1c1ccccc1n2 | 0 |
| O=C1c2ccccc2c2c1c([nH]n2)c1ccccc1 | 0 |
| C=CCn1ccc2c1cccc2 | 0 |
| NC(=O)[C@H]1CCC=CC1 | 0 |
| O=C1CC(=O)N=C(N1)O | 0 |
| FC1(F)C(F)(F)OC1(F)F | 0 |
| O=C1Nc2c(C31Nc1c(N3)cccc1)cccc2 | 0 |
| Nc1cc2ccccc2c(c1)N | 0 |
| O[C@H]1CO[C@]2([C@@H]1OC(=O)[C@]2(O)Cc1cn(c2c1cccc2)C)O | 0 |
| Cc1cccc(c1N1CCCCC1)C | 0 |
| Cc1ccc2c3c1CCCN3CCC2 | 0 |
| CCCCNc1cccc(c1)C | 0 |
| C1CC2CC[C@H](C1)O2 | 0 |
| CCC1=[N+](C)[C@@H](CC1(c1ccccc1)c1ccccc1)C | 0 |
| C/C=C(/[C@@H]1CCC(O1)(C)C)\C | 0 |
| COc1cccc2c1nc1cc(Cl)ccc1c2Cl | 0 |
| O=c1[nH]c(Cc2ccccc2)nc2c1C1(CCCC1)Cc1c2cccc1 | 0 |
| CCCN(C(=O)Cn1ncc2c1c1cc(C)ccc1OC2)c1ccc(cc1)CC | 0 |
| CNC(=O)CSc1nc(N)c(c(=O)[nH]1)NC(=O)c1ccc(cc1)F | 0 |
| CC(NC(=O)CSc1nc(N)c(c(=O)[nH]1)NC(=O)c1ccccc1)C | 0 |
| CCCNC(=O)CSc1nc(N)c(c(=O)[nH]1)NC(=O)c1ccccc1 | 0 |
| CCN1CCn2c3[C@@H]1CCCc3c1c2c(C)ccc1 | 0 |
| O=c1[nH]c(nc2c1C1(CCCCC1)Cc1c2cccc1)c1ccccc1 | 0 |
| Brc1ccc(cc1)c1nnc(s1)c1ccccc1Br | 0 |
| O=C1N[C@@](C)(Oc2c1cccc2)c1ccco1 | 0 |
| Brc1ccccc1c1cc2n(c1)c1c(n2C)cccc1 | 0 |
| O=c1[nH]nc2c(n1)n(C)c1c2cccc1 | 0 |
| Cc1ccc2c(c1)CCCN2C | 0 |
| Brc1cccc2c1OC(C2)(C)C | 0 |
| c1ccc2c(c1)COC12c2ccccc2c2c1cccc2 | 0 |
| COc1ccc(cc1OCc1ccccc1)C[N@H+]1CC[C@@H](CC1)Cc1ccccc1 | 0 |
| COc1cc(ccc1OCc1ccccc1)C[N@H+](Cc1cccc2c1cccc2)C | 0 |
| CCOc1ccccc1N1CC[NH+](CC1)Cc1ccc(cc1)N(CC)CC | 0 |
| CN(C(=O)[C@]1(C)C[C@@H]2C[C@H]1CC2)C | 0 |
| Clc1cc(Cl)c(c2c1OC(C2)(C)C)C | 0 |
| C[C@H](OC(C)(C)C)C#C | 0 |
| O=C(N1c2ccccc2Sc2c1cccc2)CSc1nc(C)c(c(=O)[nH]1)C | 0 |
| Clc1ccc(cc1)[C@@H]1Nc2cc(nn2[C@@H](C1)C(F)(F)F)/C(=N/C(C)C)/O | 0 |
| [O-]C(=O)[C@H]1C[C@H](O)CC[NH2+]1 | 0 |
| COc1cccc(c1)C[N@H+]([C@H](C(C)C)C)Cc1cccn1Cc1ccccc1C | 0 |
| O=C1C[C@@H](CN1Cc1ccccc1)c1nc2c(n1CCOc1ccccc1C)cccc2 | 0 |
| c1ccc2c(c1)n(cc2)c1c2ccccc2nc2c1cccc2 | 0 |
| c1ccc(cc1)[C@@H]1C[C@H](c2ccccc2)n2c3[C@@H]1CCCc3c1c2cccc1 | 0 |
| Clc1ccc(c(c1)Cl)[C@H]1Nc2c(O1)c(cc(c2)C(C)(C)C)C(C)(C)C | 0 |
| CCc1cc2cc3OCOc3cc2nc1SCC(=O)Nc1c[nH]c(=O)[nH]c1=O | 0 |
| Clc1ccc(cc1)C(=O)/N=C\1/S[C@H]2[C@@H](N1C)c1ccccc1CC2 | 0 |
| N#C[C@H]1[C@@H](c2ccc(cc2)C(=O)OC)[C@@H](C(=O)N)C(=O)N[C@@]1(O)c1ccccc1 | 0 |
| Clc1ccc(c(c1)Cl)Nc1ccc(cc1Cl)Cl | 0 |
| Cn1c2cc3ccccc3cc2n(c2c1cc1ccccc1c2)C | 0 |
| CCC1=C(C)CN(c2c1cccc2)C | 0 |
| Nc1ccccc1c1cc[nH]n1 | 0 |
| CO[C@@]12C=CC(c3c1cccc3)c1c2cccc1 | 0 |
| CO[C@@H]1c2ccccc2[C@@H]2[C@@H]1Cc1c2cccc1 | 0 |
| CC1(C)O[C@@H]2[C@H](O1)CCCCc1ccc(CCCC2)cc1 | 0 |
| CO/C(=C/c1ccccc1)/C | 0 |
| C[C@@H]1Cc2c(O1)cccc2 | 0 |
| Cc1ccc(n1C1c2ccccc2Oc2c1cccc2)C | 0 |
| N#CC(=C1CCC2=N[C@@]34N([C@H]2C1)C1=C(CCCC1)[C@@H]([C@@H]3CCCC4)C1CCCCC1)C#N | 0 |
| CCO[C@@H]1CCc2c(C1)cccc2 | 0 |
| COc1cc(OC)cc(c1)C(=O)Nc1ccc2c(c1)c(C)cc(n2)N1CCC(CC1)C | 0 |
| Ic1ccc(cc1)[C@@H](c1c(O)n[nH]c1C)c1c(C)n[nH]c1O | 0 |
| Cc1ccccc1N=c1c2ccccc2n(c2c1cccc2)C | 0 |
| COc1ccc(cc1)[C@H]1Nc2ccc(cc2C(=C1)c1ccccc1)Cl | 0 |
| N#Cc1cc2OCOc2cc1Cl | 0 |
| O=C([C@@]1(C)CC1(Br)Br)Oc1ccc(cc1)C(C)(C)C | 0 |
| O=C([C@]1(C)CC1(Br)Br)Oc1ccc(c(c1)C)Cl | 0 |
| CCN(c1ccc(cc1)[C@@H]1C(=C(C)Nc2n1c1ccccc1n2)C(=O)OC(C)C)CC | 0 |
| c1ccc(cc1)[C@@H]1C=C(c2ccccc2)N2[C@@]3([C@@H]1CCCC3)Nc1c2cccc1 | 0 |
| Brc1nsc2c1c(c1c(n2)CCCCC1)C(F)(F)F | 0 |
| Clc1cccc(c1)[C@H]1OCCN(C(=O)c2c1c1ccccc1[nH]2)Cc1ccccc1 | 0 |
| O=C1c2ccccc2c2c3c1cccc3c(cc2)N1CCCC1 | 0 |
| Nc1ccc2c(c1)nc1n2ncc2c1cccc2 | 0 |
| Clc1ccc(cc1)CNc1ccccc1O | 0 |
| O=C1C[C@H](c2ccc3c(c2)c2ccccc2n3C)c2c(O1)c1ccccc1n(c2=O)C | 0 |
| O=C1C[C@H](c2ccc3c(c2)c2ccccc2n3C)c2c(O1)c1ccccc1oc2=O | 0 |
| O=C([C@@]12C[C@H]3C[C@@H](C1)C[C@@H](C2)C3)OCc1ccc(cc1)I | 0 |
| c1ccc(cc1)C[N@H+](Cc1cccc(c1)OCc1ccccc1)CCc1ccccc1 | 0 |
| O=C(Nc1[nH]nc(c1)C)CSc1nc2[nH]ncc2c(=O)n1c1ccccc1C | 0 |
| C=CCN1c2ccc(cc2[C@](C1=O)(O)CC(=O)c1ccccc1O)Br | 0 |
| Fc1c(F)c(c(c(c1N1C(=O)c2c(S1(=O)=O)cccc2)F)F)C(F)(F)F | 0 |
| c1ccc(cc1)c1c(c2ccccc2)c2n3c1ccc3ccc2 | 0 |
| COc1ccc(cc1)[C@]1(O)c2cccc(c2C(=O)c2c1c(Cl)ccc2)Cl | 0 |
| Clc1ccc(cc1)COc1ccc2c(c1)C(=CC(N2)(C)C)C | 0 |
| Brc1ccc(cc1n1c(C)ccc1C)[C@@]12C[C@H]3C[C@@H](C1)C[C@@H](C2)C3 | 0 |
| C=C[C@H]1CC[C@H]([C@H](O1)C)C=C | 0 |
| CCc1ccc(cc1)N1C(=O)Nc2c([C@]1(O)C(=O)N[C@@H]1CCC[C@@H]([C@@H]1C)C)cccc2 | 0 |
| C[C@@]12C[C@@H]3C[C@]1(C)C[C@H](C2)O3 | 0 |
| Oc1ccc(c(c1)[C@H](C1=C(C)[N+](=[N+](C1=O)c1ccccc1)[CH2-])C1=C(C)[N+](=[N+](C1=O)c1ccccc1)[CH2-])O | 0 |
| O=C1Nc2c([C@@]1(O)CC(=O)c1ccc3c(c1)OCCO3)c(Cl)ccc2C | 0 |
| Nc1ccc2c(c1)nc(n2C)c1ccco1 | 0 |
| C1C[C@H]2CCCN3[C@@H]2[C@@H](C1)c1ccccc31 | 0 |
| COC1(CCCCC1)c1ccccc1 | 0 |
| c1ccc(cc1)[C@@H]1c2ccccc2O[C@]2([C@@H]1C2)c1ccccc1 | 0 |
| Clc1ccc(cc1C(F)(F)F)S(=O)(=O)N[C@@]12C[C@H]3C[C@@H](C1)C[C@@H](C2)C3 | 0 |
| ClC1(c2ccccc2)c2ccccc2N(c2c1cccc2)c1ccccc1 | 0 |
| Clc1ccc2c(c1)n(c1ccccc1)c1c(c2=O)cccc1 | 0 |
| CO[C@@H]1C[C@H]2C([C@]1(C)CC2)(C)C | 0 |
| O[C@@H]1CC[NH2+][C@@H]1C(=O)[O-] | 0 |
| [O-]C(=O)[C@@H]1[C@@H](O)CNC(=[NH+]1)C | 0 |
| O=S1Nc2ccc(cc2[C@@H]2[C@@H]1[C@H]1CC[C@@H]2C1)C | 0 |
| Brc1ccccc1/C=C/1\S[C@@H]2N(C1=O)[C@@H]1[C@H](N2)[C@H](C)[C@H](CC1)C | 0 |
| Clc1ccc(c(c1)Cl)/C=C/1\S[C@H]2N(C1=O)[C@H]1[C@@H](N2)C[C@H]([C@H](C1)C)C | 0 |
| Brc1ccc2c(c1)C=Nc1c(O2)ncnc1N1CCCCC1 | 0 |
| O=C(Nc1cccc(c1)NC(=O)C)CSc1nncc(=O)n1N | 0 |
| Clc1ccc(c(c1)Cl)Cn1c2ccccc2c2c1cccc2 | 0 |
| C#CCOC(C)(C)C | 0 |
| Cc1ccc2c(c1)OC(C2)(C)C | 0 |
| COc1cccc(c1)c1cccc(c1)F | 0 |
| O=C(NCc1ccc2c(c1)OCO2)CSc1[nH]c(=O)c2c(n1)[nH]nc2 | 0 |
| O=C(Nc1ccc(cc1)NC(=O)C)CSc1[nH]c(=O)c2c(n1)[nH]nc2 | 0 |
| C[C@H]1CC[C@@H](N1c1ccccc1)C | 0 |
| Clc1cc(Cl)c(cc1n1cccc1)Cl | 0 |
| O=C1N[C@@](C[C@H]1c1c[nH]c2c1cccc2)(O)C(F)(F)F | 0 |
| CCN([C@H](c1nnnn1C(C)(C)C)c1ccc(cc1)Cl)Cc1ccccc1 | 0 |
| NC(=O)CO[C@@H]1CO[C@H]2[C@@H]1OC[C@@H]2NS(=O)(=O)c1ccc(cc1)NC(=O)C | 0 |
| CC(c1nc(SCc2cc(=O)[nH]c3c2cccc3)c2c(n1)sc1c2CCCC1)C | 0 |
| O=c1cc(CSc2nc(nc3c2c2CCCCc2s3)C2CC2)c2c([nH]1)cccc2 | 0 |
| C(c1ccno1)C#N | 0 |
| [O-]C(=O)[C@H]1C[C@@H](O)C[NH2+]C1 | 0 |
| COc1nc(cs1)Br | 0 |
| C1CCC(CC1)[C@H]1Nc2ccccc2C(O1)(c1ccccc1)c1ccccc1 | 0 |
| CC(C#CC[NH+]1CCCCC1)(OCCOC(c1ccccc1)c1ccccc1)C | 0 |
| c1ccc2n(c1)cc(c2)[C@@]12C[C@H]3C[C@@H](C1)C[C@@H](C2)C3 | 0 |
| Cc1ccc2c(c1)[C@H](c1ccccc1)c1c(O2)cccc1 | 0 |
| c1ccc(cc1)n1c2CCCCCc2c2c1cccc2 | 0 |
| Fc1ccc(cc1)[C@@H]1Nc2ccc(cc2[C@@H]2[C@@H]1CC=C2)OC(F)(F)F | 0 |
| c1ccc(cc1)[C@@H]1Nc2ccccc2C(O1)(c1ccccc1)c1ccccc1 | 0 |
| O=C(C[C@@H](C(=O)[O-])[NH2+]Cc1cccnc1)NCc1ccco1 | 0 |
| Cn1c2c3ccccc3Cc2c2c1cccc2 | 0 |
| Cn1c2ccccc2c2c1c1ccccc1CCC2 | 0 |
| Nc1ccc2c(c1)c(=O)c1c([nH]2)cccc1 | 0 |
| O[C@H]1[C@H](O)CO[C@H]1O | 0 |
| COC(=O)c1sc2c(c1)CCC2 | 0 |
| Clc1ccc2c(c1)C1NC(=O)N([C@@](O2)(C1)C)c1cccc(c1)C(=O)NCc1ccc2c(c1)OCO2 | 0 |
| O=C(c1ccccc1I)Oc1ccc(cc1)C(C)C | 0 |
| Brc1ccc(cc1)C(=O)/N=c\1/sc2c(n1C)c(C)cc(c2)C | 0 |
| Brc1ccc(cc1)N1CCc2c1nc(SCc1ccccc1)nc2C | 0 |
| Cc1ccc(c2c1c1ccccc1n2Cc1ccccc1)C | 0 |
| C/C=C/[C@H]1O[C@@H](C)CC[C@H]1C(=C)C | 0 |
| CC1(C)O[C@H]2c3ccccc3[C@@]3([C@H]2C[C@H]1CC3)C | 0 |
| Brc1ccc(c(c1)[C@@H]1[NH2+][C@@H](Cc2c1[nH]c1c2cccc1)C(=O)[O-])O | 0 |
| COc1ccc(cc1)C[N@@H+](CC[C@@]1(CCOC(C1)(C)C)Cc1ccccc1)CC | 0 |
| CC1=CCC[C@@H](C1)[C@H]1OC[C@H]2C[C@@H]1C(=C)CC2 | 0 |
| Clc1ccc2n(c1)c(C(=O)[O-])c([nH+]2)C(F)(F)F | 0 |
| C[C@@H]1CC[C@@H]2[C@@H](C1)O[C@@H](CC2=C)c1ccccc1 | 0 |
| CC([N@@H+](Cc1ccccc1)CC[C@@]1(CCOC(C1)(C)C)Cc1ccccc1)C | 0 |
| C=C[C@H]1O[C@H]2CCCC[C@H]2C2=CCCC[C@@H]12 | 0 |
| c1ccc2c(c1)C(c1cc3c(o1)cccc3)c1c(O2)cccc1 | 0 |
| O=c1[nH]c(C)c2c(c1)c1ccccc1[nH]2 | 0 |
| Fc1ccc(cc1)[C@@H]1Sc2ccccc2N=C2[C@H]1C(=O)c1c2cccc1 | 0 |
| FC1(F)[C@H]2[C@H]3C=C[C@@H]([C@H]2C1(F)F)O3 | 0 |
| COc1ccc2c(c1)ccc(c2)[C@@]12C[C@H]3C[C@@H](C1)C[C@@H](C2)C3 | 0 |
| CCCn1c(C)ccc1C | 0 |
| C#C[C@]1(C)CCCC(O1)(C)C | 0 |
| O=C([C@@]12C[C@H]3C[C@@H](C1)C[C@@H](C2)C3)Nc1c2CCN(c2nc2c1cccc2)c1ccccc1 | 0 |
| C[C@H]1Cc2c3n1c(C)c(c3ccc2)c1ccccc1 | 0 |
| N#Cc1c(C)cc(n2c1nc1c2cccc1)Nc1cc(Cl)c(cc1Cl)Cl | 0 |
| c1ccc2c(c1)nc1c(n2)c2ccccc2c2c1cccc2 | 0 |
| OCCc1ccccn1 | 0 |
| Cn1c2ccccc2c(=c2c3ccccc3n(c3c2cccc3)C)c2c1cccc2 | 0 |
| Clc1nc2ccccc2nc1c1cccc2c1cccc2 | 0 |
| COC1=CCc2c1cccc2 | 0 |
| COC1=C[C@H](c2c1cccc2)C | 0 |
| COc1ccc2c(c1)CC2 | 0 |
| c1cc2CCc3n4c2c(c1)CCc4cc3 | 0 |
| CC(c1cc2cccc3c2n2c1ccc2c(c3)C(C)(C)C)(C)C | 0 |
| Cc1cc2cccc3c2n2c1ccc2c(c3)C | 0 |
| c1cc2ccc3n4c2c(c1)ccc4cc3 | 0 |
| c1ccc(cc1)c1cc2cccc3n2c2c1ccc2c(c3)c1ccccc1 | 0 |
| CC[C@]1(C)CCCC(O1)(C)C | 0 |
| [O-]C(=O)[C@H](C[NH+](C)C)Oc1ccc(cc1)Cl | 0 |
| CCC1(CC)O[C@@H](Nc2c1cccc2)c1ccc(c(c1)Cl)Cl | 0 |
| COc1cccc(c1)N1C(=O)Nc2c([C@@]1(O)C(=O)NC(C)C)cc(cc2)Br | 0 |
| CCc1ccc(cc1)N1C(=O)Nc2c([C@]1(O)C(=O)NCc1ccccc1)cc(cc2)Br | 0 |
| CCCNC(=O)[C@@]1(O)N(C(=O)Nc2c1cc(Br)cc2)c1ccc(c(c1)C)C | 0 |
| COc1cccc(c1)N1C(=O)Nc2c([C@]1(O)C(=O)NC1CCCCCCC1)cc(cc2)Br | 0 |
| O=C1Nc2ccccc2[C@@](N1c1ccc(c(c1)C)C)(O)C(=O)NCCC1=CCCCC1 | 0 |
| C[C@@H]1CCCC[C@@H]1NC(=O)[C@]1(O)c2ccccc2NC(=O)N1c1ccc(c(c1)Cl)Cl | 0 |
| C[C@@H](c1ccccc1)NC(=O)[C@]1(O)c2ccccc2NC(=O)N1c1ccc(c(c1)Cl)Cl | 0 |
| O=C1Nc2ccccc2[C@](N1c1ccc(c(c1)C)C)(O)C(=O)NC1CCCCCCC1 | 0 |
| C[C@@H]1C[C@H](C)[C@@H]2[C@H](C1)N1[C@@H](N2)S/C(=C/c2ccc(cc2)C(C)(C)C)/C1=O | 0 |
| N#CC1(C#N)[C@H]2C=Cc3c(N2[C@@H]([C@H]1c1ccccc1F)C(=O)C)cccc3 | 0 |
| C1CCC2=C(C1)C=CC2 | 0 |
| COC(=O)c1[nH]cc(c1)C | 0 |
| F[C@H](C(F)(F)F)CC(F)(F)F | 0 |
| O=c1[nH]c(=O)[nH]c(c1)C(=O)OCC(=O)N1CCC(CC1)Cc1ccccc1 | 0 |
| Clc1ccc(c(c1)Br)OCC(=O)c1cc(C)c(cc1C)C | 0 |
| Clc1ccc(s1)C(=O)CSc1[nH]nc(n1)c1ccccc1Br | 0 |
| COCCN(c1c(=O)[nH]c(=O)n(c1N)Cc1ccccc1)C(=O)Cn1ncc(c(c1=O)Cl)Cl | 0 |
| Cc1ccc(o1)C(=O)NC[C@H](c1c[nH]c2c1cccc2)c1ccccc1 | 0 |
| Brc1cccc(c1)OCC(=O)c1ccc(cc1)c1ccccc1 | 0 |
| CC[NH+](CCNc1sc(c(n1)c1cccs1)CC(=O)[O-])CC | 0 |
| O=C(c1ccc2c(c1)c(C)c([nH]2)C)Nc1ccc(cc1)OC(F)(F)F | 0 |
| N#CCCN(c1cc(C)cc(c1)C)C(=O)COC(=O)c1sc(c(c1)Br)Br | 0 |
| COc1ccc(cc1S(=O)(=O)NCC[NH+](C)C)C(=O)[O-] | 0 |
| Ic1ccc(cc1)C(=O)OCc1ccc(cc1Cl)Cl | 0 |
| CC(CN1c2ccccc2C(=O)C1=O)C | 0 |
| Cc1ccc(n1C1CCCCC1)C | 0 |
| O=C(Nc1ccc(cc1)C(=O)N)COC(=O)c1cc(ccc1C)S(=O)(=O)N1CCOCC1 | 0 |
| Cc1ccc(cc1)c1ccc(cc1)OC(=O)c1ccccc1I | 0 |
| Cn1c2nc(C3CCCCC3)n3c(c2c(=O)c2c1cccc2)nc1c3cccc1 | 0 |
| O=c1c(c2oc3c(n2)cccc3)c2oc3c(n2c2c1cccc2)cccc3 | 0 |
| Brc1cccc2c1C(F)(F)c1c(C2(F)F)cccc1 | 0 |
| Fc1c(C)c(F)c(c2c1c(F)c(c(c2F)F)F)F | 0 |
| FC1(F)[C@@H]2[C@@H]3C=C[C@H]([C@@H]2C1(F)F)C3 | 0 |
| COc1ccc(cc1)C1CC1 | 0 |
| OC1(CCCCC1)n1nnc2c1cccc2 | 0 |
| Cc1cc(C)c2c(c1)c1CCNC(=O)c1[nH]2 | 0 |
| CCc1c2ccccc2c(c2c1cccc2)Br | 0 |
| CCc1cc2CCc3ccc(CCc1cc2)cc3 | 0 |
| Cc1cc2CCc3ccc(CCc1cc2Br)cc3 | 0 |
| COc1cc2CCc3ccc(CCc1cc2)cc3 | 0 |
| Cc1ccc(c2c1n(C)c1c2cccc1)C | 0 |
| Brc1cc2c(C(C)C)c3ccccc3c(c2cc1Br)Br | 0 |
| Brc1cc2CCc3ccc(CCc(c1)c2)cc3 | 0 |
| C=C[C@H]1c2ccccc2C=Cc2c1cccc2 | 0 |
| Cc1ccc(cc1)S(=O)(=O)Oc1ccc2c(c1I)cccc2 | 0 |
| Clc1ccc(cc1)C(=O)Oc1ccc2c(c1I)cccc2 | 0 |
| N#Cc1cc(Br)c(c(c1)Br)OCc1c(F)cccc1Cl | 0 |
| C[C@]1(CC(C)(C)c2c3c1cccc3ccc2)c1cccc2c1cccc2 | 0 |
| CC(CCn1c2c(Br)cc(cc2c2c1ccc(c2)Br)Br)C | 0 |
| O=c1[nH]c2ccccc2[nH]c2c1cccc2 | 0 |
| c1ccc(cc1)C1=C[C@H](c2c(O1)cccc2)c1ccccc1 | 0 |
| Cc1ccccc1[C@@H]1C=C(Oc2c1cccc2)c1ccccc1 | 0 |
| Fc1c(C#Cc2ccccc2)c(F)c(c(c1F)C(F)(F)F)F | 0 |
| Cc1n2CCc3c2c(c1C)ccc3 | 0 |
| O=C(Nc1cccc(c1)C(F)(F)F)Nc1ccc(cc1)C(C)C | 0 |
| Cc1c(C)cc(c(c1c1c(C)c(C)cc(c1C)C)C)C | 0 |
| Clc1cccc2c1c(Cc1ccccc1)c1ccccc1c2Br | 0 |
| Cc1cc2CCc3cc(c(CCc1cc2C)cc3C)C | 0 |
| Brc1cc2CCc3ccc(CCc1cc2)cc3Br | 0 |
| Cc1c(C)c(C)c(c2c3c(c12)c(C)c(c(c3C)C)C)C | 0 |
| Cc1ccc2c(c1)C=CC2(C)C | 0 |
| Brc1cc(C)c2c(c1C)c(C)c(c(c2C)C)C | 0 |
| Cc1c(C)c(C)c2c(c1C)CC2 | 0 |
| Cc1c(C)c(C)c(c(c1C)C(Cl)(Cl)Cl)C | 0 |
| COC1(CCCC1)c1ccccc1 | 0 |
| Cc1c(F)c(C)c(c(c1C)C(Cl)(Cl)Cl)C | 0 |
| C1[C@@H]2C[C@@H]3[C@@H]([C@H]1C[C@H](C2)C3)C1[C@@H]2C[C@@H]3C[C@H]1C[C@H](C2)C3 | 0 |
| Clc1ccc(cc1)C#C[C@@H](c1ccccc1Br)Cc1ccccc1Br | 0 |
| Clc1ccc2c3c1ccc1c3c(cc2)c(cc1Cl)Cl | 0 |
| Fc1c2c(c(c(c1F)F)F)[C@H]1[C@@]3([C@H]2C=C3)C=C1 | 0 |
| Clc1ccc(cc1)OC1c2ccccc2c2c1cccc2 | 0 |
| CC1=CC[C@H]([C@H](C1)C)[C@H]1OCC2CC1C(=C[C@@H]2C)C | 0 |
| c1ccc(c(c1)C#Cc1ccccc1)C#Cc1ccccc1 | 0 |
| CCc1ccc(cc1)NC(=O)Nc1cccc(c1)C(F)(F)F | 0 |
| CC1(C2CCCCC2)c2ccccc2c2c1cccc2 | 0 |
| Cc1ccc(c2c1C(C)(C)CCC2(C)C)C | 0 |
| OC(c1ccccc1)(c1ccccc1)Cc1c[nH]c2c1cccc2 | 0 |
| COC1=C(C)CC=CC1 | 0 |
| O=C1Nc2c([C@@]1(O)c1cc3c([nH]1)cccc3)cccc2 | 0 |
| O=C1Nc2ccccc2[C@](N1c1ccc(c(c1)Cl)Cl)(O)C(=O)NCCc1ccccc1 | 0 |
| Fc1cccc(c1Cn1c(C)nc2c(c1=O)c1CCCCc1s2)Cl | 0 |
| Brc1cccc(c1)Cn1cnc2c(c1=O)c1CCCc1s2 | 0 |
| Ic1ccc(cc1)S(=O)(=O)Oc1ccc(c(c1)C)C(C)C | 0 |
| O=C(c1ccc(cc1)C(C)(C)C)Nc1ccc(cc1)c1nnc(n1C)SCC(=O)N1CCOCC1 | 0 |
| COC(=O)c1ccc2c(c1)nc(n(c2=O)Cc1ccco1)SCC(=O)Nc1[nH]nc(c1)C | 0 |
| O=c1cc([nH]c2n1nc(c2c1cccc2c1cccc2)Cc1ccccc1)c1ccccc1 | 0 |
| O=C(CSc1nc2ccsc2c(=O)n1NC(=O)c1ccccc1)NC[C@@H]1CCCO1 | 0 |
| COc1cc(ccc1OC)[C@H]1C[C@H](Nc2n1ncn2)c1cccc(c1)Br | 0 |
| COc1ccc2c(c1C[N@@H+](Cc1ccccc1)CCc1ccccc1)cccc2 | 0 |
| Ic1ccc(cc1)OCCCCCC[NH+]1CCCC1 | 0 |
| C1CCC(=CC1)n1c2ccccc2c2c1cccc2 | 0 |
| CC(c1ccc2c(c1)c1cc(ccc1n2C)C(C)(C)C)(C)C | 0 |
| COc1ccc(cc1N(S(=O)(=O)C)CC(=O)Nc1ccccc1C(=O)N1CCOCC1)Cl | 0 |
| O=C(c1cc2c(o1)cccc2)Nc1cc(C)c(cc1C)I | 0 |
| N#Cc1ccc(c(c1)F)C(=O)Nc1ccc(cc1C(F)(F)F)I | 0 |
| O=C(c1ccccc1I)OCc1ccc2c(c1)cccc2 | 0 |
| O=C(Nc1ccc(c(c1)Cl)N1CCOCC1)CSc1nnc(n1C)COc1ccccc1C | 0 |
| Clc1ccc2c(c1)cc([nH]2)CNC(=O)c1cnn2c1N[C@@H](C[C@@H]2C(F)(F)F)c1ccco1 | 0 |
| Clc1ccc(cc1)OCC(=O)N1CCN(CC1)C(=O)COc1ccc(cc1)Br | 0 |
| Cc1cc(n(n1)C(=O)c1sc(c(c1)Br)Br)C | 0 |
| Cc1ccc(cc1)n1nc(c(c1c1ccc(cc1)Br)C)c1ccc(cc1)Br | 0 |
| Fc1ccc(cc1)n1cc(c2c1ncnc2Nc1ccccc1C)c1ccccc1 | 0 |
| FC(n1c2ccccc2c2c1cccc2)F | 0 |
| C[C@@]1(C=C(Oc2c1cccc2)c1ccccc1)c1ccccc1 | 0 |
| CCOCN(c1c(C)cccc1CC)C(=O)Cn1cnc2c1cc(C)c(c2)C | 0 |
| O=C(Nc1ccc2c(c1)[nH]c(=O)[nH]2)CSc1nnc2n1c(C)cc(n2)C | 0 |
| Clc1ccc(cc1)CN1[C@H]2CCCN2c2c1cc(Cl)cc2 | 0 |
| O=C(Nc1ccc2c(c1)[nH]c(=O)[nH]2)COC(=O)CNC(=O)c1ccco1 | 0 |
| CC(=C1C=C(C=C1C)C(C)(C)C)C | 0 |
| COc1ccc2c(c1)ccc(c2)C=C | 0 |
| C#Cc1ccccc1C | 0 |
| CC1(C)[C@H]2CC[C@@]3(C1=CCCC3(C)C)C2 | 0 |
| CC(c1ccccc1F)C | 0 |
| COc1cc(Cl)ccc1F | 0 |
| F[C@@]1(Cl)CCC1(F)F | 0 |
| CC(=C)C(F)(F)F | 0 |
| Brc1ccc2c(c1)C=C[C@@H](O2)c1ccccc1 | 0 |
| CC1=CC(=C(C)C)C=C1 | 0 |
| N#Cc1cnn(c1N)CC(F)(F)F | 0 |
| C=CCC(F)(F)F | 0 |
| C#CCCc1ccccc1 | 0 |
| CCCCCC#CCC#C | 0 |
| CCCCC#CCC=C | 0 |
| CCCC#CC#CCCC | 0 |
| CCC#CCCC(C)C | 0 |
| CCC#CCC(C)C | 0 |
| C#CC(n1cccc1)(C)C | 0 |
| CCc1ccc(cc1)CNCCc1c[nH]c2c1cccc2 | 0 |
| FC1(F)COCC1(F)F | 0 |
| FCCC(F)(F)F | 0 |
| CCC1=C(C)CCC1 | 0 |
| Fc1ccc(c(c1F)F)NC1CC1 | 0 |
| C/C=C/C(F)(F)F | 0 |
| CC1=C[C@H]2[C@@H](C1)[C@@H]1C[C@H]2C(=C1)C | 0 |
| FC1CCCCC1 | 0 |
| CCCCc1ccc(cc1)NC | 0 |
| C1C[C@H]2[C@H](C1)C=CC=C2 | 0 |
| Cc1cc(F)cc(c1)F | 0 |
| N#Cc1c(cc([nH]c1=O)c1ccc(cc1)Br)C(F)(F)F | 0 |
| CCOc1cccc(c1)F | 0 |
| C[C@@]12CC[C@H]3[C@]1(C)CO[C@@H]2C3 | 0 |
| Cc1ccc2c(c1)N=C(c1ccccc1)c1c(O2)cccc1 | 0 |
| C#Cc1cccc(c1)C(F)(F)F | 0 |
| C#Cc1ccccc1C(F)(F)F | 0 |
| Clc1c(N)cc(cc1N)C(F)(F)F | 0 |
| Cc1ccc(cc1)c1ccc(cc1)I | 0 |
| Ic1ccc2c3c1cccc3CC2 | 0 |
| C[C@H](Cc1ccccc1)NCC#C | 0 |
| CC1=CCC=C(C1)C | 0 |
| CC1(C)C=CC(C1)(C)C | 0 |
| CC1=C(C)CC(C1)(C)C | 0 |
| C=CC(Cl)(C)C | 0 |
| COc1ccc(c(c1)C)C#C | 0 |
| CCC(=C)[C@@H](CC)C | 0 |
| Brc1ccc2c3c1c(Br)ccc3CC2 | 0 |
| C#Cc1ccc(cc1)C(C)(C)C | 0 |
| NC(=O)[C@H]1C[C@@H]2C[C@H]1C=C2 | 0 |
| FC(c1ccc(cc1)C)(F)F | 0 |
| C[C@H]1COCc2c1cc1c(c2)C([C@H](C1(C)C)C)(C)C | 0 |
| CC(Oc1cccc(c1)F)C | 0 |
| CC(Oc1ccc(cc1Cl)F)C | 0 |
| C[C@H]1C[C@@H]([C@@H]([C@H]1C)C)C | 0 |
| Fc1ccc2c(c1)C(F)(F)OC2(F)F | 0 |
| Fc1cc(C)c(c(c1)C)Cl | 0 |
| C=C1C[C@@H]1c1ccccc1 | 0 |
| N#CCn1cccn1 | 0 |
| N#Cc1cnn(c1)C | 0 |
| CCC[C@@]1(C)OC(=Cc2c1cc(OC)c(c2)OC)c1ccc(c(c1)OCC)OCC | 0 |
| C#Cc1ccc(cc1)F | 0 |
| CC(O[C@@H]1[C@@H]2CC[C@H]1CC2)(C)C | 0 |
| N#Cc1ncccn1 | 0 |
| Cc1cc(C)c2c(c1C)cc1c(c2)c2ccccc2cc1 | 0 |
| FC(Oc1ccccc1)(Cl)F | 0 |
| Cc1ccccc1CC1OCCO1 | 0 |
| Fc1ccc(cc1CC1OCCO1)F | 0 |
| COCc1cccc(c1)F | 0 |
| C1CCC2=C(C1)CC=CC2 | 0 |
| C=C1[C@H](C)CCC[C@@H]1C | 0 |
| C[C@@H]1CC(C[C@@H]1C)(C)C | 0 |
| COc1ccc(cc1I)C | 0 |
| COc1ccc(cc1Cl)C | 0 |
| Clc1ccc(cc1)c1nc(Cl)c2c(n1)ccc(c2)F | 0 |
| c1scc(c1)c1ccc2c(c1)[nH]nc2 | 0 |
| Brc1ccc(cc1)N1CCCCC1 | 0 |
| Cc1ccccc1C(F)(F)F | 0 |
| FCC(C(F)(F)F)(F)F | 0 |
| Cc1ccc2c(c1)c(Cl)c1c(c2Cl)cc(cc1)C | 0 |
| CCCC#CI | 0 |
| CC(C[C@@H](C(=O)[O-])NC(=O)[C@H]1CCCN1C(=O)[C@H](C(C)C)[NH3+])C | 0 |
| Cc1c(C)c[nH]c1c1nnc(n1N)c1[nH]cc(c1C)C | 0 |
| Clc1ccc(cc1)c1ccc(cc1)I | 0 |
| Clc1ccccc1OC(F)(F)F | 0 |
| C#Cc1ccccc1OC(F)(F)F | 0 |
| C#Cc1ccc(cc1)C(C)C | 0 |
| C#Cc1cccc(c1)Cl | 0 |
| COc1ccccc1C(F)(F)F | 0 |
| COc1ccccc1CC | 0 |
| CN1c2ccccc2N(C1(F)F)C | 0 |
| FCOc1cccc(c1)Br | 0 |
| Brc1ccc(cc1)S(=O)(=O)C[C@H]1CCc2c(C1=O)cccc2 | 0 |
| CC=C1CCCCCC1 | 0 |
| C1CCC(C1)[C@@H]1CCC=C1 | 0 |
| COc1ccccc1N[C@@H]1CC2=C(CC1(C)C)C(CCC2)(C)C | 0 |
| C#CCOc1c(F)c(F)nc(c1F)F | 0 |
| CCCN1CCCc2c1cccc2 | 0 |
| CC1=CCC2C[C@@H]1[C@@H](OC2(C)C)c1ccccc1 | 0 |
| C=CCOC(=O)[C@@H]1C(=NC2=CC(CC(=O)[C@@H]2[C@H]1c1cc(OC)c(cc1Br)OC)(C)C)C | 0 |
| O=C(c1cc2n(n1)[C@H](C[C@H](N2)c1ccc(cc1)Cl)C(F)(F)F)N1CCOCC1 | 0 |
| C1CC[C@@H]2[C@H](C1)C[C@H](OC2)c1ccccc1 | 0 |
| c1ccc(cn1)c1nc2ccc3c(c2c2c1CCC2)cccc3 | 0 |
| c1ccc(cc1)CC1=NCCn2c1ccc2 | 0 |
| CC1(C)[C@@H]2CC[C@@]1(C)[C@@H](C2)C1=CCCCC1 | 0 |
| CC#CCCC#CC | 0 |
| CCC#CC1CCCCC1 | 0 |
| CC#CC1CCCCC1 | 0 |
| CCCC#Cc1ccccc1 | 0 |
| Cc1cc2COCCc2cc1C | 0 |
| Cc1cccc2c1CCOC2 | 0 |
| CCOC1(Br)CC1 | 0 |
| Cc1ccc(cc1)CC1OCCO1 | 0 |
| Cc1ccc2c(c1c1c(C)ccc3c1cccc3)cccc2 | 0 |
| CN(c1ccccc1C(F)(F)F)C | 0 |
| ClOC(C)(C)C | 0 |
| Fc1cc(F)c(c(c1)F)I | 0 |
| CCn1c(C)cc2c1cccc2 | 0 |
| CC1(C)C[C@@H]1c1ccccc1 | 0 |
| Cc1cccc(c1C(F)(F)F)Br | 0 |
| Cc1cc(ccc1F)C1=CC(CC(C1)(C)C)(C)C | 0 |
| c1ccc2c(c1)cc1c(c2)[nH]c2c([nH]1)c1cccc3c1c2ccc3 | 0 |
| O=C1/C(=C/c2c3ccccc3cc3c2cccc3)/S[C@@H]2N1[C@@H]1CCCC[C@@H]1N2 | 0 |
| O=C(c1nn2c(c1)N[C@@H](C[C@H]2C(F)(F)F)c1ccc2c(c1)OCO2)Nc1cccnc1Cl | 0 |
| Fc1ccc(cc1)[C@H]1Nc2ccc(cc2[C@H](O1)c1ccccc1)Cl | 0 |
| O=C1c2ccccc2c2c1c(c1cccc3c1cccc3)n1c(n2)ncn1 | 0 |
| Brc1ccc(cc1)C1(Cc2ccccc2)C(=O)c2c(C1=O)cccc2 | 0 |
| C1CCC(CC1)c1cccc2c1Oc1c(C2)cccc1C1CCCCC1 | 0 |
| COc1ccc(cc1)c1nnc(c2c1cccc2)Nc1ccc(c(c1)Cl)Cl | 0 |
| CN1c2cc(ccc2C(=CC1(C)C)C)[C@@]1(C)CC(C)(C)N(c2c1cccc2)C | 0 |
| Cc1ccc2c(c1)ccc(n2)c1cnc2c(c1)cc(cc2)C | 0 |
| Clc1ccc(cc1)[C@@H]1Nc2cc(nn2[C@@H](C1)C(F)(F)F)C(=O)Nc1noc(c1)C | 0 |
| Nc1ccc2c(c1)N(C(=O)c1ccc(cc1)C)C(C[C@]2(C)c1ccccc1)(C)C | 0 |
| c1ccc(cc1)[C@@H]1C=C(c2ccccc2)n2c3[C@@H]1CCCc3c1c2cccc1 | 0 |
| Clc1ccc(cc1)CN1c2ccccc2C(=CC1(C)C)C | 0 |
| C1CC=C2[C@@H](C1)[C@@H](OC1=C2CCCC1)c1ccccc1 | 0 |
| C[C@H]1CCOC2(C1)CCCCC2 | 0 |
| Cc1cccc(c1C)n1c2cccc(c2cc2c1nc(=O)n(c2=O)C)Cl | 0 |
| COc1ccccc1C#Cc1ccccc1 | 0 |
| C1CCC2(CC1)Cc1ccccc1c1c2c2nc(nn2cn1)c1ccncc1 | 0 |
| C=CCc1cccc(c1)C | 0 |
| C#Cc1cccc(c1)C | 0 |
| Cl[C@]1(Br)[C@H]2[C@@H]1CC[C@@H]1[C@@H](CC2)[C@@]1(Cl)Br | 0 |
| C[C@H](CN(c1ccc(cc1)C)C[C@H](O)C)O | 0 |
| Clc1cc2cc(cc(c2cc1C)Cl)C(F)(F)F | 0 |
| COc1ccc(cc1)C(F)(F)F | 0 |
| C#Cc1cccc(c1)OC(F)F | 0 |
| F[C@H]1[C@H](F)C(C1(F)F)(F)F | 0 |
| CCC(CC)(Cl)C | 0 |
| COc1ccc(cc1COc1cc(C)ccc1C)[C@H]1NC(=O)c2c(N1)n(nc2)c1ccccc1 | 0 |
| Fc1ccc(cc1)OCCCC[NH+]1CCN(CC1)c1cccc(c1)C(F)(F)F | 0 |
| Brc1ccc2c(c1)C[C@@H]1c3c([C@H](C2)c2c1cccc2)cccc3 | 0 |
| c1ccc(cc1)Oc1c2ccccc2cc2c1cc1CCc3c1c2ccc3 | 0 |
| COc1ccccc1[C@H]1CCCC=C1 | 0 |
| Cc1cc(c(c(c1C)C)C)C(C)(C)C | 0 |
| C[C@@H]1CC/C(=C/c2ccccc2)/CC1 | 0 |
| CN1[C@@H](C=Cc2c1cccc2)c1ccccc1 | 0 |
| Cc1cc2[nH]c3c([nH]c2cc1C)c1c2c3cccc2ccc1 | 0 |
| CCOc1cc(C)cc(c1)C | 0 |
| Cc1ccc(cc1)N1CC=CC1 | 0 |
| CN1[C@@H](C)Cc2c1cccc2 | 0 |
| CN1c2ccc(cc2C=CC1(C)C)c1ccccc1 | 0 |
| CC(=C1CC[C@@H](C2=C(C1)[C@@H](C)CC2)C)C | 0 |
| Cc1cc(cc(c1)C)c1cc(C)cc(c1)C | 0 |
| Clc1cc(Cl)c(c(c1Cl)I)Cl | 0 |
| C#CCOc1ccccc1 | 0 |
| C#CCC1CCCC1 | 0 |
| O=C1OCCCO1 | 0 |
| CCc1ccccc1NC(=O)Nc1ccccc1C(F)(F)F | 0 |
| O=C(Nc1ccccc1C(F)(F)F)Nc1c(C)cc(cc1C)C | 0 |
| CCCCc1ccc(cc1)NC(=O)Nc1ccc(cc1C)C | 0 |
| C=Cc1ccc2c3c1cccc3c1c3c2cccc3ccc1 | 0 |
| c1ccc2c(c1)c1c(cc2)CCc2c1c1ccccc1cc2 | 0 |
| C#Cc1ccc(cc1)C#C | 0 |
| Cc1cc(C)c2c(c1C)OC(CC2)(C)C | 0 |
| CCc1cccc2c1ccc(c2)OC | 0 |
| Cc1ccc2c(c1)C=C1[C@@H](C2)c2ccc(cc2C=C1)C | 0 |
| C1=CCC2=C(C1)CC1=C(C2)CC=CC1 | 0 |
| CC(C1OC(C)(C)C2C[C@@H]1C(=CC2)C)C | 0 |
| CC1=C[C@H](C)C2[C@@H](C1C1(CCCC1)OC2)C | 0 |
| OCCCN1CC(=O)N2[C@H](C1=O)Cc1c([C@@H]2c2cccc(c2)Cl)[nH]c2c1cccc2 | 0 |
| C1CCCCCC[C@@H]2[C@@H](CCC1)COC2 | 0 |
| [O-]C(=O)[C@@H]1Cc2c3ccccc3[nH]c2[C@@H]([NH2+]1)c1cccc(c1)C(F)(F)F | 0 |
| C/C=C/n1c2ccccc2c2c1cccc2 | 0 |
| C[C@H]1O[C@H](CC(=C)C1)c1ccccc1 | 0 |
| C1CC[C@@H]2[C@H](C1)C1=CCCC[C@@H]1C1(O2)CCCC1 | 0 |
| Cc1ccc(cc1)c1nc2ccccc2c(c1)C(=O)Nc1ccc(cc1F)Br | 0 |
| CCOc1ccc(cc1)[C@@]1(C)CC(C)(C)Nc2c1cc(C)cc2 | 0 |
| O=C(c1ccc(c(c1)Br)C)OCc1ccc(c(c1)Cl)Cl | 0 |
| c1ccc(cc1)n1c2ccccc2c2c1c1ccccc1CCC2 | 0 |
| c1ccc(cc1)C#C/C=C/c1ccc2c(c1)c(ccc2)c1ccccc1 | 0 |
| COc1ccc(cc1)c1cccc2c1c1c(cc2)ccc2c1cc(C)cc2 | 0 |
| Cc1ccc2c(c1/C=C\C#Cc1ccccc1)cccc2 | 0 |
| Clc1c(Cl)c(Cl)c(c2c1CC1=C2CCC1(C)C)Cl | 0 |
| CC1(C)C2=C[C@@]3(C)N(CCN2c2c1cccc2)c1c(C3(C)C)cccc1 | 0 |
| CCN1c2ccccc2[C@@](CC1(C)C)(C)c1ccccc1 | 0 |
| CCOC(=O)C1=C(N)n2c(=C([C@@H]1c1cccc(c1)OC)C(=O)N)s/c(=C/c1cccc(c1)OC)/c2=O | 0 |
| Cn1c2CC[C@H](Cc2c2c1cccc2)C1CCCCC1 | 0 |
| Cc1cc2[nH]c(nc2cc1C)[C@H](O)C | 0 |
| Nc1ccc2c(c1)[C@@H](O)c1c2ccc(c1)Cl | 0 |
| C[C@@H]1c2ccccc2c2c(N1C)cccc2 | 0 |
| CN1c2ccccc2C[C@@H]1c1cn(c2c1cccc2)C | 0 |
| OC[C@H](COc1ccc(cc1Cl)Cl)O | 0 |
| OC[C@H](COc1cccc(c1)C(F)(F)F)O | 0 |
| C1=Cc2c(OC1)cccc2 | 0 |
| C1C[C@@H]2CC[C@H]1CC2 | 0 |
| O1Cc2c(C1)cccc2 | 0 |
| COC1CCCCC1 | 0 |
| C1C[C@@H]2CC[C@H]1C=C2 | 0 |
| FC(C(F)(F)C)(F)F | 0 |
| CCCC#CCCC | 0 |
| CC1=Cc2c(C1)cccc2 | 0 |
| C[C@@H]1CCCCC1(C)C | 0 |
| C[C@H]1CCC(C1)(C)C | 0 |
| CC[C@@H]1CCCC[C@@H]1C | 0 |
| C[C@@H]1C[C@@H](c2c1cccc2)c1ccccc1 | 0 |
| CN1CC[C@H](c2c1cccc2)c1ccccc1 | 0 |
| C1CC[C@@H](OC1)c1ccccc1 | 0 |
| COc1ccc(cc1)[C@@]1(C=Cc2c(O1)ccc1c2ccc(c1)Br)c1ccccc1F | 0 |
| CN(c1ccc2c(c1)c1ccccc1n2C)C | 0 |
| CC1(C)CCC(O1)(C)C | 0 |
| C[C@@H]1CCO[C@@H](C1)C=C(C)C | 0 |
| C=Cc1ccc(cc1)C=C | 0 |
| CCc1ccc(cc1)CC | 0 |
| CCc1ccccc1CC | 0 |
| C=C[C@H]1CC[C@H](CO1)C=C | 0 |
| CCC(Cl)(Cl)Cl | 0 |
| Clc1cc2Oc3ccccc3Oc2cc1Cl | 0 |
| C1CC=CC=CC=C1 | 0 |
| CC(OC(Cl)(C)C)(Cl)C | 0 |
| Cc1ncoc1C#N | 0 |
| CO[C@H]1CCc2c1cccc2 | 0 |
| CC(n1c2ccccc2c2c1cccc2)C | 0 |
| CCCN1c2ccccc2C(=O)C1=O | 0 |
| c1ccc2c(c1)n(c1c2cccc1)n1c2ccccc2c2c1cccc2 | 0 |
| C=Cc1ccccc1OC | 0 |
| C=C[C@]1(C)CC[C@@H](O1)C(=C)C | 0 |
| C=Cc1cccc(c1)F | 0 |
| Fc1ccc(cc1)OC(F)(F)F | 0 |
| COC(C(F)F)(F)F | 0 |
| COc1cc(ccc1OC)/C=C/1\NN2[C@@H](S1)NC(=O)/C(=C/c1ccc(cc1)O)/C2=N | 0 |
| CCC#Cc1ccccc1 | 0 |
| CCOC(F)(F)F | 0 |
| FC(C#CC(F)(F)F)(F)F | 0 |
| CC(c1ccccc1)(Cl)C | 0 |
| COC(c1ccccc1)(C)C | 0 |
| CN(c1ccc2c(c1)cccc2)C | 0 |
| CC(Oc1ccccc1)C | 0 |
| COC1=CCC=CC1 | 0 |
| Brc1cccc(c1)n1cc2c(c1)CCc1c(CC2)cn(c1)c1cccc(c1)Br | 0 |
| C/C=C(/c1ccccc1)\C | 0 |
| C1CCCCCC2=C(CCCC1)OCCC2 | 0 |
| COc1cc(C)cc2c1C(C)(C)CCC2(C)C | 0 |
| Brc1ccc2c(c1)C(C)(C)CCC2(C)C | 0 |
| Cn1ccc2c1cccc2 | 0 |
| C1CCc2c(C1)nc1c(n2)c2ccccc2c2c1cccc2 | 0 |
| C1C=Cc2c(OC1)cccc2 | 0 |
| Nc1nc(C)nc(c1)c1ccccn1 | 0 |
| c1cc2[C@@H]3[C@H](c4c2c(c1)ccc4)[C@H]1[C@@H]3c2cccc3c2c1ccc3 | 0 |
| Clc1c(C)c(Cl)c(c(c1Cl)Cl)Cl | 0 |
| COc1ccncn1 | 0 |
| Cn1cccnc1=O | 0 |
| CC1=C(C)[C@@]2([C@@]1(C)[C@@]1([C@@]2(C)C(=C1C)C)C)C | 0 |
| CCC#CC#CCC | 0 |
| Cc1ccc(c2c1c1c2c(C)ccc1C)C | 0 |
| C1CC[N+](=C(Cc2ccccc2)Cc2ccccc2)C1 | 0 |
| FCCc1ccc(cc1)C | 0 |
| FC(Cc1ccccc1)(F)F | 0 |
| FC(Cc1ccccc1)F | 0 |
| COc1cc(C)ccc1C1=CCCC1 | 0 |
| CO[C@H]1c2ccccc2CC1(C)C | 0 |
| C1CCC2(CC1)CCCO2 | 0 |
| CN(c1ccc(cc1)C=C1c2ccccc2c2c1cccc2)C | 0 |
| Cn1c2ccccc2c2c1cc1CCCCc1c2 | 0 |
| CCOc1cc2c(c3c1cccc3)c(C)c1c(c2C)cccc1 | 0 |
| O[C@@H](c1ccccc1)C[C@H](c1c(C)[nH]c2c1cccc2)C | 0 |
| CC(=C1C=CC=C1)C | 0 |
| ClC1(Cl)[C@@H]2[C@H]1CCCO2 | 0 |
| Cn1c(C)c(c(c1C)c1ccccc1)c1ccccc1 | 0 |
| CCCC1c2c(ccc3c2cccc3)Oc2c1c1ccccc1cc2 | 0 |
| c1ccc(cc1)[C@@H]1Oc2c([C@@H]1c1ccccc1)c1ccccc1c1c2cccc1 | 0 |
| CC(=C)CCC(=C)C | 0 |
| COc1c2cccc(c2cc2c1cccc2Cl)Cl | 0 |
| CC1=CCCC[C@H]1Oc1ccccc1 | 0 |
| CO[C@@H]1C=CC=CC=C1 | 0 |
| CO[C@H]1C(C1(C)C)(Br)Br | 0 |
| Nc1ccc(c(c1)O)Nc1nc2c(s1)cccc2 | 0 |
| O=c1[nH]c(=O)ncn1C1CCCCC1 | 0 |
| CC[C@H](Nc1ccccc1)C | 0 |
| CCN(C#CC)CC | 0 |
| CC[NH+](CCCN(c1ccnc2c1ccc(c2)Cl)c1ccccc1)CC | 0 |
| c1ccc(cc1)C1=CCCC1 | 0 |
| Cc1cc(C)c(c(c1)C)F | 0 |
| Fc1cc(F)c(c(c1)C(F)(F)F)F | 0 |
| Nc1ncnc2c1CN(C2)c1ccccc1 | 0 |
| c1ccc(cc1)C1(C=Cc2c(O1)cccc2)c1ccccc1 | 0 |
| Oc1cccc(c1)c1nnc(n1N)c1cccc(c1)O | 0 |
| C1=CC2C[C@@H](C1)CC2 | 0 |
| Cc1cc(C)cc(c1)C | 0 |
| CCc1cc(C)ccc1C | 0 |
| CC/C=C/C(C)(C)C | 0 |
| CC1(C)CCCCC1 | 0 |
| C=CCC1CCCC1 | 0 |
| C=CCc1ccccc1C | 0 |
| C=CCc1ccc(cc1)C | 0 |
| CC[C@@H]1CC[C@@H](C1)C | 0 |
| CC(C1CCCC1)C | 0 |
| C[C@H]1C[C@@H]([C@@H](C1)C)C | 0 |
| C=CC1CCCCC1 | 0 |
| Clc1ccc(cc1)n1ncc2c1c1ccccc1CCC2 | 0 |
| Cc1ccc(cc1)C(C)(C)C | 0 |
| COc1cccc(c1)C | 0 |
| CCOc1ccccc1Cl | 0 |
| CCOc1ccc2c(c1C)cccc2 | 0 |
| C=CCCc1ccccc1 | 0 |
| Brc1ccc2c(c1)CCC2 | 0 |
| CC1(Cl)CCCCC1 | 0 |
| CC(C(C)(C)C)(C)C | 0 |
| CCc1cccc(c1)Cl | 0 |
| COc1ccc2c(c1)Cc1c2cccc1 | 0 |
| Cc1cccc(c1)c1cccc(c1)C | 0 |
| Clc1cccc(c1)C(Cl)(Cl)Cl | 0 |
| Cn1c2ccccc2cc2c1cc1c2cccc1 | 0 |
| CN(c1ccc(cc1)c1ccccc1)C | 0 |
| Brc1ccc2c(c1)C(=O)C(=N2)c1c[nH]c2c1cc(Br)cc2 | 0 |
| OCc1cc(=O)[nH]c(=O)[nH]1 | 0 |
| ClC1(Cl)[C@@H]2[C@H]1CCCC2 | 0 |
| Cc1ccc(n1N(c1ccccc1)C)C | 0 |
| Cc1ccc(n1n1c2ccccc2c2c1cccc2)C | 0 |
| Clc1ccc(cc1)C1=NCCO1 | 0 |
| C1CCN(C1)c1ccccc1 | 0 |
| COC([C@@H]1CCC(=CC1)C)(C)C | 0 |
| O1C[C@@H]2[C@@H](C1)CC=CC2 | 0 |
| COc1c(Cl)c(Cl)c2c(c1Cl)cc(cn2)Cl | 0 |
| C/C=C/C[C@H](C=C)C | 0 |
| C=Cc1c(C)cc(cc1C)C | 0 |
| CCC1=CCC=CC1 | 0 |
| Cc1c2ccccc2cc2c1ccc1c2c(F)ccc1 | 0 |
| CCOc1cccc2c1cccc2 | 0 |
| CCN(c1ccccc1)C | 0 |
| C1CCC(C1)C1CCCC1 | 0 |
| C1CC1c1ccccc1 | 0 |
| O=C([C@H]1C(=O)N[C@@]2(CC1c1ccccc1O2)C)Nc1ccc(cc1)F | 0 |
| C1CCN(CC1)c1cccc2c1cccc2 | 0 |
| COc1cc(ccc1C)C(C)(C)C | 0 |
| CN(c1c(C)cccc1C)C | 0 |
| CC(c1ccccc1)C | 0 |
| CC(=C)CC(C)(C)C | 0 |
| COC(=O)C1Cc2c(C1)cc1c(c2)CCC1 | 0 |
| COC(=O)c1ccc2c(c1)C[C@@]1(C2)Cc2c(C1)c1CCCCc1c(c2)CC | 0 |
| c1ccc2c(c1)nc1c(n2)[nH]c2c([nH]1)cccc2 | 0 |
| Nc1ccc2c(c1)c(=O)n1c(n2)CCCCC1 | 0 |
| C1=C[C@H](CC1)O[C@H]1CCC=C1 | 0 |
| Clc1ccc(cc1Cl)C(Cl)(Cl)Cl | 0 |
| Cc1c(Br)c(C)c(c(c1C)C(Cl)(Cl)Cl)C | 0 |
| C1COC(C1)(C1CC1)C1CC1 | 0 |
| CC(=Cc1ccccc1)C | 0 |
| COc1ccc2c(c1)c1c(cc2)ccc2c1cccc2 | 0 |
| COC(=O)[C@]12CC[C@H](C1(C)C)CC2=O | 0 |
| CC1(C)CCCOc2c1cccc2 | 0 |
| c1cc2Oc3cccc4c3c3c2c(c1)ccc3cc4 | 0 |
| OC[C@@H](COc1ccc(cc1)Br)O | 0 |
| CC(=C)c1ccc(cc1)F | 0 |
| Cc1cc2c(cc1C)CN1CN2Cc2c1ccc(c2C)C | 0 |
| Brc1cc(Br)cc2c1oc(=O)[nH]2 | 0 |
| CC(C(C)C)C(C)C | 0 |
| Nc1cccc(c1)C(=O)c1ccc(c(c1)N)N | 0 |
| CCCn1ccc2c1cccc2 | 0 |
| CCn1ccc2c1cccc2 | 0 |
| Cn1ccc(=O)nc1 | 0 |
| COc1ccc2c(c1)ccc(c2)I | 0 |
| c1ccc(cc1)C1=NC(N=C1c1ccccc1)(c1ccccc1)c1ccccc1 | 0 |
| O1Cc2cccc3c2c2c(C1)cccc2CC3 | 0 |
| O1Cc2cccc3c2c2c(C1)cccc2cc3 | 0 |
| Clc1ccc(cc1)C#Cc1ccccc1 | 0 |
| C=C1CC[C@@]2([C@H]1C2)C(C)C | 0 |
| CCOc1ccccc1 | 0 |
| Cc1ccc(cc1)c1cc2n(c1)cccc2 | 0 |
| CCCC(=O)Oc1c(Br)cc(cc1Br)Br | 0 |
| Cc1ccc(cc1)Cl | 0 |
| Brc1ccc(c(c1)Br)Nc1ccc(cc1Br)Br | 0 |
| Clc1ccc2c(c1)nc(nc2N)N(C)C | 0 |
| Nc1ccc(c2c1C(=O)c1ccccc1[C@@H]2O)O | 0 |
| CC1(C)c2ccccc2N2C1=C[C@@]1(C)N(C2)c2c(C1(C)C)cccc2 | 0 |
| CCCC[NH+](CC(=O)c1ccc2c(c1)c1ccccc1c(c2)Br)CCCC | 0 |
| COc1ccc(cc1)C(C)(C)C | 0 |
| Clc1ccc2c(c1)CN(C)c1c(CN2C)cc(cc1)Cl | 0 |
| CC(Br)(C)C | 0 |
| CCC(Cl)(C)C | 0 |
| CCC(Br)(C)C | 0 |
| C[C@H]1CC(=O)Nc2c(N1)ccc(c2)C(=O)C | 0 |
| CCCC1Nc2cccc3c2c(N1)ccc3 | 0 |
| COc1cc(OCc2ccc(cc2)OCc2ccccc2)cc(c1OC)OC | 0 |
| C=C1C[C@@H]2C[C@H]1CC2 | 0 |
| COc1ccccc1c1ccccc1 | 0 |
| Cc1ccc(cc1)N(C)C | 0 |
| COc1ccc(cc1)C(=C)C | 0 |
| COc1ccc2c(c1)CCC2 | 0 |
| c1ccc2c(cc1)ccc2 | 0 |
| CC(=O)N1CCCC2=C1CCCCCCCCCC2 | 0 |
| OCCN(c1cc(C)c(cc1C)C)CCO | 0 |
| Brc1ccc(cc1)N(c1ccc(cc1)Br)c1ccc(cc1)Br | 0 |
| C#CCOc1cc(C)cc(c1)C | 0 |
| CC([C@@H]1CCC(=C)C=C1)C | 0 |
| c1ccc(cc1)n1cccc1 | 0 |
| CCc1ccc(cc1)n1cccc1 | 0 |
| Clc1ccc2c(c1)cc([nH]2)c1ccc(cc1)c1ccccc1 | 0 |
| O=C(Nc1cccc(c1)C(F)(F)F)Nc1cccc(c1)C(F)(F)F | 0 |
| Clc1cccc2c1C(F)(F)c1c(C2(F)F)cccc1 | 0 |
| O=c1cc([nH]c(=O)n1C)N1CCCC1 | 0 |
| C[C@H]1CCC[C@@H](N1c1cc(=O)n(c(=O)[nH]1)C)C | 0 |
| Clc1cccc(c1N1CCCCC1)Cl | 0 |
| FC1(F)[C@@H]2C[C@@H]3C[C@H]1C[C@H](C2)C3 | 0 |
| Ic1ccc2c(c1)Cc1c2cccc1 | 0 |
| COc1ccc(cc1)N[C@@H](c1c(C)[nH]c2c1cccc2)c1ccccn1 | 0 |
| Clc1ccc2c(c1)c(C)c(o2)c1cc(=O)oc2c1cc1OCOc1c2 | 0 |
| COC(CNc1ncnc2c1c(cn2c1cccc(c1)C(F)(F)F)c1ccccc1)OC | 0 |
| O=C(Nc1cc2OCCOc2cc1[C@@H](c1ccccc1)O)Nc1cccc(c1)Cl | 0 |
| FC(Oc1ccc2c(c1)cccc2)F | 0 |
| Nc1ccccc1NC1CCCCC1 | 0 |
| OCc1ncccc1C | 0 |
| C=Cc1ccccc1C | 0 |
| Fc1cc(N)c(cc1N1CCOCC1)N | 0 |
| COc1cc(OC)c2c(c1)n(Cc1ccccc1)cc2[C@@H]1NC(=Nc2n1c1ccccc1n2)N | 0 |
| COc1cc(CN2C(=O)CC[C@]2(C)C(=O)c2ccc(cc2)Br)cc(c1OC)OC | 0 |
| Fc1ccc(cc1)c1ccc(n1c1ccc(cc1)Cl)C | 0 |
| Clc1ccc(c(c1)S(=O)(=O)/N=C\1/SC[C@@H](N1)c1sccc1Cl)Cl | 0 |
| Brc1ccsc1c1ccn[nH]1 | 0 |
| C[C@@H]1CCc2c(C1)sc1c2c(SCC(=O)c2c[nH]c3c2cccc3)nc(n1)C | 0 |
| Cc1cccc2c1nc(cc2)c1ccc2c(n1)c(C)ccc2 | 0 |
| O=C(C1=C(C)Nc2n([C@H]1c1c(F)cccc1Cl)ncn2)OC1CCCCC1 | 0 |
| CC(O[C@H]1C[C@]21CCC12CC1)(C)C | 0 |
| C1[C@@H]2C[C@@H]3C[C@H]1C[C@H](C2)C3 | 0 |
| C#CC(Cl)(C)C | 0 |
| Cc1ccc(c(c1)C)N1COc2c(C1)c1ccccc1cc2 | 0 |
| O1CC2(C1)CC1(C2)c2ccccc2c2c1cccc2 | 0 |
| Clc1cc2CC(=Cn3c2c(c1)c1cc(Cl)ccc31)C | 0 |
| CC([C@H]1O[C@H]2CCCC[C@H]2C2=CCCC[C@@H]12)C | 0 |
| C1CCC(CC1)c1ccc2c(c1)c1CCCc3c1n2ccn3 | 0 |
| OC[C@]12CC=C([C@H](C1)[C@@H](OC2)C(CO)(C)C)C | 0 |
| CCc1nc(SCC(=O)c2c[nH]c3c2cccc3)c2c(n1)sc1c2CCCC1 | 0 |
| O=c1oc2c(cc1c1oc3c(n1)cccc3)cc1c3c2CCCN3CCC1 | 0 |
| Fc1ccc(cc1)CC1(c2ccc(cc2)Cl)C(=O)c2c(C1=O)cccc2 | 0 |
| Ic1ccc(cc1)n1cnc2c(c1=O)c1CCCCc1s2 | 0 |
| O=C(Nc1ccccc1C)CSc1nc2c(n1C)c(=O)n(c(=O)n2C)Cc1ccccc1Cl | 0 |
| CCOc1c(Cl)cc(cc1Cl)[C@@H]1NC(=O)NC(=C1C(=O)Nc1ccc(cc1)F)C | 0 |
| O=C1NC2C[C@](N1)(C)Oc1c2cccc1 | 0 |
| CC[C@@H]1CCCCO1 | 0 |
| CC[C@H]([C@@H]1CCCO1)C | 0 |
| C[C@@H]1CCc2c(C1)sc1c2c(=O)n(c(n1)C)c1ccc(cc1)Br | 0 |
| O=C(Nc1cccc(c1Cl)Cl)Nc1cccc(c1)C(F)(F)F | 0 |
| c1ccc2c3c(c2c1)cccc3 | 0 |
| Clc1ccc(cc1)N1COc2c(C1)c1ccccc1cc2 | 0 |
| O[C@@H]1NC(=O)N[C@H]1O | 0 |
| CC(=C)[C@]12CCC[C@]3([C@@H]1CC[C@@]3(C2)C)C | 0 |
| CC1(C)c2ccccc2Oc2c1cccc2 | 0 |
| O=C1N[C@@H](c2ccccc2)C2=C(N1C)CN(C2=O)c1ccc(cc1)OC(Cl)(F)F | 0 |
| COc1ccc(cc1)c1ccccc1 | 0 |
| N#Cc1c(SCC(=O)c2c(C)[nH]c3c2cccc3)nc2c(c1CC)C(=O)CC(C2)(C)C | 0 |
| CCN(c1ccc(cc1)[C@H]1N(CCN1C(=O)c1ccc(cc1)C)C(=O)c1ccccc1)CC | 0 |
| COc1ccc(cc1)N(S(=O)(=O)c1ccccc1)CC(=O)NC1=C(C)[N+](=[N+](C1=O)c1ccccc1)[CH2-] | 0 |
| Ic1ccc(c(c1)C)NC(=O)c1ccc(cc1)NS(=O)(=O)C | 0 |
| Clc1ccc(cc1)CS(=O)(=O)Nc1ccc(cc1)I | 0 |
| Cc1ccccc1C1(C)CCOCC1 | 0 |
| Ic1ccc(cc1)c1noc(n1)c1cccs1 | 0 |
| O=S1(=O)N=C2N(c3c1cccc3)Cc1c(C32CCOCC3)cccc1 | 0 |
| N#Cc1c2CCCCCCCCCCc2nc(c1C#N)I | 0 |
| Cc1c(C)c(C)c(c(c1C)I)C | 0 |
| Cc1c(cc2c(c1n1cccc1)C(C)(C)CC2(C)C)N1CCCC1 | 0 |
| COc1ccc(c2c1CCC2)C | 0 |
| CC[C@@]12C[C@H]3C[C@@H](C1)C[C@@](C2)(C3)Br | 0 |
| C1[C@@H]2C[C@@H]3[C@@H]([C@H]1C[C@H](C2)C3)c1ccccc1 | 0 |
| Cc1ccc(cc1)c1ccc(cc1)Br | 0 |
| I[C@@]12CCC[C@@](C1)(CC2)c1ccccc1 | 0 |
| COc1c(C)c(C)cc(c1C)C | 0 |
| Cc1cc(C)cc(c1)N1Cc2c(C1)cccc2 | 0 |
| CC1(C)c2ccccc2c2c1cccc2 | 0 |
| Cc1ccc2c(c1)[nH]c(n2)c1ccccc1O | 0 |
| Brc1cc(Br)c2c(c1)c(=O)oc(n2)c1cccs1 | 0 |
| O=C(Nn1c(C)nc2c(c1=O)cnn2c1ccccc1)Nc1ccc(c(c1)Cl)Cl | 0 |
| OCn1ncc(c(c1=O)Cl)Cl | 0 |
| CN1c2ccccc2C[C@H]1C(C)(C)C | 0 |
| Cc1ccccc1N1Cc2c(C1)cccc2 | 0 |
| c1ccc(cc1)[C@@H]1Oc2ccccc2[C@@H](C1)c1ccccc1 | 0 |
| Cc1cc2CCCn3c2c(c1)c(c1ccccc1)c3C | 0 |
| CC=C=C1CCC(CC1)c1ccccc1 | 0 |
| c1ccc(cc1)c1cc2n3c1ccccc3ccc2 | 0 |
| COc1ccccc1[C@@H]1Nc2c(Cl)ccc(c2[C@H]2[C@@H]1CC=C2)C(F)(F)F | 0 |
| c1ccc(cc1)[C@@H]1CCc2c(O1)cccc2 | 0 |
| CC1=C2C(=C(C1)C)C(C[C@@]12CC(C2=C(CC(=C12)C)C)(C)C)(C)C | 0 |
| C1CCC2(CC1)C=Cc1c(O2)cc2c(c1)cccc2 | 0 |
| Cc1c(Cc2c(C)c(c3n2cccc3)C)n2c(c1C)cccc2 | 0 |
| Cn1c2ccccc2c(c1c1ccccc1)C | 0 |
| Cc1ccc(n1c1cccc2c1cccc2n1c(C)ccc1C)C | 0 |
| Cc1ccc(n1c1cccc2c1cccc2)C | 0 |
| CCc1ccc(cc1)c1cn2c(c1C)c1ccc(n1cc2)C | 0 |
| CC1(C)CO[C@@H](c2c1cccc2)c1ccccc1 | 0 |
| Cc1ccc2c(c1)N(Cc1ccccc1)C(C=C2C)(C)C | 0 |
| Cc1cc2c(cc1Br)Cc1c2cccc1 | 0 |
| Cc1ccc2c(c1)C=Cc1c(O2)cccc1 | 0 |
| CC(c1ccc(c2c(c1)c(C)cc2)C)C | 0 |
| CCc1ccc(c2c(c1)c(C)cc2)C | 0 |
| CC1=CC[C@H](CC1)C(=C)C | 0 |
| CC1=CCC(=CC1)C(C)C | 0 |
| CC1=CC=C(CC1)C(C)C | 0 |
| C=C1CC[C@@H]2C[C@H]1C2(C)C | 0 |
| CC1=CC[C@H]2[C@@H](C1)C2(C)C | 0 |
| O=C1NC(=C([C@H](N1)c1ccc(cc1Cl)Cl)C(=O)Nc1ccccn1)c1ccccc1 | 0 |
| Br[C@]12C[C@@H]3C[C@](C1)(C[C@](C2)(C3)Br)Br | 0 |
| N#Cc1c2CCCCc2c(n2c1nc1c2cccc1)n1cnc2c1cccc2 | 0 |
| O=C(N1C[C@@]2(CC1CC(C2)(C)C)C)c1nn2c(c1)N[C@@H](C[C@H]2C(F)(F)F)c1ccco1 | 0 |
| CCC(=O)[C@@H](CC)O | 0 |
| [O-]C(=O)[C@H]1C[C@H](C[NH2+]1)O | 0 |
| O=c1ccc2c(o1)c(O)c1c(c2)cco1 | 0 |
| COc1ccc(cc1[C@@H]1Nc2ccc(cc2[C@H]2[C@@H]1CC=C2)F)Br | 0 |
| Cc1ccc(c(c1)NC(=O)c1nn2c(c1Br)N[C@H](C[C@H]2C(F)(F)F)c1ccco1)O | 0 |
| Brc1ccc(cc1)S(=O)(=O)c1cccc(c1)S(=O)(=O)/N=C/1\CCCCN1 | 0 |
| Clc1ccc(cc1)c1cc2c(n1C)cccc2 | 0 |
| COc1cccc2c1N[C@H](c1ccc(cc1)Br)[C@@H]1[C@H]2C=CC1 | 0 |
| Nc1c(ncnc1Cl)N1CCCC1 | 0 |
| O=c1oc2ccccc2cc1C1=NC2(CCCCC2)Cc2c1cc1OCOc1c2 | 0 |
| COc1ccc(cc1)NC(=O)N1CCC(CC1)n1c(=O)[nH]c2c1cccc2 | 0 |
| Ic1cccc(c1)c1[nH]c(c(n1)c1ccco1)c1ccco1 | 0 |
| Clc1ccc(cc1)[C@H]1Nc2cc(nn2[C@H](C1)C(F)(F)F)C(=O)Nc1cccc(c1)O | 0 |
| Brc1ccc(c(c1)/C=C\1/S[C@H]2N(C1=O)[C@H]1[C@@H](N2)CCCC1)F | 0 |
| COC(=O)c1sc(c(c1Br)Br)Br | 0 |
| CCc1ccc(c(c1)OC(=O)c1ccccc1I)C | 0 |
| Ic1ccc(cc1)NS(=O)(=O)c1ccc(cc1)C(C)(C)C | 0 |
| N#Cc1c(SCc2ccccc2F)nc2c(c1C(F)(F)F)C[C@H](CC2)C | 0 |
| Cc1ccc(cc1)c1cc(C(F)F)n2c(n1)c(cn2)C(=O)Nc1ccc(cc1)N(C)C | 0 |
| O=C(C1CCCCC1)Oc1ccc(cc1Br)Br | 0 |
| Cc1c(Br)c(C)c(c(c1Br)C)Br | 0 |
| Fc1ccc(cc1)N1Cc2c(C1)cc(c(c2)C)C | 0 |
| Fc1c2Oc3ccccc3Oc2c(c(c1F)C(F)(F)F)F | 0 |
| OCCn1nc(c2c1CCCC2=O)C | 0 |
| O=c1oc2cc3occ(c3cc2c2c1CCC2)c1ccc2c(c1)OCO2 | 0 |
| Oc1ccc(cc1)c1[nH]nc(n1)c1cccc(c1)O | 0 |
| Clc1ccc(cc1)Cn1c2ccccc2c2c1CCCC2 | 0 |
| F[C@]1(Br)[C@@H]2[C@H]1C1([C@@H]3[C@H]2[C@H]2C=C[C@@H]3C32CC3)CC1 | 0 |
| BrC1(Br)[C@@H]2[C@]1(CCCCC2)c1ccccc1 | 0 |
| C1CCC2=C(C1)C[C@H]1[C@]3(N2c2ccccc2O3)CCCC1 | 0 |

**Table S2.** Structures of the 664 compounds (in SMILE format) used for the test set together with their activities (0 or 1).

| **mol** | **Activity** |
| --- | --- |
| O[C@@H](c1cc(O)c(O)cc1)C[NH3+] | 1 |
| [N@H+]1([C@@H](CCC1)c1cccnc1)C | 1 |
| [O-]C(=O)c1cccnc1 | 1 |
| O=C(N)c1cccnc1 | 1 |
| c12c3c4c(c1ccc1c2cccc1)cccc4ccc3 | 1 |
| c12c3c(ccc2ccc2c1cccc2)cccc3 | 1 |
| O(c1cc2c(CCNC(=O)C)c[nH]c2cc1)C | 1 |
| Ic1cc(C[C@H]([NH3+])C(=O)[O-])cc(I)c1Oc1cc(I)c(O)cc1 | 1 |
| O[C@@H](c1cc(O)c(O)cc1)C[NH2+]C | 1 |
| O(c1cc2c(CCNC(=O)C)cccc2cc1)C | 1 |
| S(=O)(=O)(CCc1cc2c(C[C@H]3[N@@H+](CCC3)C)c[nH]c2cc1)c1ccccc1 | 1 |
| O(c1cc([C@H]([NH+](C)C)C)ccc1)C(=O)N(CC)C | 1 |
| O=C1C2(C(C3C(C4(C(=CC3)CC(O)CC4)C)CC2)CC1)C | 1 |
| SC[C@H](NC(=O)CC[C@H]([NH3+])C(=O)[O-])C(=O)NCC(=O)[O-] | 1 |
| [O-]C(=O)[C@@H]([NH3+])CCC(=O)N | 1 |
| ClC(CCl)CCl | 1 |
| BrC(CO)CBr | 1 |
| ClC1C(Cl)C(Cl)C=C(Cl)C1Cl | 1 |
| Clc1c(Cl)cc(Cl)c(Cl)c1 | 1 |
| Nc1c(cc(cc1)C)C | 1 |
| P(=O)(OCC1OC(n2c3ncnc(N)c3nc2)C(O)C1O)(OP(=O)(OCC(O)C(O)C(O)Cn1c2c(nc3c1nc(=O)[nH]c3=O)cc(c(c2)C)C)[O-])[O-] | 1 |
| O([C@@H](COC(=O)[C@@H]([NH3+])C(C)C)CO)Cn1c2[nH]c(nc(=O)c2nc1)N | 1 |
| S1[C@H]2N([C@@H](C1(C)C)C(=O)[O-])C(=O)[C@H]2NC(=O)[C@H]([NH3+])c1ccccc1 | 1 |
| n1(c2c(nc1N)c1c(nccc1)cc2C)C | 1 |
| Fc1cn([C@@H]2O[C@@H]([C@@H](O)[C@H]2O)C)c(=O)nc1NC(=O)OCCCCC | 1 |
| P(=O)(OCOC(=O)C(C)(C)C)(OCOC(=O)C(C)(C)C)COCCn1c2ncnc(N)c2nc1 | 1 |
| Clc1c(CC[NH+]2CCN(CC2)c2nsc3c2cccc3)cc2c(NC(=O)C2)c1 | 1 |
| s1c([C@@H](Oc2c3c(ccc2)cccc3)CC[NH2+]C)ccc1 | 1 |
| S1C[C@H](O[C@H]1CO)n1ccc(nc1=O)N | 1 |
| Fc1ccc(c2n(c(C(C)C)c(c2c2ccccc2)C(=O)Nc2ccccc2)CC[C@@H](O)C[C@@H](O)CC(=O)[O-])cc1 | 1 |
| P(=O)([O-])([O-])CO[C@@H](Cn1ccc(nc1=O)N)CO | 1 |
| N(=N\c1ccccc1)/c1ccc(N)cc1 | 1 |
| O=c1n(n(c(c1N(C)C)C)C)c1ccccc1 | 1 |
| S(=O)(=O)([O-])CCS | 1 |
| O1C(n2ccc(nc2=O)N)C(O)C(O)C1CO | 1 |
| P(=O)(OC[C@H]1O[C@@H](n2c3ncnc(N)c3nc2)[C@H](O)[C@@H]1O)(OP(=O)(OP(=O)([O-])[O-])[O-])[O-] | 1 |
| O=C1N2N(CCC[C@H]2C(=O)[O-])CCC[C@@H]1[NH2+][C@@H](CCc1ccccc1)C(=O)OCC | 1 |
| S1[C@H]2N(C(=C(C[N+]3(CCCC3)C)C1)C(=O)[O-])C(=O)[C@H]2NC(=O)/C(=N\OC)/c1nc(sc1)N | 1 |
| S1[C@H]2N(C(=O)[C@H]2NC(=O)/C(=N\OC)/c2nc(sc2)N)C(=C(C1)CSc1n([nH]c(=O)c(=O)n1)C)C(=O)[O-] | 1 |
| O(c1cc2c(c3c(C2=O)cc(OCC[NH+](CC)CC)cc3)cc1)CC[NH+](CC)CC | 1 |
| O=C(N1[C@H]2[C@@H](C[C@H]1C(=O)[O-])CCC2)[C@H]([NH2+][C@@H](CCc1ccccc1)C(=O)[O-])C | 1 |
| Clc1c(Oc2ccc(Oc3ncc(Cl)cc3Cl)cc2)ncc(Cl)c1 | 1 |
| O=C1N(c2c(CC[C@@H]1[NH2+][C@@H](CCc1ccccc1)C(=O)OCC)cccc2)CC(=O)[O-] | 1 |
| O=C(N1[C@@H](CCC1)C(=O)[O-])[C@H]([NH2+][C@@H](CCc1ccccc1)C(=O)[O-])CCCC[NH3+] | 1 |
| O([C@@H]1[C@@H]2[C@H]([C@H](C=CC2=C[C@@H](C1)C)C)CC[C@H]1OC(=O)C[C@H](O)C1)C(=O)[C@H](CC)C | 1 |
| [nH]1c2c(c3c1cccc3)c(nc(N)c2)C | 1 |
| O1CC[NH+](CC1)CCOC(=O)CC/C(=C/Cc1c(OC)c(c2c(c1O)C(=O)OC2)C)/C | 1 |
| Fc1cc2onc([C@H]3CC[N@H+](CC3)CCc3c(=O)n4CCCCc4nc3C)c2cc1 | 1 |
| S(=O)(c1[nH]c2c(n1)ccc(OC(F)F)c2)Cc1nccc(OC)c1OC | 1 |
| P(=O)([O-])([O-])C(P(=O)([O-])[O-])(O)CC[NH3+] | 1 |
| O=C1C(CCc2n(c3c(c12)cccc3)C)Cn1c(ncc1)C | 1 |
| s1c2=Nc3c(NC(=c2cc1C)N1CC[N@H+](CC1)C)cccc3 | 1 |
| Fc1c(N2CC[N@H+](CC2)C)c2OC[C@H](n3c2c(c1)c(=O)c(c3)C(=O)[O-])C | 1 |
| OC1C2(C(C3C(CC2)c2c(CC3)cc(O)cc2)CC1)C | 1 |
| O(C(=O)C1=C(NC(=C(C1c1c([N+](=O)[O-])cccc1)C(=O)OC)C)C)C | 1 |
| S(=O)(=O)(N(c1nc(C(C)C)c(c(n1)c1ccc(F)cc1)/C=C/[C@@H](O)C[C@@H](O)CC(=O)[O-])C)C | 1 |
| Clc1cc(N2CC[NH+](CC2)CCCn2nc(n(c2=O)CCOc2ccccc2)CC)ccc1 | 1 |
| Clc1cc(C(=O)[C@@H]([NH2+]C(C)(C)C)C)ccc1 | 1 |
| O[C@@H](c1c(OC)ccc(OC)c1)CNC(=O)C[NH3+] | 1 |
| Clc1cc(C(=O)NCC[NH+](CC)CC)c(OC)cc1N | 1 |
| [NH3+][C@@]12C[C@@]3(C[C@@](C1)(C[C@H](C3)C2)C)C | 1 |
| Clc1ccc(c2oc3c(n2)cc(C(C)C(=O)[O-])cc3)cc1 | 1 |
| Clc1cc2C(=NC(O)C(=O)Nc2cc1)c1c(Cl)cccc1 | 1 |
| [O-][N+](=O)c1c2c3c4c(ccc3cc1)c([N+](=O)[O-])ccc4cc2 | 1 |
| FC(F)(F)c1ccc(NC(=O)c2c(onc2)C)cc1 | 1 |
| Clc1c(c2nnc(nc2N)N)cccc1Cl | 1 |
| Clc1c(S(=O)(=O)N)cc(C(=O)NN2C(Cc3c2cccc3)C)cc1 | 1 |
| N1(CCC[NH+](C)C)c2c(CCc3c1cccc3)cccc2 | 1 |
| [NH+](CCCc1ccccc1)(CCCc1ccccc1)CC | 1 |
| S(=O)(=O)([N-]C(=O)NC1CCC(CC1)C)c1ccc(CCNC(=O)N2CC(=C(C2=O)CC)C)cc1 | 1 |
| O(C(CO)CO)Cn1c2[nH]c(nc(=O)c2nc1)N | 1 |
| Fc1c(C(O)(Cn2ncnc2)Cn2ncnc2)ccc(F)c1 | 1 |
| s1nc(N2CCOCC2)c(OC[C@@H](O)C[NH2+]C(C)(C)C)n1 | 1 |
| O=C1[C@@H](C[C@H]2CC[N@H+](CC2)Cc2ccccc2)Cc2c1cc(OC)c(OC)c2 | 1 |
| O[C@@H]1[C@H]2[C@H]3[C@@]([C@H](CC3)[C@@H](CCC(=O)[O-])C)(CC[C@@H]2[C@@]2([C@H](C1)C[C@H](O)CC2)C)C | 1 |
| Clc1c(Nc2c(CC(=O)[O-])cccc2)c(Cl)ccc1 | 1 |
| Fc1ccc([C@]2(OCc3c2ccc(c3)C#N)CCC[NH+](C)C)cc1 | 1 |
| S(Cc1nc[nH]c1C)CCN/C(=N/C)/NC#N | 1 |
| O(CCCCc1n(nnn1)C1CCCCC1)c1cc2CCC(=O)Nc2cc1 | 1 |
| O(CC(CCC)(COC(=O)N)C)C(=O)NC(C)C | 1 |
| O=C(N1c2c(C=Cc3c1cccc3)cccc2)N | 1 |
| SCC(C(=O)N1C(CCC1)C(=O)[O-])C | 1 |
| O(c1n(Cc2ccc(cc2)c2c(cccc2)c2n[n-]nn2)c2c(n1)cccc2C(=O)[O-])CC | 1 |
| O(C1CCCCC1)C(=O)OC(OC(=O)c1c2n(Cc3ccc(cc3)c3c(cccc3)c3n[n-]nn3)c(OCC)nc2ccc1)C | 1 |
| OC(CCCC(C1C2(C(CC1)/C(=C/C=C/1\CC(O)CC(O)C1=C)/CCC2)C)C)(C)C | 1 |
| OC(C1CC1)/C=C/C(C1C2(C(CC1)/C(=C/C=C/1\CC(O)CC(O)C1=C)/CCC2)C)C | 1 |
| S(=O)(=O)(OCCCCOS(=O)(=O)C)C | 1 |
| O1[C@H]2[C@]34[C@@]5(C[C@H]([C@]2(OC)CC5)[C@](O)(C(C)(C)C)C)C([N@H+](CC3)CC2CC2)Cc2c4c1c(O)cc2 | 1 |
| O=C(Nc1c(cccc1C)C)[C@H]1[N@H+](CCCC1)CCCC | 1 |
| S(=O)(=O)(N)c1c(Oc2ccccc2)c(NCCCC)cc(c1)C(=O)[O-] | 1 |
| O=C(CCC[NH+]1CCCC1)c1c(OC)cc(OC)cc1OC | 1 |
| SC(C(=O)NC(CS)C(=O)[O-])(C)C | 1 |
| Brc1sc2n3c(nnc3C)CN=C(c2c1)c1c(Cl)cccc1 | 1 |
| Brc1cc(C(=O)NCC[NH+](CC)CC)c(OC)cc1N | 1 |
| Brc1[nH]c2c3c(C4=C[C@@H](C(=O)N[C@@]5(O[C@@]6(O)N([C@@H](C(=O)N7[C@@H]6CCC7)CC(C)C)C5=O)C(C)C)C[N@@H+]([C@@H]4Cc13)C)ccc2 | 1 |
| Brc1cc2c(NC(=O)CN=C2c2ncccc2)cc1 | 1 |
| O[C@@]([C@H]1[C@H]2C[C@H](C1)C=C2)(CC[NH+]1CCCCC1)c1ccccc1 | 1 |
| P(=O)(OCC1OC(n2c3ncnc(N)c3nc2)C(O)C1O)(OP(=O)(OP(=O)([O-])[O-])[O-])[O-] | 1 |
| O(CC1CC1)CCc1ccc(OC[C@@H](O)C[NH2+]C(C)C)cc1 | 1 |
| P(=O)(OCC(=O)C1(O)C2(C(C3C(F)(C(O)C2)C2(C(=CC(=O)C=C2)CC3)C)CC1C)C)([O-])[O-] | 1 |
| [NH2+](CCc1ncccc1)C | 1 |
| o1c2c(c(OC)c3c(oc(=O)cc3)c2)cc1 | 1 |
| O1C2C(C(C(O)C2)/C=C/C(O)C(CC#CC)C)c2c1c(ccc2)CCCC(=O)[O-] | 1 |
| O(C[C@@H]([NH+]1CCCC1)CN(Cc1ccccc1)c1ccccc1)CC(C)C | 1 |
| O1C(C(O)CO)C(=O)C(=C1[O-])O | 1 |
| S1C2N(C(C1(C)C)C(=O)[O-])C(=O)C2NC(=O)Cc1ccccc1 | 1 |
| c12c3c4ccc1c1c(cc2ccc3ccc4)cccc1 | 1 |
| Brc1cc(C(=O)c2c3c(oc2CC)cccc3)cc(Br)c1O | 1 |
| O([C@H]1CCC[N@@H+](C1)Cc1ccccc1)C(=O)C1=C(NC(=C([C@H]1c1cc([N+](=O)[O-])ccc1)C(=O)OC)C)C | 1 |
| FC(F)(F)c1cc(C[C@H]([NH2+]CCOC(=O)c2ccccc2)C)ccc1 | 1 |
| ClC12C(C3C(C(O)(C(C3)C)C(=O)CO)(CC1O)C)CCC1=CC(=O)C=CC21C | 1 |
| ClC12C(C3C(C(OC(=O)CC)(C(C3)C)C(=O)COC(=O)CC)(CC1O)C)CCC1=CC(=O)C=CC21C | 1 |
| O=C1NC(=O)NC(=O)C1(CC)CC | 1 |
| S(=O)(=O)([O-])N1C(C(NC(=O)/C(=N\OC(C)(C)C(=O)[O-])/c2nc(sc2)N)C1=O)C | 1 |
| Clc1c(S(=O)(=O)N)cc(c(NCc2sccc2)c1)c1n[n-]nn1 | 1 |
| [O-]C(=O)c1c(N)cccc1 | 1 |
| O([C@H]1[C@@H]([C@H](O[C@H]2O[C@@H]([C@@H](O)[C@@](OC)(C2)C)C)[C@@H](C(=O)O[C@@H]([C@@](O)([C@@H](O)[C@H]([N@@H+](C[C@H](C[C@]1(O)C)C)C)C)C)CC)C)C)[C@H]1O[C@@H](C[C@@H]([NH+](C)C)[C@H]1O)C | 1 |
| [O-]C(=O)CCCCCCCC(=O)[O-] | 1 |
| S(c1n(cnc1[N+](=O)[O-])C)c1ncnc2nc[nH]c12 | 1 |
| Clc1cc(C(=O)N[C@H]2[C@H]3CC[N@H+](C2)CC3)c2OCC(=O)N(c2c1)C | 1 |
| O(C[C@@H](O)C[NH2+]C(C)C)c1ccc(cc1)CC(=O)N | 1 |
| Fc1ccc(Cn2c(N[C@H]3CC[N@H+](CC3)CCc3ccc(OC)cc3)nc3c2cccc3)cc1 | 1 |
| O(c1c(cccc1)C(=O)[O-])C(=O)C | 1 |
| S(C[C@@H](O)C[NH2+]C(C)(C)C)c1scc(n1)c1sc(cc1)C(=O)N | 1 |
| S(=O)(=O)(NC(C(=O)N1C(CC(CC1)C)C(=O)[O-])CCC[NH+]=C(N)N)c1c2NCC(Cc2ccc1)C | 1 |
| N(C1Cc2c(C1)cccc2)(CCC[NH+](CC)CC)c1ccccc1 | 1 |
| n1(ncnc1)Cc1cc(C(C)(C)C#N)cc(C(C)(C)C#N)c1 | 1 |
| Clc1c2CN3C(=NC(=O)C3)Nc2ccc1Cl | 1 |
| S(=O)(=O)(Nc1cc(OC)c(Nc2c3c([nH+]c4c2cccc4)cccc3)cc1)C | 1 |
| S(=O)(=O)(N(CC(O)C(NC(=O)OC1CCOC1)Cc1ccccc1)CC(C)C)c1ccc(N)cc1 | 1 |
| O1C2N(C(/C/1=C/CO)C(=O)[O-])C(=O)C2 | 1 |
| S1[C@H]2N([C@@H](C1(C)C)C(=O)[O-])C(=O)[C@H]2NC(=O)[C@H]([NH3+])c1ccc(O)cc1 | 1 |
| Clc1cc2C(=Nc3c(Oc2cc1)cccc3)N1CC[NH2+]CC1 | 1 |
| Clc1cc2[nH+]ccc(Nc3cc(C[NH+](CC)CC)c(O)cc3)c2cc1 | 1 |
| Clc1c(cc(NC(=O)Nc2ccc(Oc3cc(ncc3)C(=O)NC)cc2)cc1)C(F)(F)F | 1 |
| Clc1c([C@H]2C(=C(NC(=C2C(=O)OC)C)COCC[NH3+])C(=O)OCC)cccc1 | 1 |
| o1c2c(cc(C(C)C)cc2)c(=O)c2c1nc(N)c(c2)C(=O)[O-] | 1 |
| [NH+](CCC=C1c2c(CCc3c1cccc3)cccc2)(C)C | 1 |
| S(=O)(=O)(c1cc(C(=O)NC[C@H]2[N@H+](CCC2)CC)c(OC)cc1N)CC | 1 |
| Ic1c(OCC[NH+](CC)CC)c(I)cc(C(=O)c2c(oc3c2cccc3)CCCC)c1 | 1 |
| O=c1n(c(=O)n(c2nc[nH]c12)C)C | 1 |
| S(P(=O)([O-])[O-])CC[NH2+]CCC[NH3+] | 1 |
| Brc1c(N)c(C[NH2+][C@H]2CC[C@H](O)CC2)cc(Br)c1 | 1 |
| [NH3+][C@@]12C[C@H]3C[C@H](C2)C[C@@H](C1)C3 | 1 |
| Clc1cc2c(n3c(nnc3C)CN=C2c2ccccc2)cc1 | 1 |
| O1C(CCCC(CCCC(CCCC(C)C)C)C)(CCc2c1c(c(c(OC(=O)C)c2C)C)C)C | 1 |
| O=c1ncnc2[nH][nH]cc12 | 1 |
| O1C(CCC1)C(=O)NCCCN(c1nc2c(c(n1)N)cc(OC)c(OC)c2)C | 1 |
| OC1C/C(=C\C=C/2\C3C(C(CC3)C(CCCC(C)C)C)(CCC2)C)/C(=C)C(O)C1 | 1 |
| S(=O)(=O)(Nc1cc2c(c(oc2cc1)CCCC)C(=O)c1ccc(OCCC[NH+](CCCC)CCCC)cc1)C | 1 |
| P(=O)([O-])([O-])C(P(=O)([O-])[O-])(O)CCC[NH3+] | 1 |
| O[C@@H](C[NH2+]C(C)(C)C)c1cc(c(O)cc1)CO | 1 |
| S(c1cc2[nH]c(nc2cc1)NC(=O)OC)CCC | 1 |
| ClCC(=O)N(c1c(CC)cccc1C)COCC | 1 |
| S(=O)(=O)(N)c1sc(NC(=O)C)nn1 | 1 |
| Oc1ccc(NC(=O)C)cc1 | 1 |
| Clc1ccc(C(=O)n2c(c(c3c2ccc(OC)c3)CC(=O)OCC(=O)[O-])C)cc1 | 1 |
| O(C[C@@H](O)C[NH2+]C(C)C)c1c(cc(NC(=O)CCC)cc1)C(=O)C | 1 |
| Clc1ccc([C@H]([NH+]2CC[NH+](CC2)CCOCC(=O)[O-])c2ccccc2)cc1 | 1 |
| Clc1nc2n(C3OC(C(O)C3)CO)cnc2c(n1)N | 1 |
| s1c(c([n+](Cc2c(nc(nc2)C)N)c1)C)CCO | 1 |
| S(=O)(=O)([O-])CC[NH3+] | 1 |
| OC/C=C(/C=C/C=C(/C=C/C1=C(CCCC1(C)C)C)\C)\C | 1 |
| Oc1c(c(CO)cnc1C)CO | 1 |
| P(=O)(OCc1c(c(O)c(nc1)C)C=O)([O-])[O-] | 1 |
| O=C(N)c1nccnc1 | 1 |
| C[C@H]1C[C@H](C)O[C@@]2(C1)CCCC(=C)C2 | 0 |
| CCC[C@H]1CCc2c(N1)cccc2 | 0 |
| CC(c1ocnn1)C | 0 |
| OCCCNC(=O)c1csc(n1)c1cc2c([nH]1)cccc2 | 0 |
| O=C(Nc1ccc2c(c1)C(=O)Nc1c(O2)ccc(c1)C)NCc1cccc2c1cccc2 | 0 |
| O=C(NC(c1ccccc1)c1ccccc1)Nc1ccc2c(c1)C(=O)Nc1c(O2)ccc(c1)C | 0 |
| O=C(Nc1ccc(c(c1)Cl)Cl)Nc1ccc2c(c1)C(=O)Nc1c(O2)ccc(c1)C | 0 |
| CN(c1ccccc1)C1CCCC1 | 0 |
| COc1cc2ccccc2cc1C | 0 |
| Cc1ccc(cc1)S[C@@H]1O[C@@H]2CO[C@@H](O[C@@H]2[C@@H]([C@H]1O)O)c1ccccc1 | 0 |
| O=C(Nc1ccc(cc1)C(C)(C)C)Nc1ccc2c(c1)C(=O)Nc1c(O2)ccc(c1)C | 0 |
| O=C(Nc1ccccc1C(F)(F)F)Nc1ccc2c(c1)C(=O)Nc1c(O2)cccc1 | 0 |
| OC[C@]12C=C[C@H]([C@@]1(C)CC[C@@]1([C@@H]2CC1(C)C)O)O | 0 |
| O=C1CC(CC(=O)C1)c1ccncc1 | 0 |
| F[C@H]1CCN(C1)Cc1ccccc1 | 0 |
| Cc1ccc(cc1)S(=O)(=O)N1CCC(=O)c2c1cc(Br)cc2 | 0 |
| Cc1nn2c(c1c1ccccc1)[nH]cnc2=O | 0 |
| O=C(Oc1ccc(cc1)I)Cc1ccc(cc1)Cl | 0 |
| [O-]C(=O)[C@H]1[NH+]=C(c2ccccc2)c2c(NC1(O)O)ccc(c2)Cl | 0 |
| Clc1ncnc(c1)c1ccc(c(c1Cl)Cl)Cl | 0 |
| OCC1CC(=O)C1 | 0 |
| C#CCOc1c(C)cccc1C | 0 |
| Brc1ccc2c(c1)C=CCO2 | 0 |
| C[C@H](Oc1ccc(cc1)C)C#C | 0 |
| N#C[C@@]1(C(=O)[C@H]2[C@H]1C[C@@H]1[C@@H]2C(C1=O)(c1ccccc1)c1ccccc1)C(C)(C)C | 0 |
| N#C[C@]1(C(=O)[C@@H]2[C@H]1CC[C@@H]1[C@@H]2C(C1=O)(c1ccccc1)c1ccccc1)C(C)(C)C | 0 |
| CCC([C@H](C#N)C)(CC)O | 0 |
| Ic1ccc(cc1)/C=C\1/C=Cc2c1cccc2 | 0 |
| C1CC(C1)CNc1ccccc1 | 0 |
| C[C@@H](c1ccccc1)NCC(F)F | 0 |
| C1CCC(C1)Oc1ccccc1 | 0 |
| O=C1NC2(CCN(CC2)C(=O)c2cccc3c2cccc3)Nc2c1cccc2 | 0 |
| CC[C@@H](C(=O)N1CCOCC1)Oc1ccc2c(c1)[C@H](c1ccc(cc1)F)N(CC2)C(=O)C(C)C | 0 |
| Fc1cccc(c1)C(=O)N(c1ccc(cc1)CC(=O)N1CCN(CC1)C(=O)C)Cc1ccccc1 | 0 |
| COc1ccc(cc1)CN(C(=O)c1cccc(c1)F)c1ccc(cc1)CC(=O)N1CCOCC1 | 0 |
| CC(CN(S(=O)(=O)c1ccsc1C(=O)[O-])CC[NH+](C)C)C | 0 |
| [O-]C(=O)c1[nH]cc(c1)S(=O)(=O)NCCC[NH+]1CCCCC1 | 0 |
| NCc1nc2c([nH]1)ccc(c2)Br | 0 |
| OCc1cc(oc1C)C | 0 |
| COCC(=O)N1CCN(CC1)c1nc(nc(c1Cc1c(F)cccc1Cl)C)c1ccccc1 | 0 |
| COCC(=O)N1CCN(CC1)c1nc(nc(c1Cc1cc(C)ccc1C)CC)c1ccccc1 | 0 |
| Brc1ccc(c(c1)C(=O)c1c[nH]c2c1cccn2)Cl | 0 |
| Cc1cccc(c1)[C@@H]1N(CCc2c1cc(cc2)O[C@@H](C(=O)N1CCOCC1)C)C(=O)C1CCCC1 | 0 |
| O=C(N1CCN(CC1)C(=O)C)Cc1ccc(cc1)N(C(=O)c1ccccc1C)Cc1ccccc1 | 0 |
| OCC1CCN(CC1)c1ccc(cc1CO)F | 0 |
| Fc1ccc(cc1CNCC#C)F | 0 |
| Nc1cccc(c1NCC1CCC1)Cl | 0 |
| Nc1ccc(c(c1)F)NCC1CCC1 | 0 |
| Nc1cccc(c1)NCC1CCC1 | 0 |
| Nc1ccc(c(c1)C)NCC1CCC1 | 0 |
| Cc1ccc(c(c1)N)NCC1CCC1 | 0 |
| C[C@H]1Oc2c(C1)cc(cc2)Br | 0 |
| Fc1ccc(cc1)NCC1CC1 | 0 |
| Cc1ccc(cc1C)NCC1CC1 | 0 |
| Cc1ccc(c(c1)N)N1CCNC1=O | 0 |
| Fc1cc(Br)cc(c1)c1sc2c(c1)ncnc2Cl | 0 |
| CC(NC1(CCC1)C#N)C | 0 |
| Fc1cccc(c1)CN(Cc1cc(n(c1C)c1ccccn1)C)CCCn1cncc1 | 0 |
| O=c1nc2c3ccccc3Cn2c2c1c1CC[C@H](Cc1s2)c1ccccc1 | 0 |
| Cc1cc(nc2c1c1nc([nH]c(=O)c1s2)c1ccc(s1)Cc1ccccc1)C(F)(F)F | 0 |
| COc1cc2c(cc1OC)ncn(c2=O)CC(=O)N[C@@H](C(=O)/N=c\1/sc2c([nH]1)cccc2)C | 0 |
| CC[NH+](CCC[N-]C1=NC=NC2=NN=C[C@@H]12)CC | 0 |
| OC[C@@H]1O[C@H](Nc2ccc(cn2)Br)[C@@H]([C@@H]([C@@H]1O)O)O | 0 |
| OC[C@@H]1O[C@H]([C@@H]([C@H]1F)O)O | 0 |
| COc1cc(F)c(cc1F)C#C | 0 |
| COC(=O)c1ncccc1Br | 0 |
| COc1cc2CCN([C@@H](c2cc1Oc1c(O)c(OC)cc2c1[C@@H](CC)N(CC2)C)CC)C | 0 |
| NC(=O)c1cccc2c1[nH]c(n2)C(N)(C)C | 0 |
| Brc1ccc2c(c1)C=CC(O2)(C)C | 0 |
| Brc1ccc(s1)S(=O)(=O)NCc1sc2c(c1)cc(cc2)F | 0 |
| CCn1c2ccc(cc2c2c1cccc2)/C=C\1/C=Cc2c1cccc2 | 0 |
| CN1CCN(CC1)S(=O)(=O)c1ccc(cc1)NC(=O)c1cc(nc2c1cnn2C(C)C)C(C)C | 0 |
| COC(=O)c1c(C)[nH]c(c1[C@H]1C(=O)Nc2c1cc(C)cc2)c1ccc(cc1OC)F | 0 |
| COc1cc(NC(=O)c2ccc3c(c2)ccn3Cc2ccccc2)cc(c1OC)OC | 0 |
| CSc1ccccc1N[C@@H](c1cnn2c1N[C@H](C[C@@H]2C(F)(F)F)c1ccc(cc1)F)O | 0 |
| C#CCn1c(C)ccc1C | 0 |
| CCOc1ccc[nH]c1=O | 0 |
| Cc1cc(cc(c1)C)NC(=O)c1ccc(cc1)C/N=C/1\C(=C(C1=[N+]1CCCC1)[O-])O | 0 |
| O=C(Cc1ccc(cc1)F)NCC(O)(C)C | 0 |
| c1ccc(cc1)[C@@H]1[N-]C[C@@H]2[N@H+]1CCC2 | 0 |
| N#CCc1ccc(c(c1)Br)N | 0 |
| COc1cccc(c1)C(=O)N1CCC2(CC1)OC[C@H](N2C(=O)c1cccc(c1)F)C(=O)NCc1ccco1 | 0 |
| Cc1cccc(c1)Oc1c(CN(C(=O)C2CCC2)CC2CC2)c(nn1c1ccccc1)C | 0 |
| CCc1nn(c(c1CN(C(=O)C1CCC1)C1CC1)Oc1ccc(cc1)F)c1ccccc1 | 0 |
| CCc1nn(c(c1CN(C1CC1)C(=O)C)Oc1ccc(cc1)C)c1ccccc1 | 0 |
| O=C(Nc1ccc2c(c1)[nH]c(=O)[nH]2)CN1CCN(CC1)S(=O)(=O)c1cccs1 | 0 |
| N#Cc1c(I)nsc1c1cc(Cl)cc(c1)Cl | 0 |
| Fc1ccc(cc1)Cn1cccc1C[N@H+](C1CC1)Cc1ccc(cc1)C(C)(C)C | 0 |
| C=CC[N@H+](Cc1cccn1Cc1ccc(cc1)F)Cc1ccc(cc1)C(C)(C)C | 0 |
| Fc1ccc(cc1)Cn1cccc1C[N@@H+](C1CCCCC1)Cc1cccc(c1)C(F)(F)F | 0 |
| CC([C@H]([N@H+](Cc1cccn1Cc1ccc(cc1)C(F)(F)F)Cc1ccccc1F)C)C | 0 |
| C=CC[N@@H+](Cc1cccn1Cc1cccc(c1)C(F)(F)F)Cc1ccccc1 | 0 |
| Cc1cccc(c1)Cn1cccc1C[N@H+](Cc1ccccc1C)CC1CCCCC1 | 0 |
| Cc1ccc(cc1)C[N@H+]([C@H](C(C)C)C)Cc1cccn1Cc1ccc(cc1)C | 0 |
| Cc1ccccc1C[N@H+](Cc1cccn1Cc1ccccc1F)CC1CCCCC1 | 0 |
| CC(CC[N@@H+](Cc1cccn1Cc1ccc(cc1)F)Cc1ccccc1C)C | 0 |
| CCOc1ccc(cc1)N1[C@H](Nc2cccc(c2)C(=O)N2CCCC2)c2c(C1=O)cccc2 | 0 |
| OC[C@@H](Nc1ccc(cn1)Br)C | 0 |
| O=C(c1ccnc(c1)[C@@]12C[C@H]3C[C@@H](C1)C[C@@H](C2)C3)Nc1c(Cl)cccc1Cl | 0 |
| Brc1ccc(cc1)NC(=O)c1ccnc(c1)[C@@]12C[C@H]3C[C@@H](C1)C[C@@H](C2)C3 | 0 |
| COc1ccc2c(c1)NC1(c3n2ccc3)CCN(CC1)C(=O)Nc1ccc2c(c1)OCCO2 | 0 |
| OCCNC(=O)c1cc(F)cc(c1)Br | 0 |
| O=C(Nc1cc(Cl)ccc1n1ncnc1)CN1CCN(CC1)S(=O)(=O)/C=C/c1ccccc1 | 0 |
| COc1ccc(cc1)NC(=O)[C@H]1C[C@@H](N[C@]21C(=O)Nc1c2cc(C)cc1Cl)Cc1ccccc1 | 0 |
| N#CC1(C#N)[C@@H](c2cccc(c2)Cl)[C@@H](N2[C@H]1c1ccccc1C=C2)C(=O)C | 0 |
| C/C=C\[C@H]1OCC2[C@@H](C1C(=C[C@H]2C)C)C | 0 |
| COc1cc2CCC(=O)Nc2cc1N | 0 |
| Cc1cc(nc2c1ccc(c2)Cl)N1CCCCCC1 | 0 |
| N#Cc1c2CCCC(c2nc(c1C#N)Cl)(Br)Br | 0 |
| O=C(Nc1ccc(cc1)F)CN1CCN(CC1)C(=O)CSc1nnc(o1)c1ccc(cc1)Cl | 0 |
| C[N@H+](Cc1ccccc1OC(F)F)CC(=O)[O-] | 0 |
| [O-]C(=O)[C@H]1CCCC[N@@H+]1Cc1ccc(c2c1nccc2)F | 0 |
| C1CCCN(CC1)c1nc2ccccc2c2c1cccc2 | 0 |
| Clc1ccc(cc1)C#CC#CC(N(C)C)(C)C | 0 |
| COc1ccccc1NC(=O)c1cccc(c1)N1CCCN(C1=O)Cc1ccccc1F | 0 |
| Fc1cc(F)cc(c1)n1cccc1 | 0 |
| C1CC1n1cccc1 | 0 |
| Cc1ccc(n1C1CC1)C | 0 |
| CN(C(C#CBr)(C)C)C | 0 |
| Ic1ccc(cc1)NC(=O)c1ccc(o1)c1ccc(c(c1)Cl)Cl | 0 |
| CCn1nc(c2ccccc2)c2c(c1c1ccccc1)c(Br)cc2Br | 0 |
| COc1cc(c(cc1OC)Br)[C@@H](c1cccc(c1)Br)N | 0 |
| Fc1cc(F)c(c(c1)F)c1n[nH]c(=O)c2c1cccc2 | 0 |
| Brc1ccc2c(c1)C(=O)NC1(N2)CCN(CC1)C(=O)Nc1cccc(c1)C(F)(F)F | 0 |
| CC[C@H](NC(=O)c1nn2c(c1)C(=O)N(CC2)Cc1ccc(cc1)OC)C | 0 |
| Clc1cccc(c1Cl)c1ccc(o1)/C=C\c1cc(nc(=O)[nH]1)C(F)(F)F | 0 |
| Clc1cc(N)c2c(c1)cn[nH]2 | 0 |
| COc1ccc(c(c1)NC(=O)C1CCC1)N1CCCN(C1=O)Cc1cccc(c1)Cl | 0 |
| COc1ccc(c(c1)NC(=O)CC(C)(C)C)N1CCCN(C1=O)Cc1ccccc1 | 0 |
| COc1ccccc1C(=O)Nc1cc(C)ccc1N1CCCN(C1=O)Cc1ccc(cc1)C | 0 |
| Clc1ccccc1[C@H](Oc1ccccc1F)CC[NH+]1CCN(CC1)Cc1ccccc1 | 0 |
| Cc1ccc(cc1C)c1csc(=O)[nH]1 | 0 |
| O=c1sc2c([nH]1)c1ccccc1C(C2)(C)C | 0 |
| O=c1[nH]c2c(s1)CCCCCCCCCC2 | 0 |
| CCC[C@H]1NCCc2c1cccc2 | 0 |
| COc1ccc(cc1)C=C1CC1 | 0 |
| Cn1ccc(c1)C(=C)C | 0 |
| COc1cc2CCCCc2cc1C | 0 |
| CC1(C)CCC[C@]2([C@H]1CC[C@@]1([C@H]2CCO1)C)C | 0 |
| C1CC=C[C@H](C1)Oc1ccccc1 | 0 |
| Clc1ccc2c(c1)cc(n2C)I | 0 |
| C#CCNc1ccc(c(c1)C(F)(F)F)F | 0 |
| Fc1ccc(cc1)Cn1cccc1 | 0 |
| Nc1cc(N)c(cc1F)Br | 0 |
| Cc1cc2cc3c(cc2cc1C)c1c3nc2c(n1)cccc2 | 0 |
| CNc1ccc(cc1N)Br | 0 |
| [O-]C(=O)C1(N)C[NH2+]C1 | 0 |
| C=CC[C@]12CCOC1=CCC2 | 0 |
| CNS(=O)(=O)c1ccc2c(c1)cc([nH]2)O | 0 |
| COc1ccnnc1 | 0 |
| C[C@@H]1CCCCCO1 | 0 |
| C#CCNc1ccc(c(c1)Cl)Cl | 0 |
| O=C1CCc2c(N1C)ccc(c2)C(=O)C | 0 |
| Oc1cc2ccccc2c(c1)N | 0 |
| O=C(N1CCCCC1)CC(F)(F)F | 0 |
| [O-]C(=O)[C@@]1([NH3+])CCCNC1 | 0 |
| C#CCOc1cccc(c1)Cl | 0 |
| CC(=CCc1cccc2c1NCC2)C | 0 |
| Brc1cccc(n1)N(C)C | 0 |
| C1CCC(CC1)n1cccc1 | 0 |
| C#CCOc1ccccc1F | 0 |
| CN1Cc2ccccc2[C@@H](C1)c1ccccc1 | 0 |
| Nc1ccsc1Br | 0 |
| CNc1nncn1C | 0 |
| Cc1nc(co1)C#N | 0 |
| Brc1ccc2c(c1)C(=Cc1ccc(cc1)N(C)C)c1c2ccc(c1)Br | 0 |
| CN1Cc2ccccc2C[C@@H]1c1ccccc1 | 0 |
| O=C1N[C@@H](c2cccc(c2)Oc2ccccc2)[C@H]([C@@](N1)(O)C(F)(F)F)C(=O)c1ccccc1 | 0 |
| [O-]C(=O)c1c2CC/C(=C/c3ccccc3)/c2[nH+]c2c1cccc2 | 0 |
| CN(c1ccccc1I)C | 0 |
| C[N@H+]1CC[C@](CC1)(C(=O)[O-])c1ccc(c(c1)Cl)Cl | 0 |
| O=C(Nc1snnc1C(=O)NC[C@@H]1CCCO1)NC[C@@H]1CCCO1 | 0 |
| Brc1ccc2c(c1)CCCN2C | 0 |
| Cn1c2ccccc2c(c1C)Br | 0 |
| CN(C(C#CC#Cc1ccccc1)(C)C)C | 0 |
| Clc1cccc(c1)N1CC[NH+](CC1)C/C=C\c1ccccc1 | 0 |
| O=C(Nc1ccc(cc1)Cl)N/C=C\c1ccc(c(c1)Cl)Cl | 0 |
| COCCNC(=O)c1ccccc1NS(=O)(=O)c1c(C)[nH]c(=O)[nH]c1=O | 0 |
| O=C(CSc1nnc(n1C)Cc1c(C)nc2n(c1=O)ccc(c2)C)NCc1nc2c([nH]1)cccc2 | 0 |
| CN1CCN(CC1)C[C@H](c1ccc2c(c1)[nH]cc2)O | 0 |
| COc1cc(nc2c1cc(cc2)NC(=O)Nc1ccc2c(c1)OCO2)c1ccc(cc1)C | 0 |
| COc1cc(nc2c1cc(cc2)NC(=O)Nc1ccc2c(c1)OCCO2)c1ccc(cc1)C | 0 |
| Cc1ccc2c(c1)c(=O)n(cn2)C1CCN(CC1)C(=O)Nc1ccc(c(c1)C)C | 0 |
| Fc1ccc(c(c1)F)NC(=O)N1CCC(CC1)n1cnc2c(c1=O)cc(cc2)C | 0 |
| O=C(Nc1ccc(c(c1)F)F)Nc1cccc(c1)c1noc(n1)C | 0 |
| CC(Cn1c(=O)c2ccc(cc2n2c1nnc2SCC(=O)N)C(=O)NC1CCCC1)C | 0 |
| NC(=O)C1(CCN(CC1)c1ccc(cc1C(=O)[O-])NS(=O)(=O)C)[NH+]1CCCCC1 | 0 |
| Clc1ccc2c(c1)[C@H]1OCC[C@H]1[C@@H](N2)c1ccc(cc1)C(F)(F)F | 0 |
| Cc1ccc(o1)CNC(=O)[C@@H]1CCCN(C1)c1[nH]c2c(n1)cc(cc2)Br | 0 |
| N#Cc1cc2C(=O)C[C@@H](Cc2n(c1=O)c1cccc(c1)F)c1ccccc1 | 0 |
| N#Cc1cc2C(=O)C[C@H](Cc2n(c1=O)c1ccc(cc1)C)c1ccccc1 | 0 |
| CCN(c1c(=O)[nH]c(=O)n(c1N)Cc1ccccc1)C(=O)CSc1nnc(n1c1ccc(cc1)C)C | 0 |
| Nc1cccc(c1C)c1nnc2n1CCC2 | 0 |
| O=C(CCc1n[nH]c(=O)[nH]c1=O)OCC(=O)c1ccccc1 | 0 |
| C[C@@H]1C[C@@H]1C(=O)N(C1CCCC1)C | 0 |
| NC(=O)c1[nH]c2c(c1)c(C)cc(c2C)C | 0 |
| Fc1ccc(cc1)c1csc2c1c(=O)[nH]c(n2)CSc1ncc(cc1Cl)Cl | 0 |
| Fc1ccc(c(c1)Cl)S(=O)(=O)Nc1ccc(c(c1)I)C | 0 |
| CCOC(=O)C1=C(COC(=O)c2nccnc2N)NC(=O)N[C@@H]1CC | 0 |
| CC(Cn1nc(C(=O)N2CCN(CC2)Cc2cccc(c2)C(F)(F)F)c2c(c1=O)cccc2)C | 0 |
| O=C1COc2c(N1)cc(cc2)NC(=O)c1ccc2c(c1)Cc1c2cccc1 | 0 |
| Brc1ccc(cc1)/C=c\1/s/c(=C/2\CCCC2=O)/n(c1=O)c1ccccc1 | 0 |
| O=C(Nc1ccccc1N1CCN(CC1)S(=O)(=O)C)CSc1ncnc2c1[nH]cn2 | 0 |
| COC(=O)C1=C(C)Nc2c([C@@H]1c1ccc(c(c1)OC)O)c(=O)[nH]c(n2)SC | 0 |
| N#C[C@]12C[C@H](C1)CN2Cc1ccccc1 | 0 |
| N#C[C@@]12C[C@H]1CCN2Cc1ccccc1 | 0 |
| Cc1ccc(cc1)N1C[C@H]2N(C1)Cc1c(C2)cccc1 | 0 |
| N#Cc1ccccc1c1ncccn1 | 0 |
| CC[C@H](n1c(=O)cc(c2c1c(OC)ccc2)C)C(=O)Nc1ccc(cc1)S(=O)(=O)N1CCOCC1 | 0 |
| CCCCc1c([O-])[nH+]c([nH]c1=O)Nc1nc(C)c2c(n1)cc(cc2)OC | 0 |
| COc1ccc(cc1)C#Cc1ccc(cc1)C#C | 0 |
| CCOC1CCCCC1 | 0 |
| Cc1cc(C)c2c(c1)c(N)cc(=O)[nH]2 | 0 |
| O[C@H](C(C#N)(C)C)C(C)C | 0 |
| CN1c2ccccc2C([C@H]1C)(C)C | 0 |
| CN(c1ccccc1)CC#C | 0 |
| COc1ccc(cc1)c1n[nH]c(=O)o1 | 0 |
| C#CCn1cccc1 | 0 |
| C1CCc2n(C1)ccc2 | 0 |
| CC(CNc1ccccc1)(C)C | 0 |
| Clc1ccc(c(c1)Nc1ncnc2c1nc[nH]2)O | 0 |
| Brc1ccc2c(c1)C(C)(C)c1c2ccc(c1)Br | 0 |
| COCCN1C(=O)c2ccccc2[C@@H](C21CCCC2)C(=O)N=c1[nH]c2c([nH]1)cccc2 | 0 |
| Nc1ccc(cc1)c1n[nH]c(c1)C | 0 |
| Nc1ccc2c(c1)c(n[nH]2)c1ccccc1 | 0 |
| CC1CCN(CC1)c1nc2c(o1)ccc(c2)N | 0 |
| O=C1Nc2c(OC1(C)C)cccc2N | 0 |
| O=C([C@@H]1CCCN(C1)S(=O)(=O)c1c(C)[nH]c(=O)[nH]c1=O)NCc1ccncc1 | 0 |
| Clc1ccc(c(c1)Cl)CN1CCN(CC1)c1nc(N)nc2c1nc[nH]2 | 0 |
| Brc1ccc(cc1)[C@@H]1c2c(C)[nH]nc2Nc2c1c(=O)[nH]c(=O)[nH]2 | 0 |
| C#CCN1CCCCC1 | 0 |
| Fc1ccc(cc1)c1csc2c1c(=O)[nH]c(n2)SCc1ccc(nc1)Cl | 0 |
| O=C(c1ccc2c(c1)[nH]c(=O)c(=O)[nH]2)NCCCSc1ccccc1 | 0 |
| CO/C=C\c1ccccc1 | 0 |
| Oc1cc(F)c(cc1N)Br | 0 |
| O=C(c1ccccc1C)N[C@@H](c1nc2c([nH]1)cccc2)Cc1ccccc1 | 0 |
| COc1ccc(cc1OC)C(=O)CCNc1ccc2c(c1)n(C)c(=O)n2C | 0 |
| Clc1ccc2c(c1)[C@H]1OCCC[C@H]1[C@H](N2)c1cccc(c1)C | 0 |
| O=C(c1n[nH]c(=O)c2c1cccc2)Nc1ccc(cc1)n1ncc2c1nc[nH]c2=O | 0 |
| Brc1ccc(cc1)c1n[nH]c(c1)C(=O)Nc1ccc2c(c1)OCCCO2 | 0 |
| COc1cc(cc(c1OC)OC)C(=O)Nc1ccnn1Cc1cccc(c1)C | 0 |
| OCCNc1ncccc1I | 0 |
| CSc1cccc(c1)O | 0 |
| [O-]c1nc(nc2c1cccc2)C[N@@H+]([C@@H]1CCc2c1cccc2)C | 0 |
| CCOCCCNC(=O)c1ccn(n1)Cn1nc(c(c1C)Cl)C | 0 |
| COc1ccc(c(c1)OC)Nc1nnc(n(c1=O)N)SCc1ccc(c(c1)OC)OC | 0 |
| O=C1CCC(CC1)(c1cc(F)ccc1F)S(=O)(=O)c1ccc(cc1)Cl | 0 |
| COc1ccc2c(c1[C@@H]1NC(=O)NC(=C1C(=O)Nc1ccc(cc1)Cl)C)cccc2 | 0 |
| Cc1cc(C)c2c(c1)oc(c2C)C(=O)N(Cc1ccc(cc1)N(C)C)C[C@@H]1CCCO1 | 0 |
| O=C(N(c1ccccn1)Cc1ccc(cc1)N(C)C)[C@H](Oc1cc(C)c(c(c1)C)Cl)C | 0 |
| COCc1cccc(c1)Br | 0 |
| Brc1ccc(c(c1)Cl)N(C)C | 0 |
| O=C(c1sc(c(c1)Br)Br)Nc1ccccc1C(F)(F)F | 0 |
| N#Cc1ccccc1NC(=O)C[C@H](C(=O)[O-])[NH2+]C[C@@H]1CCCO1 | 0 |
| COc1ccc(cc1Cl)NC(=O)C[C@H](C(=O)[O-])[NH2+]Cc1ccco1 | 0 |
| CCCNc1c(N/C(=N\c2ccc(cc2F)Br)/O)c(=O)oc2c1cccc2 | 0 |
| Cc1ccc(cc1)c1cn2c3n1CCCCc3c(c2)c1ccc2c(c1)CCCC2 | 0 |
| CCc1ccccc1N(C(=O)NC1CCCCC1)Cc1nnc2n1CCCCC2 | 0 |
| C1[C@@H]2C[C@@H]3C[C@H]1C[C@H](C2)O3 | 0 |
| CN1CCc2c(C1)c1cc(F)ccc1n2c1ccccc1 | 0 |
| O=C1N[C@@H](c2ccc(o2)c2ccccc2Cl)[C@@H]([C@](N1)(O)C(F)(F)F)C(=O)c1ccc(cc1)F | 0 |
| c1ccc(cc1)[C@@H]1OCc2c1cccc2 | 0 |
| Clc1ccc(cc1)c1cn2c3c1CCCCn3c(c2)c1ccc(cc1)Cl | 0 |
| CC(=O)Nc1nc(C)cc(n1)Cl | 0 |
| COc1ccc(ccc1=O)Br | 0 |
| [O-]C(=O)[C@H]1C[NH2+]C[C@H]1N | 0 |
| CN1c2ccccc2C(=CC1(C)C)C | 0 |
| CN1c2ccccc2[C@@H](CC1(C)C)C | 0 |
| C=CC#CCN1CCCC1 | 0 |
| Cn1c2ccccc2c2c1[C@@]1(CCCN1CC2)c1ccccc1 | 0 |
| CN[C@](CC(C)C)(C#N)C | 0 |
| CCNc1ccc(cc1N)Br | 0 |
| Clc1ccc(c(c1)N)NC1CCCC1 | 0 |
| Cc1ccc(cc1)n1nnc2c1ccc(c2)N | 0 |
| COc1cccc2c1ncc1c2[NH2+]N(C1=O)[CH2-] | 0 |
| Nc1ccc(c2c1cccc2)N | 0 |
| Cc1cc2[nH]c3CCCc3c(=O)[nH]c2cc1C | 0 |
| c1ccnnc1C#N | 0 |
| O=c1ccc(n[nH]1)c1ccc2c(c1)cccc2 | 0 |
| Ic1cccc2c1OCC2 | 0 |
| CCCn1c(CN2CCC(CC2)C)nc2c1ccc(c2)NC(=O)c1ccc(cc1)OC | 0 |
| O=c1cnn(c(=O)[nH]1)c1ccccc1 | 0 |
| O=C1COc2c(N1)cc(c(c2)NCc1c(C)cc(c(c1C)C(=O)C)C)Cl | 0 |
| C=C[C@@]1(C)CC(=O)[C@]2([C@@](O1)(C)[C@@H](O)[C@H]([C@@H]1[C@]2(C)[C@@H](O)CCC1(C)C)O)O | 0 |
| C[C@@H]1O[C@H](C)CN(C1)C1CCCCCCCCCCC1 | 0 |
| Fc1ccc2c(c1)c1CN(CCc1[nH]2)C(=O)c1ccccc1C(=O)c1ccccc1 | 0 |
| O=C(NCc1ccc(s1)Cl)CCc1c(C)[nH]c(=O)[nH]c1=O | 0 |
| COc1ccc(cc1)c1nn(cc1[C@@H]1NC(=Nc2n1c1ccccc1n2)N)c1ccccc1 | 0 |
| O=C(c1ccco1)N1CCC(CC1)NS(=O)(=O)c1ccc2c(c1)[nH]c(=O)c(=O)[nH]2 | 0 |
| O=c1[nH]c2cc(ccc2[nH]c1=O)S(=O)(=O)NCc1ccc(nc1)n1cccn1 | 0 |
| COc1ccccc1[C@H](N(C(=O)C1CCN(CC1)c1ccc(cn1)C(F)(F)F)C)C | 0 |
| O=C(c1ccc(c(c1)F)C)N[C@H](c1ccc2c(c1)[nH]cn2)c1ccccc1 | 0 |
| NC(=O)NCc1ccc(cc1)C(=O)OCc1nc(N)nc(n1)N(C)C | 0 |
| COc1ccc(cc1)N1C(=O)CC[C@@H]([C@@H]1c1ccccc1OC)C(=O)Nc1ccc(cc1)n1ncnc1 | 0 |
| COc1ccc(c2c1N[C@@]1(CC[N@@H+](CC1)Cc1cccc(c1)C#N)[N-]S2(=O)=O)C | 0 |
| Fc1cccc(c1)NC(=O)N1CCC(CC1)Oc1ccc(cc1)c1ccc(nc1)N(C)C | 0 |
| CC1=CC2=NC(=CC3=NC(=CC(=N1)[C@@H]23)C)C | 0 |
| O=C(N(CC(=O)N1CCN(CC1)c1ncc(cc1Cl)C(F)(F)F)C)Cc1ccccc1Cl | 0 |
| O=C1COc2c(N1)cc(cc2)NC(=O)c1cc(nc2c1cccc2)C1CC1 | 0 |
| COc1ccc(c(c1)OC)N1[C@H](Nc2c(C1=O)cccc2)c1cc(Cl)c(c(c1)OC)O | 0 |
| O=C1CCc2c(N1)ccc(c2)C(=O)CSc1nnc(n1CCN1CCOCC1)c1ccc(cc1)F | 0 |
| C1CC(O1)c1ccccc1 | 0 |
| Oc1ccccc1N1CCN(CC1)Cc1[nH]c(=O)c2c(n1)c1ccccc1o2 | 0 |
| O1CCc2c(CC1)cccc2 | 0 |
| COc1cccc(c1OCC(=O)Nc1ccc(c(c1)S(=O)(=O)N1CCOCC1)C)C(=O)N | 0 |
| C[C@@H]1CCC[C@H](C1)N1CCOCC1 | 0 |
| O=C(N[C@H](c1n[nH]c(=O)[nH]1)C)Nc1nnc(s1)CCc1ccccc1 | 0 |
| Cc1ccc(cc1)C[N@@H+]1CCC[C@@H](C1)N1CCCC1 | 0 |
| Cc1ccc(cc1CN1CCOCC1)C | 0 |
| CN1CC[NH+](CC1)Cc1cccc(c1)C(F)(F)F | 0 |
| O=C(Nc1ccc(cc1)S(=O)(=O)N)CCc1c(C)[nH]c(=O)n(c1=O)C | 0 |
| COc1ccc(c(c1)CN(C[C@H]1CC[N@@H+](CC1)C)CCc1ccccc1)F | 0 |
| NC(=O)CSc1nnc2n1c1cc(ccc1c(=O)n2Cc1ccccc1)C(=O)NC(C)C | 0 |
| O=C(Cn1cnc2c(c1=O)[nH]c1c2cc(cc1)C)NCc1ccc(cc1)Br | 0 |
| CC(=O)N1c2cc(ccc2CCc2c1cccc2)NC(=O)[C@@]12C[C@H]3C[C@@H](C1)C[C@@](C2)(C3)Cl | 0 |
| CCCCn1c(=O)[nH]c(=O)c(c1N)N(C(=O)Cn1cnc2c(c1=O)cc(cc2)F)Cc1ccco1 | 0 |
| Brc1ccc2c(c1)cc([nH]2)C(=O)NC1CCN(CC1)C(=O)c1cocc1 | 0 |
| CCN(c1c(=O)[nH]c(=O)n(c1N)Cc1ccccc1)C(=O)CCc1onc(n1)c1ccco1 | 0 |
| CN1CCN(CC1)[C@@H]1CCC[C@@H]1O | 0 |
| Brc1cccc(c1)c1c(Cl)c(nn1c1ccc(cc1)F)c1cccc(c1)Br | 0 |
| Brc1ccc(s1)CN1CCc2c(C1)[nH]c1c2cccc1 | 0 |
| Cc1cc(C)c(cc1CNCCc1c[nH]c2c1cccc2)C | 0 |
| Fc1ccc(cc1)COc1coc(cc1=O)CN1CCN(CC1)C(=O)c1c(F)c(F)cc(c1F)F | 0 |
| Cc1cc(COc2coc(cc2=O)CN2CCN(CC2)C(=O)CCc2ccccc2)cc(c1)C | 0 |
| Clc1cccc(c1Cl)n1ccc2c(c1=O)cnc1n2c2ccccc2n1 | 0 |
| Brc1ccc(cc1)c1nn(c(c1C)c1ccc(cc1)Br)C1CCCC1 | 0 |
| Cc1ccc(cc1)c1c(C)sc2c1c(=O)[nH]c(n2)CSc1ccc(cn1)C(F)(F)F | 0 |
| C[C@@H]1CCc2c(C1)sc1c2c(=O)[nH]c(n1)[C@H](Sc1ccc(cn1)C(F)(F)F)C | 0 |
| O=C(NC(c1ccccc1)c1ccccc1)Nc1ccc2c(c1)NC(=O)C(CO2)(C)C | 0 |
| O=C(Nc1ccc2c(c1)c(=O)n(c(n2)C)c1ccc(cc1)Cl)Nc1ccc2c(c1)OCO2 | 0 |
| COc1ccc(cc1)n1c(C)nc2c(c1=O)cc(cc2)NC(=O)Nc1ccc(cc1C)C | 0 |
| c1ccc(cc1)/C=C\CN1CC[N@@H+](CC1)CCOC(c1ccccc1)c1ccccc1 | 0 |
| CCOc1cccc2c1NC(=O)C2=O | 0 |
| Clc1ccc(c(c1)C)N1CCCC1 | 0 |
| CN(c1ccc(cc1)C1=NCCC1)C | 0 |
| COc1cc(cc(c1OC)OC)[C@H]1NCCN(C1)C(=O)OC(C)(C)C | 0 |
| C=Cc1ccc2c(c1)c1ccccc1n2C | 0 |
| O=C(c1cccnc1Nc1ccc(cc1)C(C)C)Nc1ccccc1C | 0 |
| O=C(c1cccnc1Nc1ccc(c(c1)Cl)F)Nc1ccccc1C | 0 |
| COc1ccccc1CN[C@@H](C(=O)c1c[nH]c2c1cccc2)c1ccccc1 | 0 |
| C[C@@H]1O[C@H]([C@@H]([C@@H]1O)O)O | 0 |
| COc1cc(C)cc(c1)Br | 0 |
| [O-]C(=O)[C@H]1C[C@@H](C[NH2+]1)N | 0 |
| COc1ccccc1c1sc2ccccc2c(=O)c1I | 0 |
| Ic1c(oc2c(c1=O)cccc2)c1cscc1 | 0 |
| C1CCN(C1)c1cccc2c1cccc2 | 0 |
| COC1=CCCc2c1cccc2 | 0 |
| CCO[C@@H]1C=CC=Cc2c1cccc2 | 0 |
| Fc1ccc(cc1)[C@@H]1OCc2c1ccc(c2)Br | 0 |
| NCc1nc2c([nH]1)c(C)c(cc2)C | 0 |
| Fc1ccc(cc1)CNC(=O)N1CCC2(CC1)Nc1ccccc1n1c2ccc1 | 0 |
| O=C(N1CCC2(CC1)Nc1ccccc1n1c2ccc1)Nc1ccccc1C | 0 |
| COc1cc(ccc1OC)[C@@H]1N(Cc2c(n3c1ccc3)cccc2)C(=O)NC1CCCC1 | 0 |
| CCOC(=O)c1nn2c(c1)C(=O)N([C@@](C2)(C)C(=O)NCc1ccc(cc1)Cl)Cc1ccc(o1)C | 0 |
| Fc1ccc(cc1)c1nc2n(c1NC(=O)c1ccc3c(c1)OCCCO3)ccc(c2)C | 0 |
| Ic1cccc(c1)OS(=O)(=O)/C=C\c1ccccc1 | 0 |
| Fc1ccc(cc1)S(=O)(=O)Oc1cccc(c1)I | 0 |
| CCC(=O)N1CCC[C@H]1c1nc2c(n1CCOc1ccc(cc1)Cl)cccc2 | 0 |
| O=C1CCn2c(N1)cc(n2)c1ccccc1C | 0 |
| Fc1ccc(cc1)N1C(=O)c2nc3c(n2C[C@@]1(C)C(=O)NC1CCCCCC1)cccc3 | 0 |
| [O-]C(=O)c1cc([nH+]c2c1cccc2)N1CCc2c1cccc2 | 0 |
| COc1ccc2c(c1)ccc(c2)[C@@H](C(=O)N(Cc1ccccc1N1CCOCC1)C)C | 0 |
| O=C(N1CCN(CC1)C(=O)c1ccco1)CCc1csc(n1)NC(=O)c1ccc[nH]c1=O | 0 |
| COc1cc(ccc1OC)c1noc(n1)NCCN1CCCc2c1cccc2 | 0 |
| CSc1ccc(cc1)CNC(=O)CNC(=O)[C@@]1(C)CN(CC(=O)N1C)S(=O)(=O)C | 0 |
| Brc1ccc2c(c1)CCCO2 | 0 |
| [O-]C(=O)C1([NH3+])CCNCC1 | 0 |
| CNC(=O)[C@@H]1CC[C@@H](CC1)Cn1c(=O)c2sccc2n(c1=O)CC(=O)Nc1cccc(c1)C(=O)C | 0 |
| N#CC1(CCCC1)c1ccc(cc1)NC(=O)c1cc(I)ccc1Cl | 0 |
| CCc1ccc(cc1)NC(=O)C1=C(C)Nc2n([C@@H]1c1ccc(cc1)O)c1ccccc1n2 | 0 |
| COc1cccc(c1O)[C@@H]1Nc2ccccc2n2c1c1n(C)c(=O)n(c(=O)c1c2c1ccccc1)C | 0 |
| COc1ccccc1NC(=O)c1cccc(c1)N1C(=O)NC2C[C@]1(C)Oc1c2cc(cc1)Cl | 0 |
| O=C(N1CCc2c(C1)cccc2)c1cccc(c1)N1C(=O)NC2C[C@]1(C)Oc1c2cccc1 | 0 |
| COc1ccccc1CNC(=O)c1cccc(c1)N1C(=O)NC2C[C@@]1(C)Oc1c2cccc1 | 0 |
| Oc1cccc(c1)[C@H]1C(=C(C)Nc2n1c1ccccc1n2)C(=O)Nc1ccc(cc1)Cl | 0 |
| CCOc1ccccc1[C@@H]1Nc2c(C)cc(cc2[C@H]2[C@H]1CC=C2)C | 0 |
| COc1ccc(cc1)NC(=O)C1CCN(CC1)S(=O)(=O)c1c(C)[nH]c(=O)[nH]c1=O | 0 |
| Cc1cccc(c1)[C@@H]1Nc2ccc(cc2[C@H]2[C@H]1CC=C2)Oc1ccccc1 | 0 |
| CC(Oc1cccc2c1N[C@@H]([C@@H]1[C@H]2C=CC1)c1cccc(c1Cl)Cl)C | 0 |
| CCOc1ccc2c(c1)[C@H]1C=CC[C@H]1[C@H](N2)c1ccc2c(c1)cccc2 | 0 |
| Clc1ccccc1[C@H]1Nc2ccc(cc2[C@H]2[C@H]1CC=C2)Oc1ccccc1 | 0 |
| COc1cccc(c1O)[C@@H]1Nc2c([C@H]3[C@H]1CC=C3)cccc2C(=O)N(C)C | 0 |
| COc1cc(ccc1O)[C@@H]1Nc2c([C@H]3[C@H]1CC=C3)cccc2C(=O)N1CCCCC1 | 0 |
| CC(Oc1cccc2c1N[C@H](c1ccc(cc1)C)[C@@H]1[C@H]2C=CC1)C | 0 |
| CN(c1ccc(cc1)[C@H]1Nc2c(cccc2[C@H]2[C@@H]1CC=C2)C(C)C)C | 0 |
| Clc1ccc(c2c1N[C@@H](c1ccc(cc1)N(C)C)[C@H]1[C@H]2C=CC1)Cl | 0 |
| CCOc1cccc2c1N[C@H](c1ccc(cc1)Br)[C@@H]1[C@H]2C=CC1 | 0 |
| C[C@@H]1CC(=O)c2c(C1)n(nc2Nc1ccc(c(c1)Cl)Cl)c1ccccc1 | 0 |
| Brc1ccc2c(c1)sc(n2)N(C(=O)Cc1ccc(cc1)F)CCCN1CCOCC1 | 0 |
| CCC(Nc1ccc(cc1F)N)(C)C | 0 |
| CN1CCN(CC1)c1cc(C)c2c(n1)ccc(c2)NC(=O)Nc1ccc2c(c1)OCCO2 | 0 |
| O=C(Cn1ncc2c(c1=O)[nH]c1c2cc(cc1)C)Nc1cccc(c1)C(F)(F)F | 0 |
| Brc1ccc2c(c1)c(C(=O)[O-])c1c([nH+]2)[C@H](C)CCC1 | 0 |
| COc1ccc(cc1)[C@@H]1N(CCc2c1[nH]c1c2cc(cc1)OC)C(=O)Nc1ccc2c(c1)OCCO2 | 0 |
| COc1ccc(cc1)N1[C@@H](Nc2ccc(cc2)OC(F)(F)F)c2c(C1=O)cccn2 | 0 |
| [O-]c1n[nH]c([nH+]1)CCNC(=O)Nc1nnc(s1)[C@@H](CCC)C | 0 |
| O=C(Nc1ccc(cc1)C(=O)N1CCCC1)COc1ccc2c(c1)OCO2 | 0 |
| Cn1nc(c(c1)[C@@H]1C(=C(C)Nc2n1nc(n2)C(F)(F)F)C(=O)Nc1ccccc1C)C | 0 |
| COC(=O)c1cc(nc2c1c(=O)n(CC(=O)Nc1cccc(c1)C)c(=O)n2c1ccccc1)C1CC1 | 0 |
| Cc1ccc2c(c1)c(C(=O)[O-])c1c([nH+]2)CC[C@H](C1)C(C)(C)C | 0 |
| CCOC(=O)c1cccc(c1)c1ccc(o1)/C=C/1\C(=O)N[C@H]2N(C1=N)N/C(=C/C(=O)N1CCCC1)/S2 | 0 |
| COC(=O)c1cc(C)nc2c1c(=O)n(CC(=O)Nc1cccc(c1C)Cl)c(=O)n2c1ccccc1 | 0 |
| C[C@H]1O[C@H](C)CN(C1)C(=O)c1ccc(=O)[nH]c1 | 0 |
| CN1CCN(CC1)C(=O)c1ccc(=O)[nH]c1 | 0 |
| O[C@H](c1ccc2c(c1)[nH]cc2)CN1CCOCC1 | 0 |
| [O-]C(=O)[C@@]1([NH3+])CNCC1 | 0 |
| C#CCN1CCC(CC1)C(F)(F)F | 0 |
| Nc1cccc(c1)c1ncc[nH]1 | 0 |
| O=C1NCCN1c1ccc(c(c1)N)Cl | 0 |
| OCC[C@H]1CN(CCN1C)Cc1cc2c([nH]1)cccc2 | 0 |
| Oc1cccc(c1)NC1CCCCC1 | 0 |
| C[C@H](C#N)NCCC | 0 |
| N#C[C@@H](c1cccc(c1)Br)N | 0 |
| Nc1ccc(c(c1)F)NC(C)(C)C | 0 |
| CCc1ccccc1NCC(F)(F)F | 0 |
| CCc1ccc(cc1)NCC(F)(F)F | 0 |
| Cc1ccc(cc1C)NCC(F)(F)F | 0 |
| Clc1ncnc2c1sc1c2c2CCCCc2c(n1)N1CCCCC1 | 0 |
| CCOc1ccc2c(c1)cc(c(=O)[nH]2)[C@H](c1nnnn1C(C)(C)C)N1CCN(CC1)c1ccc(cc1)OC | 0 |
| COCCn1nnnc1[C@@H](c1cc2cc(OCC)ccc2[nH]c1=O)N1CCN(CC1)c1cccc(c1C)C | 0 |
| Fc1ccc2c(c1)N(C)C(=O)C2=O | 0 |
| CCCNc1ccccc1C | 0 |
| N#C[C@@H]1C(=O)Nc2c([C@H]1c1cccc3c1cccc3)c(=O)[nH]c(n2)N1CCCC1 | 0 |
| CCNc1ccc(cc1C)F | 0 |
| Fc1ccc(c(c1)C)NCc1cccc(c1)O | 0 |
| O=C(c1cnn(c1c1ccco1)c1ncc2c(n1)c1ccccc1CC2)NCC(N1CCOCC1)(C)C | 0 |
| Oc1cccc(c1)CNc1cc(C)cc(c1)C | 0 |
| CCCNc1ccccc1F | 0 |
| Fc1ccccc1NC1CCCC1 | 0 |
| N#C[C@H](c1cccnc1)N1CCCC1 | 0 |
| CC(=C)C#CCN1CCCCC1 | 0 |
| CCCNc1cccc(c1)Cl | 0 |
| CC(CNc1ccc(c(c1)F)F)C | 0 |
| COc1cc(ccc1OCc1ccc(cc1)Br)[C@@H]1NC(=N)Nc2n1c1ccccc1n2 | 0 |
| Cc1cn(c2c1c(Cl)c1ccccc1n2)c1ccccc1 | 0 |
| CC(=O)c1c(=O)oc2c1ccccc2 | 0 |
| [NH3+]CCCCCCNC(=O)c1c(=O)[nH]c2c(c1[O-])CCCC2 | 0 |
| Clc1ccc(cc1)[C@@H]1OC(c2c1cccc2)(C)C | 0 |
| CCC1=Cn2c3c(C1)cccc3c1c2cccc1 | 0 |
| c1ccc(cc1)CCCN1CC[NH+](CC1)Cc1cccc(c1)Oc1ccccc1 | 0 |
| FC(c1ccccc1C[N@H+]1CC[C@@H](CC1)N1CCCC1)(F)F | 0 |
| C[C@@H]1C[C@@H](C)CN(C1)[C@H]1CC[N@H+](CC1)Cc1cccc(c1)C | 0 |
| c1ccc(cc1)CN([C@H]1CC[N@@H+](CC1)Cc1c[nH]c2c1cccc2)CCc1ccccc1 | 0 |
| C1=C[C@H]2[C@@H](C=C1)[N-]C1=c3c(=[NH+]C1=N2)cccc3 | 0 |
| CCNc1cccc(c1)C#C | 0 |
| C#CCN[C@@H]1CCCc2c1cccc2 | 0 |
| CO/C/1=C/CCCCCCCCCC1 | 0 |
| O=C(N=c1c2ccccc2[nH]c2c1cccc2)CSc1nc2c(s1)cccc2 | 0 |
| O=C1NCCN[C@@]1(c1c[nH]c2c1cccc2)C(F)(F)F | 0 |
| Clc1ccc(cc1)n1c([O-])nc(=O)c2c1[NH+]=C(C)NC2(C(F)(F)F)C(F)(F)F | 0 |
| Cc1ccc2c(c1)c(cc([nH+]2)[C@@]12C[C@H]3C[C@@H](C1)C[C@@H](C2)C3)C(=O)[O-] | 0 |
| O=C1OCC[C@H]1S(=O)(=O)c1ccc(c2c1cccc2)Br | 0 |
| CSc1cccc(c1)Nc1nc(SC)nc(c1C#N)c1ccc(cc1)Cl | 0 |
| N#Cc1c(SCc2ccc(cc2)Cl)nc(c(c1c1ccc(cc1)Br)C#N)N | 0 |
| N#Cc1c(SCc2ccc(cc2)C(C)(C)C)nc(c(c1c1ccccc1)C#N)N | 0 |
| Clc1cccc(c1)N(C(C)(C)C)C | 0 |
| C#CC(N1C[C@H](C)C(=O)C[C@H]1C)(C)C | 0 |
| Brc1cccc(c1)CNCCc1c[nH]c2c1cccc2 | 0 |
| CN[C@]1(Oc2ccccc2C(=C1C)C#Cc1ccc(cc1)C)c1ccccc1 | 0 |
| Cc1ccc(cc1)CN1CC[NH+](CC1)C[C@H]1C[C@@H]2C[C@H]1C=C2 | 0 |
| CC(Nc1ccccc1Cl)C | 0 |
| CC(Nc1cccc(c1)Cl)C | 0 |
| CCNc1ccc(cc1C)C | 0 |
| CC(C#CCN1CCOCC1)(O)C | 0 |
| COc1ccc(cc1)NC(=O)c1cn(nc1CC1CCN(CC1)C(=O)C)C | 0 |
| FC(Oc1ccc(cc1)Cl)F | 0 |
| Clc1ccccc1CNCCc1c[nH]c2c1cccc2 | 0 |
| O=C1NCCCN1c1ccc(cc1)N | 0 |
| COc1ccc(cc1)C#Cc1c2ccccc2c(c2c1cc(Cl)cc2)C#Cc1ccc(cc1)OC | 0 |
| FC(c1ccccc1CNCCc1c[nH]c2c1cccc2)(F)F | 0 |
| Cc1ccc(cc1)N1[NH2+]C(=C(C1=O)CC(=O)N1CC[C@@](C1)(O)c1ccc(cc1)C)C | 0 |
| O=C1Nc2c([C@H]1CCC(=O)N1CC[C@@H](CC1)c1nc3c([nH]1)cc(cc3)C)cccc2 | 0 |
| C[C@@H]1CCC[C@H](N1CC#C)C | 0 |
| COc1cc(ccc1[O-])[C@@H]1[NH2+]CCc2c1cc(O)c(c2)O | 0 |
| COc1ccc(cc1)[C@@H]1N(CCc2c1nc[nH]2)C(=O)Cc1nc2c([nH]1)ccc(c2)Cl | 0 |
| [O-]c1c(ccc2c1nccc2)C[NH+]1CCN(CC1)Cc1ccc2c(c1O)nccc2 | 0 |
| c1ccc(cc1)CC[N@H+]1CC[C@@H](CC1)N(Cc1ccccc1)CCc1ccccc1 | 0 |
| c1ccc(cc1)/C=C/CN1CC[N@@H+](CC1)C1C[C@@H]2C[C@H]1CC2 | 0 |
| Clc1ccc(cc1)CN1CCOCC1 | 0 |
| C#CC(N1CCC(=O)[C@@H]2[C@@H]1CCC2)(C)C | 0 |
| Brc1ccc2c(c1)c(cc([nH+]2)[C@@]12C[C@H]3C[C@@H](C1)C[C@@H](C2)C3)C(=O)[O-] | 0 |
| O=C1CC[C@@]2(N1)CCCN([C@@H]2c1ccc(cc1)F)C(=O)c1ccc2c(c1)nc([nH]2)C | 0 |
| O=C(c1cc(C)c([nH]c1=O)C)N1Cc2c(C1)c(=O)n1c([nH]2)c(c2ccccc2)c(n1)C | 0 |
| Cc1ccccc1C[N@@H+]1CCN(CC1)C/C=C\c1ccccc1 | 0 |
| CC[NH+]1CCN(CC1)Cc1cccc(c1)Br | 0 |
| CCCn1ncc(c1C)CN([C@@H]1COC[C@H]1c1ccc(cc1)F)Cc1ccc(cc1)OC | 0 |
| CC(CNCC(F)(F)F)C | 0 |
| O=C(N1CCNCC1)Cc1c[nH]c2c1cccc2 | 0 |
| C[C@@H]1O[C@H](C)CN(C1)c1nc2C[N@@H+](CCc2c(n1)[O-])Cc1ccccc1O | 0 |
| Fc1ccc(cc1)CNCC(F)(F)F | 0 |
| O=C(C(C)(C)C)N1CCC(=O)CC1 | 0 |
| Nc1ccc2c(c1)nc([nH]2)c1cccc(c1)F | 0 |
| O=C(c1ccc(s1)C)N1CCNCC1 | 0 |
| O=C1CC[C@H](N1)C(=O)N1CC[C@@H](CC1)c1nc2ccccc2cc1N | 0 |
| OC[C@@H]1CN(C[C@]21CCc1c(C(=O)N2)cccc1)S(=O)(=O)c1ccc(cc1)F | 0 |
| c1ccc2c(c1)C(N1CCN(CC1)C1c3ccccc3CCc3c1cccc3)c1ccccc1CC2 | 0 |
| Fc1ccc(cc1)CN1CC[NH+](CC1)Cc1ccccc1C | 0 |
| CN1CCN(CC1)CC#CC(O)(C)C | 0 |

**Table S4.** Structures of 1042 hepatotoxic compounds (in SMILE format) in top 10 herbs predicted by consensus model 3 (C-3).

**Name	Compounds**	mol

| Chaihu | MOL000057 | O(C(=O)c1ccccc1C(OCC(C)C)=O)CC(C)C |
| --- | --- | --- |
| Chaihu | MOL000069 | O=C([O-])CCCCCCCCCCCCCCC |
| Chaihu | MOL000103 | Oc1ccc(cc1)C(=O)[O-] |
| Chaihu | MOL000114 | O(C)c1cc(ccc1O)C(=O)[O-] |
| Chaihu | MOL000131 | O=C([O-])CCCCCCC\C=C/C\C=C/CCCCC |
| Chaihu | MOL000211 | OC1CCC2(C(CCC3(C2CCC2C4C(CCC23C)(CCC4C(C)=C)C(=O)[O-])C)C1(C)C)C |
| Chaihu | MOL000219 | O=C([O-])c1ccccc1 |
| Chaihu | MOL000223 | Oc1cc(ccc1O)\C=C\C(=O)[O-] |
| Chaihu | MOL000256 | O(C(COC(=O)CCCCCCC\C=C/CCCCCCCC)COC(=O)CCCCCCC\C=C/CCCCCCCC)C(=O)CCCCCCC\C=C/CCCCCCCC |
| Chaihu | MOL000263 | OC1CCC2(C(CCC3(C2CC=C2C4CC(CCC4(CCC23C)C(=O)[O-])(C)C)C)C1(C)C)C |
| Chaihu | MOL000305 | O=C([O-])CCCCCCCCCCC |
| Chaihu | MOL000346 | O=C([O-])CCC(=O)[O-] |
| Chaihu | MOL000360 | O(C)c1cc(ccc1O)\C=C\C(=O)[O-] |
| Chaihu | MOL000392 | O1C=C(C(=O)c2c1cc(O)cc2)c1ccc(OC)cc1 |
| Chaihu | MOL000394 | OCC[N+](C)(C)C |
| Chaihu | MOL000476 | O(C)c1cc(O)c2c(c1)C(=O)c1c(C2=O)c(O)cc(c1)C |
| Chaihu | MOL000511 | OC1CCC2(C(CCC3(C2CC=C2C4C(C)C(CCC4(CCC23C)C(=O)[O-])C)C)C1(C)C)C |
| Chaihu | MOL000515 | O=C([O-])CCCCCCCCCCCCCCCCCCCCCCCCCCCCC |
| Chaihu | MOL000550 | OC(C(O)C(O)CO)C(O)CO |
| Chaihu | MOL000635 | O(C)c1cc(ccc1O)C=O |
| Chaihu | MOL000675 | O=C([O-])CCCCCCC\C=C/CCCCCCCC |
| Chaihu | MOL000676 | O(C(=O)c1ccccc1C(OCCCC)=O)CCCC |
| Chaihu | MOL000708 | O=Cc1ccccc1 |
| Chaihu | MOL000714 | O=CCc1ccccc1 |
| Chaihu | MOL000775 | O(C(=O)C)CC |
| Chaihu | MOL000860 | O=C([O-])CCCCCCCCCCCCCCCCC |
| Chaihu | MOL000874 | O(C)c1cc(O)c(cc1)C(=O)C |
| Chaihu | MOL000879 | O(C(=O)CCCCCCCCCCCCCCC)C |
| Chaihu | MOL001335 | OCc1ccccc1 |
| Chaihu | MOL001393 | O=C([O-])CCCCCCCCCCCCC |
| Chaihu | MOL001452 | Oc1cc(ccc1O)C=O |
| Chaihu | MOL001456 | OC(CC(=O)[O-])(CC(=O)[O-])C(=O)[O-] |
| Chaihu | MOL001494 | O(C(=O)CCCCCCC\C=C/C\C=C/CCCCC)CC |
| Chaihu | MOL001641 | O(C(=O)CCCCCCC\C=C/C\C=C/CCCCC)C |
| Chaihu | MOL001689 | O1c2c(C(=O)C=C1c1ccc(OC)cc1)c(O)cc(O)c2 |
| Chaihu | MOL001729 | Oc1c2c(cc(c1)C)C(=O)c1c(C2=O)c(O)ccc1 |
| Chaihu | MOL001794 | O=C([O-])\C=C/C(=O)[O-] |
| Chaihu | MOL001807 | O(C)c1cc(cc(OC)c1O)C(=O)[O-] |
| Chaihu | MOL001859 | O=C[O-] |
| Chaihu | MOL001880 | O=C([O-])C(=O)[O-] |
| Chaihu | MOL001940 | OC(C=C)C#CC#CC(O)\C=C/CCCCCCC |
| Chaihu | MOL001945 | O1c2c(C=CC1=O)c(OC)c1c(occ1)c2 |
| Chaihu | MOL001955 | O(C(=O)\C=C\c1cc(O)c(O)cc1)C1CC(O)(CC(O)C1O)C(=O)[O-] |
| Chaihu | MOL002092 | Oc1ccc(cc1C(C)(C)C)C(C)(C)C |
| Chaihu | MOL002299 | OC(CC(=O)[O-])C(=O)[O-] |
| Chaihu | MOL002558 | O1c2cc(O)ccc2C=CC1=O |
| Chaihu | MOL002691 | O=C([O-])CC(C)C |
| Chaihu | MOL002850 | Oc1c(cc(cc1C(C)(C)C)C)C(C)(C)C |
| Chaihu | MOL002883 | O(C(=O)CCCCCCC\C=C/CCCCCCCC)CC |
| Chaihu | MOL003050 | O=C([O-])CCCCCCCC |
| Chaihu | MOL003205 | O1C2CC(CC3C4=CCC5C(CCC6C(C)(C)C(O)CCC56C)(C)C4(CCC23C)C)(C)C1=O |
| Chaihu | MOL003206 | O1C2C(CCC(O)(C34OC3C3OC3(C24)C)C)C(=C)C1=O |
| Chaihu | MOL003207 | O=C1NCCCN(CCCC[NH2+]C(C1)c1ccccc1)C(=O)\C=C\c1ccccc1 |
| Chaihu | MOL003208 | o1cc(cc1)C(=O)N1CCCC[NH2+]C(CC(=O)NCCC1)c1ccccc1 |
| Chaihu | MOL003209 | O=C1NCCCN(CCCC[NH2+]C(C1)c1ccccc1)C(=O)\C=C/c1ccccc1 |
| Chaihu | MOL003210 | O1C23C(C)(C(OC(=O)c4cccnc4)C(OC(=O)C)C(C1(C)C)C2OC(=O)c1ccoc1)C(OC(=O)C)CCC3C |
| Chaihu | MOL003212 | O(C)c1c(C(C)=C)c(O)c2c(c1O)C1(C(CC2=O)C(=C)C(CC1)C)C |
| Chaihu | MOL003214 | O1CC(C2C3C1OCC3(O)C=C2)C(OC)=O |
| Chaihu | MOL003215 | O=C1NC(=O)NC1NC(=O)[O-] |
| Chaihu | MOL003217 | O1c2c(C(=O)CC1c1ccc(O)cc1)c(OC)cc(O)c2C\C=C(\C)/C |
| Chaihu | MOL003220 | O1C2\C=C(\C)/C(O)C\C=C(\CC(OC(=O)\C(=C\C)\C)C2C(=C)C1=O)/C |
| Chaihu | MOL003221 | OC1CCC2(C(CCC3(C2CC=C2C4C(CCC23C)(C)C(O)CC(C(OC)=O)C4C)C)C1(C)C)C |
| Chaihu | MOL003223 | OC=1C(=O)C=C2C(=CC=C3C4(CCC5(C(CC(C)C(=O)C5)C4(CCC23C)C)C)C)C=1C |
| Chaihu | MOL003224 | O1C2C1(C(C)C)C(O)C1=C(C3(C(CC1=O)C1=C(CC3)C(OC1)=O)C)C2O |
| Chaihu | MOL003226 | O=C1CCC2(C(CC=C3C2CCC2(C)C3(CCC2C(CC\C=C(/CO)\C)C=O)C)C1(C)C)C |
| Chaihu | MOL003229 | Oc1c2c(ccc1C(C)C)C1(C(CC2)C(C)=C(CC1)C(=O)[O-])C |
| Chaihu | MOL003230 | OC1CCC2(C(CCC3(C2CC=C2C4CC(CCC4(CCC23C)C)(C(OC)=O)CO)C)C1(C)C)C |
| Chaihu | MOL003231 | O(C)c1c2c(ccc1C(C)C)C1(C(CC2)C(C)=C(CC1)C(=O)[O-])C |
| Chaihu | MOL003232 | O1C23CC(CC(OC(=O)c4ccccc4)C2(C)C(OC(=O)c2ccccc2)CCC3(O)C)C1(C)C |
| Chaihu | MOL003235 | O1C23C(COC(=O)C)(C(Oc4ccccc4)C(=O)C(C1(C)C)C2OC(=O)C)C(OC(=O)\C=C/c1ccccc1)CCC3(O)C |
| Chaihu | MOL003236 | O1C23C(COC(=O)C)(C(Oc4ccccc4)C(OC(=O)C)C(C1(C)C)C2OC(=O)C)C(OC(=O)\C=C/c1ccccc1)CCC3(O)C |
| Chaihu | MOL003240 | O1C23C(COC(=O)C)(C(OC(=O)c4ccccc4)C(OC(=O)C)C(C1(C)C)C2OC(=O)C)C(OC(=O)c1ccccc1)C(OC(=O)C)CC3(O)C |
| Chaihu | MOL003242 | O1C23C(COC(=O)C)(C(OC(=O)c4ccccc4)C(OC(=O)c4cccnc4)C(C1(C)C)C2OC(=O)C)C(OC(=O)\C=C/c1ccccc1)CCC3(O)C |
| Chaihu | MOL003243 | O1C2C3OC34C3(OC3CC3C5=C(CCC34C)C(OC5)=O)C(O)C12C(O)(C)C |
| Chaihu | MOL003244 | O1C2C3OC34C3(OC3CC3C5=C(CCC34C)C(OC5)=O)C(=O)C12C(C)C |
| Chaihu | MOL003245 | O(C)c1cc2c(CCC3C(C)=C(CCC23C)C(=O)[O-])c(O)c1C(C)C |
| Chaihu | MOL003246 | O1C2C1C[NH+]1C3C4(C(Nc5cc(O)ccc45)=C(CC23CC)C(OC)=O)CC1 |
| Chaihu | MOL003247 | O(C)c1c2c(C3(C(CC2)C(C)(C)C(=O)CC3)C)c(O)cc1C(C)C |
| Chaihu | MOL003248 | Oc1c2c(ccc1C(C)C)C1(C(CC2)C(C)(C)C(=O)CC1)C |
| Chaihu | MOL003249 | OC1C(C2CCC3(C(CC=C4C5C(O)(C)C(CCC5(CCC34C)C(=O)[O-])C)C2(CC1O)C)C)(C)C |
| Chaihu | MOL003250 | O1C23C(CCC(O)C2(C)C)C2(C(CC13)C1(CCC3(C(CC(CC3)(C(=O)[O-])C)C1(CC2)C)C)C)C |
| Chaihu | MOL003251 | OC1CCC2(C(CCC3(C2CC=C2C4CC(CC(O)C4(CCC23C)C)(C(=O)[O-])C)C)C1(C)C)C |
| Chaihu | MOL003252 | OC1CCC2(C(CCC3(C2CC=C2C4CC(CC(O)C4(CCC23C)C)(C(=O)[O-])C)C)C1(C)C)C |
| Chaihu | MOL003253 | OC1CCC2(C(CCC3(C2CC=C2C4C(CCC23C)(C)C(O)CC(C(=O)[O-])C4C)C)C1(C)C)C |
| Chaihu | MOL003254 | OC1CCC2(C(CCC3(C2CC=C2C4C5(CC(CC5=O)C23C)CCC(C4)(C)C)C)C1(C)C)C |
| Chaihu | MOL003255 | O1C2C(OC(=O)C)C(OC(=O)C)C3(C4(OC(C(C(OC(=O)C)C3OC(=O)C)C4OC(=O)C)(COC(=O)c3c(nccc3)CCC(OC(=O)c3ccccc3)(C)C1=O)C)C2(O)C)COC(=O)C |
| Chaihu | MOL003257 | O1C2C(OC(=O)C)C(OC(=O)C)C3(C4(OC(C(C(OC(=O)C)C3OC(=O)C)C4OC(=O)c3ccccc3)(COC(=O)c3c(nccc3)CCC(OC(=O)c3ccccc3)(C)C1=O)C)C2(O)C)COC(=O)C |
| Chaihu | MOL003258 | O1C2C(OC(=O)C)C(OC(=O)C)C3(C4(OC(C(C(OC(=O)C)C3OC(=O)C)C4OC(=O)C)(COC(=O)c3c(nccc3)CCC(OC(=O)c3occc3)(C)C1=O)C)C2(O)C)COC(=O)C |
| Chaihu | MOL003259 | O1C2C(OC(=O)C)C(OC(=O)C)C3(C4(OC(C(C(=O)C3OC(=O)C)C4OC(=O)C)(COC(=O)c3c(nccc3)CCC(O)(C)C1=O)C)C2(O)C)COC(=O)C |
| Chaihu | MOL003261 | O1C2C(OC(=O)c3ccccc3)C(OC(=O)C)C3(C4(OC(C(C(OC(=O)C)C3OC(=O)C)C4OC(=O)c3ccccc3)(COC(=O)c3c(ccnc3)C(C)C(O)(C)C1=O)C)C2(O)C)COC(=O)C |
| Chaihu | MOL003262 | O1C2C(OC(=O)C)C(O)C3(C4(OC(C(C(OC(=O)C)C3OC(=O)C)C4OC(=O)C)(COC(=O)c3c(ccnc3)C(C)C(C)C1=O)C)C2(O)C)COC(=O)C |
| Chaihu | MOL003263 | O=C([O-])c1cccnc1CC(CC(=O)[O-])C |
| Chaihu | MOL003264 | O1C2C(OC(=O)c3ccccc3)C(OC(=O)C)C3(C4(OC(C(C(OC(=O)C)C3OC(=O)C)C4OC(=O)C)(COC(=O)c3c(ccnc3)C(C)C(O)(C)C1=O)C)C2(O)C)COC(=O)C |
| Chaihu | MOL003265 | O1C2CC(CC3C4=CCC5C(CCC6C(C)(C)C(=O)CCC56C)(C)C4(CCC23C)C)(C)C1=O |
| Chaihu | MOL003267 | O1C2C(OC(=O)C)C(OC(=O)C)C3(C4(OC(C(C(OC(=O)C)C3OC(=O)C)C4OC(=O)C)(COC(=O)c3c(nccc3)CCC(C)C1=O)C)C2(O)C)COC(=O)C |
| Chaihu | MOL003268 | O1C2C(OC(=O)c3cccnc3)C(OC(=O)C)C3(C4(C5C(C5(OC(=O)C)C(C4)(COC(=O)c4c(nccc4)CCC(C)C1=O)C)C(OC(=O)C)C3OC(=O)C)C2(O)C)COC(=O)C |
| Chaihu | MOL003269 | O1CC=2C3CCC(=O)CC3(CCC=2C1=O)C |
| Chaihu | MOL003271 | O1C2C(OC(=O)c3ccoc3)C(OC(=O)C)C3(C4(OC(C(C(OC(=O)C)C3OC(=O)C)C4OC(=O)C)(COC(=O)c3c(nccc3)CCC(O)(C)C1=O)C)C2(O)C)COC(=O)C |
| Chaihu | MOL003272 | O1C23C4([NH+](CC12)CCCc1cc(OC)c(OC)cc14)CC(OC)C=C3 |
| Chaihu | MOL003273 | O1C2C(OC(=O)C)C(OC(=O)C)C3(C4(OC(C(C(OC(=O)C)C3OC(=O)C)C4OC(=O)C)(COC(=O)c3c(nccc3)CCC(C)C1=O)C)C2(O)C)COC(=O)C |
| Chaihu | MOL003275 | O1C2C(OC(=O)c3ccccc3)C(OC(=O)C)C3(C4(OC(C(C(OC(=O)C)C3OC(=O)C)C4OC(=O)C)(COC(=O)c3c(nccc3)CCC(O)(C)C1=O)C)C2(O)C)COC(=O)C |
| Chaihu | MOL003276 | O1C2C(OC(=O)c3ccccc3)C(OC(=O)C)C3(C4(OC(C(C(OC(=O)C)C3OC(=O)C)C4OC(=O)C)(COC(=O)c3c(nccc3)CCC(C)C1=O)C)C2(O)C)COC(=O)C |
| Chaihu | MOL003277 | O1C2C(OC(=O)c3ccoc3)C(OC(=O)C)C3(C4(OC(C(C(OC(=O)C)C3OC(=O)C)C4OC(=O)C)(COC(=O)c3c(nccc3)CCC(O)(C)C1=O)C)C2(O)C)COC(=O)C |
| Chaihu | MOL003278 | O1CC23C(CCC1(O)C2C)C1(C(CC3)C2(CCC3(C(CC(CC3)(C(=O)[O-])C)C2(CC1)C)C)C)C |
| Chaihu | MOL003279 | O1C2C3OC34C3(OC3CC3C5=C(CCC34C)C(OC5)=O)C(O)C12C(O)(C)C |
| Chaihu | MOL003280 | O1CC=2C3CC(=O)c4c(ccc(C(C)C)c4O)C3(CCC=2C1=O)C |
| Chaihu | MOL003483 | O(C(=O)CCCCCCC)CC |
| Chaihu | MOL003508 | OCCCCCCCCCC |
| Chaihu | MOL003590 | O1c2c(C=CC1=O)ccc1occc12 |
| Chaihu | MOL004281 | O(C(=O)C)CCCCCCCCCCCC |
| Chaihu | MOL004480 | O=C([O-])C |
| Chaihu | MOL005612 | O(C)c1cc(O)ccc1OC |
| Chaihu | MOL005783 | O(C(=O)C=C)CCO |
| Chaihu | MOL005788 | O(C(=O)CCCCCCCCC)CC |
| Chaihu | MOL005796 | OCCCCCCCCCCCCCCCCCCCCCCCC |
| Chaihu | MOL005797 | O=CCCCCCCCCCCCC |
| Chaihu | MOL005798 | O(C(=O)CCCCCCCCCC)CC |
| Chaihu | MOL005799 | O(CC)C(=O)\C=C\C |
| Chaihu | MOL005802 | O(C(=O)CCCCCCC\C=C/CCCCCCCC)CC(O)C |
| Chaihu | MOL005803 | O1C(C)C(C)C(O)c2n(CC1=O)c(cc2)C=O |
| Chaihu | MOL005804 | P(OCCCCCCCCCCCCCCCCCC)(OCCCCCCCCCCCCCCCCCC)(=O)[O-] |
| Chaihu | MOL005805 | c12c3c4ccc1cccc2ccc3ccc4 |
| Chaihu | MOL005808 | O(C(CCCCC)CC)C(=O)C |
| Chaihu | MOL005810 | O(C(=O)CCCCCCCCCCCCCCC)CCCCCCCCCCCC |
| Chaihu | MOL006735 | o1cccc1CO |
| Chaihu | MOL006985 | O1c2c-3c4C([NH+](CCc4cc2OC1)C)Cc1c-3cccc1OC |
| Chaihu | MOL008658 | OC(CCO)C |
| Chaihu | MOL009360 | O(C)C1CCC2(C(CCC3C2=CC(=O)C2(C4CCC(C(C)C)C4(CCC23C)C)C)C1(C)C)C |
| Chaihu | MOL009361 | O(C(=O)C)C12C3C(C45C6C(C[NH+](C4C1C6OC)CC)(COC)C(O)CC5OC)CC(C3OC(=O)c1ccccc1)C(OC)C2 |
| Chaihu | MOL009362 | Oc1cc(cc(O)c1C)\C=C/c1ccccc1 |
| Chaihu | MOL010687 | [NH3+]CC(CC)C |
| Chaihu | MOL010688 | O(C(=O)C(C)=C)CCCCCCCCCCCC |
| Chaihu | MOL010690 | O(C(=O)CCCCCCC\C=C/CCCCCCCC)CCCC |
| Chaihu | MOL010691 | O(C(=O)CCCCCCC\C=C\CCCCCC)CC |
| Chaihu | MOL010692 | O1C(C(=O)[O-])C(OC2OC(CO)C(O)CC2NC(=O)C)C(O)C(O)C1O |
| Chaihu | MOL010695 | O(C(CCOC(=O)C)C)C(=O)C |
| Chaihu | MOL011169 | O1OC23C4CCC(C(\C=C\C(C(C)C)C)C)C4(CCC2C2(C1(CC(O)CC2)C=C3)C)C |
| Chaihu | MOL011564 | OC(C(=O)C)C |
| Chaihu | MOL011865 | Oc1cc(ccc1O)CC(OC(=O)\C=C\c1cc(O)c(O)cc1)C(=O)[O-] |
| Chaihu | MOL011957 | Oc1cc(ccc1)C(=O)[O-] |
| Chaihu | MOL013333 | O1C(C(=O)[O-])C(O)C(O)C(OC2OC(C(=O)[O-])C(O)C(O)C2O)C1Oc1cc(O)c2c(OC(=CC2=O)c2ccc(O)cc2)c1 |
| Jinyinhua | MOL000042 | O=C([O-])C([NH3+])C |
| Jinyinhua | MOL000050 | O=C([O-])C[NH3+] |
| Jinyinhua | MOL000052 | O=C([O-])C([NH3+])CCC(=O)[O-] |
| Jinyinhua | MOL000053 | OC(C(O)C(=O)CO)C(O)CO |
| Jinyinhua | MOL000054 | O=C([O-])C([NH3+])CCCNC(=[NH2+])N |
| Jinyinhua | MOL000055 | O=C([O-])C([NH3+])CCCC[NH3+] |
| Jinyinhua | MOL000064 | OCC([NH3+])C(=O)[O-] |
| Jinyinhua | MOL000065 | O=C([O-])C([NH3+])CC(=O)[O-] |
| Jinyinhua | MOL000067 | O=C([O-])C([NH3+])C(C)C |
| Jinyinhua | MOL000068 | O=C([O-])C([NH3+])C(CC)C |
| Jinyinhua | MOL000069 | O=C([O-])CCCCCCCCCCCCCCC |
| Jinyinhua | MOL000114 | O(C)c1cc(ccc1O)C(=O)[O-] |
| Jinyinhua | MOL000116 | O=CCCCCCCCC |
| Jinyinhua | MOL000121 | O=CCCCCCCCCC |
| Jinyinhua | MOL000131 | O=C([O-])CCCCCCC\C=C/C\C=C/CCCCC |
| Jinyinhua | MOL000219 | O=C([O-])c1ccccc1 |
| Jinyinhua | MOL000223 | Oc1cc(ccc1O)\C=C\C(=O)[O-] |
| Jinyinhua | MOL000263 | OC1CCC2(C(CCC3(C2CC=C2C4CC(CCC4(CCC23C)C(=O)[O-])(C)C)C)C1(C)C)C |
| Jinyinhua | MOL000360 | O(C)c1cc(ccc1O)\C=C\C(=O)[O-] |
| Jinyinhua | MOL000421 | O=C([O-])c1cccnc1 |
| Jinyinhua | MOL000429 | O=C(N)CC([NH3+])C(=O)[O-] |
| Jinyinhua | MOL000476 | O(C)c1cc(O)c2c(c1)C(=O)c1c(C2=O)c(O)cc(c1)C |
| Jinyinhua | MOL000511 | OC1CCC2(C(CCC3(C2CC=C2C4C(C)C(CCC4(CCC23C)C(=O)[O-])C)C)C1(C)C)C |
| Jinyinhua | MOL000513 | Oc1c(O)cc(cc1O)C(=O)[O-] |
| Jinyinhua | MOL000515 | O=C([O-])CCCCCCCCCCCCCCCCCCCCCCCCCCCCC |
| Jinyinhua | MOL000550 | OC(C(O)C(O)CO)C(O)CO |
| Jinyinhua | MOL000627 | O(C)c1c2c(CC3[NH+](C2)CCc2cc(OC)c(O)cc23)ccc1O |
| Jinyinhua | MOL000659 | O=C([O-])CCCCCCCCCCCCCCCCCCCCCCCCCCC |
| Jinyinhua | MOL000662 | O=C([O-])CCCCCCCCCCCCCCCCCCCCCCCCC |
| Jinyinhua | MOL000663 | O=C([O-])CCCCCCCCCCCCCCCCCCCCCCC |
| Jinyinhua | MOL000675 | O=C([O-])CCCCCCC\C=C/CCCCCCCC |
| Jinyinhua | MOL000705 | O=CCCCCCC |
| Jinyinhua | MOL000749 | O=C([O-])CCCCCCC\C=C\C\C=C\CCCCC |
| Jinyinhua | MOL000766 | OCCCCCCCCCCCCCCCCCCCCCCCCCC |
| Jinyinhua | MOL000771 | Oc1ccc(cc1)\C=C\C(=O)[O-] |
| Jinyinhua | MOL000783 | O=C([O-])CCC=1C2=N\C(=C/c3[nH]c(\C=C\4/N=C(C=c5[nH]c(=C2)c(CCC(=O)[O-])c5C)C(C=C)=C/4C)c(C=C)c3C)\C=1C |
| Jinyinhua | MOL000787 | O1c2c3c(CC(=O)c4c(CC[NH+](C3)C)cc3OCOc3c4)ccc2OC1 |
| Jinyinhua | MOL000842 | O1C(OC2OC(CO)C(O)C(O)C2O)(CO)C(O)C(O)C1CO |
| Jinyinhua | MOL000860 | O=C([O-])CCCCCCCCCCCCCCCCC |
| Jinyinhua | MOL000879 | O(C(=O)CCCCCCCCCCCCCCC)C |
| Jinyinhua | MOL001456 | OC(CC(=O)[O-])(CC(=O)[O-])C(=O)[O-] |
| Jinyinhua | MOL001468 | OC(CC(=O)[O-])C(=O)[O-] |
| Jinyinhua | MOL001691 | O1C(C(O)CO)C([O-])=C(O)C1=O |
| Jinyinhua | MOL001798 | O1c2c(C(=O)CC1c1cc(OC)cc(O)c1)c(O)cc(O)c2 |
| Jinyinhua | MOL001801 | Oc1ccccc1C(=O)[O-] |
| Jinyinhua | MOL001945 | O1c2c(C=CC1=O)c(OC)c1c(occ1)c2 |
| Jinyinhua | MOL001996 | O=C1CCC2(C(CCC3(C2CCC2C4C(CCC23C)(CCC4C(C)=C)C(=O)[O-])C)C1(C)C)C |
| Jinyinhua | MOL002001 | O(C(=O)C)C1CCC2(C(CCC3(C2CC=C2C4CC(CCC4(CCC23C)C(=O)[O-])(C)C)C)C1(C)C)C |
| Jinyinhua | MOL002722 | OC1(\C=C\C(O)C)C(CC(=O)C=C1C)(C)C |
| Jinyinhua | MOL003396 | OC1CCC2(C(CCC3(C2CC=C2C4CC(CCC4(C(=O)[O-])C(O)CC23C)(C)C)C)C1(C)C)C |
| Jinyinhua | MOL003590 | O1c2c(C=CC1=O)ccc1occc12 |
| Jinyinhua | MOL003776 | O=C1CCC2(C(CCC3(C2CC=C2C4CC(CCC4(CCC23C)C(=O)[O-])(C)C)C)C1(C)C)C |
| Jinyinhua | MOL003971 | OC(C([NH3+])C(=O)[O-])C |
| Jinyinhua | MOL003991 | O=C1c2c(ccc(c2)C)C(=O)c2c1cccc2 |
| Jinyinhua | MOL004082 | O(C(COC(=O)CCCCCCCCCCCCCCCCC)COC(=O)CCCCCCCCCCCCCCCCC)C(=O)CCCCCCCCCCCCCCCCC |
| Jinyinhua | MOL004349 | O1C(C)C(O)C(OC)C(O)C1OC1CC2CCC3C(CCC4(C)C(CCC34O)C3=CC(OC3)=O)C2(CC1)CO |
| Jinyinhua | MOL004481 | S(SCC([NH3+])C(=O)[O-])CC([NH3+])C(=O)[O-] |
| Jinyinhua | MOL004498 | O(C(=O)c1cccnc1)C1CC2C(O)(CC=C3CC(O)CCC23C)C2(O)CCC(C(=O)C)C12C |
| Jinyinhua | MOL004738 | O(CCCc1ccccc1)C(=O)N |
| Jinyinhua | MOL004739 | O=C([O-])C([NH3+])C |
| Jinyinhua | MOL004743 | O(Cc1ccccc1)C(=O)NC(CC(C)C)C(=O)[O-] |
| Jinyinhua | MOL004779 | O=C([O-])\C(=C/C)\C |
| Jinyinhua | MOL005100 | O1c2c(C(=O)CC1c1cc(O)c(OC)cc1)c(O)cc(O)c2 |
| Jinyinhua | MOL005386 | O1C2C3C(CCC2C(C)C1=O)(C)C(=O)C=CC3(O)C |
| Jinyinhua | MOL005390 | OC(C(CO)(C)C)C(=O)NCCC(=O)[O-] |
| Jinyinhua | MOL005448 | O=C([O-])C([NH3+])CC(C)C |
| Jinyinhua | MOL005449 | S(CCC([NH3+])C(=O)[O-])C |
| Jinyinhua | MOL005559 | OC1C(C2CCC3(C(CC=C4C5CC(CCC5(CCC34C)C(=O)[O-])(C)C)C2(CC1O)C)C)(C)C |
| Jinyinhua | MOL005638 | O1c2c(C=CC1(C)C)c(C(OC)=O)c(O)c1c2cccc1 |
| Jinyinhua | MOL005928 | O(C)c1ccc(cc1O)\C=C\C(=O)[O-] |
| Jinyinhua | MOL006077 | s1c[n+](Cc2cnc(nc2N)C)c(C)c1CCO |
| Jinyinhua | MOL006135 | Oc1c2c(cc(O)c1C)C(=O)c1c(ccc(O)c1)C2=O |
| Jinyinhua | MOL006139 | O(C)c1c2c(cc(OC)c1C(=O)[O-])C(=O)c1c(cccc1)C2=O |
| Jinyinhua | MOL006141 | Oc1c2c(cc(O)c1CO)C(=O)c1c(ccc(c1)C)C2=O |
| Jinyinhua | MOL006142 | Oc1c2c(cc(O)c1COCC)C(=O)c1c(cccc1)C2=O |
| Jinyinhua | MOL006143 | O=C1C2=C(CC([O-])(C=C2[O-])C)C(=O)c2c1cccc2 |
| Jinyinhua | MOL006144 | Oc1c2c(C(=O)c3c(cc(cc3)C)C2=O)c(O)cc1 |
| Jinyinhua | MOL006145 | O(C)c1cc2c(cc1)C(=O)c1c(ccc(C)c1O)C2=O |
| Jinyinhua | MOL006146 | O(C)c1c2c(ccc1)C(=O)c1c(C2=O)c(O)ccc1 |
| Jinyinhua | MOL006147 | O(C)c1ccc2c(c1O)C(=O)c1c(cccc1)C2=O |
| Jinyinhua | MOL006148 | Oc1c2c(ccc1CO)C(=O)c1c(cccc1)C2=O |
| Jinyinhua | MOL006149 | O=C([O-])c1c2c(-c3c(CC2)c(C)c([O-])cc3)c(cc1)C=C |
| Jinyinhua | MOL006151 | O(C(C)=C)c1c2c(cc(O)c1C)C(=O)c1c(ccc(O)c1)C2=O |
| Jinyinhua | MOL006153 | O1c2c(CC(O)C1(C)C)c(C(OC)=O)c(O)c1c2cccc1 |
| Jinyinhua | MOL006155 | O=C1c2c(cc(cc2[O-])C(=O)[O-])C(=O)c2c1cccc2 |
| Jinyinhua | MOL006156 | O(C)C1C2C(C(=C1)CO)C(O)CC=C2C(=O)[O-] |
| Jinyinhua | MOL006158 | Oc1c2c(cc(O)c1C)C(=O)c1c(cccc1)C2=O |
| Jinyinhua | MOL006160 | Oc1c2c(ccc1O)C(=O)c1c(cccc1)C2=O |
| Jinyinhua | MOL006161 | Oc1c2c(C(=O)c3c(cccc3)C2=O)c(O)cc1C |
| Jinyinhua | MOL006162 | Oc1c2c(cc(O)c1C=O)C(=O)c1c(cccc1)C2=O |
| Jinyinhua | MOL006163 | Oc1c2c(ccc1C)C(=O)c1c(cccc1)C2=O |
| Jinyinhua | MOL006164 | O(C)C1=CC(=O)C=C(CCCCCCCCC\C=C/CCCCCC)C1=O |
| Jinyinhua | MOL006165 | o1c2c(cc1)c(C(OC)=O)c(O)c1c2cccc1 |
| Jinyinhua | MOL006167 | O1c2c(CCC1(C)C)c(C(OC)=O)c(O)c1c2cccc1 |
| Jinyinhua | MOL006168 | O1C2C(C=C(O)C3C2=CC=CC3C(OC)=O)C=2C3(Oc4c5c(C(C(OC)=O)C(=O)CC5(C)C)c(O)cc4C13)COC=2 |
| Jinyinhua | MOL006170 | Oc1c2c(cc(O)c1CO)C(=O)c1c(cccc1)C2=O |
| Jinyinhua | MOL006172 | O1c2c(C=CC1=O)c(C(OC)=O)c(O)c1c2cccc1 |
| Jinyinhua | MOL006173 | OC1CCC2(C(CCC3C2=CCC2(C4C(CCC23C)(C)C(CC4O)C(C)C)C)C1(C)C)C |
| Jinyinhua | MOL006175 | O1c2c(cccc2C(=O)[O-])C(=O)c2c1cccc2 |
| Jinyinhua | MOL006358 | O=C1C2=CC(=O)CCC2(C2C(C3CCC(C(CCC(C(C)C)CC)C)C3(CC2)C)C1)C |
| Jinyinhua | MOL006986 | O(C)c1c-2c3C([NH2+]CCc3cc1OC)Cc1c-2cccc1 |
| Jinyinhua | MOL007157 | o1cc(c2c1-c1c(c3c(cc1)c(ccc3)C)C(=O)C2=O)C |
| Jinyinhua | MOL007180 | S(=O)([O-])(=NC(=O)NC1CCC(CC1)C)c1ccc(cc1)CCNC(=O)N1CC(C)=C(CC)C1=O |
| Jinyinhua | MOL007253 | OC1C(C2CCC3(C(CC=C4C5C(C)C(CCC5(CCC34C)C(=O)[O-])C)C2(CC1O)C)C)(CO)C |
| Jinyinhua | MOL007341 | OC1C(C2CCC3(C(CC=C4C5C(CCC34C)(CCC5C(C)=C)C(=O)[O-])C2(CC1O)C)C)(C)C |
| Jinyinhua | MOL007347 | Oc1ccc(cc1)CC(NC(OC(C)(C)C)=O)C(=O)[O-] |
| Jinyinhua | MOL008032 | O=C1CCC2(C(CCC3(C2CC=C2C4C(C)C(CCC4(CCC23C)C(=O)[O-])C)C)C1(C)C)C |
| Jinyinhua | MOL008034 | OC1C(C(=O)[O-])C2(C(CCC3(C2CCC2C4C(CCC23C)(CCC4C(C)=C)C(=O)[O-])C)C1(C)C)C |
| Jinyinhua | MOL008680 | O=CC |
| Jinyinhua | MOL008695 | O1c2c(cc(O)cc2C)CCC1(CCCC(CCCC(CCCC(C)C)C)C)C |
| Jinyinhua | MOL009506 | Oc1c2c(ccc1)C(=O)c1c(cccc1)C2=O |
| Jinyinhua | MOL010527 | O=C([O-])CCCCCCCC=C=CCCCCCCC |
| Jinyinhua | MOL011748 | O1c2c(CC1C(C)C)cc1C=CC(Oc1c2)=O |
| Jinyinhua | MOL011960 | O(C(=O)CCCCCC\C=C/CCCCC(CC)C)C |
| Jinyinhua | MOL011976 | O(C)c1ccc(cc1)C(OCCc1ccc(O)cc1)=O |
| Jinyinhua | MOL011977 | O(C)c1cc(ccc1O)\C=C\C(OCCc1ccc(O)cc1)=O |
| Jinyinhua | MOL012994 | O1C23C(COC(=O)C)(C(OC(=O)c4cccnc4)C(OC(=O)c4ccccc4)C(C1(C)C)C2OC(=O)C)C(OC(=O)C)CCC3(O)C |
| Jinyinhua | MOL012995 | OC1C(C=O)C2(C(CCC3(C2CCC2C4C(CCC23C)(CCC4C(C)=C)C(=O)[O-])C)C1(C)C)C |
| Jinyinhua | MOL012996 | OC1C(C2CCC3(C(CCC4C5C(CCC34C)(CCC5C(C)=C)C(=O)[O-])C2(CC1O)C)C)(C)C |
| Jinyinhua | MOL012997 | n1cn(c-2ncnc-2c1N)C\C=C(\C)/C |
| Yinxingye | MOL000008 | O1c2c(C(=O)C=C1c1ccc(O)cc1)c(O)cc(O)c2 |
| Yinxingye | MOL000069 | O=C([O-])CCCCCCCCCCCCCCC |
| Yinxingye | MOL000105 | Oc1cc(ccc1O)C(=O)[O-] |
| Yinxingye | MOL000114 | O(C)c1cc(ccc1O)C(=O)[O-] |
| Yinxingye | MOL000116 | O=CCCCCCCCC |
| Yinxingye | MOL000121 | O=CCCCCCCCCC |
| Yinxingye | MOL000131 | O=C([O-])CCCCCCC\C=C/C\C=C/CCCCC |
| Yinxingye | MOL000172 | o1cccc1C=O |
| Yinxingye | MOL000219 | O=C([O-])c1ccccc1 |
| Yinxingye | MOL000223 | Oc1cc(ccc1O)\C=C\C(=O)[O-] |
| Yinxingye | MOL000259 | Oc1cc(ccc1C)C(C)C |
| Yinxingye | MOL000263 | OC1CCC2(C(CCC3(C2CC=C2C4CC(CCC4(CCC23C)C(=O)[O-])(C)C)C)C1(C)C)C |
| Yinxingye | MOL000346 | O=C([O-])CCC(=O)[O-] |
| Yinxingye | MOL000360 | O(C)c1cc(ccc1O)\C=C\C(=O)[O-] |
| Yinxingye | MOL000397 | Oc1ccc(cc1)\C=C/C(=O)[O-] |
| Yinxingye | MOL000481 | O1C=C(C(=O)c2c1cc(O)cc2O)c1ccc(O)cc1 |
| Yinxingye | MOL000511 | OC1CCC2(C(CCC3(C2CC=C2C4C(C)C(CCC4(CCC23C)C(=O)[O-])C)C)C1(C)C)C |
| Yinxingye | MOL000551 | OC1CCC2(C(CCC3(C2CC=C2C4CC(CCC4(CCC23C)C(=O)[O-])(C)C)C)C1(CO)C)C |
| Yinxingye | MOL000676 | O(C(=O)c1ccccc1C(OCCCC)=O)CCCC |
| Yinxingye | MOL000708 | O=Cc1ccccc1 |
| Yinxingye | MOL000732 | O1C(OC2OC(COC3OC(COC4OC(CO)C(O)C(O)C4O)C(O)C(O)C3O)C(O)C(O)C2O)(CO)C(O)C(O)C1CO |
| Yinxingye | MOL000945 | Oc1ccc(cc1)\C=C/C(=O)CC(=O)\C=C\c1ccc(O)cc1 |
| Yinxingye | MOL000946 | O(C)c1cc(ccc1O)\C=C\C(=O)CC(=O)\C=C\c1ccc(O)cc1 |
| Yinxingye | MOL000949 | OC1(C2C(CC1)C(CC(=O)/C(/C2)=C(/C)\C)=C)C |
| Yinxingye | MOL000955 | Oc1cc(ccc1C)C(CC(=O)\C=C(\C)/C)C |
| Yinxingye | MOL000959 | OC1(CC(=O)/C(/CC2C1CCC2(O)C)=C(/C)\C)C |
| Yinxingye | MOL000961 | OC1(C2C(CC1)C(=CC(=O)/C(/C2)=C(/C)\C)C)C |
| Yinxingye | MOL000966 | Oc1cc(ccc1C(CC(=O)\C=C(\C)/C)C)C |
| Yinxingye | MOL000991 | O=C\C=C\c1ccccc1 |
| Yinxingye | MOL001040 | O1c2c(C(=O)CC1c1ccc(O)cc1)c(O)cc(O)c2 |
| Yinxingye | MOL001103 | O(C(CC\C=C(\C)/C)(C=C)C)C(=O)C |
| Yinxingye | MOL001392 | O(C(=O)CCCCCCCCCCCCC)C |
| Yinxingye | MOL001398 | O(C(=O)CCCCCCC\C=C/C\C=C/C\C=C/CC)C |
| Yinxingye | MOL001434 | [O+]1=C2C(=C(O)[CH-]C(O)=C2)C(=O)C(O)=C1c1cc(O)c(O)cc1 |
| Yinxingye | MOL001498 | O(C(=O)CCCCCCCCCCCCCCCCC)CC |
| Yinxingye | MOL001584 | O(C(CC\C=C(\C)/C)(C=C)C)C(=O)CC |
| Yinxingye | MOL001644 | O=CCCCCCCCCCCC |
| Yinxingye | MOL001691 | O1C(C(O)CO)C([O-])=C(O)C1=O |
| Yinxingye | MOL001817 | O(C(=O)CCCCCCCCCCCCCCCCC)C |
| Yinxingye | MOL001819 | O(C(=O)CCCCCCCCCCCCCC)C |
| Yinxingye | MOL001837 | Oc1ccc(cc1)\C=C\C(OC)=O |
| Yinxingye | MOL001843 | Oc1ccc(cc1)C=O |
| Yinxingye | MOL001873 | Oc1ccc(cc1)C(OCC)=O |
| Yinxingye | MOL001875 | O(C(=O)\C=C/c1cc(O)c(O)cc1)C1CC(O)(CC(OC(=O)\C=C\c2cc(O)c(O)cc2)C1O)C(=O)[O-] |
| Yinxingye | MOL001878 | O(C(=O)\C=C\c1cc(O)c(O)cc1)C1C(OC(=O)\C=C\c2cc(O)c(O)cc2)CC(O)(CC1O)C(=O)[O-] |
| Yinxingye | MOL001945 | O1c2c(C=CC1=O)c(OC)c1c(occ1)c2 |
| Yinxingye | MOL001955 | O(C(=O)\C=C\c1cc(O)c(O)cc1)C1CC(O)(CC(O)C1O)C(=O)[O-] |
| Yinxingye | MOL002027 | O(C(=O)CCCCCCCCCCCCCCCCCCCCC)C |
| Yinxingye | MOL002042 | Oc1cc(ccc1C(C)C)C |
| Yinxingye | MOL002558 | O1c2cc(O)ccc2C=CC1=O |
| Yinxingye | MOL002882 | O(C(=O)CCCCCCC\C=C/CCCCCCCC)CC(O)CO |
| Yinxingye | MOL003177 | O(C)c1cc(cc(OC)c1O)C=O |
| Yinxingye | MOL003717 | O(C(=O)CCCCCCC\C=C/CCCCCC)C |
| Yinxingye | MOL003902 | O(C(=O)CCCCCCCCC\C=C/CCCCCCCC)C |
| Yinxingye | MOL003924 | O(C(=O)CCCCCCCC(OC)=O)C |
| Yinxingye | MOL003962 | O(C(=O)CC)C\C=C(\CC\C=C(\C)/C)/C |
| Yinxingye | MOL003968 | O(C(=O)CCCC)C\C=C(/CC\C=C(\C)/C)\C |
| Yinxingye | MOL004087 | O(C(=O)\C=C\c1cc(O)c(O)cc1)C1CC(OC(=O)\C=C\c2cc(O)c(O)cc2)(CC(O)C1O)C(=O)[O-] |
| Yinxingye | MOL004682 | O(C(=O)CCCCCCC)C |
| Yinxingye | MOL004795 | O(C)C1=CC(=O)c2c(cccc2)C1=O |
| Yinxingye | MOL005530 | O1c2c(C(=O)C=C1c1cc(O)c(O)cc1)c(O)cc(OC)c2 |
| Yinxingye | MOL005573 | O1c2c(C(=O)C=C1c1ccc(O)cc1)c(O)cc(OC)c2 |
| Yinxingye | MOL005574 | O1C2\C=C(\CC\C=C(\C=O)/C(O)C(OC(=O)CCC)C2C(=C)C1=O)/C |
| Yinxingye | MOL005575 | O1c2c(C(=O)c3c1ccc(O)c3OC)c(O)cc(OC)c2 |
| Yinxingye | MOL005577 | O=CCCCCCCCCCC |
| Yinxingye | MOL005797 | O=CCCCCCCCCCCCC |
| Yinxingye | MOL006370 | O(C(=O)\C=C\c1cc(O)c(O)cc1)C1CC(O)(CC(O)C1O)C(=O)[O-] |
| Yinxingye | MOL006378 | O(C(=O)\C=C\c1cc(O)c(O)cc1)C1CC(O)(CC(O)C1O)C(=O)[O-] |
| Yinxingye | MOL006387 | O(C(=O)\C=C\c1cc(O)c(O)cc1)C1CC(O)(CC(O)C1O)C(=O)[O-] |
| Yinxingye | MOL006407 | O(C(=O)\C=C\c1cc(O)c(O)cc1)C1CC(O)(CC(O)C1O)C(=O)[O-] |
| Yinxingye | MOL006501 | O(C(=O)\C=C\c1cc(O)c(O)cc1)C1C(OC(=O)\C=C\c2cc(O)c(O)cc2)CC(O)(CC1O)C(=O)[O-] |
| Yinxingye | MOL007192 | O1C(C(=O)[O-])C(O)C(O)C(O)C1OC1=C(Oc2c(C1=O)c(O)cc(O)c2)c1cc(O)c(O)cc1 |
| Yinxingye | MOL007193 | O1C(C(=O)[O-])C(O)C(O)C(O)C1OC1=C(Oc2c(C1=O)c(O)cc(O)c2)c1cc(O)c(O)cc1 |
| Yinxingye | MOL007201 | OC1C(C2C(C3CC=C4C5C(C)C(CCC5(CCC4(C)C3(CC2O)C)C(=O)[O-])C)(CC1O)C)(CO)C |
| Yinxingye | MOL007223 | O1C(C(=O)[O-])C(O)C(O)C(O)C1OC1=C(Oc2c(C1=O)c(O)cc(O)c2)c1cc(O)c(O)cc1 |
| Yinxingye | MOL007253 | OC1C(C2CCC3(C(CC=C4C5C(C)C(CCC5(CCC34C)C(=O)[O-])C)C2(CC1O)C)C)(CO)C |
| Yinxingye | MOL007254 | OC1C(C2CCC3(C(CC=C4C5CC(CCC5(CCC34C)C(=O)[O-])(C)C)C2(CC1O)C)C)(CO)C |
| Yinxingye | MOL007302 | O1C(COC2OC(CO)C(OOC3OC(C)C(O)C(O)C3O)C(O)C2O)C(O)C(O)C(O)C1OC(=O)C12C(C3=CCC4C(CC(O)C5C(CO)(C)C(O)C(O)CC45C)(C)C3(CC1)C)C(C)C(CC2)C |
| Yinxingye | MOL007303 | OC1C(C2C(C3CC=C4C5C(C)C(CCC5(CCC4(C)C3(CC2O)C)C(=O)[O-])C)(CC1O)C)(CO)C |
| Yinxingye | MOL007304 | O(Oc1cc(ccc1O)\C=C\C(=O)C)C1C(OOc2cc(ccc2O)\C=C\C(=O)C)CC(O)(CC1O)C(=O)[O-] |
| Yinxingye | MOL007305 | O(C(=O)\C=C\c1cc(O)c(O)cc1)C1C(OC(=O)\C=C\c2cc(O)c(O)cc2)CC(O)(CC1O)C(=O)[O-] |
| Yinxingye | MOL007306 | O(C(=O)CC(=O)[O-])C1C(OC(=O)\C=C\c2cc(O)c(O)cc2)CC(O)(CC1OC(=O)\C=C\c1cc(O)c(O)cc1)C(=O)[O-] |
| Yinxingye | MOL007310 | OC1C(C2CCC3(C(CC=C4C5C(CC(C)C34C)C(CC(C)C5C)C(=O)[O-])C2(CC1O)C)C)(CO)C |
| Yinxingye | MOL007311 | O1C(COC2OC(CO)C(OOC3OC(C)C(O)C(O)C3O)C(O)C2O)C(O)C(O)C(O)C1OC(=O)C12C(C3=CCC4C(CC(O)C5C(CO)(C)C(O)C(O)CC45C)(C)C3(CC1)C)CC(CC2)(C)C |
| Yinxingye | MOL007312 | OC1C(C2C(C3CC=C4C5CC(CCC5(CCC4(C)C3(CC2O)C)C(=O)[O-])(C)C)(CC1O)C)(CO)C |
| Yinxingye | MOL007313 | O1c2c(cccc2)C(c2c1cccc2)C(=O)[O-] |
| Yinxingye | MOL007314 | O1C(C(=O)[O-])C(O)C(O)C(O)C1OC1=C(Oc2c(C1=O)c(O)cc(O)c2)c1ccc(O)cc1 |
| Yinxingye | MOL007315 | O(Oc1cc(ccc1O)\C=C\C(=O)C)C1CC(O)(CC(OOc2cc(ccc2O)\C=C\C(=O)C)C1O)C(=O)[O-] |
| Yinxingye | MOL007316 | O(C(=O)\C=C\c1cc(O)c(O)cc1)C1CC(O)(CC(OC(=O)\C=C\c2cc(O)c(O)cc2)C1O)C(=O)[O-] |
| Yinxingye | MOL007317 | O1C(C)C(O)C(O)C(O)C1OC1C(O)C(O)C(OC1CO)OCC1OC(OC(=O)C23C(C=4C5(C6(C(CC=4)C4(C(CC6)C(CO)(C)C(O)C(O)C4)C)C5)CC2)C(C)C(CC3)C)C(O)C(O)C1O |
| Yinxingye | MOL007318 | OC1C(C2CCC34C(CC=C5C6C(C)C(CCC6(CCC35C4)C(=O)[O-])C)C2(CC1O)C)(CO)C |
| Yinxingye | MOL007319 | OC1C(C2C(C3CC=C4C5C(C)C(CCC5(CCC4(C)C3(CC2O)C)C(=O)[O-])C)(CC1O)C)(CO)C |
| Yinxingye | MOL007320 | O(C(\C=C/CCCCC)C#CC#CC(O)C=C)C(=O)C |
| Yinxingye | MOL007323 | OC1C(C2C(C3CC=C4C5C(C)C(CCC5(CCC4(C)C3(CC2O)C)C(=O)[O-])C)(CC1O)C)(C)C |
| Yinxingye | MOL007326 | O(C(=O)\C=C\c1cc(O)c(O)cc1)C1CC(OC(=O)\C=C\c2cc(O)c(O)cc2)(CC(O)C1O)C(=O)[O-] |
| Yinxingye | MOL007327 | Clc1c(Cl)c(OCC(=O)[O-])ccc1C(=O)C(CC)=C |
| Yinxingye | MOL007328 | O1C(COC2OC(CO)C(OOC3OC(C)C(O)C(O)C3O)C(O)C2O)C(O)C(O)C(O)C1OC(=O)C12C(C3=CCC4C(CCC5C(CO)(C)C(O)C(O)CC45C)(C)C3(CC1)C)C(C)C(CC2)C |
| Yinxingye | MOL007329 | OC1C(C2CCC3(C(CC=C4C5C(C)C(CCC5(CCC34C)C(=O)[O-])C)C2(CC1O)C)C)(CO)C |
| Yinxingye | MOL008121 | O(C(CO)CO)C(=O)CCCCCCC\C=C/CCCCCCCC |
| Yinxingye | MOL008218 | O(C(=O)CCCCCCC\C=C/CCCCCCCC)CC(O)CO |
| Yinxingye | MOL008594 | O(C(=O)CCCCC\C=C/CCCCCCCC)C |
| Yinxingye | MOL008595 | O(C(=O)CCCCCCCCCCCCCCCCCCCC)C |
| Yinxingye | MOL008596 | o1c2c(c3c1c1c(cccc1)c(O)c3OC)C(=O)c1c(cccc1)C2=O |
| Yinxingye | MOL008598 | O(C(=O)CCC\C=C/C\C=C/C\C=C/C\C=C/C\C=C/CC)C |
| Yinxingye | MOL008599 | O(C(=O)CCCCCCC\C=C\CCCCCCCC)CC |
| Yinxingye | MOL008600 | O(C(=O)CCCCCCC\C=C\CCCCCCCC)CC(O)CO |
| Yinxingye | MOL008601 | O(C(=O)CCC\C=C/C\C=C/C\C=C/C\C=C/CCCCC)C |
| Yinxingye | MOL008602 | O(C(=O)\C=C\CCCCCCCCCCCCC)C |
| Yinxingye | MOL010928 | O(C(=O)CCCCCCCCCCCCCCCCC)CC(O)CO |
| Yinxingye | MOL010929 | O(C(=O)CCCCCCC\C=C/C\C=C/C\C=C/CC)CC(O)CO |
| Yinxingye | MOL012152 | O1C(=Cc2c(C1=O)c(OC)c1c(occ1)c2)C |
| Yinxingye | MOL012154 | O1C(Cc2c(c3OC(C)(C)C(O)Cc3c(OC)c2)C1=O)C |
| Moyao | MOL000002 | [o+]1c2c(cc(O)c1-c1cc(O)c(O)cc1)c(O)cc(O)c2 |
| Moyao | MOL000041 | O=C([O-])C([NH3+])Cc1ccccc1 |
| Moyao | MOL000042 | O=C([O-])C([NH3+])C |
| Moyao | MOL000050 | O=C([O-])C[NH3+] |
| Moyao | MOL000052 | O=C([O-])C([NH3+])CCC(=O)[O-] |
| Moyao | MOL000055 | O=C([O-])C([NH3+])CCCC[NH3+] |
| Moyao | MOL000056 | Oc1ccc(cc1)CC([NH3+])C(=O)[O-] |
| Moyao | MOL000065 | O=C([O-])C([NH3+])CC(=O)[O-] |
| Moyao | MOL000067 | O=C([O-])C([NH3+])C(C)C |
| Moyao | MOL000068 | O=C([O-])C([NH3+])C(CC)C |
| Moyao | MOL000103 | Oc1ccc(cc1)C(=O)[O-] |
| Moyao | MOL000105 | Oc1cc(ccc1O)C(=O)[O-] |
| Moyao | MOL000114 | O(C)c1cc(ccc1O)C(=O)[O-] |
| Moyao | MOL000131 | O=C([O-])CCCCCCC\C=C/C\C=C/CCCCC |
| Moyao | MOL000172 | o1cccc1C=O |
| Moyao | MOL000206 | O(C)c1cc(ccc1O)\C=C\C |
| Moyao | MOL000223 | Oc1cc(ccc1O)\C=C\C(=O)[O-] |
| Moyao | MOL000263 | OC1CCC2(C(CCC3(C2CC=C2C4CC(CCC4(CCC23C)C(=O)[O-])(C)C)C)C1(C)C)C |
| Moyao | MOL000303 | O=C([O-])CCCCCCC |
| Moyao | MOL000305 | O=C([O-])CCCCCCCCCCC |
| Moyao | MOL000389 | O(C)c1cc(ccc1O)\C=C/C(=O)[O-] |
| Moyao | MOL000414 | Oc1cc(ccc1O)\C=C/C(=O)[O-] |
| Moyao | MOL000421 | O=C([O-])c1cccnc1 |
| Moyao | MOL000511 | OC1CCC2(C(CCC3(C2CC=C2C4C(C)C(CCC4(CCC23C)C(=O)[O-])C)C)C1(C)C)C |
| Moyao | MOL000513 | Oc1c(O)cc(cc1O)C(=O)[O-] |
| Moyao | MOL000538 | O(C)C1C2(O)CC3C(C(OC(=O)C)(C4C5[NH+](CC6(C(C35C(OC)CC6)C4OC)COC)C)C1O)C2OC(=O)c1ccccc1 |
| Moyao | MOL000598 | OCCCCCCCCCCCCCCCCCCCCCCCCCCCC |
| Moyao | MOL000771 | Oc1ccc(cc1)\C=C\C(=O)[O-] |
| Moyao | MOL001393 | O=C([O-])CCCCCCCCCCCCC |
| Moyao | MOL001398 | O(C(=O)CCCCCCC\C=C/C\C=C/C\C=C/CC)C |
| Moyao | MOL001456 | OC(CC(=O)[O-])(CC(=O)[O-])C(=O)[O-] |
| Moyao | MOL001468 | OC(CC(=O)[O-])C(=O)[O-] |
| Moyao | MOL001477 | Oc1ccc(cc1)CC[NH3+] |
| Moyao | MOL001494 | O(C(=O)CCCCCCC\C=C/C\C=C/CCCCC)CC |
| Moyao | MOL001495 | O(C(=O)CCCCCCC\C=C\C\C=C\C\C=C\CC)CC |
| Moyao | MOL001624 | Oc1cc(O)cc(O)c1C(=O)CCc1ccc(O)cc1 |
| Moyao | MOL001681 | O1C=C(C2C(C(C)C(O)C2)C1O)C(OC)=O |
| Moyao | MOL001691 | O1C(C(O)CO)C([O-])=C(O)C1=O |
| Moyao | MOL001739 | O=C([O-])CCCCCCC\C=C/CCCCCC |
| Moyao | MOL001816 | O=C(N)CCCCCCCCCCCCCCC |
| Moyao | MOL001886 | OC(C(O)C(=O)[O-])C(=O)[O-] |
| Moyao | MOL001889 | O(C(=O)CCCCCCC\C=C\C\C=C\CCCCC)C |
| Moyao | MOL001955 | O(C(=O)\C=C\c1cc(O)c(O)cc1)C1CC(O)(CC(O)C1O)C(=O)[O-] |
| Moyao | MOL001996 | O=C1CCC2(C(CCC3(C2CCC2C4C(CCC23C)(CCC4C(C)=C)C(=O)[O-])C)C1(C)C)C |
| Moyao | MOL002046 | O=C([O-])CCCCC |
| Moyao | MOL002343 | O1c2c3C([NH+](CCc3cc(OC)c2OC)C)Cc2cc(Oc3ccc(CC4[NH+](CCc5cc(OC)c1cc45)C)cc3)c(OC)cc2 |
| Moyao | MOL002373 | O1c2c(cc(O)c(C)c2C)CCC1(CCCC(CCCC(CCCC(C)C)C)C)C |
| Moyao | MOL002434 | OC1CCC2C(=CCC3C4(CCC(C(CC\C=C(\CO)/C)C)C4(CC(=O)C23C)C)C)C1(C)C |
| Moyao | MOL002435 | O(C)C1C2(O)CC3C(C(O)(C4C5[NH+](CC6(C(C35C(OC)CC6O)C4OC)COC)CC)C1O)C2O |
| Moyao | MOL002436 | O(C(=O)c1ccccc1)C1C2C3C45C6C(CCC4OC)C[NH+](C5C(C6)C2(O)CC(OC)C1(O)C3)CC |
| Moyao | MOL002437 | O1C23C4(OC1)C1C(C56C(C(CCC5OC)(C[NH+](C26)CC)COC)C3OC)CC(C1O)C(OC)C4 |
| Moyao | MOL002438 | O1C23C4(OC1)C1C(C56C(C(CCC5OC)(C[NH+](C26)CC)COC)C3OC)CC(C1OC)C(OC)C4 |
| Moyao | MOL002484 | O=CCC(C)C |
| Moyao | MOL002850 | Oc1c(cc(cc1C(C)(C)C)C)C(C)(C)C |
| Moyao | MOL002883 | O(C(=O)CCCCCCC\C=C/CCCCCCCC)CC |
| Moyao | MOL003010 | O1C(C(=O)[O-])C(O)C(O)C(O)C1OC1=C(Oc2c(C1=O)c(O)cc(O)c2)c1cc(O)c(O)cc1 |
| Moyao | MOL003374 | Oc1c(cccc1O)CCCCCCC\C=C\C\C=C\CCC |
| Moyao | MOL003484 | c12c3c(ccc1cccc2)cccc3 |
| Moyao | MOL003788 | O(Cc1ccccc1)C(=O)\C=C\c1ccccc1 |
| Moyao | MOL003877 | OCCC(C)C |
| Moyao | MOL003940 | O=C(N)CCCCCCCCCCCCCCCCC |
| Moyao | MOL003955 | S(SCC([NH3+])C(=O)[O-])CC([NH3+])C(=O)[O-] |
| Moyao | MOL003969 | OCC([NH3+])C(=O)[O-] |
| Moyao | MOL003971 | OC(C([NH3+])C(=O)[O-])C |
| Moyao | MOL004048 | OC(C(O)C(=O)CO)C(O)CO |
| Moyao | MOL004284 | O=C(CCCCCCCCCCCCCCCCC)C |
| Moyao | MOL004784 | OCCCCCCCCCCCCCCCCCC |
| Moyao | MOL005272 | O(C(=O)C)CCCCCCCCCCCCC=C |
| Moyao | MOL005386 | O1C2C3C(CCC2C(C)C1=O)(C)C(=O)C=CC3(O)C |
| Moyao | MOL005449 | S(CCC([NH3+])C(=O)[O-])C |
| Moyao | MOL005462 | O1C(OC)C2(OC2C12OC1C(C3(C)C(C=4C5(CC3OC(=O)C)C3(C(CC=4)C(C)(C)C(OC4OCC(O)C(O)C4O)CC3)C5)(C1)C)C(C2)C)C |
| Moyao | MOL005463 | O1C(OC)C2(OC2C12OC1C(C3(C)C(C=4C5(CC3OC(=O)C)C3(C(CC=4)C(C)(C)C(O)CC3)C5)(C1)C)C(C2)C)C |
| Moyao | MOL005464 | OC1CC2(C(CCC3C2C(=O)C=C(C=C)C3C)C(C)(C)C1=O)C |
| Moyao | MOL005469 | O1C(CO)C(O)C(OC(=O)c2cc(O)c(O)c(O)c2)C(OC(=O)c2cc(O)c(O)c(O)c2)C1OC(=O)c1cc(O)c(O)c(O)c1 |
| Moyao | MOL005472 | O(C(=O)c1ccccc1C(=O)[O-])CC(CCCC)CC |
| Moyao | MOL005473 | O=C(C)c1c2c(ccc1)c(ccc2)C(=O)C |
| Moyao | MOL005475 | ClCCCCCCCCCCCCCCCCCCCC |
| Moyao | MOL005478 | O(C(=O)CCCCCCCCC\C=C\C\C=C\CCC)C |
| Moyao | MOL005480 | O(C(C)C)CCO |
| Moyao | MOL005482 | o1cccc1C(=O)[O-] |
| Moyao | MOL005484 | O=C([O-])C(C)c1ccccc1 |
| Moyao | MOL005485 | S(=O)(=O)([O-])c1cc2oc3c(c2cc1)cccc3 |
| Moyao | MOL005487 | Oc1cc(cc(O)c1)C(=O)[O-] |
| Moyao | MOL005488 | Oc1c(cc(cc1C(C)(C)C)C=O)C(C)(C)C |
| Moyao | MOL005490 | O=C([O-])c1ccc(cc1)-c1ccc(cc1)CC |
| Moyao | MOL005494 | S(OCCCC)(=O)(=O)C(CCCCCCCC)CCCCC |
| Moyao | MOL005495 | Oc1c2c(ccc1CC)C(=O)C([O-])=CC2=O |
| Moyao | MOL005500 | O=C([O-])CCCCCCC\C=C\C\C=C\C\C=C\CC |
| Moyao | MOL005501 | c12c(cc3c(c1)cccc3)cccc2 |
| Moyao | MOL005502 | O(C(CC(=O)[O-])C(=O)[O-])CCCC |
| Moyao | MOL005503 | O(C)C1=CC(=O)C=C(CC(OC(=O)C)CCCCCCCCCCC)C1=O |
| Moyao | MOL005508 | OC1C(C2CCC3(C(CC=C4C5C(C)C(CCC5(CCC34C)C(=O)[O-])C)C2(CC1O)C)C)(C)C |
| Moyao | MOL005511 | O1c2c(cc(O)cc2C)CCC1(CCCC(CCCC(CCCC(C)C)C)C)C |
| Moyao | MOL005513 | o1c2c(c3c1cccc3)cccc2 |
| Moyao | MOL005514 | O(C(=O)C(O)CCCCCCCCCCCC)CC |
| Moyao | MOL005515 | O(C)c1cc(OC)cc(OC)c1C(=O)CC(=O)C |
| Moyao | MOL005522 | [NH3+]CCC(C)C |
| Moyao | MOL005530 | O1c2c(C(=O)C=C1c1cc(O)c(O)cc1)c(O)cc(OC)c2 |
| Moyao | MOL005559 | OC1C(C2CCC3(C(CC=C4C5CC(CCC5(CCC34C)C(=O)[O-])(C)C)C2(CC1O)C)C)(C)C |
| Moyao | MOL006077 | s1c[n+](Cc2cnc(nc2N)C)c(C)c1CCO |
| Moyao | MOL006356 | OC(C(O)C(O)CO)C(O)CO |
| Moyao | MOL008457 | O1C=C(C2C(C[NH+]3C(C2)c2[nH]c4c(c2CC3)cccc4)C1C)C(OC)=O |
| Moyao | MOL008886 | O(C)c1ccccc1CC[NH3+] |
| Moyao | MOL008887 | [NH3+]C(C)c1ccccc1 |
| Moyao | MOL008890 | [NH2+](CC)CC |
| Moyao | MOL008891 | [NH3+]CC |
| Moyao | MOL008892 | [NH3+]CC(C)C |
| Chuanxiong | MOL000003 | OC(C(O)C(O)CO)C(O)CO |
| Chuanxiong | MOL000116 | O=CCCCCCCCC |
| Chuanxiong | MOL000121 | O=CCCCCCCCCC |
| Chuanxiong | MOL000131 | O=C([O-])CCCCCCC\C=C/C\C=C/CCCCC |
| Chuanxiong | MOL000303 | O=C([O-])CCCCCCC |
| Chuanxiong | MOL000305 | O=C([O-])CCCCCCCCCCC |
| Chuanxiong | MOL000388 | O=C([O-])CCC[NH3+] |
| Chuanxiong | MOL000394 | OCC[N+](C)(C)C |
| Chuanxiong | MOL000399 | O=C([O-])CCCCCCCCCCCCCCCCCCCCC |
| Chuanxiong | MOL000430 | O=C([O-])C[N+](C)(C)C |
| Chuanxiong | MOL000602 | O=C([O-])\C=C\C(=O)[O-] |
| Chuanxiong | MOL000663 | O=C([O-])CCCCCCCCCCCCCCCCCCCCCCC |
| Chuanxiong | MOL000705 | O=CCCCCCC |
| Chuanxiong | MOL000714 | O=CCc1ccccc1 |
| Chuanxiong | MOL000860 | O=C([O-])CCCCCCCCCCCCCCCCC |
| Chuanxiong | MOL000924 | O=C(CCCCCCCCC)C |
| Chuanxiong | MOL001393 | O=C([O-])CCCCCCCCCCCCC |
| Chuanxiong | MOL001396 | O=C([O-])CCCCCCCCCCCCCC |
| Chuanxiong | MOL001762 | O=Cc1c2c([nH]c1)cccc2 |
| Chuanxiong | MOL001768 | o1c(ccc1CO)C(=O)[O-] |
| Chuanxiong | MOL001774 | OC12C(CCCC1(C)C)C1(C(=CC(CC1O)(C=C)C)C(=O)C2)C |
| Chuanxiong | MOL001778 | O(C)c1cc(cc(OC)c1O)\C=C\C=O |
| Chuanxiong | MOL001779 | O(C)C1=CC23c4c(CC([NH+](CC2)C)C3=CC1=O)ccc(OC)c4O |
| Chuanxiong | MOL001787 | O1C(CO)C(O)C(O)C1n1c2ncnc(N)c2nc1 |
| Chuanxiong | MOL001788 | [nH]1c2ncnc(N)c2nc1 |
| Chuanxiong | MOL001789 | Oc1cc(O)ccc1C(=O)\C=C\c1ccc(O)cc1 |
| Chuanxiong | MOL001792 | O1c2c(ccc(O)c2)C(=O)CC1c1ccc(O)cc1 |
| Chuanxiong | MOL001794 | O=C([O-])\C=C/C(=O)[O-] |
| Chuanxiong | MOL001795 | S(\C(=N\OS(=O)(=O)[O-])\Cc1c2c(n(OC)c1)cccc2)C1OC(CO)C(O)C(O)C1O |
| Chuanxiong | MOL001796 | S(O\N=C(\[S-])/Cc1c2c(n(OC)c1)cccc2)(=O)(=O)[O-] |
| Chuanxiong | MOL001798 | O1c2c(C(=O)CC1c1cc(OC)cc(O)c1)c(O)cc(O)c2 |
| Chuanxiong | MOL001799 | O=C1c2c=3[nH]c(=CC4=NC(=Cc5[nH]c(C=C6N=C(C=3C1)C(CCC(OC)=O)C6C)c(C)c5C=C)C(C)=C4CC)c2C |
| Chuanxiong | MOL001801 | Oc1ccccc1C(=O)[O-] |
| Chuanxiong | MOL001807 | O(C)c1cc(cc(OC)c1O)C(=O)[O-] |
| Chuanxiong | MOL001814 | O(C)c1cc(cc(OC)c1)/C(/[O-])=C/1\c2c(NC\1=O)cccc2 |
| Chuanxiong | MOL001816 | O=C(N)CCCCCCCCCCCCCCC |
| Chuanxiong | MOL001817 | O(C(=O)CCCCCCCCCCCCCCCCC)C |
| Chuanxiong | MOL001818 | O(C(=O)CCCCCCC\C=C\CCCCCC)C |
| Chuanxiong | MOL001819 | O(C(=O)CCCCCCCCCCCCCC)C |
| Chuanxiong | MOL001822 | o1c(ccc1COC)C(=O)[O-] |
| Chuanxiong | MOL001943 | P(OCC(OC(=O)CCCCCCC\C=C/C\C=C/CCCCC)COC(=O)CCCCCCCCCCCCCCC)(OCC[N+](C)(C)C)(=O)[O-] |
| Chuanxiong | MOL002046 | O=C([O-])CCCCC |
| Chuanxiong | MOL003050 | O=C([O-])CCCCCCCC |
| Chuanxiong | MOL003509 | OCCCCCCCCC |
| Chuanxiong | MOL005577 | O=CCCCCCCCCCC |
| Chuanxiong | MOL005971 | O=C([O-])CCCCCCCCCCCCCCCCCC |
| Chuanxiong | MOL008143 | OC(CCCCCC)C\C=C/CCCCCCCC(=O)[O-] |
| Chuanxiong | MOL010597 | OCCCCCCCCCCCCCCCCCCCCCCCCCCCCCCC |
| Chuanxiong | MOL010610 | OC\C=C\CCC |
| Chuanxiong | MOL010784 | O=C([O-])C(C)C |
| Chuanxiong | MOL011262 | O(C(=O)C)C1CC(C(CC(=O)\C=C(\C(=O)[O-])/C)C)C2(CC=C3C(=CCC4C(C)(C)C(O)CCC34C)C12C)C |
| Chuanxiong | MOL011263 | O(C(=O)C)C1CC(C(CC(=O)\C=C(\C(=O)[O-])/C)C)C2(CC=C3C(=CCC4C(C)(C)C(OC(=O)C)CCC34C)C12C)C |
| Chuanxiong | MOL011264 | O(C(=O)C)C1CCC2(C(CC=C3C2=CCC2(C)C3(C)C(O)CC2C(CC(=O)\C=C(\C(=O)[O-])/C)C)C1(C)C)C |
| Chuanxiong | MOL011265 | O(C(=O)C)C1CCC2(C(CC=C3C2=CCC2(C)C3(CCC2C(CC\C=C(\C(=O)[O-])/C)C)C)C1(C)C)C |
| Chuanxiong | MOL011266 | OC1CCC2(C(CC=C3C2=CCC2(C)C3(CCC2C(CC\C=C(\C=O)/C)C)C)C1(C)C)C |
| Chuanxiong | MOL011267 | O=C1CCC2(C(CC(=O)C3=C2CCC2(C)C3(CCC2C(CC\C=C(\C=O)/C)C)C)C1(C)C)C |
| Chuanxiong | MOL011268 | OC1CCC2(C(CC(=O)C3=C2CCC2(C)C3(CCC2C(CC\C=C(\C=O)/C)C)C)C1(C)C)C |
| Chuanxiong | MOL011269 | O(C(=O)C)C1C2(C)C(C3=C(C1=O)C1(C(CC3O)C(C)(C)C(O)CC1)C)(C)C(=O)CC2C(CCC(=O)[O-])C |
| Chuanxiong | MOL011270 | OC1C2=C(C3(C(C1)C(C)(C)C(=O)CC3)C)C(=O)CC1(C)C2(C)C(=O)CC1C(CCC(=O)[O-])C |
| Chuanxiong | MOL011271 | OC1C2(C)C(C3=C(C1=O)C1(C(CC3O)C(C)(C)C(=O)CC1)C)(C)C(=O)CC2C(CCC(=O)[O-])C |
| Chuanxiong | MOL011272 | OC1C2(C)C(C3=C(C1=O)C1(C(CC3O)C(C)(C)C(O)CC1)C)(C)C(=O)CC2C(CCC(=O)[O-])C |
| Chuanxiong | MOL011273 | O=C1C2(C)C(C3=C(C1=O)C1(C(CC3=O)C(C)(C)C(=O)CC1)C)(C)C(=O)CC2C(CCC(=O)[O-])C |
| Chuanxiong | MOL011274 | O(C(=O)C)C1C2(C)C(C3=C(C1=O)C1(C(CC3=O)C(C)(C)C(=O)CC1)C)(C)C(=O)CC2C(CCC(=O)[O-])C |
| Chuanxiong | MOL011275 | OC1C2(C)C(C3=C(C1=O)C1(C(CC3O)C(C)(C)C(=O)CC1)C)(C)C(=O)CC2C(CCC(=O)[O-])C |
| Chuanxiong | MOL011276 | O(C(=O)C)C1C2(C)C(C3=C(C1=O)C1(C(CC3=O)C(C)(C)C(O)CC1)C)(C)C(=O)CC2C(CCC(=O)[O-])C |
| Chuanxiong | MOL011277 | O=C1CC(C(CCC(=O)[O-])C)C2(CC(=O)C3=C(C12C)C(=O)CC1C(C)(C)C(=O)CCC13C)C |
| Chuanxiong | MOL011278 | OC1CC(C(CCC(=O)[O-])C)C2(CC(=O)C3=C(C12C)C(O)CC1C(CO)(C)C(=O)CCC13C)C |
| Chuanxiong | MOL011279 | OC1CCC2(C(CC(O)C3=C2C(=O)CC2(C)C3(C)C(=O)CC2C(CCC(=O)[O-])C)C1(CO)C)C |
| Chuanxiong | MOL011280 | OC1CCC2(C(CC(=O)C3=C2C(=O)CC2(C)C3(C)C(=O)CC2C(CCC(=O)[O-])C)C1(CO)C)C |
| Chuanxiong | MOL011281 | OC1C2(C)C(C3=C(C1=O)C1(C(CC3=O)C(CO)(C)C(O)CC1)C)(C)C(=O)CC2C(CCC(=O)[O-])C |
| Chuanxiong | MOL011282 | OC1C2(C)C(C3=C(C1=O)C1(C(CC3=O)C(C)(C)C(=O)CC1)C)(C)C(=O)CC2C(CCC(=O)[O-])C |
| Chuanxiong | MOL011283 | OC1C2(C)C(C3=C(C1=O)C1(C(CC3=O)C(C)(C)C(O)CC1)C)(C)C(=O)CC2C(CCC(=O)[O-])C |
| Chuanxiong | MOL011284 | OC1CC(C(CCC(=O)[O-])C)C2(CC(=O)C3=C(C12C)C(O)CC1C(C)(C)C(O)CCC13C)C |
| Chuanxiong | MOL011285 | OC1CCC2(C(CC(O)C3=C2C(=O)CC2(C)C3(C)C(=O)CC2C(CCC(=O)[O-])C)C1(C)C)C |
| Chuanxiong | MOL011286 | OC1C2=C(C3(C(C1)C(C)(C)C(=O)CC3)C)C(=O)CC1(C)C2(C)C(=O)CC1C1(CC(=O)CC1)C |
| Chuanxiong | MOL011287 | OC1CCC2(C(CC(O)C3=C2C(=O)CC2(C)C3(C)C(=O)CC2C(=O)C)C1(C)C)C |
| Chuanxiong | MOL011288 | OC1C2=C(C3(C(C1)C(C)C(=O)CC3)C)C(=O)CC1(C)C2(C)C(=O)CC1C(=O)C |
| Chuanxiong | MOL011289 | OC1CC(C(=O)C)C2(CC(=O)C3=C(C12C)C(O)CC1C(C)(C)C(O)CCC13C)C |
| Chuanxiong | MOL011292 | O(C(=O)C)C1C2(C)C(C3=C(C1=O)C1(C(CC3=O)C(C)(C)C(O)CC1)C)(C)C(=O)CC2C(CCC(=O)[O-])C |
| Chuanxiong | MOL011293 | OC1C2(C)C(C3=C(C1=O)C1(C(CC3=O)C(C)(C)C(O)CC1)C)(C)C(=O)CC2C(CCC(OC)=O)C |
| Chuanxiong | MOL011294 | OC1CC(C(CC(=O)CC(C(OC)=O)C)C)C2(CC(=O)C3C(C12C)C(=O)CC1C(C)(C)C(=O)CCC13C)C |
| Chuanxiong | MOL011295 | OC1CC(C(CC(=O)CC(C(OC)=O)C)C)C2(CC(=O)C3=C(C12C)C(O)CC1C(C)(C)C(=O)CCC13C)C |
| Chuanxiong | MOL011296 | OC1CCC2(C(CC(O)C3=C2C(=O)CC2(C)C3(C)C(=O)CC2C(CC(=O)CC(C(OC)=O)C)C)C1(C)C)C |
| Chuanxiong | MOL011297 | OC1C2=C(C3(C(C1)C(C)(C)C(=O)CC3)C)C(=O)CC1(C)C2(C)C(=O)CC1C(CC(=O)CC(C(OC)=O)C)C |
| Chuanxiong | MOL011298 | O(C(=O)C)C1C2(C)C(C3=C(C1=O)C1(C(CC3=O)C(C)(C)C(=O)CC1)C)(C)C(=O)CC2C(CC(=O)CC(C(OC)=O)C)C |
| Chuanxiong | MOL011299 | OC1C2(C)C(C3=C(C1=O)C1(C(CC3O)C(C)(C)C(O)CC1)C)(C)C(=O)CC2C(CC(=O)CC(C(OC)=O)C)C |
| Chuanxiong | MOL011300 | O(C(=O)C)C1C2(C)C(C3=C(C1=O)C1(C(CC3=O)C(C)(C)C(O)CC1)C)(C)C(=O)CC2C(CC(=O)CC(C(OC)=O)C)C |
| Chuanxiong | MOL011301 | OC1C2(C)C(C3=C(C1=O)C1(C(CC3O)C(C)(C)C(O)CC1)C)(C)C(=O)CC2C(O)(CC(=O)CC(C(OC)=O)C)C |
| Chuanxiong | MOL011302 | OC1CC(C(CC(=O)CC(C(OC)=O)C)C)C2(CC(=O)C3=C(C12C)C(=O)CC1C(C)(C)C(=O)CCC13C)C |
| Chuanxiong | MOL011303 | O=C1CCC2(C(CC(=O)C3=C2CCC2(C)C3(CCC2C(CC\C=C(\C(OC)=O)/C)C)C)C1(C)C)C |
| Chuanxiong | MOL011304 | OC1CC(C(CC\C=C(\C(OC)=O)/C)C)C2(CC=C3C(=CCC4C(C)(C)C(=O)CCC34C)C12C)C |
| Chuanxiong | MOL011305 | OC1C2=C(C3(C(C1)C(C)(C)C(=O)CC3)C)C(=O)CC1(C)C2(C)C(=O)CC1C(CCC(OC)=O)C |
| Chuanxiong | MOL011306 | O(C(=O)C)C1C2(C)C(C3=C(C1=O)C1(C(CC3=O)C(C)(C)C(=O)CC1)C)(C)C(=O)CC2C(CCC(OC)=O)C |
| Chuanxiong | MOL011307 | O(C(=O)C)C1C2(C)C(C3=C(C1=O)C1(C(CC3=O)C(C)(C)C(O)CC1)C)(C)C(=O)CC2C(CCC(OC)=O)C |
| Chuanxiong | MOL011308 | O(C(=O)C)C1C2(C)C(C3=C(C1=O)C1(C(CC3O)C(C)(C)C(O)CC1)C)(C)C(=O)CC2C(CCC(OC)=O)C |
| Chuanxiong | MOL011309 | OC1CC(C(CCC(OC)=O)C)C2(CC(=O)C3=C(C12C)C(O)CC1C(C)(C)C(=O)CCC13C)C |
| Chuanxiong | MOL011311 | O=C([O-])CCC[N+](C)(C)C |
| Chuanxiong | MOL011576 | OC(C)c1ccccc1 |
| Chuanxiong | MOL012345 | SCCCCCCCCCCCCCC(=O)C |
| Chuanxiong | MOL012346 | OC1C(C2CCC3(C(CC=C4C5CC(CCC5(CCC34C)C(=O)[O-])(C)C)C2(CC1O)C)C)(CO)C |
| Chuanxiong | MOL012347 | OC\C=C\C\C=C\CC |
| Mahuang | MOL000002 | [o+]1c2c(cc(O)c1-c1cc(O)c(O)cc1)c(O)cc(O)c2 |
| Mahuang | MOL000008 | O1c2c(C(=O)C=C1c1ccc(O)cc1)c(O)cc(O)c2 |
| Mahuang | MOL000053 | OC(C(O)C(=O)CO)C(O)CO |
| Mahuang | MOL000069 | O=C([O-])CCCCCCCCCCCCCCC |
| Mahuang | MOL000103 | Oc1ccc(cc1)C(=O)[O-] |
| Mahuang | MOL000116 | O=CCCCCCCCC |
| Mahuang | MOL000121 | O=CCCCCCCCCC |
| Mahuang | MOL000131 | O=C([O-])CCCCCCC\C=C/C\C=C/CCCCC |
| Mahuang | MOL000172 | o1cccc1C=O |
| Mahuang | MOL000223 | Oc1cc(ccc1O)\C=C\C(=O)[O-] |
| Mahuang | MOL000254 | O(C)c1cc(ccc1O)CC=C |
| Mahuang | MOL000263 | OC1CCC2(C(CCC3(C2CC=C2C4CC(CCC4(CCC23C)C(=O)[O-])(C)C)C)C1(C)C)C |
| Mahuang | MOL000303 | O=C([O-])CCCCCCC |
| Mahuang | MOL000305 | O=C([O-])CCCCCCCCCCC |
| Mahuang | MOL000346 | O=C([O-])CCC(=O)[O-] |
| Mahuang | MOL000383 | OC(C(O)C(O)C=O)C(O)C(=O)[O-] |
| Mahuang | MOL000420 | OC(C(O)CO)C(O)C=O |
| Mahuang | MOL000432 | O=C([O-])CCCCCCC\C=C/C\C=C/C\C=C/CC |
| Mahuang | MOL000433 | O=C1NC(=Nc2ncc(nc12)CNc1ccc(cc1)C(=O)NC(CCC(=O)[O-])C(=O)[O-])N |
| Mahuang | MOL000472 | Oc1c2c(cc(c1)C)C(=O)c1c(C2=O)c(O)cc(O)c1 |
| Mahuang | MOL000511 | OC1CCC2(C(CCC3(C2CC=C2C4C(C)C(CCC4(CCC23C)C(=O)[O-])C)C)C1(C)C)C |
| Mahuang | MOL000675 | O=C([O-])CCCCCCC\C=C/CCCCCCCC |
| Mahuang | MOL000697 | OC(C(O)CO)CO |
| Mahuang | MOL000705 | O=CCCCCCC |
| Mahuang | MOL000766 | OCCCCCCCCCCCCCCCCCCCCCCCCCC |
| Mahuang | MOL001335 | OCc1ccccc1 |
| Mahuang | MOL001393 | O=C([O-])CCCCCCCCCCCCC |
| Mahuang | MOL001456 | OC(CC(=O)[O-])(CC(=O)[O-])C(=O)[O-] |
| Mahuang | MOL001468 | OC(CC(=O)[O-])C(=O)[O-] |
| Mahuang | MOL001494 | O(C(=O)CCCCCCC\C=C/C\C=C/CCCCC)CC |
| Mahuang | MOL001496 | O(C(=O)CCCCCCCCCCCCC)CC |
| Mahuang | MOL001640 | O=C([O-])CCCCCCCCC |
| Mahuang | MOL001644 | O=CCCCCCCCCCCC |
| Mahuang | MOL001691 | O1C(C(O)CO)C([O-])=C(O)C1=O |
| Mahuang | MOL001729 | Oc1c2c(cc(c1)C)C(=O)c1c(C2=O)c(O)ccc1 |
| Mahuang | MOL001732 | OC(CO)CO |
| Mahuang | MOL001739 | O=C([O-])CCCCCCC\C=C/CCCCCC |
| Mahuang | MOL001818 | O(C(=O)CCCCCCC\C=C\CCCCCC)C |
| Mahuang | MOL001880 | O=C([O-])C(=O)[O-] |
| Mahuang | MOL001886 | OC(C(O)C(=O)[O-])C(=O)[O-] |
| Mahuang | MOL001955 | O(C(=O)\C=C\c1cc(O)c(O)cc1)C1CC(O)(CC(O)C1O)C(=O)[O-] |
| Mahuang | MOL002046 | O=C([O-])CCCCC |
| Mahuang | MOL002213 | O=C(\C=C/c1ccccc1)c1ccccc1 |
| Mahuang | MOL002267 | O1C(CO)C(O)C(O)C(O)C1Oc1c2c(cc(c1)C(=O)[O-])C(=O)c1c(C2=O)c(OC2OC(CO)C(O)C(O)C2O)ccc1 |
| Mahuang | MOL002268 | Oc1c2c(cc(c1)C(=O)[O-])C(=O)c1c(C2=O)c(O)ccc1 |
| Mahuang | MOL002274 | Oc1c2c(cc(c1)C(=O)[O-])C(c1c(C2=O)c(O)ccc1)C1c2c(C(=O)c3c1cccc3O)c(O)cc(c2)C(=O)[O-] |
| Mahuang | MOL002275 | O1C(CO)C(O)C(O)C(O)C1Oc1c2c(ccc1)C(c1c(C2=O)c(O)cc(c1)C(=O)[O-])C1c2c(C(=O)c3c1cccc3OC1OC(CO)C(O)C(O)C1O)c(O)cc(c2)CO |
| Mahuang | MOL002276 | Oc1c2c(cc(c1)C(=O)[O-])C(c1c(C2=O)c(O)ccc1)C1c2c(C(=O)c3c1cccc3O)c(O)cc(c2)CO |
| Mahuang | MOL002278 | O1C(COC(=O)C(=O)[O-])C(O)C(O)C(O)C1Oc1c2c(ccc1)C(c1c(C2=O)c(O)cc(c1)CO)C1c2c(C(=O)c3c1cccc3OC1OC(CO)C(O)C(O)C1O)c(O)cc(c2)C(=O)[O-] |
| Mahuang | MOL002279 | Oc1cc2c([nH]cc2CC[NH3+])cc1 |
| Mahuang | MOL002290 | O1C(CO)C(O)C(O)C(O)C1Oc1c2c(ccc1)C(c1c(C2=O)c(O)cc(c1)C(=O)[O-])C1c2c(C(=O)c3c1cccc3OC1OC(CO)C(O)C(O)C1O)c(O)cc(c2)C(=O)[O-] |
| Mahuang | MOL002292 | O1C(CO)C(O)C(O)C(O)C1Oc1c2c(ccc1)C(c1c(C2=O)c(O)cc(c1)C(=O)[O-])C1c2c(C(=O)c3c1cccc3OC1OC(CO)C(O)C(O)C1O)c(O)cc(c2)CO |
| Mahuang | MOL002293 | Oc1c2c(cc(c1)C(=O)[O-])C(c1c(C2=O)c(O)ccc1)C1c2c(C(=O)c3c1cccc3O)c(O)cc(c2)CO |
| Mahuang | MOL002298 | OC=1C2C(C=CC=1)C(=O)c1c(C2=O)c(O)cc(c1)CO |
| Mahuang | MOL002299 | OC(CC(=O)[O-])C(=O)[O-] |
| Mahuang | MOL002367 | O1C(C(O)C(O)C(O)C1CO)c1c2c(C(=O)c3c(cc(cc3O)C(=O)[O-])C2=O)c(O)cc1 |
| Mahuang | MOL002368 | O1C(CO)C(O)C(O)C(O)C1Oc1c2c(ccc1)C(c1c(C2=O)c(O)cc(c1)C(=O)[O-])C1c2c(C(=O)c3c1cccc3OC1OC(CO)C(O)C(O)C1O)c(O)cc(c2)C(=O)[O-] |
| Mahuang | MOL002369 | Oc1c2c(cc(c1)C(=O)[O-])C(c1c(C2=O)c(O)ccc1)C1c2c(C(=O)c3c1cccc3O)c(O)cc(c2)C(=O)[O-] |
| Mahuang | MOL002373 | O1c2c(cc(O)c(C)c2C)CCC1(CCCC(CCCC(CCCC(C)C)C)C)C |
| Mahuang | MOL002379 | O=CCCCC |
| Mahuang | MOL002382 | Oc1c2c(cc(C)c1C(=O)C)cc(O)cc2O |
| Mahuang | MOL002588 | OC1CCC2(C(CCC3=C2CCC2(C)C3(CCC2C(CCC(C(C)C)=C)C)C)C1(C)C)C |
| Mahuang | MOL002771 | O1c2c(CCC1(CCCC(CCCC(CCCC(C)C)C)C)C)c(C)c(O)c(C)c2C |
| Mahuang | MOL002850 | Oc1c(cc(cc1C(C)(C)C)C)C(C)(C)C |
| Mahuang | MOL002930 | Oc1ccc(cc1)CCO |
| Mahuang | MOL002998 | Oc1ccccc1 |
| Mahuang | MOL003508 | OCCCCCCCCCC |
| Mahuang | MOL004480 | O=C([O-])C |
| Mahuang | MOL004961 | O1c2c(C(=O)C(OC)=C1c1cc(OC)c(O)cc1)c(O)cc(O)c2 |
| Mahuang | MOL005100 | O1c2c(C(=O)CC1c1cc(O)c(OC)cc1)c(O)cc(O)c2 |
| Mahuang | MOL005120 | O=CCCCCCCCCCCCCCCC |
| Mahuang | MOL005121 | O=CCCCCCCCCCCCCCCCCC |
| Mahuang | MOL005270 | OCCCCCCCCCCCCCCCCC |
| Mahuang | MOL005577 | O=CCCCCCCCCCC |
| Mahuang | MOL005732 | O=CCCCCCCCCCCCCCC |
| Mahuang | MOL005797 | O=CCCCCCCCCCCCC |
| Mahuang | MOL005909 | O1c2c(C(=O)C(OC)=C1c1cc(OC)c(O)cc1)c(O)cc(OC)c2 |
| Mahuang | MOL005910 | O1c2c(C(=O)C(OC)=C1c1cc(OC)c(OC)cc1)c(O)cc(OC)c2 |
| Mahuang | MOL005976 | O=CCCCCCCCCCCCCC |
| Mahuang | MOL006077 | s1c[n+](Cc2cnc(nc2N)C)c(C)c1CCO |
| Mahuang | MOL006214 | O=C1CCC2(C3C(C4CCC(C(=O)C)C4(CC3)C)CCC2=C1)C |
| Mahuang | MOL006838 | [o+]1c2c(ccc1-c1ccccc1)cccc2 |
| Mahuang | MOL007914 | O=C1CCC2(C(CC(CC2)C(C)=C)=C1C)C |
| Mahuang | MOL008420 | O(C)c1cc(O)cc(C(=O)[O-])c1C(=O)Cc1ccc(O)cc1 |
| Mahuang | MOL008424 | OC1CCC(=O)c2c1cccc2O |
| Mahuang | MOL009042 | O1C2C(C(=C)C1=O)C(O)C1(C(C=CC1=O)C(C2)C)C |
| Mahuang | MOL010268 | O(C(=O)c1ccccc1C(=O)[O-])C |
| Mahuang | MOL010269 | O=CCCCCCCCCCCCCCCCCCC |
| Mahuang | MOL010270 | O(C(=O)CCCCC)CCCCC |
| Mahuang | MOL010277 | P(OCc1cnc(C)c(O)c1C)(=O)([O-])[O-] |
| Mahuang | MOL010278 | O1c2c(CCC1(CCCC(CCCC(CCCC(C)C)C)C)C)c(C)c(O)c(C)c2C |
| Mahuang | MOL010279 | O1c2c(CC(CCCC(CCCC(CCCC(C)C)C)C)C1C)c(C)c(O)c(C)c2C |
| Mahuang | MOL010280 | O1c2c(CC(CCCC(CCCC(CCCC(C)C)C)C)C1C)c(C)c(O)cc2C |
| Mahuang | MOL010281 | O1c2c(CC(CCCC(CCCC(CCCC(C)C)C)C)C1C)cc(O)cc2C |
| Mahuang | MOL010282 | O1c2c(CC(CCCC(CCCC(CCCC(C)C)C)C)C1C)cc(O)c(C)c2C |
| Renshen | MOL000003 | OC(C(O)C(O)CO)C(O)CO |
| Renshen | MOL000008 | O1c2c(C(=O)C=C1c1ccc(O)cc1)c(O)cc(O)c2 |
| Renshen | MOL000041 | O=C([O-])C([NH3+])Cc1ccccc1 |
| Renshen | MOL000042 | O=C([O-])C([NH3+])C |
| Renshen | MOL000050 | O=C([O-])C[NH3+] |
| Renshen | MOL000052 | O=C([O-])C([NH3+])CCC(=O)[O-] |
| Renshen | MOL000054 | O=C([O-])C([NH3+])CCCNC(=[NH2+])N |
| Renshen | MOL000055 | O=C([O-])C([NH3+])CCCC[NH3+] |
| Renshen | MOL000056 | Oc1ccc(cc1)CC([NH3+])C(=O)[O-] |
| Renshen | MOL000065 | O=C([O-])C([NH3+])CC(=O)[O-] |
| Renshen | MOL000067 | O=C([O-])C([NH3+])C(C)C |
| Renshen | MOL000068 | O=C([O-])C([NH3+])C(CC)C |
| Renshen | MOL000069 | O=C([O-])CCCCCCCCCCCCCCC |
| Renshen | MOL000103 | Oc1ccc(cc1)C(=O)[O-] |
| Renshen | MOL000114 | O(C)c1cc(ccc1O)C(=O)[O-] |
| Renshen | MOL000131 | O=C([O-])CCCCCCC\C=C/C\C=C/CCCCC |
| Renshen | MOL000223 | Oc1cc(ccc1O)\C=C\C(=O)[O-] |
| Renshen | MOL000305 | O=C([O-])CCCCCCCCCCC |
| Renshen | MOL000346 | O=C([O-])CCC(=O)[O-] |
| Renshen | MOL000388 | O=C([O-])CCC[NH3+] |
| Renshen | MOL000394 | OCC[N+](C)(C)C |
| Renshen | MOL000432 | O=C([O-])CCCCCCC\C=C/C\C=C/C\C=C/CC |
| Renshen | MOL000477 | O(C(=O)CCCCCCCCCCC)CC |
| Renshen | MOL000515 | O=C([O-])CCCCCCCCCCCCCCCCCCCCCCCCCCCCC |
| Renshen | MOL000602 | O=C([O-])\C=C\C(=O)[O-] |
| Renshen | MOL000635 | O(C)c1cc(ccc1O)C=O |
| Renshen | MOL000659 | O=C([O-])CCCCCCCCCCCCCCCCCCCCCCCCCCC |
| Renshen | MOL000662 | O=C([O-])CCCCCCCCCCCCCCCCCCCCCCCCC |
| Renshen | MOL000663 | O=C([O-])CCCCCCCCCCCCCCCCCCCCCCC |
| Renshen | MOL000675 | O=C([O-])CCCCCCC\C=C/CCCCCCCC |
| Renshen | MOL000734 | OC(C(O)C(O)C=O)C(O)CO |
| Renshen | MOL000747 | OCC(=O)CCC(=O)[O-] |
| Renshen | MOL000748 | o1c(ccc1C=O)CO |
| Renshen | MOL000749 | O=C([O-])CCCCCCC\C=C\C\C=C\CCCCC |
| Renshen | MOL000753 | O1C(COC2OC(COC3OC(C)C(O)C(O)C3O)C(O)C(O)C2O)C(O)C(O)C(O)C1OC1COC(OC2C(O)C(OC(OC3C(O)C(O)COC3OC3CCC4(C(CCC5(C4CC=C4C6CC(CCC6(CCC45C)C(=O)[O-])(C)C)C)C3(C)C)C)C2O)C)C(O)C1O |
| Renshen | MOL000754 | O1C(C)C(O)C(OC2OCC(O)C(O)C2O)C(O)C1OC1C(O)C(O)COC1OC1CCC2(C(CCC3(C2CC=C2C4CC(CCC4(CCC23C)C(=O)[O-])(C)C)C)C1(C)C)C |
| Renshen | MOL000763 | O1c2cc(c3c(c4c(cc3[N+](=O)[O-])c(OC)cc(O)c4)c2OC1)C(=O)[O-] |
| Renshen | MOL000764 | O(C)c1cc2CC[N+](C3Cc4c(-c(c23)c1O)c(O)c(OC)cc4)(C)C |
| Renshen | MOL000813 | O1C(CO)C(O)C(O)C(O)C1O |
| Renshen | MOL000814 | O1C(CO)C(O)C(O)C(O)C1O |
| Renshen | MOL000860 | O=C([O-])CCCCCCCCCCCCCCCCC |
| Renshen | MOL000971 | O(C(=O)CCCCCCCCCCCCCCC)CC |
| Renshen | MOL001393 | O=C([O-])CCCCCCCCCCCCC |
| Renshen | MOL001398 | O(C(=O)CCCCCCC\C=C/C\C=C/C\C=C/CC)C |
| Renshen | MOL001494 | O(C(=O)CCCCCCC\C=C/C\C=C/CCCCC)CC |
| Renshen | MOL001552 | O(C(=O)C(CO)c1ccccc1)C1CC2[NH+](C(C1)CC2)C |
| Renshen | MOL001553 | O1C2C3[NH+](C(CC(OC(=O)C(CO)c4ccccc4)C3)C12)C |
| Renshen | MOL001554 | O1C2C3[NH+](C(CC(OC(=O)C(CO)c4ccccc4)C3)C12)C |
| Renshen | MOL001640 | O=C([O-])CCCCCCCCC |
| Renshen | MOL001641 | O(C(=O)CCCCCCC\C=C/C\C=C/CCCCC)C |
| Renshen | MOL001739 | O=C([O-])CCCCCCC\C=C/CCCCCC |
| Renshen | MOL001943 | P(OCC(OC(=O)CCCCCCC\C=C/C\C=C/CCCCC)COC(=O)CCCCCCCCCCCCCCC)(OCC[N+](C)(C)C)(=O)[O-] |
| Renshen | MOL001945 | O1c2c(C=CC1=O)c(OC)c1c(occ1)c2 |
| Renshen | MOL001952 | O(C)c1cc(cc(OC)c1O)\C=C\CO |
| Renshen | MOL001955 | O(C(=O)\C=C\c1cc(O)c(O)cc1)C1CC(O)(CC(O)C1O)C(=O)[O-] |
| Renshen | MOL002038 | O=C([O-])CCCCCCC\C=C\C\C=C/CCCCC |
| Renshen | MOL002083 | O1c2c(C(=O)C=C1c1cc(OC)c(O)c(OC)c1)c(O)cc(O)c2 |
| Renshen | MOL002254 | O(C(COC(=O)CCCCCCCCCCCCCCC)COC(=O)CCCCCCCCCCCCCCC)C(=O)CCCCCCCCCCCCCCC |
| Renshen | MOL002439 | O1C23C4(OC1)C1C(C56C(C(CCC5OC)(C[NH+](C26)CC)COC(=O)c2ccccc2N2C(=O)C(CC2=O)C)C3OC)CC(C1OC)C(OC)C4 |
| Renshen | MOL002440 | OC1CC2(C3CC=C4C(CC(O)C(=O)C4(C)C)C3(C)C(=O)CC2(C)C1C(O)(C(=O)\C=C\C(OC(=O)C)(C)C)C)C |
| Renshen | MOL002441 | O1C(CO)C(OC2OC(C)C(O)C(O)C2O)C(O)C(OC2OC(C)C(O)C(O)C2O)C1OC1CC2=CCC3C4CC5OC6(OCC(CC6)C)C(C5C4(CCC3C2(CC1)C)C)C |
| Renshen | MOL002442 | O(C)c1cc(ccc1O)\C=C/C(OC1CC2=CCC3C4CCC(C(CCCC(C)C)C)C4(CCC3C2(CC1)C)C)=O |
| Renshen | MOL002443 | OC1CC2(C3CC=C4C(CCC(O)C4(C)C)C3(CO)C(=O)CC2(C)C1C(O)(C(=O)\C=C\C(OC(=O)C)(C)C)C)C |
| Renshen | MOL002444 | OC1CC2(C3CC=C4C(C=C(O)C(=O)C4(C)C)C3(C)C(=O)CC2(C)C1C(O)(C(=O)\C=C\C(OC(=O)C)(C)C)C)C |
| Renshen | MOL002447 | OC1CC2(C3CC=C4C(CC(O)C(=O)C4(C)C)C3(C)C(=O)CC2(C)C1C(O)(C(=O)\C=C\C(O)(C)C)C)C |
| Renshen | MOL002682 | OC(C(O)C(O)C=O)C(O)CO |
| Renshen | MOL003050 | O=C([O-])CCCCCCCC |
| Renshen | MOL003955 | S(SCC([NH3+])C(=O)[O-])CC([NH3+])C(=O)[O-] |
| Renshen | MOL003969 | OCC([NH3+])C(=O)[O-] |
| Renshen | MOL003971 | OC(C([NH3+])C(=O)[O-])C |
| Renshen | MOL004285 | O(C(=O)c1ccccc1C(=O)[O-])CCCC |
| Renshen | MOL004691 | OC(C(O)CO)C(O)C=O |
| Renshen | MOL004739 | O=C([O-])C([NH3+])C |
| Renshen | MOL004743 | O(Cc1ccccc1)C(=O)NC(CC(C)C)C(=O)[O-] |
| Renshen | MOL004752 | O(C(=O)CCCCCCCCCCCC)CC |
| Renshen | MOL005448 | O=C([O-])C([NH3+])CC(C)C |
| Renshen | MOL005449 | S(CCC([NH3+])C(=O)[O-])C |
| Renshen | MOL005530 | O1c2c(C(=O)C=C1c1cc(O)c(O)cc1)c(O)cc(OC)c2 |
| Renshen | MOL007177 | SCC([NH3+])C(=O)[O-] |
| Renshen | MOL009087 | OC1CC(C2CC=C(CCC12C)C(OC)=O)CC(OC)=O |
| Renshen | MOL009091 | O(C)c1cc(O)c(C(=O)\C=C\c2ccc(O)cc2)c(O)c1C\C=C(\C)/C |
| Renshen | MOL009294 | OC(Cc1ccccc1)C(=O)[O-] |
| Renshen | MOL009295 | o1c(ccc1CO)-c1nc(cc2c1[nH]c1c2cccc1)C(=O)[O-] |
| Renshen | MOL009296 | O1C2C(CCC(C3C2C(CC3)=C)=C)C(C[NH+]2CCCC2C(=O)[O-])C1=O |
| Renshen | MOL009297 | O1c2c(c(O)c(OC)c(O)c2)C(=O)C=C1c1cc(OC)c(O)cc1 |
| Renshen | MOL009300 | O1C2C(C(C)C1=O)C(O)CC(C1C2C(CC1)=C)=C |
| Renshen | MOL009301 | OC(C(O)CCO)C(O)CCO |
| Renshen | MOL009658 | O=C([O-])C([NH3+])CCC(=O)[O-] |
| Renshen | MOL010336 | O=C([O-])C([NH3+])CC(=O)[O-] |
| Renshen | MOL010337 | O(C)c1c2c3c4c(c1O)C(=O)C=C(OC)c4c1c4c(c(O)c(OC)c(CC(O)(C)C2C(=O)C)c34)C(=O)C=C1OC |
| Renshen | MOL010338 | O(C)C=1C(O)=C2c3c4c(c(OC)cc3O)c(OC)cc3c4C=4C(CC(=C(C2=4)C=1C(=O)C)C)=C(OC)C(=O)C3=O |
| Renshen | MOL010339 | O(C)C=1C(O)=C2C3=C4C(=C(OC)CC3=O)C(OC)=CC3=C4C=4C(CC(=C(C2=4)C=1C(=O)C)C)=C(OC)C(O)C3=O |
| Renshen | MOL010340 | O(C)C1=C2C=3C=4C(c5c6c(C(OC)=CC(C=36)=C(O)C1=O)c(OC)cc5O)C(=O)C(OC)=CC=4C(O)(C)C2C(=O)C |
| Renshen | MOL010341 | O(C)C1=C2c3c4C(CC(O)(C)C2C(=O)C)=C(OC)C(=O)c2c4c(c4c3c(C1=O)c(O)cc4OC)c(OC)cc2O |
| Renshen | MOL010342 | O(C)C1=C2c3c4C(CC(C)=C2C(=O)C)=C(OC)C(=O)c2c4c(c4c3c(C1=O)c(O)cc4OC)c(OC)cc2O |
| Lingzhi | MOL000069 | O=C([O-])CCCCCCCCCCCCCCC |
| Lingzhi | MOL000105 | Oc1cc(ccc1O)C(=O)[O-] |
| Lingzhi | MOL000116 | O=CCCCCCCCC |
| Lingzhi | MOL000128 | O(C(=O)C)C\C=C(\CC\C=C(\C)/C)/C |
| Lingzhi | MOL000131 | O=C([O-])CCCCCCC\C=C/C\C=C/CCCCC |
| Lingzhi | MOL000219 | O=C([O-])c1ccccc1 |
| Lingzhi | MOL000224 | Oc1cc(ccc1O)CCC(=O)\C=C\C=C\c1cc(O)c(O)cc1 |
| Lingzhi | MOL000225 | Oc1cc(ccc1O)\C=C\C1CC(O)CCc2c1cc(O)c(O)c2 |
| Lingzhi | MOL000226 | O=C(\C=C(/[O-])\C=C\c1ccccc1)CCc1ccccc1 |
| Lingzhi | MOL000228 | O1c2c(C(=O)CC1c1ccccc1)c(OC)cc(O)c2 |
| Lingzhi | MOL000229 | O(C)c1cc(O)cc(O)c1C(=O)\C=C\c1ccccc1 |
| Lingzhi | MOL000230 | O1c2c(C(=O)CC1c1ccccc1)c(O)cc(c2)CO |
| Lingzhi | MOL000238 | OC(\C=C\c1ccccc1)CC(=O)CCc1ccccc1 |
| Lingzhi | MOL000239 | O1c2c(C(=O)C(OC)=C1c1ccc(O)cc1)c(O)cc(OC)c2 |
| Lingzhi | MOL000241 | O(C)c1cc(O)cc(O)c1C(=O)\C=C/c1ccccc1 |
| Lingzhi | MOL000242 | O1c2c(C(=O)CC1c1cc(O)c(O)cc1)c(O)cc(OC)c2 |
| Lingzhi | MOL000243 | O1C2(OOC(O)(C3C2C(CC3)C)C)C(=CC1=O)C(C)C |
| Lingzhi | MOL000245 | O1C(=CC(OC)=CC1=O)\C=C\c1ccccc1 |
| Lingzhi | MOL000246 | O1c2c(C(=O)CC1c1ccccc1)c(O)cc(O)c2 |
| Lingzhi | MOL000249 | O(C(=O)\C=C\c1ccccc1)C |
| Lingzhi | MOL000250 | O=C\C=C/c1ccccc1 |
| Lingzhi | MOL000251 | O1c2c(C(=O)C(O)=C1c1ccc(O)cc1)c(O)cc(OC)c2 |
| Lingzhi | MOL000254 | O(C)c1cc(ccc1O)CC=C |
| Lingzhi | MOL000255 | O(C(COC(=O)CCCCCCCCCCCCC)COC(=O)CCCCCCCCCCCCC)C(=O)CCCCCCCCCCCCC |
| Lingzhi | MOL000256 | O(C(COC(=O)CCCCCCC\C=C/CCCCCCCC)COC(=O)CCCCCCC\C=C/CCCCCCCC)C(=O)CCCCCCC\C=C/CCCCCCCC |
| Lingzhi | MOL000258 | O1c2c(CC1c1cc(OC)c(O)cc1)cc(cc2OC)\C=C\C |
| Lingzhi | MOL000259 | Oc1cc(ccc1C)C(C)C |
| Lingzhi | MOL000263 | OC1CCC2(C(CCC3(C2CC=C2C4CC(CCC4(CCC23C)C(=O)[O-])(C)C)C)C1(C)C)C |
| Lingzhi | MOL000303 | O=C([O-])CCCCCCC |
| Lingzhi | MOL000305 | O=C([O-])CCCCCCCCCCC |
| Lingzhi | MOL000346 | O=C([O-])CCC(=O)[O-] |
| Lingzhi | MOL000360 | O(C)c1cc(ccc1O)\C=C\C(=O)[O-] |
| Lingzhi | MOL000384 | OC(C(O)C(O)C=O)C(O)C(=O)[O-] |
| Lingzhi | MOL000392 | O1C=C(C(=O)c2c1cc(O)cc2)c1ccc(OC)cc1 |
| Lingzhi | MOL000399 | O=C([O-])CCCCCCCCCCCCCCCCCCCCC |
| Lingzhi | MOL000432 | O=C([O-])CCCCCCC\C=C/C\C=C/C\C=C/CC |
| Lingzhi | MOL000579 | Oc1ccc(O)cc1 |
| Lingzhi | MOL000675 | O=C([O-])CCCCCCC\C=C/CCCCCCCC |
| Lingzhi | MOL000676 | O(C(=O)c1ccccc1C(OCCCC)=O)CCCC |
| Lingzhi | MOL000748 | o1c(ccc1C=O)CO |
| Lingzhi | MOL000749 | O=C([O-])CCCCCCC\C=C\C\C=C\CCCCC |
| Lingzhi | MOL000860 | O=C([O-])CCCCCCCCCCCCCCCCC |
| Lingzhi | MOL001050 | Oc1cc(ccc1)C |
| Lingzhi | MOL001227 | O(C(=O)C)CCCCCCCCCC |
| Lingzhi | MOL001285 | OCCCCCCCC |
| Lingzhi | MOL001306 | Oc1ccc(cc1C(=O)C)C |
| Lingzhi | MOL001311 | O=C([O-])CCCCCCC\C=C\C=C\CCCCCC |
| Lingzhi | MOL001312 | O=C([O-])CCCCCCC\C=C\CCCCCC |
| Lingzhi | MOL001313 | OC(CCCCCCCC)C(O)\C=C\C(O)CCCCC(=O)[O-] |
| Lingzhi | MOL001314 | O=C([O-])CCCCCCCC(=O)[O-] |
| Lingzhi | MOL001393 | O=C([O-])CCCCCCCCCCCCC |
| Lingzhi | MOL001396 | O=C([O-])CCCCCCCCCCCCCC |
| Lingzhi | MOL001494 | O(C(=O)CCCCCCC\C=C/C\C=C/CCCCC)CC |
| Lingzhi | MOL001501 | O=C([O-])CCCCCCCCCCCCCCCC |
| Lingzhi | MOL001631 | O=C([O-])CCCCCCCCCCC\C=C/CCCCCCCC |
| Lingzhi | MOL001739 | O=C([O-])CCCCCCC\C=C/CCCCCC |
| Lingzhi | MOL001752 | O(C)c1cc(ccc1O)C=C |
| Lingzhi | MOL001792 | O1c2c(ccc(O)c2)C(=O)CC1c1ccc(O)cc1 |
| Lingzhi | MOL001943 | P(OCC(OC(=O)CCCCCCC\C=C/C\C=C/CCCCC)COC(=O)CCCCCCCCCCCCCCC)(OCC[N+](C)(C)C)(=O)[O-] |
| Lingzhi | MOL001945 | O1c2c(C=CC1=O)c(OC)c1c(occ1)c2 |
| Lingzhi | MOL001950 | O1c2c(C=CC1=O)cc1c(occ1)c2 |
| Lingzhi | MOL002046 | O=C([O-])CCCCC |
| Lingzhi | MOL002154 | OC\C=C\CCCCCC |
| Lingzhi | MOL002379 | O=CCCCC |
| Lingzhi | MOL002558 | O1c2cc(O)ccc2C=CC1=O |
| Lingzhi | MOL002818 | Oc1ccc(cc1)C(=O)C |
| Lingzhi | MOL002830 | Oc1ccc(cc1)C |
| Lingzhi | MOL002850 | Oc1c(cc(cc1C(C)(C)C)C)C(C)(C)C |
| Lingzhi | MOL003050 | O=C([O-])CCCCCCCC |
| Lingzhi | MOL003177 | O(C)c1cc(cc(OC)c1O)C=O |
| Lingzhi | MOL003484 | c12c3c(ccc1cccc2)cccc3 |
| Lingzhi | MOL004102 | Oc1ccccc1\C=C\C(=O)[O-] |
| Lingzhi | MOL004464 | O(C)c1ccc(O)cc1 |
| Lingzhi | MOL004687 | O=C(CCCCCC)C |
| Lingzhi | MOL005319 | O(C(=O)c1ccccc1C(OC(C)(C)C)=O)C(C)(C)C |
| Lingzhi | MOL007344 | P(OCC([NH3+])C(=O)[O-])(OCC(OC(=O)CCC)COC(=O)CC)(=O)[O-] |
| Lingzhi | MOL007349 | P(OC1C(O)C(O)C(O)C(O)C1O)(OCC(OC(=O)CCCCCCCCCCCCCCCCC)COC(=O)CCCCCCCCCCCCCCCCC)(=O)[O-] |
| Lingzhi | MOL007887 | O(CCCCCC)C=O |
| Lingzhi | MOL008326 | O(C(=O)CCCCCCCCCCCC\C=C\CC=C)C |
| Lingzhi | MOL008328 | O(\C=C\C(=O)[O-])C(CCCCCCCCCCCC)C |
| Lingzhi | MOL008329 | OC1CC(CCC1C)C(C)=C |
| Lingzhi | MOL008331 | O(C(CCCCCCCCC)CC)C(=O)C=C |
| Lingzhi | MOL008332 | O(C(CCCCCCCCCC)CCCC)C(=O)C=C |
| Lingzhi | MOL008333 | O(\C=C/CCCCC(CCCCCCC)C)C(=O)C |
| Lingzhi | MOL008335 | OC\C=C/C\C=C/CCCCCCCCC |
| Lingzhi | MOL008337 | O=C([O-])CCCCCCCC1CC1 |
| Lingzhi | MOL008338 | OCCNC(=O)CCCCCCCCC |
| Lingzhi | MOL008340 | O(C(=O)C)CCCCCC(OCC)=O |
| Lingzhi | MOL008341 | OCCNC(=O)CCCCCCC |
| Lingzhi | MOL010886 | OC(\C=C\CCCCCCCCCCCCC)C([NH3+])CO |
| Lingzhi | MOL012937 | O=C([O-])\C=C\C=C\CCCCCCCCCCCCC |
| Lingzhi | MOL012938 | P(OCC(NC(=O)CCCCCCC\C=C/CCCCCCCC)C(O)\C=C\CCCCCCCCCCCCC)(OCC[N+](C)(C)C)(=O)[O-] |
| Lajiao | MOL000002 | [o+]1c2c(cc(O)c1-c1cc(O)c(O)cc1)c(O)cc(O)c2 |
| Lajiao | MOL000003 | OC(C(O)C(O)CO)C(O)CO |
| Lajiao | MOL000012 | O=C([O-])CCCCCCCCCCCCCCCCCCC |
| Lajiao | MOL000069 | O=C([O-])CCCCCCCCCCCCCCC |
| Lajiao | MOL000105 | Oc1cc(ccc1O)C(=O)[O-] |
| Lajiao | MOL000114 | O(C)c1cc(ccc1O)C(=O)[O-] |
| Lajiao | MOL000206 | O(C)c1cc(ccc1O)\C=C\C |
| Lajiao | MOL000210 | Oc1ccc(cc1-c1cc(ccc1O)CC=C)CC=C |
| Lajiao | MOL000211 | OC1CCC2(C(CCC3(C2CCC2C4C(CCC23C)(CCC4C(C)=C)C(=O)[O-])C)C1(C)C)C |
| Lajiao | MOL000212 | O(\C=C/1\CCC23C(CCC4C2(CCC2(C)C4(CCC2C(CC\C=C(/C(=O)[O-])\C)C)C)C3)C\1(C)C)C(=O)C |
| Lajiao | MOL000213 | O(C)c1c(C(Oc2cc(C)c(C(=O)[O-])c(O)c2C)=O)c(cc(OC)c1C)C |
| Lajiao | MOL000214 | O=C1CCC23C(CCC4C2(CCC2(C)C4(CCC2C(CC\C=C(\C(=O)[O-])/C)C)C)C3)C1(C)C |
| Lajiao | MOL000223 | Oc1cc(ccc1O)\C=C\C(=O)[O-] |
| Lajiao | MOL000254 | O(C)c1cc(ccc1O)CC=C |
| Lajiao | MOL000263 | OC1CCC2(C(CCC3(C2CC=C2C4CC(CCC4(CCC23C)C(=O)[O-])(C)C)C)C1(C)C)C |
| Lajiao | MOL000346 | O=C([O-])CCC(=O)[O-] |
| Lajiao | MOL000399 | O=C([O-])CCCCCCCCCCCCCCCCCCCCC |
| Lajiao | MOL000414 | Oc1cc(ccc1O)\C=C/C(=O)[O-] |
| Lajiao | MOL000481 | O1C=C(C(=O)c2c1cc(O)cc2O)c1ccc(O)cc1 |
| Lajiao | MOL000663 | O=C([O-])CCCCCCCCCCCCCCCCCCCCCCC |
| Lajiao | MOL000675 | O=C([O-])CCCCCCC\C=C/CCCCCCCC |
| Lajiao | MOL000676 | O(C(=O)c1ccccc1C(OCCCC)=O)CCCC |
| Lajiao | MOL000708 | O=Cc1ccccc1 |
| Lajiao | MOL000714 | O=CCc1ccccc1 |
| Lajiao | MOL000860 | O=C([O-])CCCCCCCCCCCCCCCCC |
| Lajiao | MOL000861 | OCCCCCCCCCCCCCCCCCCCCCC |
| Lajiao | MOL000924 | O=C(CCCCCCCCC)C |
| Lajiao | MOL000971 | O(C(=O)CCCCCCCCCCCCCCC)CC |
| Lajiao | MOL000983 | OCCCCCCCCCCCCCCCCCCCCCCCCCCCCCC |
| Lajiao | MOL001301 | O=C([O-])\C=C/c1ccccc1 |
| Lajiao | MOL001335 | OCc1ccccc1 |
| Lajiao | MOL001393 | O=C([O-])CCCCCCCCCCCCC |
| Lajiao | MOL001398 | O(C(=O)CCCCCCC\C=C/C\C=C/C\C=C/CC)C |
| Lajiao | MOL001445 | O(C)c1cc(cc(OC)c1O)C(OCCCCNC(=[NH2+])N)=O |
| Lajiao | MOL001501 | O=C([O-])CCCCCCCCCCCCCCCC |
| Lajiao | MOL001552 | O(C(=O)C(CO)c1ccccc1)C1CC2[NH+](C(C1)CC2)C |
| Lajiao | MOL001641 | O(C(=O)CCCCCCC\C=C/C\C=C/CCCCC)C |
| Lajiao | MOL001645 | O(C(=O)C)CCCCCCCC\C=C\C\C=C\CCCCC |
| Lajiao | MOL001668 | O1C(CO)C(O)C(O)C(O)C1OC1OC=C(C2C1C(=CC2)CO)C(=O)[O-] |
| Lajiao | MOL001669 | O1C=C(C2C(C(=CC2)CO)C1O)C(=O)[O-] |
| Lajiao | MOL001732 | OC(CO)CO |
| Lajiao | MOL001739 | O=C([O-])CCCCCCC\C=C/CCCCCC |
| Lajiao | MOL001955 | O(C(=O)\C=C\c1cc(O)c(O)cc1)C1CC(O)(CC(O)C1O)C(=O)[O-] |
| Lajiao | MOL001965 | O(c1cc(ccc1O)CC1[NH+](CCc2c1cc(OC)c(OC)c2)C)c1ccc(cc1)CC1[NH+](CCc2c1cc(OC)c(OC)c2)C |
| Lajiao | MOL002038 | O=C([O-])CCCCCCC\C=C\C\C=C/CCCCC |
| Lajiao | MOL002067 | Oc1c2c3c(c4c5c(C(=O)c6c7c(c8c(c(C2=O)c(O)cc8O)c3c57)c(O)cc6O)c(O)cc4C)c(c1)C |
| Lajiao | MOL002211 | O=C([O-])CCCCCCCCC\C=C\C\C=C\CCCCC |
| Lajiao | MOL002212 | OC12C=3C(CCC1(C)C(CC2)C(O)(C(O)CCC(O)(C)C)C)C1(C(CC(O)C(O)C1)C(=O)C=3)C |
| Lajiao | MOL002213 | O=C(\C=C/c1ccccc1)c1ccccc1 |
| Lajiao | MOL002214 | O1C(C(=O)[O-])C(O)C(OC2OCC(O)C(O)C2O)C(O)C1OC1CCC2(C(CCC3(C2CC=C2C4CC(CCC4(CCC23C)C(=O)[O-])(C)C)C)C1(C)C)C |
| Lajiao | MOL002215 | O=C([O-])C12C(C3=CCC4C(CCC5C(CCCC45C)(C)C)(C)C3(CC1)C)CC(CC2)(C)C |
| Lajiao | MOL002622 | OC=1C(C)(C)C(=O)C(C(=O)CCC)=C([O-])C=1Cc1c(O)c(CC=2C([O-])=C(C(=O)CCC)C(=O)C(C)(C)C=2O)c(O)c(C(=O)CCC)c1O |
| Lajiao | MOL002850 | Oc1c(cc(cc1C(C)(C)C)C)C(C)(C)C |
| Lajiao | MOL002930 | Oc1ccc(cc1)CCO |
| Lajiao | MOL003202 | O1C(CO)C(O)C(O)C(O)C1OC1OC=C(C2C1C(C)C(O)C2)C(=O)[O-] |
| Lajiao | MOL003203 | O1C=C(C2C(C(C)C(O)C2)C1O)C(=O)[O-] |
| Lajiao | MOL003493 | c12c(cccc1)cccc2 |
| Lajiao | MOL003509 | OCCCCCCCCC |
| Lajiao | MOL003975 | O(C(=O)CCCCCCCCC\C=C\C\C=C\C\C=C\CC)C |
| Lajiao | MOL003992 | O=C(CCCCCCCCCCCCC)C |
| Lajiao | MOL004082 | O(C(COC(=O)CCCCCCCCCCCCCCCCC)COC(=O)CCCCCCCCCCCCCCCCC)C(=O)CCCCCCCCCCCCCCCCC |
| Lajiao | MOL004459 | OCCCCCCCCCCCCC |
| Lajiao | MOL004647 | O=C([O-])CCCCCCCCCCCC |
| Lajiao | MOL005270 | OCCCCCCCCCCCCCCCCC |
| Lajiao | MOL005320 | O=C([O-])CCC\C=C\C\C=C\C\C=C\C\C=C\CCCCC |
| Lajiao | MOL005810 | O(C(=O)CCCCCCCCCCCCCCC)CCCCCCCCCCCC |
| Lajiao | MOL006378 | O(C(=O)\C=C\c1cc(O)c(O)cc1)C1CC(O)(CC(O)C1O)C(=O)[O-] |
| Lajiao | MOL006549 | O1C(CO)C(OC(=O)\C=C/c2cc(O)c(O)cc2)C(O)C(O)C1OCCc1cc(O)c(O)cc1 |
| Lajiao | MOL007647 | S(SSC)CCC |
| Lajiao | MOL007861 | O(C\C=C(\C)/C)c1cc(O)c(CC=2C([O-])=C(C(=O)C(C)C)C(=O)C(C)(C)C=2O)c(O)c1C(=O)c1ccccc1 |
| Lajiao | MOL007862 | O1c2c(C=CC1(C)C)c(O)c(CC=1C([O-])=C(C(=O)C(C)C)C(=O)C(C)(C)C=1O)c(O)c2C(=O)C(C)C |
| Lajiao | MOL007863 | OC=1C(C)(C)C(=O)C(C(=O)C(C)C)=C([O-])C=1Cc1c(O)c(C(=O)C(C)C)c(O)c(C)c1O |
| Lajiao | MOL007864 | OC=1C(C)(C)C(=O)C(C(=O)C(C)C)=C([O-])C=1Cc1c(O)c(C(=O)C(CC)C)c(O)c(C)c1O |
| Lajiao | MOL007865 | OC=1C(C)(C)C(=O)C(C(=O)C(CC)C)=C([O-])C=1Cc1c(O)c(C(=O)C(CC)C)c(O)c(C)c1O |
| Lajiao | MOL007866 | OC=1C(C)(C)C(=O)C(C(=O)C(C)C)=C([O-])C=1CC=1C([O-])=C(C(=O)C(C)C)C(=O)C(C)(C)C=1O |
| Lajiao | MOL007867 | O1c2c(C=CC1(CC\C=C(\C)/C)C)c(O)c(CC=1C(=O)C(C(=O)C(C)C)=C([O-])C(C)(C)C=1[O-])c(O)c2C(=O)C(C)C |
| Lajiao | MOL007868 | O(C\C=C(\C)/C)c1cc(O)c(CC=2C(=O)C(C(=O)C(C)C)=C([O-])C(C)(C)C=2[O-])c(O)c1C(=O)c1ccccc1 |
| Lajiao | MOL007869 | Oc1c(CC=2C(=O)C(C(=O)C(C)C)=C([O-])C(C)(C)C=2[O-])c(O)c(C(=O)C(C)C)c(O)c1CC=1C(=O)C(C(=O)C(C)C)=C([O-])C(C)(C)C=1[O-] |
| Lajiao | MOL007870 | Oc1c(CC=2C(=O)C(C(=O)C(C)C)=C([O-])C(C)(C)C=2[O-])c(O)c(C\C=C(\CC\C=C(\C)/C)/C)c(O)c1C(=O)C(C)C |
| Lajiao | MOL007871 | OC=1C(C)(C)C(=O)C(C(=O)C(C)C)=C([O-])C=1Cc1c(O)c(C(=O)c2ccccc2)c(O)c(C\C=C(\CC\C=C(\C)/C)/C)c1O |
| Lajiao | MOL007874 | O(C(=O)CCCCCCCCCCCCCCCCC)CCCCCCCCCCCC |
| Lajiao | MOL007877 | O(CC(=O)[O-])CCOCCOCCOCCOCCOCCOCCOCCOCCOCCOC |
| Lajiao | MOL007880 | O1c2c(C(O)CC1c1cc(O)cc(O)c1)c(O)cc(O)c2 |
| Lajiao | MOL007916 | Oc1c2c(-c3c(CC2)c(C)c(O)cc3)c(cc1C)C(O)C |
| Lajiao | MOL007917 | O(C)c1ccc2-c3c(CCc2c1C)c(O)cc(C)c3C=C |
| Lajiao | MOL007918 | O(C)c1cc(c-2c(CCc3c-2ccc(O)c3C)c1C)C=C |
| Lajiao | MOL007919 | Oc1ccc-2c(CCc3c-2c(cc(c3)C(=O)[O-])C=C)c1C |
| Lajiao | MOL008284 | O=C([O-])CCC |
| Lajiao | MOL008401 | O=C([O-])CCCCCCCCCCCCCCCCCCCC |
| Lajiao | MOL008852 | O=C([O-])C1(C2CC=C3C(CCC(=C3)C(C)C)C2(CCC1)C)C |
| Lajiao | MOL012679 | O=C(CCCCCCCCCCC)CC |
| Danshen | MOL000008 | O1c2c(C(=O)C=C1c1ccc(O)cc1)c(O)cc(O)c2 |
| Danshen | MOL000069 | O=C([O-])CCCCCCCCCCCCCCC |
| Danshen | MOL000105 | Oc1cc(ccc1O)C(=O)[O-] |
| Danshen | MOL000121 | O=CCCCCCCCCC |
| Danshen | MOL000219 | O=C([O-])c1ccccc1 |
| Danshen | MOL000223 | Oc1cc(ccc1O)\C=C\C(=O)[O-] |
| Danshen | MOL000254 | O(C)c1cc(ccc1O)CC=C |
| Danshen | MOL000259 | Oc1cc(ccc1C)C(C)C |
| Danshen | MOL000263 | OC1CCC2(C(CCC3(C2CC=C2C4CC(CCC4(CCC23C)C(=O)[O-])(C)C)C)C1(C)C)C |
| Danshen | MOL000305 | O=C([O-])CCCCCCCCCCC |
| Danshen | MOL000346 | O=C([O-])CCC(=O)[O-] |
| Danshen | MOL000360 | O(C)c1cc(ccc1O)\C=C\C(=O)[O-] |
| Danshen | MOL000471 | Oc1c2c(cc(c1)CO)C(=O)c1c(C2=O)c(O)ccc1 |
| Danshen | MOL000472 | Oc1c2c(cc(c1)C)C(=O)c1c(C2=O)c(O)cc(O)c1 |
| Danshen | MOL000476 | O(C)c1cc(O)c2c(c1)C(=O)c1c(C2=O)c(O)cc(c1)C |
| Danshen | MOL000511 | OC1CCC2(C(CCC3(C2CC=C2C4C(C)C(CCC4(CCC23C)C(=O)[O-])C)C)C1(C)C)C |
| Danshen | MOL000552 | O1c2c(c(O)c(OC)c(OC)c2OC)C(=O)C=C1c1ccccc1O |
| Danshen | MOL000553 | [NH+]1(C2Nc3c(cccc3)C2(CC1)C12CC[NH+](C1Nc1c2cccc1)C)C |
| Danshen | MOL000554 | O1C(COC(=O)c2cc(O)c(O)c(O)c2)C(O)C(O)C(O)C1Oc1cc(cc(O)c1O)C(=O)[O-] |
| Danshen | MOL000555 | O(C(=O)C=1C[NH+](CCC=1)C)CC |
| Danshen | MOL000557 | O1C(CO)C(O)C(O)C(O)C1Oc1cc(ccc1O)C(=O)[O-] |
| Danshen | MOL000559 | O1C(C(=O)[O-])C(c2c(cc(O)c(O)c2O)C1=O)/C(=C/C(=O)[O-])/C(OC1C2OC(=O)c3c(-c4c(cc(O)c(O)c4O)C(OCC1OC(OC(=O)c1cc(O)c(O)c(O)c1)C2O)=O)c(O)c(O)c(O)c3)=O |
| Danshen | MOL000560 | Oc1ccc(O)cc1C |
| Danshen | MOL000569 | O(C(=O)c1cc(O)c(O)c(O)c1)c1cc(cc(O)c1O)C(=O)[O-] |
| Danshen | MOL000570 | N(c1cc2c(cc1)cccc2)c1ccccc1 |
| Danshen | MOL000572 | Oc1c2CC(=CCc2c(O)cc1C)C |
| Danshen | MOL000675 | O=C([O-])CCCCCCC\C=C/CCCCCCCC |
| Danshen | MOL000771 | Oc1ccc(cc1)\C=C\C(=O)[O-] |
| Danshen | MOL000794 | O(C)c1c2-c3c(CC4[N+](CCc(cc1OC)c24)(C)C)ccc(OC)c3O |
| Danshen | MOL001227 | O(C(=O)C)CCCCCCCCCC |
| Danshen | MOL001439 | O=C([O-])CCC\C=C/C\C=C/C\C=C/C\C=C/CCCCC |
| Danshen | MOL001456 | OC(CC(=O)[O-])(CC(=O)[O-])C(=O)[O-] |
| Danshen | MOL001468 | OC(CC(=O)[O-])C(=O)[O-] |
| Danshen | MOL001501 | O=C([O-])CCCCCCCCCCCCCCCC |
| Danshen | MOL001640 | O=C([O-])CCCCCCCCC |
| Danshen | MOL001689 | O1c2c(C(=O)C=C1c1ccc(OC)cc1)c(O)cc(O)c2 |
| Danshen | MOL001729 | Oc1c2c(cc(c1)C)C(=O)c1c(C2=O)c(O)ccc1 |
| Danshen | MOL001733 | O1c2c(c(O)c(OC)c(OC)c2)C(=O)C=C1c1cc(O)c(OC)cc1 |
| Danshen | MOL001739 | O=C([O-])CCCCCCC\C=C/CCCCCC |
| Danshen | MOL001886 | OC(C(O)C(=O)[O-])C(=O)[O-] |
| Danshen | MOL001965 | O(c1cc(ccc1O)CC1[NH+](CCc2c1cc(OC)c(OC)c2)C)c1ccc(cc1)CC1[NH+](CCc2c1cc(OC)c(OC)c2)C |
| Danshen | MOL002046 | O=C([O-])CCCCC |
| Danshen | MOL002295 | O=C([O-])\C=C\c1ccccc1 |
| Danshen | MOL002328 | O1C(Cc2c(C1=O)c(O)cc(O)c2)Cc1cc(O)cc(O)c1C(=O)C |
| Danshen | MOL002343 | O1c2c3C([NH+](CCc3cc(OC)c2OC)C)Cc2cc(Oc3ccc(CC4[NH+](CCc5cc(OC)c1cc45)C)cc3)c(OC)cc2 |
| Danshen | MOL002558 | O1c2cc(O)ccc2C=CC1=O |
| Danshen | MOL002836 | O(C)c1ccc(cc1)C=O |
| Danshen | MOL002891 | O(C)c1cc2CC[N+](C3Cc4c(-c(c23)c1O)c(O)c(OC)cc4)(C)C |
| Danshen | MOL004328 | O1c2c(C(=O)CC1c1ccc(O)cc1)c(O)cc(O)c2 |
| Danshen | MOL004647 | O=C([O-])CCCCCCCCCCCC |
| Danshen | MOL005038 | O1c2c(c(C)c(O)c(O)c2)C(=O)C=C1CC(=O)C |
| Danshen | MOL005045 | OC1CC(c2c(C1)cc(O)cc2O)C |
| Danshen | MOL005049 | O1C(=CC(OC)=CC1=O)c1c(cc(O)cc1O)C |
| Danshen | MOL005054 | O1c2c(c(cc(O)c2)C)C(=O)C=C1CC(=O)C |
| Danshen | MOL005055 | Oc1c2c(cc(c1)C)C(=O)c1c(C2=O)c(O)ccc1O |
| Danshen | MOL005056 | O1c2c(CC(CCCC(CCCC(CCCC(C)C)C)C)C1C)c(C)c(O)c(C)c2C |
| Danshen | MOL005057 | O=C([O-])\C=C\C=C\CCCCCCCCCCC |
| Danshen | MOL005059 | O1C(c2c(cc3c(c2OC)c(O)ccc3)C1=O)C |
| Danshen | MOL005062 | Oc1c2c(cc3c1cccc3)cccc2 |
| Danshen | MOL005573 | O1c2c(C(=O)C=C1c1ccc(O)cc1)c(O)cc(OC)c2 |
| Danshen | MOL005944 | O=C1N2C(C3C4[NH+](CCC3)CCCC4C2)CCC1 |
| Danshen | MOL006219 | O(C(=O)c1ccccc1)C |
| Danshen | MOL006634 | O=C1N2C(C3C4[N+]([O-])(CCC3)CCCC4C2)CCC1 |
| Danshen | MOL009149 | O1c2c3c(CC4[NH+](CCc5cc(O)c(OC)cc45)C3)ccc2OC1 |
| Danshen | MOL011613 | O=C1CC(CC=C1C)C(OC(=O)C)(C)C |
| Danshen | MOL011614 | Oc1cc(ccc1O)C1c2cc(O)c(O)cc2C=C(C(OC(Cc2cc(O)c(O)cc2)C(=O)[O-])=O)C1C(=O)[O-] |
| Danshen | MOL011617 | Oc1cc(ccc1O)C1c2cc(O)c(O)cc2C=C(C(=O)[O-])C1C(=O)[O-] |
| Danshen | MOL011619 | Oc1c2c(C=C(C(OC(Cc3cc(O)c(O)cc3)C(=O)[O-])=O)C(C2C(OC(Cc2cc(O)c(O)cc2)C(=O)[O-])=O)c2cc(O)c(O)cc2)ccc1O |
| Danshen | MOL011623 | Oc1c2c(C=C(C(OC(Cc3cc(O)c(O)cc3)C(OC)=O)=O)C(C2C(OC(Cc2cc(O)c(O)cc2)C(=O)[O-])=O)c2cc(O)c(O)cc2)ccc1O |
| Danshen | MOL011624 | Oc1c2c(C=C(C(=O)[O-])C(C2C(OC(Cc2cc(O)c(O)cc2)C(=O)[O-])=O)c2cc(O)c(O)cc2)ccc1O |
| Danshen | MOL011865 | Oc1cc(ccc1O)CC(OC(=O)\C=C\c1cc(O)c(O)cc1)C(=O)[O-] |
| Danshen | MOL012902 | O(c1cc(ccc1O)CC1[NH+](CCc2c1cc(OC)c(O)c2)C)c1ccc(cc1)CC1[NH+](CCc2c1cc(OC)c(OC)c2)C |
| Danshen | MOL012903 | ClC1CC23[NH2+]CCC2(CC(=O)C(OC)=C3OC)C12C(O)C(OC)=CC2=O |
| Danshen | MOL012904 | ClC1CC23[NH+](CCC2(CC(=O)C(OC)=C3OC)C12C(O)C(OC)=CC2=O)C |
| Danshen | MOL012905 | O(C)C1=CC2C3(CC1=O)c1c(CC2[NH+](CC3)C)c(cc(OC)c1O)-c1cc(OC)c(O)c2c1CC1[NH+](CCC23CC(=O)C(OC)=CC13)C |
| Danshen | MOL012907 | ClC1CC23[NH2+]CCC2(CC(=O)C(OC)=C3OC)C12C(O)C(OC)=CC2=O |
| Danshen | MOL012908 | ClC1CC23[NH+](CCC2(CC(=O)C(OC)=C3OC)C12C(O)C(OC)=CC2=O)C |
| Danshen | MOL012909 | ClC1CC23[NH+](CCC2(CC(=O)C(OC)=C3OC)C12CC(OC)=CC2=O)C |
| Danshen | MOL012913 | O(C)c1cc2CC[NH+](Cc2cc1O)C |
| Danshen | MOL012914 | O(c1cc(ccc1O)CC1[NH+](CCc2c1cc(OC)c(O)c2)C)c1ccc(cc1)CC1[NH+](CCc2c1cc(OC)c(OC)c2)C |
| Danshen | MOL012915 | O(c1cc(ccc1O)CC1[NH+](CCc2c1cc(C)c(OC)c2)C)c1ccc(cc1)CC1[NH+](CCc2c1cc([O-])c(OC)c2)C |
| Danshen | MOL012916 | O(c1cc(ccc1O)CC1[NH+](CCc2c1cc(OC)c(O)c2)C)c1ccc(cc1)CC1[NH+](CCc2c1cc(OC)c(O)c2)C |
| Danshen | MOL012917 | O(c1cc(ccc1O)CC1[NH+](CCc2c1cc(OC)c(OC)c2)C)c1ccc(cc1)CC1[NH+](CCc2c1cc(OC)c(O)c2)C |
| Danshen | MOL012918 | O(C)c1c(O)c2c3c(ccnc3-c3c(cc(OC)cc3)C2=O)c1OC |
| Danshen | MOL012919 | O(c1cc(ccc1O)CC1[NH+](CCc2c1cc(O)c(OC)c2)C)c1ccc(cc1)CC1[NH+](CCc2c1cc(OC)c(OC)c2)C |
| Danshen | MOL012920 | O(C)C1=CC2C3(CC1=O)c1c(CC2[NH+](CC3)C)ccc(OC)c1O |
| Danshen | MOL012921 | O(C)c1c2c3C([NH2+]CCc3cc1OC)CC12C=CC(=O)C=C1 |
| Danshen | MOL012922 | O(C)c1c2c(CC3[NH+](C2)CCc2cc(OC)c(O)cc23)ccc1O |
|  |  |  |

**Table S5.** Detailed information for C-T network.

| **CID** | **Gene Symbols** | **Target type** | **Interactions** |
| --- | --- | --- | --- |
| CID126 | ALDH5A1 | Non-hepatotoxic | Known |
| CID177 | JUN | Hepatotoxic | Known |
| CID177 | TNFSF13B | Hepatotoxic | Known |
| CID177 | HLA-DRB1 | Hepatotoxic | Known |
| CID177 | C3 | Non-hepatotoxic | Known |
| CID177 | CASP1 | Non-hepatotoxic | Known |
| CID177 | CASP3 | Hepatotoxic | Known |
| CID177 | CASP8 | Hepatotoxic | Known |
| CID177 | ESR1 | Hepatotoxic | Known |
| CID177 | FES | Non-hepatotoxic | Known |
| CID177 | FN1 | Hepatotoxic | Known |
| CID177 | FYN | Non-hepatotoxic | Known |
| CID177 | HBB | Hepatotoxic | Known |
| CID177 | LCK | Non-hepatotoxic | Known |
| CID177 | PTPN1 | Non-hepatotoxic | Known |
| CID177 | SOD1 | Hepatotoxic | Known |
| CID177 | HLA-DRA | Hepatotoxic | Known |
| CID177 | DNASE1 | Non-hepatotoxic | Known |
| CID177 | GZMM | Non-hepatotoxic | Known |
| CID177 | SYK | Non-hepatotoxic | Known |
| CID177 | TRAF2 | Non-hepatotoxic | Known |
| CID177 | ABL1 | Hepatotoxic | Known |
| CID177 | AKR1A1 | Non-hepatotoxic | Known |
| CID177 | AKT1 | Hepatotoxic | Known |
| CID177 | ATP5A1 | Non-hepatotoxic | Known |
| CID177 | ATP5C1 | Non-hepatotoxic | Known |
| CID177 | BCL2L1 | Hepatotoxic | Known |
| CID177 | BMP1 | Non-hepatotoxic | Known |
| CID177 | CA2 | Non-hepatotoxic | Known |
| CID177 | CASP6 | Hepatotoxic | Known |
| CID177 | CCNA2 | Non-hepatotoxic | Known |
| CID177 | CDK2 | Non-hepatotoxic | Known |
| CID177 | DDR2 | Non-hepatotoxic | Known |
| CID177 | EIF4E | Non-hepatotoxic | Known |
| CID177 | ESR2 | Hepatotoxic | Known |
| CID177 | F2 | Hepatotoxic | Known |
| CID177 | FCGRT | Non-hepatotoxic | Known |
| CID177 | FOLH1 | Non-hepatotoxic | Known |
| CID177 | GNMT | Hepatotoxic | Known |
| CID177 | GZMB | Non-hepatotoxic | Known |
| CID177 | HPN | Non-hepatotoxic | Known |
| CID177 | MDM2 | Hepatotoxic | Known |
| CID177 | MDM4 | Non-hepatotoxic | Known |
| CID177 | PADI4 | Non-hepatotoxic | Known |
| CID177 | PLK1 | Non-hepatotoxic | Known |
| CID177 | RNASE1 | Non-hepatotoxic | Known |
| CID177 | SERPINB2 | Non-hepatotoxic | Known |
| CID177 | SERPINC1 | Hepatotoxic | Known |
| CID177 | SERPINE1 | Hepatotoxic | Known |
| CID177 | SRC | Non-hepatotoxic | Known |
| CID177 | TGM2 | Non-hepatotoxic | Known |
| CID177 | ACTC1 | Non-hepatotoxic | Known |
| CID177 | AK1 | Non-hepatotoxic | Known |
| CID177 | AKR1B1 | Non-hepatotoxic | Known |
| CID177 | ANKRA2 | Non-hepatotoxic | Known |
| CID177 | ATP2A1 | Non-hepatotoxic | Known |
| CID177 | ATP5B | Non-hepatotoxic | Known |
| CID177 | BACE1 | Non-hepatotoxic | Known |
| CID177 | CA3 | Non-hepatotoxic | Known |
| CID177 | CAPN1 | Non-hepatotoxic | Known |
| CID177 | CAPN9 | Non-hepatotoxic | Known |
| CID177 | CASP2 | Non-hepatotoxic | Known |
| CID177 | CDC14B | Non-hepatotoxic | Known |
| CID177 | CELA1 | Hepatotoxic | Known |
| CID177 | CETN2 | Non-hepatotoxic | Known |
| CID177 | CUL1 | Non-hepatotoxic | Known |
| CID177 | DCUN1D1 | Non-hepatotoxic | Known |
| CID177 | DLG3 | Non-hepatotoxic | Known |
| CID177 | ENAH | Non-hepatotoxic | Known |
| CID177 | F7 | Hepatotoxic | Known |
| CID177 | FABP1 | Non-hepatotoxic | Known |
| CID177 | FNTA | Non-hepatotoxic | Known |
| CID177 | FNTB | Non-hepatotoxic | Known |
| CID177 | GNB1 | Non-hepatotoxic | Known |
| CID177 | GNG2 | Non-hepatotoxic | Known |
| CID177 | GNGT1 | Non-hepatotoxic | Known |
| CID177 | GRAP2 | Hepatotoxic | Known |
| CID177 | GRB2 | Non-hepatotoxic | Known |
| CID177 | GRK6 | Non-hepatotoxic | Known |
| CID177 | GZMH | Non-hepatotoxic | Known |
| CID177 | HBA1; | Non-hepatotoxic | Known |
| CID177 | HBG2 | Non-hepatotoxic | Known |
| CID177 | HBZ | Non-hepatotoxic | Known |
| CID177 | HDAC8 | Non-hepatotoxic | Known |
| CID177 | HGFAC | Non-hepatotoxic | Known |
| CID177 | ITGAM | Non-hepatotoxic | Known |
| CID177 | KLK5 | Non-hepatotoxic | Known |
| CID177 | KNTC1 | Non-hepatotoxic | Known |
| CID177 | LDHA | Non-hepatotoxic | Known |
| CID177 | LRP6 | Non-hepatotoxic | Known |
| CID177 | MASP1 | Non-hepatotoxic | Known |
| CID177 | NCK2 | Non-hepatotoxic | Known |
| CID177 | PARVB | Non-hepatotoxic | Known |
| CID177 | PDC | Non-hepatotoxic | Known |
| CID177 | PFN1 | Non-hepatotoxic | Known |
| CID177 | PLCG1 | Non-hepatotoxic | Known |
| CID177 | PNKP | Non-hepatotoxic | Known |
| CID177 | RBM5 | Non-hepatotoxic | Known |
| CID177 | RHO | Non-hepatotoxic | Known |
| CID177 | S100A10 | Non-hepatotoxic | Known |
| CID177 | S100A11 | Non-hepatotoxic | Known |
| CID177 | S100A3 | Non-hepatotoxic | Known |
| CID177 | SERPINB1 | Non-hepatotoxic | Known |
| CID177 | SERPINH1 | Non-hepatotoxic | Known |
| CID177 | SETD7 | Non-hepatotoxic | Known |
| CID177 | SIRT5 | Non-hepatotoxic | Known |
| CID177 | STIP1 | Non-hepatotoxic | Known |
| CID177 | TLL1 | Non-hepatotoxic | Known |
| CID177 | TPSAB1 | Non-hepatotoxic | Known |
| CID177 | TSG101 | Non-hepatotoxic | Known |
| CID177 | WDR5 | Non-hepatotoxic | Known |
| CID190 | ADA | Hepatotoxic | Known |
| CID190 | ACACB | Hepatotoxic | Known |
| CID190 | ACP1 | Hepatotoxic | Known |
| CID190 | ACVR2B | Non-hepatotoxic | Known |
| CID190 | APRT | Non-hepatotoxic | Known |
| CID190 | HSP90AA1 | Non-hepatotoxic | Known |
| CID190 | MTAP | Non-hepatotoxic | Known |
| CID190 | PNMT | Non-hepatotoxic | Known |
| CID190 | PRKCI | Non-hepatotoxic | Known |
| CID190 | XDH | Hepatotoxic | Known |
| CID190 | PECR | Non-hepatotoxic | Known |
| CID190 | PYGM | Hepatotoxic | Known |
| CID190 | SF3B1 | Non-hepatotoxic | Known |
| CID190 | SF3B6 | Non-hepatotoxic | Known |
| CID190 | SRPK2 | Non-hepatotoxic | Known |
| CID190 | STK24 | Non-hepatotoxic | Known |
| CID190 | AHCYL1 | Non-hepatotoxic | Known |
| CID190 | LMNA | Non-hepatotoxic | Known |
| CID190 | TSHR | Non-hepatotoxic | Predicted |
| CID190 | BLM | Non-hepatotoxic | Predicted |
| CID190 | HTT | Non-hepatotoxic | Predicted |
| CID190 | ALDH1A1 | Non-hepatotoxic | Predicted |
| CID190 | HSD17B10 | Non-hepatotoxic | Predicted |
| CID190 | ACHE | Non-hepatotoxic | Predicted |
| CID190 | MAPT | Non-hepatotoxic | Predicted |
| CID190 | HIF1A | Hepatotoxic | Predicted |
| CID190 | RAB9A | Non-hepatotoxic | Predicted |
| CID190 | CYP3A4 | Hepatotoxic | Predicted |
| CID190 | CCNE1 | Non-hepatotoxic | Predicted |
| CID190 | SMN1 | Non-hepatotoxic | Predicted |
| CID190 | TP53 | Hepatotoxic | Predicted |
| CID190 | GDA | Non-hepatotoxic | Predicted |
| CID190 | NPC1 | Non-hepatotoxic | Predicted |
| CID190 | CYP2D6 | Hepatotoxic | Predicted |
| CID190 | CYP2C19 | Hepatotoxic | Predicted |
| CID190 | RECQL | Non-hepatotoxic | Predicted |
| CID190 | TRPA1 | Non-hepatotoxic | Predicted |
| CID190 | PMP22 | Non-hepatotoxic | Predicted |
| CID191 | HIF1A | Hepatotoxic | Known |
| CID191 | LMNA | Non-hepatotoxic | Known |
| CID191 | HTT | Non-hepatotoxic | Known |
| CID191 | HSD17B10 | Non-hepatotoxic | Predicted |
| CID191 | TSHR | Non-hepatotoxic | Predicted |
| CID191 | CYP2D6 | Hepatotoxic | Predicted |
| CID191 | ALDH1A1 | Non-hepatotoxic | Predicted |
| CID191 | THPO | Non-hepatotoxic | Predicted |
| CID191 | TP53 | Hepatotoxic | Predicted |
| CID191 | CYP2C19 | Hepatotoxic | Predicted |
| CID191 | ALOX15 | Non-hepatotoxic | Predicted |
| CID191 | SMN1 | Non-hepatotoxic | Predicted |
| CID191 | MAPT | Non-hepatotoxic | Predicted |
| CID191 | CYP3A4 | Hepatotoxic | Predicted |
| CID191 | NFKB1 | Hepatotoxic | Predicted |
| CID191 | TDP1 | Non-hepatotoxic | Predicted |
| CID191 | RAB9A | Non-hepatotoxic | Predicted |
| CID191 | JAK2 | Hepatotoxic | Predicted |
| CID191 | KMT2A | Non-hepatotoxic | Predicted |
| CID191 | BLM | Non-hepatotoxic | Predicted |
| CID191 | KCNN3 | Non-hepatotoxic | Predicted |
| CID191 | NPC1 | Non-hepatotoxic | Predicted |
| CID191 | PKM | Non-hepatotoxic | Predicted |
| CID240 | CYP2A6 | Hepatotoxic | Known |
| CID240 | ALDH1A1 | Non-hepatotoxic | Known |
| CID240 | CYP2A13 | Hepatotoxic | Predicted |
| CID240 | TRPA1 | Non-hepatotoxic | Predicted |
| CID240 | TAAR1 | Non-hepatotoxic | Predicted |
| CID240 | HPGD | Non-hepatotoxic | Predicted |
| CID240 | MAPT | Non-hepatotoxic | Predicted |
| CID240 | CYP1A2 | Hepatotoxic | Predicted |
| CID240 | HSD17B10 | Non-hepatotoxic | Predicted |
| CID240 | ALOX15 | Non-hepatotoxic | Predicted |
| CID240 | TDP1 | Non-hepatotoxic | Predicted |
| CID240 | TSHR | Non-hepatotoxic | Predicted |
| CID240 | CYP2E1 | Hepatotoxic | Predicted |
| CID240 | LMNA | Non-hepatotoxic | Predicted |
| CID240 | CYP3A4 | Hepatotoxic | Predicted |
| CID240 | MIF | Hepatotoxic | Predicted |
| CID240 | CYP2D6 | Hepatotoxic | Predicted |
| CID240 | CYP2C19 | Hepatotoxic | Predicted |
| CID240 | IMPDH2 | Non-hepatotoxic | Predicted |
| CID240 | KDM4E | Non-hepatotoxic | Predicted |
| CID240 | MAPK1 | Non-hepatotoxic | Predicted |
| CID240 | CYP2C9 | Hepatotoxic | Predicted |
| CID242 | AXIN1 | Non-hepatotoxic | Known |
| CID242 | BCHE | Non-hepatotoxic | Known |
| CID242 | CES1 | Hepatotoxic | Known |
| CID242 | DAO | Non-hepatotoxic | Known |
| CID242 | ACSM1 | Non-hepatotoxic | Known |
| CID242 | ACSM2B | Non-hepatotoxic | Known |
| CID242 | HRSP12 | Non-hepatotoxic | Known |
| CID242 | NDRG2 | Non-hepatotoxic | Known |
| CID242 | PAFAH1B1 | Non-hepatotoxic | Known |
| CID242 | PRDX5 | Non-hepatotoxic | Known |
| CID242 | SDCBP | Non-hepatotoxic | Known |
| CID242 | YWHAZ | Non-hepatotoxic | Known |
| CID242 | TYR | Non-hepatotoxic | Predicted |
| CID242 | SRD5A2 | Hepatotoxic | Predicted |
| CID242 | TSHR | Non-hepatotoxic | Predicted |
| CID242 | MAPT | Non-hepatotoxic | Predicted |
| CID242 | LMNA | Non-hepatotoxic | Predicted |
| CID242 | CA1 | Non-hepatotoxic | Predicted |
| CID242 | CA2 | Non-hepatotoxic | Known |
| CID242 | ALDH1A1 | Non-hepatotoxic | Predicted |
| CID242 | CYP3A4 | Hepatotoxic | Predicted |
| CID242 | RAB9A | Non-hepatotoxic | Known |
| CID242 | ABCB1 | Hepatotoxic | Predicted |
| CID242 | CA12 | Non-hepatotoxic | Predicted |
| CID242 | CA9 | Non-hepatotoxic | Predicted |
| CID242 | ABCG2 | Hepatotoxic | Predicted |
| CID242 | CYP19A1 | Hepatotoxic | Predicted |
| CID242 | TDP1 | Non-hepatotoxic | Predicted |
| CID242 | TP53 | Hepatotoxic | Predicted |
| CID242 | NPC1 | Non-hepatotoxic | Predicted |
| CID242 | FABP3 | Non-hepatotoxic | Predicted |
| CID242 | KMT2A | Non-hepatotoxic | Predicted |
| CID244 | TSHR | Non-hepatotoxic | Known |
| CID244 | LMNA | Non-hepatotoxic | Predicted |
| CID244 | TP53 | Hepatotoxic | Predicted |
| CID244 | LTA4H | Non-hepatotoxic | Predicted |
| CID244 | TBXAS1 | Non-hepatotoxic | Predicted |
| CID244 | DAO | Non-hepatotoxic | Predicted |
| CID244 | NFKB1 | Hepatotoxic | Predicted |
| CID244 | THPO | Non-hepatotoxic | Predicted |
| CID244 | CYP2C19 | Hepatotoxic | Predicted |
| CID244 | TDP1 | Non-hepatotoxic | Predicted |
| CID244 | CYP3A4 | Hepatotoxic | Predicted |
| CID244 | HPGD | Non-hepatotoxic | Predicted |
| CID244 | HSD17B10 | Non-hepatotoxic | Predicted |
| CID244 | HIF1A | Hepatotoxic | Predicted |
| CID244 | ALOX15 | Non-hepatotoxic | Predicted |
| CID244 | MAPK1 | Non-hepatotoxic | Predicted |
| CID244 | CYP2D6 | Hepatotoxic | Predicted |
| CID244 | NPSR1 | Non-hepatotoxic | Predicted |
| CID244 | ALDH1A1 | Non-hepatotoxic | Predicted |
| CID244 | CYP1A2 | Hepatotoxic | Predicted |
| CID244 | TAAR1 | Non-hepatotoxic | Predicted |
| CID305 | ACHE | Non-hepatotoxic | Known |
| CID305 | BCHE | Non-hepatotoxic | Known |
| CID305 | SLC5A7 | Non-hepatotoxic | Known |
| CID305 | CHAT | Non-hepatotoxic | Known |
| CID305 | CHKA | Non-hepatotoxic | Known |
| CID305 | CHKB | Non-hepatotoxic | Known |
| CID305 | ETNK1 | Non-hepatotoxic | Known |
| CID305 | LYZ | Non-hepatotoxic | Known |
| CID342 | ACHE | Non-hepatotoxic | Known |
| CID342 | INS-IGF2 | Non-hepatotoxic | Known |
| CID342 | BCHE | Non-hepatotoxic | Predicted |
| CID342 | GDA | Non-hepatotoxic | Predicted |
| CID342 | LMNA | Non-hepatotoxic | Predicted |
| CID342 | ADORA2A | Non-hepatotoxic | Predicted |
| CID342 | TOP1 | Non-hepatotoxic | Predicted |
| CID342 | KCNH2 | Non-hepatotoxic | Predicted |
| CID342 | CYP2A6 | Hepatotoxic | Predicted |
| CID342 | SENP8 | Non-hepatotoxic | Predicted |
| CID342 | CYP3A4 | Hepatotoxic | Predicted |
| CID342 | CYP2A13 | Hepatotoxic | Predicted |
| CID342 | CYP2D6 | Hepatotoxic | Predicted |
| CID342 | CYP2C19 | Hepatotoxic | Predicted |
| CID342 | CASP1 | Non-hepatotoxic | Predicted |
| CID342 | NMUR2 | Non-hepatotoxic | Predicted |
| CID342 | BACE1 | Non-hepatotoxic | Predicted |
| CID342 | BLM | Non-hepatotoxic | Predicted |
| CID342 | CYP2C9 | Hepatotoxic | Predicted |
| CID342 | HSD17B10 | Non-hepatotoxic | Predicted |
| CID342 | PRKCE | Hepatotoxic | Predicted |
| CID342 | CES1 | Hepatotoxic | Predicted |
| CID453 | LMNA | Non-hepatotoxic | Known |
| CID785 | RELA | Hepatotoxic | Known |
| CID785 | TNF | Hepatotoxic | Known |
| CID785 | CA12 | Non-hepatotoxic | Known |
| CID785 | NFKB1 | Hepatotoxic | Known |
| CID785 | BLM | Non-hepatotoxic | Known |
| CID785 | KMT2A | Non-hepatotoxic | Known |
| CID785 | HIF1A | Hepatotoxic | Known |
| CID785 | CA2 | Non-hepatotoxic | Known |
| CID785 | CA3 | Non-hepatotoxic | Known |
| CID785 | LMNA | Non-hepatotoxic | Known |
| CID785 | CYP3A4 | Hepatotoxic | Known |
| CID785 | MAPT | Non-hepatotoxic | Known |
| CID785 | HPGD | Non-hepatotoxic | Known |
| CID785 | HSD17B10 | Non-hepatotoxic | Known |
| CID785 | CA9 | Non-hepatotoxic | Predicted |
| CID785 | CA1 | Non-hepatotoxic | Predicted |
| CID785 | CA7 | Non-hepatotoxic | Predicted |
| CID785 | CA14 | Non-hepatotoxic | Predicted |
| CID785 | CA5A | Non-hepatotoxic | Predicted |
| CID785 | CA5B | Non-hepatotoxic | Predicted |
| CID785 | CA4 | Non-hepatotoxic | Predicted |
| CID785 | CA6 | Non-hepatotoxic | Predicted |
| CID785 | ALDH1A1 | Non-hepatotoxic | Predicted |
| CID785 | ALOX15 | Non-hepatotoxic | Predicted |
| CID785 | TP53 | Hepatotoxic | Predicted |
| CID785 | KDM4E | Non-hepatotoxic | Predicted |
| CID785 | TDP1 | Non-hepatotoxic | Predicted |
| CID785 | TSHR | Non-hepatotoxic | Predicted |
| CID785 | CYP2C19 | Hepatotoxic | Predicted |
| CID785 | THPO | Non-hepatotoxic | Predicted |
| CID785 | CA13 | Non-hepatotoxic | Predicted |
| CID785 | RECQL | Non-hepatotoxic | Predicted |
| CID785 | MAPK1 | Non-hepatotoxic | Predicted |
| CID785 | CYP2D6 | Hepatotoxic | Predicted |
| CID854 | PTGS2 | Hepatotoxic | Known |
| CID854 | NOS2 | Hepatotoxic | Known |
| CID854 | MAPK1 | Non-hepatotoxic | Known |
| CID931 | JUN | Hepatotoxic | Known |
| CID931 | BCL2 | Non-hepatotoxic | Known |
| CID931 | MPO | Hepatotoxic | Known |
| CID931 | FUS | Non-hepatotoxic | Known |
| CID931 | FOS | Non-hepatotoxic | Known |
| CID931 | RAF1 | Hepatotoxic | Known |
| CID931 | ADRA2A | Hepatotoxic | Known |
| CID931 | NPY1R | Non-hepatotoxic | Known |
| CID931 | NPY2R | Non-hepatotoxic | Known |
| CID931 | NPY5R | Hepatotoxic | Known |
| CID931 | CYP1A1 | Hepatotoxic | Known |
| CID931 | UCHL1 | Non-hepatotoxic | Known |
| CID931 | EIF6 | Non-hepatotoxic | Known |
| CID931 | PRDX5 | Non-hepatotoxic | Known |
| CID931 | CXCL8 | Non-hepatotoxic | Known |
| CID931 | HTR7 | Non-hepatotoxic | Predicted |
| CID931 | ADRA2B | Non-hepatotoxic | Predicted |
| CID931 | SLC6A2 | Non-hepatotoxic | Predicted |
| CID931 | SLC6A3 | Non-hepatotoxic | Predicted |
| CID931 | NISCH | Non-hepatotoxic | Predicted |
| CID931 | ADRA1A | Hepatotoxic | Predicted |
| CID931 | ADRA2C | Non-hepatotoxic | Known |
| CID931 | TAAR1 | Non-hepatotoxic | Predicted |
| CID931 | ADRA1B | Non-hepatotoxic | Predicted |
| CID931 | ADRA1C | Non-hepatotoxic | Predicted |
| CID931 | HRH3 | Non-hepatotoxic | Predicted |
| CID931 | ADRA1D | Non-hepatotoxic | Predicted |
| CID931 | SLC6A4 | Hepatotoxic | Predicted |
| CID931 | HTR1D | Non-hepatotoxic | Predicted |
| CID931 | ADRB3 | Hepatotoxic | Predicted |
| CID931 | ADRB1 | Non-hepatotoxic | Predicted |
| CID931 | HTR1A | Hepatotoxic | Predicted |
| CID931 | HTR1B | Non-hepatotoxic | Predicted |
| CID931 | LMNA | Non-hepatotoxic | Predicted |
| CID931 | CYP2D6 | Hepatotoxic | Predicted |
| CID932 | RELA | Hepatotoxic | Known |
| CID932 | BCL2 | Non-hepatotoxic | Known |
| CID932 | PPARG | Hepatotoxic | Known |
| CID932 | ADIPOQ | Hepatotoxic | Known |
| CID932 | ESR1 | Hepatotoxic | Known |
| CID932 | SOD1 | Hepatotoxic | Known |
| CID932 | LDLR | Hepatotoxic | Known |
| CID932 | CASP3 | Hepatotoxic | Known |
| CID932 | AKT1 | Hepatotoxic | Known |
| CID932 | BAD | Non-hepatotoxic | Known |
| CID932 | PPARA | Hepatotoxic | Known |
| CID932 | HMGCR | Hepatotoxic | Known |
| CID932 | ESR2 | Hepatotoxic | Known |
| CID932 | NOS2 | Hepatotoxic | Known |
| CID932 | AKR1C1 | Hepatotoxic | Known |
| CID932 | UGT1A1 | Hepatotoxic | Known |
| CID932 | CAT | Hepatotoxic | Known |
| CID932 | APOB | Hepatotoxic | Known |
| CID932 | MAPK3 | Non-hepatotoxic | Known |
| CID932 | SREBF1 | Hepatotoxic | Known |
| CID932 | GSR | Non-hepatotoxic | Known |
| CID932 | GSTP1 | Hepatotoxic | Known |
| CID932 | CES1 | Hepatotoxic | Known |
| CID932 | CYP19A1 | Hepatotoxic | Known |
| CID932 | MTTP | Hepatotoxic | Known |
| CID932 | SOAT2 | Non-hepatotoxic | Known |
| CID932 | PLB1 | Non-hepatotoxic | Known |
| CID932 | GOT1 | Hepatotoxic | Known |
| CID932 | ABAT | Non-hepatotoxic | Known |
| CID932 | SOAT1 | Non-hepatotoxic | Known |
| CID932 | ABCC1 | Hepatotoxic | Known |
| CID932 | CYP3A4 | Hepatotoxic | Known |
| CID932 | CYP2C19 | Hepatotoxic | Predicted |
| CID932 | MAPT | Non-hepatotoxic | Predicted |
| CID932 | HPGD | Non-hepatotoxic | Predicted |
| CID932 | MAOB | Hepatotoxic | Predicted |
| CID932 | CYP2C9 | Hepatotoxic | Predicted |
| CID932 | MAPK1 | Non-hepatotoxic | Known |
| CID932 | TP53 | Hepatotoxic | Predicted |
| CID932 | CYP1A2 | Hepatotoxic | Predicted |
| CID932 | ALOX12 | Non-hepatotoxic | Predicted |
| CID932 | CYP2D6 | Hepatotoxic | Predicted |
| CID932 | AR | Hepatotoxic | Predicted |
| CID932 | HSD17B10 | Non-hepatotoxic | Predicted |
| CID932 | ALDH1A1 | Non-hepatotoxic | Predicted |
| CID932 | SMN1 | Non-hepatotoxic | Predicted |
| CID932 | CYP1A1 | Hepatotoxic | Predicted |
| CID932 | CYP1B1 | Hepatotoxic | Predicted |
| CID932 | FASN | Non-hepatotoxic | Known |
| CID932 | KDM4E | Non-hepatotoxic | Predicted |
| CID932 | ALOX15 | Non-hepatotoxic | Predicted |
| CID932 | CYP11B2 | Hepatotoxic | Predicted |
| CID957 | PRKCB | Hepatotoxic | Known |
| CID995 | TSHR | Non-hepatotoxic | Known |
| CID995 | TDP1 | Non-hepatotoxic | Known |
| CID995 | LMNA | Non-hepatotoxic | Predicted |
| CID995 | HPGD | Non-hepatotoxic | Predicted |
| CID995 | RECQL | Non-hepatotoxic | Predicted |
| CID995 | TP53 | Hepatotoxic | Predicted |
| CID995 | HSD17B10 | Non-hepatotoxic | Predicted |
| CID995 | ALDH1A1 | Non-hepatotoxic | Predicted |
| CID995 | APEX1 | Non-hepatotoxic | Predicted |
| CID995 | ALOX15 | Non-hepatotoxic | Predicted |
| CID995 | MAPT | Non-hepatotoxic | Predicted |
| CID995 | KDM4E | Non-hepatotoxic | Predicted |
| CID995 | L3MBTL1 | Non-hepatotoxic | Predicted |
| CID995 | THPO | Non-hepatotoxic | Predicted |
| CID995 | NFKB1 | Hepatotoxic | Predicted |
| CID995 | HPRT1 | Non-hepatotoxic | Predicted |
| CID995 | CYP2C19 | Hepatotoxic | Predicted |
| CID995 | HIF1A | Hepatotoxic | Predicted |
| CID995 | LTA4H | Non-hepatotoxic | Predicted |
| CID995 | KIF11 | Non-hepatotoxic | Predicted |
| CID995 | CYP1A2 | Hepatotoxic | Predicted |
| CID995 | CYP3A4 | Hepatotoxic | Predicted |
| CID998 | AOC3 | Non-hepatotoxic | Known |
| CID998 | AOC2 | Non-hepatotoxic | Known |
| CID1115 | PPARG | Hepatotoxic | Known |
| CID1115 | SREBF2 | Non-hepatotoxic | Known |
| CID1115 | FOS | Non-hepatotoxic | Known |
| CID1115 | NR1I3 | Hepatotoxic | Known |
| CID1115 | ALAD | Non-hepatotoxic | Known |
| CID1115 | SREBF1 | Hepatotoxic | Known |
| CID1115 | COL1A1 | Hepatotoxic | Known |
| CID1115 | ACSL4 | Hepatotoxic | Known |
| CID1115 | LCT | Hepatotoxic | Known |
| CID1115 | AMY1A | Non-hepatotoxic | Known |
| CID1115 | FOSL2 | Non-hepatotoxic | Known |
| CID1115 | CHI3L1 | Non-hepatotoxic | Known |
| CID1115 | CYP1A2 | Hepatotoxic | Known |
| CID1115 | HK1 | Hepatotoxic | Known |
| CID1115 | AMY2A | Non-hepatotoxic | Known |
| CID1115 | GLUL | Non-hepatotoxic | Known |
| CID1115 | G6PC | Non-hepatotoxic | Known |
| CID1115 | AKR1B1 | Non-hepatotoxic | Known |
| CID1115 | COL7A1 | Non-hepatotoxic | Known |
| CID1115 | ACSL1 | Non-hepatotoxic | Known |
| CID1115 | SI | Non-hepatotoxic | Known |
| CID1130 | TPK1 | Non-hepatotoxic | Known |
| CID1183 | JUN | Hepatotoxic | Known |
| CID1183 | MMP9 | Hepatotoxic | Known |
| CID1183 | MAPK1 | Non-hepatotoxic | Known |
| CID1183 | TDP1 | Non-hepatotoxic | Known |
| CID1183 | RECQL | Non-hepatotoxic | Predicted |
| CID1183 | APEX1 | Non-hepatotoxic | Predicted |
| CID1183 | ALDH1A1 | Non-hepatotoxic | Predicted |
| CID1183 | HPGD | Non-hepatotoxic | Predicted |
| CID1183 | HSD17B10 | Non-hepatotoxic | Predicted |
| CID1183 | L3MBTL1 | Non-hepatotoxic | Predicted |
| CID1183 | KDM4E | Non-hepatotoxic | Predicted |
| CID1183 | ALOX15 | Non-hepatotoxic | Predicted |
| CID1183 | MAPT | Non-hepatotoxic | Predicted |
| CID1183 | TSHR | Non-hepatotoxic | Predicted |
| CID1183 | BLM | Non-hepatotoxic | Predicted |
| CID1183 | POLB | Non-hepatotoxic | Predicted |
| CID1183 | ABCC1 | Hepatotoxic | Predicted |
| CID1183 | BRCA1 | Non-hepatotoxic | Predicted |
| CID1183 | LMNA | Non-hepatotoxic | Predicted |
| CID1183 | TP53 | Hepatotoxic | Predicted |
| CID1183 | HIF1A | Hepatotoxic | Predicted |
| CID1183 | MCL1 | Hepatotoxic | Predicted |
| CID1183 | USP2 | Non-hepatotoxic | Predicted |
| CID1183 | AHR | Hepatotoxic | Predicted |
| CID2355 | TSHR | Non-hepatotoxic | Known |
| CID2355 | CYP3A4 | Hepatotoxic | Known |
| CID2355 | CYP2D6 | Hepatotoxic | Known |
| CID2355 | CYP2C9 | Hepatotoxic | Known |
| CID2355 | CYP2C19 | Hepatotoxic | Known |
| CID2355 | MAPT | Non-hepatotoxic | Predicted |
| CID2355 | LMNA | Non-hepatotoxic | Predicted |
| CID2355 | TP53 | Hepatotoxic | Predicted |
| CID2355 | HIF1A | Hepatotoxic | Predicted |
| CID2355 | HSD17B10 | Non-hepatotoxic | Predicted |
| CID2355 | NFKB1 | Hepatotoxic | Predicted |
| CID2355 | ALDH1A1 | Non-hepatotoxic | Predicted |
| CID2355 | HPGD | Non-hepatotoxic | Predicted |
| CID2355 | THPO | Non-hepatotoxic | Predicted |
| CID2355 | MAPK1 | Non-hepatotoxic | Predicted |
| CID2355 | SMN1 | Non-hepatotoxic | Predicted |
| CID2355 | ALOX15 | Non-hepatotoxic | Predicted |
| CID2355 | PARP1 | Hepatotoxic | Predicted |
| CID2355 | KDM4E | Non-hepatotoxic | Predicted |
| CID2355 | TDP1 | Non-hepatotoxic | Predicted |
| CID2355 | CYP2A6 | Hepatotoxic | Predicted |
| CID2355 | CYP2B6 | Hepatotoxic | Predicted |
| CID2355 | ACHE | Non-hepatotoxic | Predicted |
| CID2355 | CYP1A1 | Hepatotoxic | Predicted |
| CID2355 | THRB | Non-hepatotoxic | Predicted |
| CID2879 | ACHE | Non-hepatotoxic | Known |
| CID2879 | BCHE | Non-hepatotoxic | Predicted |
| CID2879 | GDA | Non-hepatotoxic | Predicted |
| CID2879 | LMNA | Non-hepatotoxic | Predicted |
| CID2879 | ADORA2A | Non-hepatotoxic | Predicted |
| CID2879 | TOP1 | Non-hepatotoxic | Predicted |
| CID2879 | CYP2A6 | Hepatotoxic | Predicted |
| CID2879 | KCNH2 | Non-hepatotoxic | Predicted |
| CID2879 | SENP8 | Non-hepatotoxic | Predicted |
| CID2879 | CYP2A13 | Hepatotoxic | Predicted |
| CID2879 | CYP2C19 | Hepatotoxic | Predicted |
| CID2879 | CYP2D6 | Hepatotoxic | Predicted |
| CID2879 | CYP3A4 | Hepatotoxic | Predicted |
| CID2879 | CASP1 | Non-hepatotoxic | Predicted |
| CID2879 | NMUR2 | Non-hepatotoxic | Predicted |
| CID2879 | BACE1 | Non-hepatotoxic | Predicted |
| CID2879 | CYP2C9 | Hepatotoxic | Predicted |
| CID2879 | BLM | Non-hepatotoxic | Predicted |
| CID2879 | HSD17B10 | Non-hepatotoxic | Predicted |
| CID2879 | PRKCE | Hepatotoxic | Predicted |
| CID2879 | CES1 | Hepatotoxic | Predicted |
| CID3220 | ESR1 | Hepatotoxic | Known |
| CID3220 | PRKCE | Hepatotoxic | Known |
| CID3220 | CASP3 | Hepatotoxic | Known |
| CID3220 | MMP9 | Hepatotoxic | Known |
| CID3220 | PPARG | Hepatotoxic | Known |
| CID3220 | FLT1 | Hepatotoxic | Known |
| CID3220 | KDR | Hepatotoxic | Known |
| CID3220 | IL1B | Hepatotoxic | Known |
| CID3220 | TNF | Hepatotoxic | Known |
| CID3220 | MYC | Hepatotoxic | Known |
| CID3220 | TGFB1 | Hepatotoxic | Known |
| CID3220 | BTK | Non-hepatotoxic | Known |
| CID3220 | PRKCD | Non-hepatotoxic | Known |
| CID3220 | TSHR | Non-hepatotoxic | Known |
| CID3220 | BLM | Non-hepatotoxic | Known |
| CID3220 | HIF1A | Hepatotoxic | Known |
| CID3220 | CSNK2A1 | Non-hepatotoxic | Known |
| CID3220 | ESR2 | Hepatotoxic | Known |
| CID3220 | ACTA2 | Non-hepatotoxic | Known |
| CID3220 | CSF2 | Hepatotoxic | Known |
| CID3220 | FLT4 | Non-hepatotoxic | Known |
| CID3220 | SLC2A1 | Hepatotoxic | Known |
| CID3220 | SLC2A4 | Hepatotoxic | Known |
| CID3220 | MMP1 | Hepatotoxic | Known |
| CID3220 | NOS2 | Hepatotoxic | Known |
| CID3220 | CDKN1A | Non-hepatotoxic | Known |
| CID3220 | EGF | Hepatotoxic | Known |
| CID3220 | PTP4A3 | Non-hepatotoxic | Known |
| CID3220 | MAOB | Hepatotoxic | Known |
| CID3220 | CYP1A1 | Hepatotoxic | Known |
| CID3220 | LMNA | Non-hepatotoxic | Known |
| CID3220 | CYP3A4 | Hepatotoxic | Known |
| CID3220 | MAPT | Non-hepatotoxic | Known |
| CID3220 | CYP2C9 | Hepatotoxic | Known |
| CID3220 | ALOX15 | Non-hepatotoxic | Known |
| CID3220 | CYP2C19 | Hepatotoxic | Known |
| CID3220 | HSD17B10 | Non-hepatotoxic | Known |
| CID3220 | CSNK2B | Non-hepatotoxic | Known |
| CID3220 | TP53 | Hepatotoxic | Known |
| CID3220 | HPGD | Non-hepatotoxic | Predicted |
| CID3220 | ALDH1A1 | Non-hepatotoxic | Predicted |
| CID3220 | CYP2D6 | Hepatotoxic | Predicted |
| CID3220 | KDM4E | Non-hepatotoxic | Predicted |
| CID3220 | THPO | Non-hepatotoxic | Predicted |
| CID3220 | TDP1 | Non-hepatotoxic | Predicted |
| CID3220 | NFKB1 | Hepatotoxic | Predicted |
| CID3220 | KMT2A | Non-hepatotoxic | Predicted |
| CID3220 | RECQL | Non-hepatotoxic | Predicted |
| CID3220 | MAPK1 | Non-hepatotoxic | Predicted |
| CID3220 | USP2 | Non-hepatotoxic | Predicted |
| CID3220 | PKM | Non-hepatotoxic | Predicted |
| CID3220 | ALOX12 | Non-hepatotoxic | Predicted |
| CID3220 | UGT1A1 | Hepatotoxic | Predicted |
| CID3220 | SMN1 | Non-hepatotoxic | Predicted |
| CID3220 | CFTR | Hepatotoxic | Predicted |
| CID3220 | APEX1 | Non-hepatotoxic | Predicted |
| CID3220 | NPC1 | Non-hepatotoxic | Predicted |
| CID3220 | CCNB3 | Non-hepatotoxic | Predicted |
| CID3314 | ALOX5 | Non-hepatotoxic | Known |
| CID3314 | RELA | Hepatotoxic | Known |
| CID3314 | PTGS2 | Hepatotoxic | Known |
| CID3314 | AHR | Hepatotoxic | Known |
| CID3314 | CD86 | Hepatotoxic | Known |
| CID3314 | MAOA | Non-hepatotoxic | Known |
| CID3314 | NOS2 | Hepatotoxic | Known |
| CID3314 | PPP3CA | Non-hepatotoxic | Known |
| CID3314 | CYP1B1 | Hepatotoxic | Known |
| CID3314 | TRPV3 | Non-hepatotoxic | Known |
| CID3314 | MAOB | Hepatotoxic | Known |
| CID3314 | MUC1 | Non-hepatotoxic | Known |
| CID3314 | CRYZ | Non-hepatotoxic | Known |
| CID3314 | ATP2C1 | Non-hepatotoxic | Known |
| CID3314 | ABCC2 | Hepatotoxic | Known |
| CID3314 | TRPC3 | Non-hepatotoxic | Known |
| CID3314 | ENOX2 | Non-hepatotoxic | Known |
| CID3314 | CYP3A4 | Hepatotoxic | Known |
| CID3314 | MAPT | Non-hepatotoxic | Predicted |
| CID3314 | CYP2C19 | Hepatotoxic | Predicted |
| CID3314 | HPGD | Non-hepatotoxic | Predicted |
| CID3314 | MAPK1 | Non-hepatotoxic | Predicted |
| CID3314 | CYP2C9 | Hepatotoxic | Predicted |
| CID3314 | CYP2D6 | Hepatotoxic | Predicted |
| CID3314 | SMN1 | Non-hepatotoxic | Predicted |
| CID3314 | HSD17B10 | Non-hepatotoxic | Predicted |
| CID3314 | TP53 | Hepatotoxic | Predicted |
| CID3314 | ALDH1A1 | Non-hepatotoxic | Predicted |
| CID3314 | LMNA | Non-hepatotoxic | Predicted |
| CID3314 | KDM4E | Non-hepatotoxic | Predicted |
| CID3314 | ALOX15 | Non-hepatotoxic | Predicted |
| CID3314 | TSHR | Non-hepatotoxic | Predicted |
| CID3314 | NFKB1 | Hepatotoxic | Predicted |
| CID3314 | THPO | Non-hepatotoxic | Predicted |
| CID3314 | CYP1A1 | Hepatotoxic | Known |
| CID3314 | AR | Hepatotoxic | Predicted |
| CID3314 | CYP1A2 | Hepatotoxic | Predicted |
| CID3314 | HIF1A | Hepatotoxic | Predicted |
| CID3593 | MAPT | Non-hepatotoxic | Known |
| CID3593 | CYP3A4 | Hepatotoxic | Predicted |
| CID3593 | TP53 | Hepatotoxic | Predicted |
| CID3593 | KMT2A | Non-hepatotoxic | Predicted |
| CID3593 | ALDH1A1 | Non-hepatotoxic | Predicted |
| CID3593 | KDM4E | Non-hepatotoxic | Predicted |
| CID3593 | ALOX15 | Non-hepatotoxic | Predicted |
| CID3593 | RAB9A | Non-hepatotoxic | Predicted |
| CID3593 | RECQL | Non-hepatotoxic | Predicted |
| CID3593 | MAPK1 | Non-hepatotoxic | Predicted |
| CID3593 | HPGD | Non-hepatotoxic | Predicted |
| CID3593 | LMNA | Non-hepatotoxic | Predicted |
| CID3593 | NPC1 | Non-hepatotoxic | Predicted |
| CID3593 | SMN1 | Non-hepatotoxic | Predicted |
| CID3593 | USP2 | Non-hepatotoxic | Predicted |
| CID3593 | CYP2C9 | Hepatotoxic | Predicted |
| CID3593 | TDP1 | Non-hepatotoxic | Predicted |
| CID3593 | CYP2C19 | Hepatotoxic | Predicted |
| CID3593 | GAA | Non-hepatotoxic | Predicted |
| CID3593 | BLM | Non-hepatotoxic | Predicted |
| CID3593 | THRB | Non-hepatotoxic | Predicted |
| CID4770 | LMNA | Non-hepatotoxic | Known |
| CID4770 | TSHR | Non-hepatotoxic | Predicted |
| CID4770 | BLM | Non-hepatotoxic | Predicted |
| CID4770 | HTT | Non-hepatotoxic | Predicted |
| CID4770 | MAPT | Non-hepatotoxic | Predicted |
| CID4770 | ALDH1A1 | Non-hepatotoxic | Predicted |
| CID4770 | HSD17B10 | Non-hepatotoxic | Predicted |
| CID4770 | ACHE | Non-hepatotoxic | Predicted |
| CID4770 | RAB9A | Non-hepatotoxic | Predicted |
| CID4770 | CYP3A4 | Hepatotoxic | Predicted |
| CID4770 | HIF1A | Hepatotoxic | Predicted |
| CID4770 | TP53 | Hepatotoxic | Predicted |
| CID4770 | SMN1 | Non-hepatotoxic | Predicted |
| CID4770 | NPC1 | Non-hepatotoxic | Predicted |
| CID4770 | CYP2D6 | Hepatotoxic | Predicted |
| CID4770 | CYP2C19 | Hepatotoxic | Predicted |
| CID4770 | TRPA1 | Non-hepatotoxic | Predicted |
| CID4770 | PMP22 | Non-hepatotoxic | Predicted |
| CID4770 | ALOX15 | Non-hepatotoxic | Predicted |
| CID4770 | GSG2 | Non-hepatotoxic | Predicted |
| CID4770 | TDP1 | Non-hepatotoxic | Predicted |
| CID4788 | KMT2A | Non-hepatotoxic | Known |
| CID4788 | ESR1 | Hepatotoxic | Known |
| CID4788 | SLC16A7 | Non-hepatotoxic | Known |
| CID4788 | LMNA | Non-hepatotoxic | Known |
| CID4788 | POLB | Non-hepatotoxic | Known |
| CID4788 | CYP3A4 | Hepatotoxic | Known |
| CID4788 | CYP2C9 | Hepatotoxic | Known |
| CID4788 | CYP2C19 | Hepatotoxic | Known |
| CID4788 | ESR2 | Hepatotoxic | Predicted |
| CID4788 | MAPT | Non-hepatotoxic | Predicted |
| CID4788 | CYP2D6 | Hepatotoxic | Predicted |
| CID4788 | HSD17B10 | Non-hepatotoxic | Predicted |
| CID4788 | APEX1 | Non-hepatotoxic | Predicted |
| CID4788 | TP53 | Hepatotoxic | Predicted |
| CID4788 | ALDH1A1 | Non-hepatotoxic | Predicted |
| CID4788 | HIF1A | Hepatotoxic | Predicted |
| CID4788 | GAA | Non-hepatotoxic | Predicted |
| CID4788 | HPGD | Non-hepatotoxic | Predicted |
| CID4788 | TSHR | Non-hepatotoxic | Predicted |
| CID4788 | KDM4E | Non-hepatotoxic | Predicted |
| CID4788 | BLM | Non-hepatotoxic | Predicted |
| CID4788 | PSIP1 | Non-hepatotoxic | Predicted |
| CID4788 | ALOX15 | Non-hepatotoxic | Predicted |
| CID4788 | MAPK1 | Non-hepatotoxic | Predicted |
| CID4788 | NPC1 | Non-hepatotoxic | Predicted |
| CID4788 | TDP1 | Non-hepatotoxic | Predicted |
| CID4788 | THPO | Non-hepatotoxic | Predicted |
| CID4788 | SMN1 | Non-hepatotoxic | Predicted |
| CID5422 | USP2 | Non-hepatotoxic | Known |
| CID6199 | NFKB1 | Hepatotoxic | Known |
| CID6199 | ESR1 | Hepatotoxic | Known |
| CID6199 | ESR2 | Hepatotoxic | Known |
| CID6199 | ACHE | Non-hepatotoxic | Known |
| CID6199 | CYP2A6 | Hepatotoxic | Known |
| CID6199 | CRH | Non-hepatotoxic | Known |
| CID6199 | CYP2D6 | Hepatotoxic | Known |
| CID6199 | CYP2C9 | Hepatotoxic | Known |
| CID6199 | CYP2C19 | Hepatotoxic | Known |
| CID6199 | CYP3A4 | Hepatotoxic | Predicted |
| CID6199 | CYP2A13 | Hepatotoxic | Predicted |
| CID6199 | BCHE | Non-hepatotoxic | Predicted |
| CID6199 | LMNA | Non-hepatotoxic | Predicted |
| CID6199 | HIF1A | Hepatotoxic | Predicted |
| CID6199 | TSHR | Non-hepatotoxic | Predicted |
| CID6199 | MAPT | Non-hepatotoxic | Predicted |
| CID6199 | HSD17B10 | Non-hepatotoxic | Predicted |
| CID6199 | ALDH1A1 | Non-hepatotoxic | Predicted |
| CID6199 | THPO | Non-hepatotoxic | Predicted |
| CID6199 | TP53 | Hepatotoxic | Predicted |
| CID6199 | RELA | Hepatotoxic | Predicted |
| CID6199 | MAPK1 | Non-hepatotoxic | Predicted |
| CID6199 | CYP1A2 | Hepatotoxic | Predicted |
| CID6199 | TAAR1 | Non-hepatotoxic | Predicted |
| CID6199 | NPC1 | Non-hepatotoxic | Predicted |
| CID6199 | SMN1 | Non-hepatotoxic | Predicted |
| CID6199 | PARP1 | Hepatotoxic | Predicted |
| CID6199 | ALOX15 | Non-hepatotoxic | Predicted |
| CID6199 | RAB9A | Non-hepatotoxic | Predicted |
| CID6293 | BCL2 | Non-hepatotoxic | Known |
| CID6293 | MCL1 | Hepatotoxic | Known |
| CID6293 | MAPT | Non-hepatotoxic | Known |
| CID6293 | ALOX15 | Non-hepatotoxic | Known |
| CID6293 | MAPK1 | Non-hepatotoxic | Known |
| CID6293 | PGD | Hepatotoxic | Predicted |
| CID6293 | DNMT1 | Non-hepatotoxic | Predicted |
| CID6293 | KDM4E | Non-hepatotoxic | Predicted |
| CID6293 | CYP3A4 | Hepatotoxic | Predicted |
| CID6293 | ALOX12 | Non-hepatotoxic | Predicted |
| CID6293 | POLB | Non-hepatotoxic | Predicted |
| CID6293 | KMT2A | Non-hepatotoxic | Predicted |
| CID6293 | APEX1 | Non-hepatotoxic | Predicted |
| CID6293 | TDP1 | Non-hepatotoxic | Predicted |
| CID6293 | RECQL | Non-hepatotoxic | Predicted |
| CID6293 | ALDH1A1 | Non-hepatotoxic | Predicted |
| CID6293 | HPGD | Non-hepatotoxic | Predicted |
| CID6293 | HSD17B10 | Non-hepatotoxic | Predicted |
| CID6293 | L3MBTL1 | Non-hepatotoxic | Predicted |
| CID6293 | TP53 | Hepatotoxic | Predicted |
| CID6293 | USP2 | Non-hepatotoxic | Predicted |
| CID6293 | MET | Hepatotoxic | Predicted |
| CID6293 | PSIP1 | Non-hepatotoxic | Predicted |
| CID6293 | LMNA | Non-hepatotoxic | Predicted |
| CID6293 | NPC1 | Non-hepatotoxic | Predicted |
| CID6989 | ELANE | Hepatotoxic | Known |
| CID6989 | TRPA1 | Non-hepatotoxic | Known |
| CID6989 | LMNA | Non-hepatotoxic | Known |
| CID6989 | MIF | Hepatotoxic | Predicted |
| CID6989 | ALDH1A1 | Non-hepatotoxic | Predicted |
| CID6989 | CYP2E1 | Hepatotoxic | Predicted |
| CID6989 | CTSL | Non-hepatotoxic | Predicted |
| CID6989 | CTSB | Non-hepatotoxic | Predicted |
| CID6989 | TSHR | Non-hepatotoxic | Predicted |
| CID6989 | HPGD | Non-hepatotoxic | Predicted |
| CID6989 | CYP2A13 | Hepatotoxic | Predicted |
| CID6989 | HTT | Non-hepatotoxic | Predicted |
| CID6989 | PTGS1 | Non-hepatotoxic | Predicted |
| CID6989 | BLM | Non-hepatotoxic | Predicted |
| CID6989 | TERT | Hepatotoxic | Predicted |
| CID6989 | TRPV1 | Non-hepatotoxic | Predicted |
| CID6989 | MAPT | Non-hepatotoxic | Predicted |
| CID6989 | HSD17B10 | Non-hepatotoxic | Predicted |
| CID6989 | RAB9A | Non-hepatotoxic | Predicted |
| CID6989 | CYP3A4 | Hepatotoxic | Predicted |
| CID6989 | ACHE | Non-hepatotoxic | Predicted |
| CID6989 | HIF1A | Hepatotoxic | Predicted |
| CID6989 | NPC1 | Non-hepatotoxic | Predicted |
| CID7362 | HBB | Hepatotoxic | Known |
| CID7362 | HBA1; | Non-hepatotoxic | Known |
| CID8063 | LYZ | Non-hepatotoxic | Known |
| CID8063 | PRKACA | Non-hepatotoxic | Known |
| CID8093 | ALOX5 | Non-hepatotoxic | Known |
| CID8130 | TSHR | Non-hepatotoxic | Known |
| CID8130 | LMNA | Non-hepatotoxic | Predicted |
| CID8130 | TP53 | Hepatotoxic | Predicted |
| CID8130 | LTA4H | Non-hepatotoxic | Predicted |
| CID8130 | TBXAS1 | Non-hepatotoxic | Predicted |
| CID8130 | NFKB1 | Hepatotoxic | Predicted |
| CID8130 | DAO | Non-hepatotoxic | Predicted |
| CID8130 | THPO | Non-hepatotoxic | Predicted |
| CID8130 | CYP2C19 | Hepatotoxic | Predicted |
| CID8130 | TDP1 | Non-hepatotoxic | Predicted |
| CID8130 | CYP3A4 | Hepatotoxic | Predicted |
| CID8130 | HPGD | Non-hepatotoxic | Predicted |
| CID8130 | HSD17B10 | Non-hepatotoxic | Predicted |
| CID8130 | MAPK1 | Non-hepatotoxic | Predicted |
| CID8130 | CYP2D6 | Hepatotoxic | Predicted |
| CID8130 | HIF1A | Hepatotoxic | Predicted |
| CID8130 | ALOX15 | Non-hepatotoxic | Predicted |
| CID8130 | ALDH1A1 | Non-hepatotoxic | Predicted |
| CID8130 | NPSR1 | Non-hepatotoxic | Predicted |
| CID8130 | CYP1A2 | Hepatotoxic | Predicted |
| CID8130 | MAPT | Non-hepatotoxic | Predicted |
| CID8163 | ACOT13 | Non-hepatotoxic | Known |
| CID8181 | IL10 | Hepatotoxic | Known |
| CID8181 | PTGS2 | Hepatotoxic | Known |
| CID8181 | IL6 | Hepatotoxic | Known |
| CID8181 | TNF | Hepatotoxic | Known |
| CID8181 | PTGER3 | Non-hepatotoxic | Known |
| CID8181 | RELA | Hepatotoxic | Known |
| CID8186 | OR1D2 | Non-hepatotoxic | Known |
| CID8221 | LMNA | Non-hepatotoxic | Known |
| CID8221 | TSHR | Non-hepatotoxic | Predicted |
| CID8221 | BLM | Non-hepatotoxic | Predicted |
| CID8221 | HTT | Non-hepatotoxic | Predicted |
| CID8221 | ALDH1A1 | Non-hepatotoxic | Predicted |
| CID8221 | MAPT | Non-hepatotoxic | Predicted |
| CID8221 | HSD17B10 | Non-hepatotoxic | Predicted |
| CID8221 | ACHE | Non-hepatotoxic | Predicted |
| CID8221 | RAB9A | Non-hepatotoxic | Predicted |
| CID8221 | CYP3A4 | Hepatotoxic | Predicted |
| CID8221 | HIF1A | Hepatotoxic | Predicted |
| CID8221 | SMN1 | Non-hepatotoxic | Predicted |
| CID8221 | TP53 | Hepatotoxic | Predicted |
| CID8221 | NPC1 | Non-hepatotoxic | Predicted |
| CID8221 | CYP2D6 | Hepatotoxic | Predicted |
| CID8221 | CYP2C19 | Hepatotoxic | Predicted |
| CID8221 | TRPA1 | Non-hepatotoxic | Predicted |
| CID8221 | ALOX15 | Non-hepatotoxic | Predicted |
| CID8221 | PMP22 | Non-hepatotoxic | Predicted |
| CID8221 | GSG2 | Non-hepatotoxic | Predicted |
| CID8221 | TDP1 | Non-hepatotoxic | Predicted |
| CID8434 | CA12 | Non-hepatotoxic | Known |
| CID8434 | CA1 | Non-hepatotoxic | Known |
| CID8434 | CA2 | Non-hepatotoxic | Known |
| CID8434 | CA9 | Non-hepatotoxic | Known |
| CID8434 | CA14 | Non-hepatotoxic | Known |
| CID8434 | CA7 | Non-hepatotoxic | Known |
| CID8434 | ESR2 | Hepatotoxic | Known |
| CID8434 | CA13 | Non-hepatotoxic | Predicted |
| CID8434 | ESR1 | Hepatotoxic | Predicted |
| CID8434 | CA6 | Non-hepatotoxic | Predicted |
| CID8434 | CA4 | Non-hepatotoxic | Predicted |
| CID8434 | CA5A | Non-hepatotoxic | Predicted |
| CID8434 | CA5B | Non-hepatotoxic | Predicted |
| CID8434 | CA3 | Non-hepatotoxic | Predicted |
| CID8434 | HSD17B10 | Non-hepatotoxic | Predicted |
| CID8434 | HSD17B3 | Non-hepatotoxic | Predicted |
| CID8434 | UGT1A1 | Hepatotoxic | Predicted |
| CID8434 | LMNA | Non-hepatotoxic | Predicted |
| CID8434 | AR | Hepatotoxic | Predicted |
| CID8434 | MB | Non-hepatotoxic | Predicted |
| CID8434 | GLA | Non-hepatotoxic | Predicted |
| CID8434 | KDM4E | Non-hepatotoxic | Predicted |
| CID8434 | CYP3A4 | Hepatotoxic | Predicted |
| CID8434 | MAPT | Non-hepatotoxic | Predicted |
| CID8434 | ESRRG | Non-hepatotoxic | Predicted |
| CID8434 | TDP1 | Non-hepatotoxic | Predicted |
| CID8434 | HPGD | Non-hepatotoxic | Predicted |
| CID8768 | ODC1 | Non-hepatotoxic | Known |
| CID8768 | KDM4E | Non-hepatotoxic | Known |
| CID8768 | RAB9A | Non-hepatotoxic | Known |
| CID8768 | NPC1 | Non-hepatotoxic | Predicted |
| CID8768 | MAPT | Non-hepatotoxic | Predicted |
| CID8768 | HSD17B10 | Non-hepatotoxic | Predicted |
| CID8768 | THRB | Non-hepatotoxic | Predicted |
| CID8768 | ALOX15 | Non-hepatotoxic | Predicted |
| CID8768 | LMNA | Non-hepatotoxic | Predicted |
| CID8768 | TP53 | Hepatotoxic | Predicted |
| CID8768 | APEX1 | Non-hepatotoxic | Predicted |
| CID8768 | RECQL | Non-hepatotoxic | Predicted |
| CID8768 | CYP3A4 | Hepatotoxic | Predicted |
| CID8768 | RELA | Hepatotoxic | Predicted |
| CID8768 | KMT2A | Non-hepatotoxic | Predicted |
| CID8768 | NPSR1 | Non-hepatotoxic | Predicted |
| CID8768 | TDP1 | Non-hepatotoxic | Predicted |
| CID8768 | L3MBTL1 | Non-hepatotoxic | Predicted |
| CID8768 | ALDH1A1 | Non-hepatotoxic | Predicted |
| CID8768 | HPGD | Non-hepatotoxic | Predicted |
| CID8768 | NFKB1 | Hepatotoxic | Predicted |
| CID8768 | MAPK1 | Non-hepatotoxic | Predicted |
| CID8768 | TNKS | Non-hepatotoxic | Predicted |
| CID8857 | CES1 | Hepatotoxic | Known |
| CID8857 | LMNA | Non-hepatotoxic | Known |
| CID8857 | TSHR | Non-hepatotoxic | Predicted |
| CID8857 | BLM | Non-hepatotoxic | Predicted |
| CID8857 | HTT | Non-hepatotoxic | Predicted |
| CID8857 | MAPT | Non-hepatotoxic | Predicted |
| CID8857 | ALDH1A1 | Non-hepatotoxic | Predicted |
| CID8857 | HSD17B10 | Non-hepatotoxic | Predicted |
| CID8857 | CYP3A4 | Hepatotoxic | Predicted |
| CID8857 | ACHE | Non-hepatotoxic | Predicted |
| CID8857 | RAB9A | Non-hepatotoxic | Predicted |
| CID8857 | HIF1A | Hepatotoxic | Predicted |
| CID8857 | TP53 | Hepatotoxic | Predicted |
| CID8857 | SMN1 | Non-hepatotoxic | Predicted |
| CID8857 | NPC1 | Non-hepatotoxic | Predicted |
| CID8857 | CYP2D6 | Hepatotoxic | Predicted |
| CID8857 | CYP2C19 | Hepatotoxic | Predicted |
| CID8857 | TRPA1 | Non-hepatotoxic | Predicted |
| CID8857 | ALOX15 | Non-hepatotoxic | Predicted |
| CID8857 | PMP22 | Non-hepatotoxic | Predicted |
| CID8857 | TDP1 | Non-hepatotoxic | Predicted |
| CID8857 | GSG2 | Non-hepatotoxic | Predicted |
| CID10207 | MYC | Hepatotoxic | Known |
| CID10207 | IL1B | Hepatotoxic | Known |
| CID10207 | TNF | Hepatotoxic | Known |
| CID10207 | CASP3 | Hepatotoxic | Known |
| CID10207 | PRKCE | Hepatotoxic | Known |
| CID10207 | PRKCD | Non-hepatotoxic | Known |
| CID10207 | KMT2A | Non-hepatotoxic | Known |
| CID10207 | CCNA2 | Non-hepatotoxic | Known |
| CID10207 | CDK2 | Non-hepatotoxic | Known |
| CID10207 | PCNA | Non-hepatotoxic | Known |
| CID10207 | PRKCA | Hepatotoxic | Known |
| CID10207 | CCNB1 | Non-hepatotoxic | Known |
| CID10207 | CDK1 | Hepatotoxic | Known |
| CID10207 | CDKN1A | Non-hepatotoxic | Known |
| CID10207 | FASN | Non-hepatotoxic | Known |
| CID10207 | BAX | Hepatotoxic | Known |
| CID10207 | EIF6 | Non-hepatotoxic | Known |
| CID10207 | MAPT | Non-hepatotoxic | Predicted |
| CID10207 | GSG2 | Non-hepatotoxic | Predicted |
| CID10207 | MAPK1 | Non-hepatotoxic | Predicted |
| CID10207 | ALOX15 | Non-hepatotoxic | Predicted |
| CID10207 | PIM1 | Non-hepatotoxic | Predicted |
| CID10207 | RAB9A | Non-hepatotoxic | Predicted |
| CID10207 | ALDH1A1 | Non-hepatotoxic | Predicted |
| CID10207 | ATR | Non-hepatotoxic | Predicted |
| CID10207 | GAA | Non-hepatotoxic | Predicted |
| CID10207 | BRCA1 | Non-hepatotoxic | Predicted |
| CID10207 | NPC1 | Non-hepatotoxic | Predicted |
| CID10207 | USP2 | Non-hepatotoxic | Predicted |
| CID10207 | HSD17B10 | Non-hepatotoxic | Predicted |
| CID10207 | ALOX5 | Non-hepatotoxic | Predicted |
| CID10207 | TP53 | Hepatotoxic | Known |
| CID10207 | LMNA | Non-hepatotoxic | Predicted |
| CID10207 | POLB | Non-hepatotoxic | Predicted |
| CID10207 | CYP3A4 | Hepatotoxic | Predicted |
| CID10207 | NPSR1 | Non-hepatotoxic | Predicted |
| CID10207 | CYP2C9 | Hepatotoxic | Predicted |
| CID10208 | MAPT | Non-hepatotoxic | Known |
| CID10208 | MAPK1 | Non-hepatotoxic | Known |
| CID10208 | RECQL | Non-hepatotoxic | Known |
| CID10208 | TDP1 | Non-hepatotoxic | Predicted |
| CID10208 | CYP3A4 | Hepatotoxic | Predicted |
| CID10208 | ALOX15 | Non-hepatotoxic | Predicted |
| CID10208 | KDM4E | Non-hepatotoxic | Predicted |
| CID10208 | APEX1 | Non-hepatotoxic | Predicted |
| CID10208 | HPGD | Non-hepatotoxic | Predicted |
| CID10208 | KMT2A | Non-hepatotoxic | Predicted |
| CID10208 | ALDH1A1 | Non-hepatotoxic | Predicted |
| CID10208 | HSD17B10 | Non-hepatotoxic | Predicted |
| CID10208 | LMNA | Non-hepatotoxic | Predicted |
| CID10208 | BLM | Non-hepatotoxic | Predicted |
| CID10208 | TP53 | Hepatotoxic | Predicted |
| CID10208 | POLB | Non-hepatotoxic | Predicted |
| CID10208 | SMN1 | Non-hepatotoxic | Predicted |
| CID10208 | USP2 | Non-hepatotoxic | Predicted |
| CID10208 | RAB9A | Non-hepatotoxic | Predicted |
| CID10208 | NPC1 | Non-hepatotoxic | Predicted |
| CID10208 | L3MBTL1 | Non-hepatotoxic | Predicted |
| CID10208 | NFKB1 | Hepatotoxic | Predicted |
| CID10208 | TSHR | Non-hepatotoxic | Predicted |
| CID10256 | XDH | Hepatotoxic | Known |
| CID10364 | PTGS1 | Non-hepatotoxic | Known |
| CID10364 | TRPA1 | Non-hepatotoxic | Known |
| CID10364 | MIF | Hepatotoxic | Predicted |
| CID10364 | PTGS2 | Hepatotoxic | Predicted |
| CID10364 | ALDH1A1 | Non-hepatotoxic | Predicted |
| CID10364 | STK33 | Non-hepatotoxic | Predicted |
| CID10364 | PDE4B | Non-hepatotoxic | Predicted |
| CID10364 | CYP2E1 | Hepatotoxic | Predicted |
| CID10364 | ALOX5 | Non-hepatotoxic | Predicted |
| CID10364 | LMNA | Non-hepatotoxic | Predicted |
| CID10364 | CTSL | Non-hepatotoxic | Predicted |
| CID10364 | CTSB | Non-hepatotoxic | Predicted |
| CID10364 | FASN | Non-hepatotoxic | Predicted |
| CID10364 | TRPV1 | Non-hepatotoxic | Predicted |
| CID10364 | PDE4D | Non-hepatotoxic | Predicted |
| CID10364 | ITGA2B | Non-hepatotoxic | Predicted |
| CID10364 | HPGD | Non-hepatotoxic | Predicted |
| CID10364 | CYP2A13 | Hepatotoxic | Predicted |
| CID10364 | SLC5A2 | Non-hepatotoxic | Predicted |
| CID10364 | TERT | Hepatotoxic | Predicted |
| CID10364 | BCR | Non-hepatotoxic | Predicted |
| CID10364 | ITGB3 | Hepatotoxic | Predicted |
| CID10393 | CA2 | Non-hepatotoxic | Known |
| CID10393 | CA5A | Non-hepatotoxic | Known |
| CID10393 | CA5B | Non-hepatotoxic | Known |
| CID10393 | CA1 | Non-hepatotoxic | Predicted |
| CID10393 | CA14 | Non-hepatotoxic | Predicted |
| CID10393 | CA12 | Non-hepatotoxic | Predicted |
| CID10393 | CA6 | Non-hepatotoxic | Predicted |
| CID10393 | CA7 | Non-hepatotoxic | Predicted |
| CID10393 | CA4 | Non-hepatotoxic | Predicted |
| CID10393 | CA9 | Non-hepatotoxic | Predicted |
| CID10393 | CA3 | Non-hepatotoxic | Predicted |
| CID10393 | HCAR2 | Non-hepatotoxic | Predicted |
| CID10393 | LMNA | Non-hepatotoxic | Predicted |
| CID10393 | TSHR | Non-hepatotoxic | Predicted |
| CID10393 | CASP2 | Non-hepatotoxic | Predicted |
| CID10393 | CYP3A4 | Hepatotoxic | Predicted |
| CID10393 | THPO | Non-hepatotoxic | Predicted |
| CID10393 | CA13 | Non-hepatotoxic | Predicted |
| CID10393 | HSD17B10 | Non-hepatotoxic | Predicted |
| CID10393 | TDP1 | Non-hepatotoxic | Predicted |
| CID10393 | HRH4 | Non-hepatotoxic | Predicted |
| CID10393 | SERPINE1 | Hepatotoxic | Predicted |
| CID10393 | MTOR | Non-hepatotoxic | Predicted |
| CID10639 | ELANE | Hepatotoxic | Known |
| CID10639 | MAPT | Non-hepatotoxic | Known |
| CID10639 | CYP3A4 | Hepatotoxic | Predicted |
| CID10639 | TP53 | Hepatotoxic | Predicted |
| CID10639 | KMT2A | Non-hepatotoxic | Predicted |
| CID10639 | ALDH1A1 | Non-hepatotoxic | Predicted |
| CID10639 | ALOX15 | Non-hepatotoxic | Predicted |
| CID10639 | KDM4E | Non-hepatotoxic | Predicted |
| CID10639 | RAB9A | Non-hepatotoxic | Predicted |
| CID10639 | RECQL | Non-hepatotoxic | Predicted |
| CID10639 | MAPK1 | Non-hepatotoxic | Predicted |
| CID10639 | HPGD | Non-hepatotoxic | Predicted |
| CID10639 | NPC1 | Non-hepatotoxic | Predicted |
| CID10639 | LMNA | Non-hepatotoxic | Predicted |
| CID10639 | USP2 | Non-hepatotoxic | Predicted |
| CID10639 | SMN1 | Non-hepatotoxic | Predicted |
| CID10639 | TDP1 | Non-hepatotoxic | Predicted |
| CID10639 | CYP2C9 | Hepatotoxic | Predicted |
| CID10639 | CYP2C19 | Hepatotoxic | Predicted |
| CID10639 | GAA | Non-hepatotoxic | Predicted |
| CID10639 | THRB | Non-hepatotoxic | Predicted |
| CID10639 | BLM | Non-hepatotoxic | Predicted |
| CID10658 | NFKB1 | Hepatotoxic | Known |
| CID10658 | CYP1A1 | Hepatotoxic | Known |
| CID10658 | RELA | Hepatotoxic | Predicted |
| CID10658 | THPO | Non-hepatotoxic | Predicted |
| CID10658 | TSHR | Non-hepatotoxic | Predicted |
| CID10658 | HSD17B10 | Non-hepatotoxic | Predicted |
| CID10658 | HIF1A | Hepatotoxic | Predicted |
| CID10658 | CYP3A4 | Hepatotoxic | Predicted |
| CID10658 | CYP2D6 | Hepatotoxic | Predicted |
| CID10658 | NPC1 | Non-hepatotoxic | Predicted |
| CID10658 | RAB9A | Non-hepatotoxic | Predicted |
| CID10658 | NPSR1 | Non-hepatotoxic | Predicted |
| CID10658 | MAPT | Non-hepatotoxic | Predicted |
| CID10658 | LMNA | Non-hepatotoxic | Predicted |
| CID10658 | MAPK1 | Non-hepatotoxic | Predicted |
| CID10658 | ALPL | Hepatotoxic | Predicted |
| CID10658 | NFE2L2 | Non-hepatotoxic | Predicted |
| CID10658 | CYP2C19 | Hepatotoxic | Predicted |
| CID10658 | GABRR2 | Hepatotoxic | Predicted |
| CID10658 | GABRR3 | Non-hepatotoxic | Predicted |
| CID10658 | KDM4E | Non-hepatotoxic | Predicted |
| CID10658 | ALOX15 | Non-hepatotoxic | Predicted |
| CID11092 | ICAM1 | Hepatotoxic | Known |
| CID11092 | IL2 | Hepatotoxic | Known |
| CID11092 | TNF | Hepatotoxic | Known |
| CID11092 | RELA | Hepatotoxic | Known |
| CID11092 | NFKBIA | Hepatotoxic | Known |
| CID11092 | BCL2 | Non-hepatotoxic | Known |
| CID11092 | MAOA | Non-hepatotoxic | Known |
| CID11092 | BAX | Hepatotoxic | Known |
| CID11092 | MAPK1 | Non-hepatotoxic | Known |
| CID11092 | TYR | Non-hepatotoxic | Known |
| CID11092 | AKT1 | Hepatotoxic | Known |
| CID11092 | PTEN | Hepatotoxic | Known |
| CID11092 | MAOB | Hepatotoxic | Known |
| CID11092 | AHSA1 | Non-hepatotoxic | Known |
| CID11092 | CYP3A4 | Hepatotoxic | Known |
| CID11092 | MAPT | Non-hepatotoxic | Predicted |
| CID11092 | HPGD | Non-hepatotoxic | Predicted |
| CID11092 | CYP2C19 | Hepatotoxic | Predicted |
| CID11092 | CYP2D6 | Hepatotoxic | Predicted |
| CID11092 | KDM4E | Non-hepatotoxic | Predicted |
| CID11092 | SMN1 | Non-hepatotoxic | Predicted |
| CID11092 | ALOX15 | Non-hepatotoxic | Predicted |
| CID11092 | LMNA | Non-hepatotoxic | Predicted |
| CID11092 | ALDH1A1 | Non-hepatotoxic | Predicted |
| CID11092 | HSD17B10 | Non-hepatotoxic | Predicted |
| CID11092 | KMT2A | Non-hepatotoxic | Predicted |
| CID11092 | CYP2C9 | Hepatotoxic | Predicted |
| CID11092 | TSHR | Non-hepatotoxic | Predicted |
| CID11092 | TP53 | Hepatotoxic | Predicted |
| CID11092 | NFKB1 | Hepatotoxic | Predicted |
| CID11092 | USP2 | Non-hepatotoxic | Predicted |
| CID11092 | MAP2K1 | Non-hepatotoxic | Predicted |
| CID11092 | AR | Hepatotoxic | Predicted |
| CID11092 | TDP1 | Non-hepatotoxic | Predicted |
| CID11092 | RBP4 | Hepatotoxic | Predicted |
| CID11146 | MT-CO1 | Non-hepatotoxic | Known |
| CID11146 | MT-CO3 | Non-hepatotoxic | Known |
| CID11146 | COX2 | Non-hepatotoxic | Known |
| CID11146 | COX5A | Non-hepatotoxic | Known |
| CID11146 | COX5B | Non-hepatotoxic | Known |
| CID11146 | COX6A2 | Non-hepatotoxic | Known |
| CID11146 | COX6B1 | Non-hepatotoxic | Known |
| CID11146 | COX7A1 | Non-hepatotoxic | Known |
| CID11146 | COX7C | Non-hepatotoxic | Known |
| CID13165 | TSHR | Non-hepatotoxic | Known |
| CID13165 | LMNA | Non-hepatotoxic | Predicted |
| CID13165 | TP53 | Hepatotoxic | Predicted |
| CID13165 | LTA4H | Non-hepatotoxic | Predicted |
| CID13165 | TBXAS1 | Non-hepatotoxic | Predicted |
| CID13165 | NFKB1 | Hepatotoxic | Predicted |
| CID13165 | DAO | Non-hepatotoxic | Predicted |
| CID13165 | THPO | Non-hepatotoxic | Predicted |
| CID13165 | CYP2C19 | Hepatotoxic | Predicted |
| CID13165 | TDP1 | Non-hepatotoxic | Predicted |
| CID13165 | CYP3A4 | Hepatotoxic | Predicted |
| CID13165 | HPGD | Non-hepatotoxic | Predicted |
| CID13165 | MAPK1 | Non-hepatotoxic | Predicted |
| CID13165 | HSD17B10 | Non-hepatotoxic | Predicted |
| CID13165 | ALOX15 | Non-hepatotoxic | Predicted |
| CID13165 | HIF1A | Hepatotoxic | Predicted |
| CID13165 | CYP2D6 | Hepatotoxic | Predicted |
| CID13165 | NPSR1 | Non-hepatotoxic | Predicted |
| CID13165 | ALDH1A1 | Non-hepatotoxic | Predicted |
| CID13165 | CYP1A2 | Hepatotoxic | Predicted |
| CID13165 | MAPT | Non-hepatotoxic | Predicted |
| CID24699 | KMT2A | Non-hepatotoxic | Known |
| CID24699 | MAPT | Non-hepatotoxic | Predicted |
| CID24699 | GSG2 | Non-hepatotoxic | Predicted |
| CID24699 | MAPK1 | Non-hepatotoxic | Predicted |
| CID24699 | ALOX15 | Non-hepatotoxic | Predicted |
| CID24699 | PIM1 | Non-hepatotoxic | Predicted |
| CID24699 | RAB9A | Non-hepatotoxic | Predicted |
| CID24699 | ALDH1A1 | Non-hepatotoxic | Predicted |
| CID24699 | ATR | Non-hepatotoxic | Predicted |
| CID24699 | BRCA1 | Non-hepatotoxic | Predicted |
| CID24699 | GAA | Non-hepatotoxic | Predicted |
| CID24699 | NPC1 | Non-hepatotoxic | Predicted |
| CID24699 | ALOX5 | Non-hepatotoxic | Predicted |
| CID24699 | USP2 | Non-hepatotoxic | Predicted |
| CID24699 | HSD17B10 | Non-hepatotoxic | Predicted |
| CID24699 | LMNA | Non-hepatotoxic | Predicted |
| CID24699 | TP53 | Hepatotoxic | Predicted |
| CID24699 | POLB | Non-hepatotoxic | Predicted |
| CID24699 | NPSR1 | Non-hepatotoxic | Predicted |
| CID24699 | CYP3A4 | Hepatotoxic | Predicted |
| CID24699 | CYP2C9 | Hepatotoxic | Predicted |
| CID31244 | TYR | Non-hepatotoxic | Known |
| CID31244 | CYP2A6 | Hepatotoxic | Known |
| CID31244 | ALDH1A1 | Non-hepatotoxic | Known |
| CID31244 | CYP2A13 | Hepatotoxic | Predicted |
| CID31244 | TRPA1 | Non-hepatotoxic | Predicted |
| CID31244 | TAAR1 | Non-hepatotoxic | Predicted |
| CID31244 | HPGD | Non-hepatotoxic | Predicted |
| CID31244 | MAPT | Non-hepatotoxic | Predicted |
| CID31244 | CYP1A2 | Hepatotoxic | Predicted |
| CID31244 | HSD17B10 | Non-hepatotoxic | Predicted |
| CID31244 | ALOX15 | Non-hepatotoxic | Predicted |
| CID31244 | TDP1 | Non-hepatotoxic | Predicted |
| CID31244 | CYP2E1 | Hepatotoxic | Predicted |
| CID31244 | CYP3A4 | Hepatotoxic | Predicted |
| CID31244 | LMNA | Non-hepatotoxic | Predicted |
| CID31244 | MIF | Hepatotoxic | Predicted |
| CID31244 | CYP2D6 | Hepatotoxic | Predicted |
| CID31244 | TSHR | Non-hepatotoxic | Predicted |
| CID31244 | CYP2C19 | Hepatotoxic | Predicted |
| CID31244 | IMPDH2 | Non-hepatotoxic | Predicted |
| CID31244 | KDM4E | Non-hepatotoxic | Predicted |
| CID31244 | MAPK1 | Non-hepatotoxic | Predicted |
| CID31244 | CYP2C9 | Hepatotoxic | Predicted |
| CID31404 | KDR | Hepatotoxic | Known |
| CID31404 | PECAM1 | Non-hepatotoxic | Known |
| CID31404 | CA2 | Non-hepatotoxic | Known |
| CID31404 | HMOX1 | Hepatotoxic | Known |
| CID31404 | BMP4 | Non-hepatotoxic | Known |
| CID31404 | SFTPB | Non-hepatotoxic | Known |
| CID31404 | PCDH12 | Non-hepatotoxic | Known |
| CID31404 | POLB | Non-hepatotoxic | Known |
| CID31404 | SMN1 | Non-hepatotoxic | Known |
| CID31404 | CA1 | Non-hepatotoxic | Predicted |
| CID31404 | CA12 | Non-hepatotoxic | Predicted |
| CID31404 | CA14 | Non-hepatotoxic | Predicted |
| CID31404 | CA9 | Non-hepatotoxic | Predicted |
| CID31404 | CA7 | Non-hepatotoxic | Predicted |
| CID31404 | APEX1 | Non-hepatotoxic | Predicted |
| CID31404 | CA5A | Non-hepatotoxic | Predicted |
| CID31404 | CA5B | Non-hepatotoxic | Predicted |
| CID31404 | GAA | Non-hepatotoxic | Predicted |
| CID31404 | CA6 | Non-hepatotoxic | Predicted |
| CID31404 | PSIP1 | Non-hepatotoxic | Predicted |
| CID31404 | CA4 | Non-hepatotoxic | Predicted |
| CID31404 | MAPT | Non-hepatotoxic | Predicted |
| CID31404 | MCL1 | Hepatotoxic | Predicted |
| CID31404 | RECQL | Non-hepatotoxic | Predicted |
| CID31404 | CA3 | Non-hepatotoxic | Predicted |
| CID31404 | CYP3A4 | Hepatotoxic | Predicted |
| CID31404 | TDP1 | Non-hepatotoxic | Predicted |
| CID31404 | LMNA | Non-hepatotoxic | Predicted |
| CID31404 | HPGD | Non-hepatotoxic | Predicted |
| CID31423 | UGT1A1 | Hepatotoxic | Known |
| CID31423 | UGT1A6 | Hepatotoxic | Known |
| CID31423 | CYP1A1 | Hepatotoxic | Known |
| CID31423 | CYP1A2 | Hepatotoxic | Known |
| CID31423 | CYP1B1 | Hepatotoxic | Known |
| CID31423 | CXCL8 | Non-hepatotoxic | Known |
| CID31423 | CYP3A4 | Hepatotoxic | Known |
| CID31423 | ABCC1 | Hepatotoxic | Predicted |
| CID31423 | ABCB1 | Hepatotoxic | Predicted |
| CID31423 | BCR | Non-hepatotoxic | Predicted |
| CID31423 | XDH | Hepatotoxic | Predicted |
| CID31423 | CYP2C19 | Hepatotoxic | Predicted |
| CID31423 | AR | Hepatotoxic | Predicted |
| CID31423 | NR3C1 | Hepatotoxic | Predicted |
| CID31423 | GSTP1 | Hepatotoxic | Predicted |
| CID31423 | CYP2C9 | Hepatotoxic | Predicted |
| CID31423 | MAPK10 | Non-hepatotoxic | Predicted |
| CID31423 | ABL1 | Hepatotoxic | Predicted |
| CID31423 | HPGD | Non-hepatotoxic | Predicted |
| CID31423 | CYP19A1 | Hepatotoxic | Predicted |
| CID31423 | PRKDC | Hepatotoxic | Predicted |
| CID31423 | MAPT | Non-hepatotoxic | Predicted |
| CID31423 | CYP2A6 | Hepatotoxic | Predicted |
| CID31423 | CYP2D6 | Hepatotoxic | Predicted |
| CID31423 | TSHR | Non-hepatotoxic | Predicted |
| CID31423 | MAPK1 | Non-hepatotoxic | Predicted |
| CID31423 | SMN1 | Non-hepatotoxic | Predicted |
| CID72300 | CNR2 | Hepatotoxic | Known |
| CID72300 | PPARG | Hepatotoxic | Known |
| CID72300 | CNR1 | Hepatotoxic | Known |
| CID72300 | RXRA | Non-hepatotoxic | Known |
| CID72300 | MAPT | Non-hepatotoxic | Known |
| CID72300 | TRPV1 | Non-hepatotoxic | Predicted |
| CID72300 | ALOX5 | Non-hepatotoxic | Predicted |
| CID72300 | FAAH | Non-hepatotoxic | Predicted |
| CID72300 | GPR18 | Non-hepatotoxic | Predicted |
| CID72300 | GPR55 | Non-hepatotoxic | Predicted |
| CID72300 | KMT2A | Non-hepatotoxic | Predicted |
| CID72300 | CYP3A4 | Hepatotoxic | Predicted |
| CID72300 | TP53 | Hepatotoxic | Predicted |
| CID72300 | ALDH1A1 | Non-hepatotoxic | Predicted |
| CID72300 | GLRA1 | Non-hepatotoxic | Predicted |
| CID72300 | TRPA1 | Non-hepatotoxic | Predicted |
| CID72300 | ALOX15 | Non-hepatotoxic | Predicted |
| CID72300 | KDM4E | Non-hepatotoxic | Predicted |
| CID72300 | RAB9A | Non-hepatotoxic | Predicted |
| CID72300 | HPGD | Non-hepatotoxic | Predicted |
| CID72300 | MAPK1 | Non-hepatotoxic | Predicted |
| CID72300 | RECQL | Non-hepatotoxic | Predicted |
| CID72300 | LMNA | Non-hepatotoxic | Predicted |
| CID72300 | NPC1 | Non-hepatotoxic | Predicted |
| CID72300 | SMN1 | Non-hepatotoxic | Predicted |
| CID80103 | KMT2A | Non-hepatotoxic | Known |
| CID80103 | NPC1 | Non-hepatotoxic | Known |
| CID80103 | RAB9A | Non-hepatotoxic | Known |
| CID80103 | MAPT | Non-hepatotoxic | Predicted |
| CID80103 | RELA | Hepatotoxic | Predicted |
| CID80103 | LMNA | Non-hepatotoxic | Predicted |
| CID80103 | TP53 | Hepatotoxic | Predicted |
| CID80103 | KDM4E | Non-hepatotoxic | Predicted |
| CID80103 | XBP1 | Non-hepatotoxic | Predicted |
| CID80103 | MITF | Non-hepatotoxic | Predicted |
| CID80103 | NFKB1 | Hepatotoxic | Predicted |
| CID80103 | TNKS | Non-hepatotoxic | Predicted |
| CID80103 | SMN1 | Non-hepatotoxic | Predicted |
| CID80103 | NPSR1 | Non-hepatotoxic | Predicted |
| CID80103 | ABCG2 | Hepatotoxic | Predicted |
| CID80103 | CYP3A4 | Hepatotoxic | Predicted |
| CID80103 | MAPK1 | Non-hepatotoxic | Predicted |
| CID80103 | CYP2C9 | Hepatotoxic | Predicted |
| CID80103 | HTT | Non-hepatotoxic | Predicted |
| CID80103 | ALDH1A1 | Non-hepatotoxic | Predicted |
| CID80103 | RECQL | Non-hepatotoxic | Predicted |
| CID80103 | AR | Hepatotoxic | Predicted |
| CID80103 | BCL2A1 | Non-hepatotoxic | Predicted |
| CID97214 | AURKB | Non-hepatotoxic | Known |
| CID97214 | CYP1A1 | Hepatotoxic | Known |
| CID97214 | CYP1B1 | Hepatotoxic | Known |
| CID97214 | CYP1A2 | Hepatotoxic | Predicted |
| CID97214 | ABCC1 | Hepatotoxic | Predicted |
| CID97214 | BCR | Non-hepatotoxic | Predicted |
| CID97214 | ABCB1 | Hepatotoxic | Predicted |
| CID97214 | XDH | Hepatotoxic | Predicted |
| CID97214 | GSTP1 | Hepatotoxic | Predicted |
| CID97214 | CYP3A4 | Hepatotoxic | Predicted |
| CID97214 | ABL1 | Hepatotoxic | Predicted |
| CID97214 | MAPK10 | Non-hepatotoxic | Predicted |
| CID97214 | NR3C1 | Hepatotoxic | Predicted |
| CID97214 | AR | Hepatotoxic | Predicted |
| CID97214 | PRKDC | Hepatotoxic | Predicted |
| CID97214 | CYP2C19 | Hepatotoxic | Predicted |
| CID97214 | CYP2C9 | Hepatotoxic | Predicted |
| CID97214 | CYP19A1 | Hepatotoxic | Predicted |
| CID97214 | AMY1A | Non-hepatotoxic | Predicted |
| CID97214 | UGT1A3 | Hepatotoxic | Predicted |
| CID97214 | UGT1A10 | Hepatotoxic | Predicted |
| CID97214 | NPC1 | Non-hepatotoxic | Predicted |
| CID97214 | RAB9A | Non-hepatotoxic | Predicted |
| CID104817 | CRP | Hepatotoxic | Known |
| CID114917 | ICAM1 | Hepatotoxic | Known |
| CID114917 | PTPN11 | Non-hepatotoxic | Known |
| CID114917 | VEGFA | Hepatotoxic | Known |
| CID114917 | EED | Non-hepatotoxic | Known |
| CID114917 | CES2 | Hepatotoxic | Known |
| CID114917 | VCAM1 | Non-hepatotoxic | Known |
| CID114917 | PTPN6 | Non-hepatotoxic | Known |
| CID114917 | MAPT | Non-hepatotoxic | Known |
| CID114917 | CES1 | Hepatotoxic | Predicted |
| CID114917 | APAF1 | Non-hepatotoxic | Predicted |
| CID114917 | RBBP4 | Non-hepatotoxic | Predicted |
| CID114917 | TERT | Hepatotoxic | Predicted |
| CID114917 | ACHE | Non-hepatotoxic | Predicted |
| CID114917 | AKR1B1 | Non-hepatotoxic | Predicted |
| CID114917 | PRKCH | Non-hepatotoxic | Predicted |
| CID114917 | ALDH1A1 | Non-hepatotoxic | Predicted |
| CID114917 | MAPK1 | Non-hepatotoxic | Predicted |
| CID114917 | HSD11B2 | Hepatotoxic | Predicted |
| CID114917 | TDP1 | Non-hepatotoxic | Predicted |
| CID114917 | STAT3 | Hepatotoxic | Predicted |
| CID114917 | CYP3A4 | Hepatotoxic | Predicted |
| CID114917 | TP53 | Hepatotoxic | Predicted |
| CID114917 | KMT2A | Non-hepatotoxic | Predicted |
| CID114917 | HSD11B1 | Hepatotoxic | Predicted |
| CID114917 | AKR1B10 | Non-hepatotoxic | Predicted |
| CID114917 | LMNA | Non-hepatotoxic | Predicted |
| CID114917 | SLCO1B3 | Hepatotoxic | Predicted |
| CID114917 | HPGD | Non-hepatotoxic | Predicted |
| CID119389 | PPARG | Hepatotoxic | Known |
| CID119389 | CEBPA | Non-hepatotoxic | Known |
| CID119389 | SREBF1 | Hepatotoxic | Known |
| CID119389 | LPL | Hepatotoxic | Known |
| CID119389 | GPD2 | Non-hepatotoxic | Known |
| CID119389 | FABP4 | Non-hepatotoxic | Known |
| CID119389 | SCARB2 | Non-hepatotoxic | Known |
| CID119389 | LIPE | Non-hepatotoxic | Known |
| CID237332 | HBB | Hepatotoxic | Known |
| CID237332 | TSHR | Non-hepatotoxic | Known |
| CID237332 | HBA1; | Non-hepatotoxic | Known |
| CID237332 | LMNA | Non-hepatotoxic | Predicted |
| CID237332 | TP53 | Hepatotoxic | Predicted |
| CID237332 | LTA4H | Non-hepatotoxic | Predicted |
| CID237332 | TBXAS1 | Non-hepatotoxic | Predicted |
| CID237332 | NFKB1 | Hepatotoxic | Predicted |
| CID237332 | DAO | Non-hepatotoxic | Predicted |
| CID237332 | THPO | Non-hepatotoxic | Predicted |
| CID237332 | CYP2C19 | Hepatotoxic | Predicted |
| CID237332 | TDP1 | Non-hepatotoxic | Predicted |
| CID237332 | CYP3A4 | Hepatotoxic | Predicted |
| CID237332 | HPGD | Non-hepatotoxic | Predicted |
| CID237332 | MAPK1 | Non-hepatotoxic | Predicted |
| CID237332 | HSD17B10 | Non-hepatotoxic | Predicted |
| CID237332 | ALOX15 | Non-hepatotoxic | Predicted |
| CID237332 | HIF1A | Hepatotoxic | Predicted |
| CID237332 | CYP2D6 | Hepatotoxic | Predicted |
| CID237332 | ALDH1A1 | Non-hepatotoxic | Predicted |
| CID237332 | CYP2A6 | Hepatotoxic | Predicted |
| CID237332 | NPSR1 | Non-hepatotoxic | Predicted |
| CID237332 | CYP1A2 | Hepatotoxic | Predicted |
| CID251561 | ADRA2A | Hepatotoxic | Known |
| CID251561 | ADRA2C | Non-hepatotoxic | Known |
| CID637511 | TLR4 | Hepatotoxic | Known |
| CID637511 | C5AR1 | Hepatotoxic | Known |
| CID637511 | TRPV1 | Non-hepatotoxic | Known |
| CID637511 | NFKBIA | Hepatotoxic | Known |
| CID637511 | NFE2L2 | Non-hepatotoxic | Known |
| CID637511 | RELA | Hepatotoxic | Known |
| CID637511 | PTGS2 | Hepatotoxic | Known |
| CID637511 | IFNB1 | Hepatotoxic | Known |
| CID637511 | IRF3 | Non-hepatotoxic | Known |
| CID637511 | TXNRD1 | Non-hepatotoxic | Known |
| CID637511 | TRPA1 | Non-hepatotoxic | Known |
| CID637511 | TRPV4 | Non-hepatotoxic | Known |
| CID637511 | ALDH1A1 | Non-hepatotoxic | Known |
| CID637511 | LMNA | Non-hepatotoxic | Known |
| CID637511 | MIF | Hepatotoxic | Predicted |
| CID637511 | HPGD | Non-hepatotoxic | Predicted |
| CID637511 | MAPT | Non-hepatotoxic | Predicted |
| CID637511 | CYP2E1 | Hepatotoxic | Predicted |
| CID637511 | HSD17B10 | Non-hepatotoxic | Predicted |
| CID637511 | TSHR | Non-hepatotoxic | Predicted |
| CID637511 | CYP2A6 | Hepatotoxic | Predicted |
| CID637511 | CYP3A4 | Hepatotoxic | Predicted |
| CID637511 | CTSL | Non-hepatotoxic | Predicted |
| CID637511 | ALOX15 | Non-hepatotoxic | Predicted |
| CID637511 | TDP1 | Non-hepatotoxic | Predicted |
| CID637511 | CTSB | Non-hepatotoxic | Predicted |
| CID637511 | HIF1A | Hepatotoxic | Predicted |
| CID637511 | HTT | Non-hepatotoxic | Predicted |
| CID637511 | TP53 | Hepatotoxic | Predicted |
| CID637511 | KDM4E | Non-hepatotoxic | Predicted |
| CID637511 | SMN1 | Non-hepatotoxic | Predicted |
| CID637511 | BLM | Non-hepatotoxic | Predicted |
| CID637511 | KMT2A | Non-hepatotoxic | Predicted |
| CID637511 | PTGS1 | Non-hepatotoxic | Predicted |
| CID638278 | JAK2 | Hepatotoxic | Known |
| CID638278 | GBA | Non-hepatotoxic | Known |
| CID638278 | FOS | Non-hepatotoxic | Known |
| CID638278 | VCAM1 | Non-hepatotoxic | Known |
| CID638278 | SELE | Hepatotoxic | Known |
| CID638278 | TYR | Non-hepatotoxic | Known |
| CID638278 | SLC2A1 | Hepatotoxic | Known |
| CID638278 | BAX | Hepatotoxic | Known |
| CID638278 | AKR1B1 | Non-hepatotoxic | Known |
| CID638278 | GABBR1 | Non-hepatotoxic | Known |
| CID638278 | F11R | Non-hepatotoxic | Known |
| CID638278 | MT2A | Non-hepatotoxic | Known |
| CID638278 | LMNA | Non-hepatotoxic | Known |
| CID638278 | CYP3A4 | Hepatotoxic | Known |
| CID638278 | MAPT | Non-hepatotoxic | Known |
| CID638278 | CYP2C9 | Hepatotoxic | Known |
| CID638278 | CYP2C19 | Hepatotoxic | Known |
| CID638278 | GANAB | Non-hepatotoxic | Predicted |
| CID638278 | CYP2D6 | Hepatotoxic | Predicted |
| CID638278 | SI | Non-hepatotoxic | Predicted |
| CID638278 | AGL | Non-hepatotoxic | Predicted |
| CID638278 | MGAM | Non-hepatotoxic | Predicted |
| CID638278 | TP53 | Hepatotoxic | Predicted |
| CID638278 | AKR1B10 | Non-hepatotoxic | Predicted |
| CID638278 | HSD17B10 | Non-hepatotoxic | Predicted |
| CID638278 | ALDH1A1 | Non-hepatotoxic | Predicted |
| CID638278 | ALOX15 | Non-hepatotoxic | Predicted |
| CID638278 | TSHR | Non-hepatotoxic | Predicted |
| CID638278 | GAA | Non-hepatotoxic | Predicted |
| CID638278 | HPGD | Non-hepatotoxic | Predicted |
| CID638278 | KMT2A | Non-hepatotoxic | Predicted |
| CID638278 | HIF1A | Hepatotoxic | Predicted |
| CID638278 | MAPK1 | Non-hepatotoxic | Predicted |
| CID638278 | SMN1 | Non-hepatotoxic | Predicted |
| CID638278 | RECQL | Non-hepatotoxic | Predicted |
| CID638278 | ALOX12 | Non-hepatotoxic | Predicted |
| CID638278 | RAB9A | Non-hepatotoxic | Predicted |
| CID641785 | RELA | Hepatotoxic | Known |
| CID641785 | PTGS2 | Hepatotoxic | Known |
| CID641785 | NOS2 | Hepatotoxic | Known |
| CID641785 | AKT1 | Hepatotoxic | Known |
| CID641785 | KCNA3 | Non-hepatotoxic | Known |
| CID641785 | CYP3A4 | Hepatotoxic | Predicted |
| CID641785 | MAPT | Non-hepatotoxic | Predicted |
| CID641785 | ABCG2 | Hepatotoxic | Predicted |
| CID641785 | CYP19A1 | Hepatotoxic | Predicted |
| CID641785 | MAOA | Non-hepatotoxic | Predicted |
| CID641785 | RAB9A | Non-hepatotoxic | Predicted |
| CID641785 | NPC1 | Non-hepatotoxic | Predicted |
| CID641785 | ALDH1A1 | Non-hepatotoxic | Predicted |
| CID641785 | KMT2A | Non-hepatotoxic | Predicted |
| CID641785 | ABCB1 | Hepatotoxic | Predicted |
| CID641785 | TP53 | Hepatotoxic | Predicted |
| CID641785 | LMNA | Non-hepatotoxic | Predicted |
| CID641785 | BACE1 | Non-hepatotoxic | Predicted |
| CID641785 | ACHE | Non-hepatotoxic | Predicted |
| CID641785 | MAPK1 | Non-hepatotoxic | Predicted |
| CID641785 | KDM4E | Non-hepatotoxic | Predicted |
| CID641785 | CYP2C19 | Hepatotoxic | Predicted |
| CID641785 | TSHR | Non-hepatotoxic | Predicted |
| CID641785 | PTPN1 | Non-hepatotoxic | Predicted |
| CID641785 | TDP1 | Non-hepatotoxic | Predicted |
| CID853433 | IL2 | Hepatotoxic | Known |
| CID853433 | RELA | Hepatotoxic | Known |
| CID853433 | NFATC3 | Non-hepatotoxic | Known |
| CID853433 | LPO | Non-hepatotoxic | Known |
| CID5273469 | LMNA | Non-hepatotoxic | Known |
| CID5273469 | TSHR | Non-hepatotoxic | Predicted |
| CID5273469 | BLM | Non-hepatotoxic | Predicted |
| CID5273469 | HTT | Non-hepatotoxic | Predicted |
| CID5273469 | MAPT | Non-hepatotoxic | Predicted |
| CID5273469 | ALDH1A1 | Non-hepatotoxic | Predicted |
| CID5273469 | HSD17B10 | Non-hepatotoxic | Predicted |
| CID5273469 | CYP3A4 | Hepatotoxic | Predicted |
| CID5273469 | RAB9A | Non-hepatotoxic | Predicted |
| CID5273469 | ACHE | Non-hepatotoxic | Predicted |
| CID5273469 | HIF1A | Hepatotoxic | Predicted |
| CID5273469 | TP53 | Hepatotoxic | Predicted |
| CID5273469 | SMN1 | Non-hepatotoxic | Predicted |
| CID5273469 | NPC1 | Non-hepatotoxic | Predicted |
| CID5273469 | CYP2D6 | Hepatotoxic | Predicted |
| CID5273469 | CYP2C19 | Hepatotoxic | Predicted |
| CID5273469 | TRPA1 | Non-hepatotoxic | Predicted |
| CID5273469 | ALOX15 | Non-hepatotoxic | Predicted |
| CID5273469 | TDP1 | Non-hepatotoxic | Predicted |
| CID5273469 | PMP22 | Non-hepatotoxic | Predicted |
| CID5273469 | GSG2 | Non-hepatotoxic | Predicted |
| CID5273621 | SMN1 | Non-hepatotoxic | Known |
| CID5273621 | CYP3A4 | Hepatotoxic | Predicted |
| CID5273621 | TP53 | Hepatotoxic | Predicted |
| CID5273621 | MAPT | Non-hepatotoxic | Predicted |
| CID5273621 | LMNA | Non-hepatotoxic | Predicted |
| CID5273621 | PARP1 | Hepatotoxic | Predicted |
| CID5273621 | HSD17B10 | Non-hepatotoxic | Predicted |
| CID5273621 | MAPK1 | Non-hepatotoxic | Predicted |
| CID5273621 | NPC1 | Non-hepatotoxic | Predicted |
| CID5273621 | IDO1 | Non-hepatotoxic | Predicted |
| CID5273621 | RAB9A | Non-hepatotoxic | Predicted |
| CID5273621 | HPGD | Non-hepatotoxic | Predicted |
| CID5273621 | ALDH1A1 | Non-hepatotoxic | Predicted |
| CID5273621 | CYP2C19 | Hepatotoxic | Predicted |
| CID5273621 | CASP1 | Non-hepatotoxic | Predicted |
| CID5273621 | MAOB | Hepatotoxic | Predicted |
| CID5273621 | THPO | Non-hepatotoxic | Predicted |
| CID5273621 | CYP2D6 | Hepatotoxic | Predicted |
| CID5273621 | TSHR | Non-hepatotoxic | Predicted |
| CID5273621 | HTT | Non-hepatotoxic | Predicted |
| CID5273621 | HSP90AB1 | Non-hepatotoxic | Predicted |
| CID5280343 | JUN | Hepatotoxic | Known |
| CID5280343 | GJA1 | Non-hepatotoxic | Known |
| CID5280343 | ICAM1 | Hepatotoxic | Known |
| CID5280343 | IFNG | Hepatotoxic | Known |
| CID5280343 | IL10 | Hepatotoxic | Known |
| CID5280343 | BIRC5 | Non-hepatotoxic | Known |
| CID5280343 | STAT6 | Non-hepatotoxic | Known |
| CID5280343 | ABCB1 | Hepatotoxic | Known |
| CID5280343 | ALOX5 | Non-hepatotoxic | Known |
| CID5280343 | EGFR | Hepatotoxic | Known |
| CID5280343 | MAPK14 | Hepatotoxic | Known |
| CID5280343 | MMP9 | Hepatotoxic | Known |
| CID5280343 | PIK3R1 | Non-hepatotoxic | Known |
| CID5280343 | STK17B | Non-hepatotoxic | Known |
| CID5280343 | PARP1 | Hepatotoxic | Known |
| CID5280343 | PPARG | Hepatotoxic | Known |
| CID5280343 | ESR1 | Hepatotoxic | Known |
| CID5280343 | PTGS2 | Hepatotoxic | Known |
| CID5280343 | PTGER3 | Non-hepatotoxic | Known |
| CID5280343 | RELA | Hepatotoxic | Known |
| CID5280343 | TNF | Hepatotoxic | Known |
| CID5280343 | AHR | Hepatotoxic | Known |
| CID5280343 | RB1 | Hepatotoxic | Known |
| CID5280343 | IL6 | Hepatotoxic | Known |
| CID5280343 | IL1B | Hepatotoxic | Known |
| CID5280343 | BCL2 | Non-hepatotoxic | Known |
| CID5280343 | CASP3 | Hepatotoxic | Known |
| CID5280343 | IL2 | Hepatotoxic | Known |
| CID5280343 | SOD1 | Hepatotoxic | Known |
| CID5280343 | CASP8 | Hepatotoxic | Known |
| CID5280343 | CCL2 | Hepatotoxic | Known |
| CID5280343 | HIF1A | Hepatotoxic | Known |
| CID5280343 | TP53 | Hepatotoxic | Known |
| CID5280343 | VEGFA | Hepatotoxic | Known |
| CID5280343 | NFE2L2 | Non-hepatotoxic | Known |
| CID5280343 | CRP | Hepatotoxic | Known |
| CID5280343 | CHUK | Hepatotoxic | Known |
| CID5280343 | STAT1 | Hepatotoxic | Known |
| CID5280343 | SPP1 | Hepatotoxic | Known |
| CID5280343 | TGFB1 | Hepatotoxic | Known |
| CID5280343 | MYC | Hepatotoxic | Known |
| CID5280343 | NFKBIA | Hepatotoxic | Known |
| CID5280343 | ALOX12 | Non-hepatotoxic | Known |
| CID5280343 | AXL | Non-hepatotoxic | Known |
| CID5280343 | CA12 | Non-hepatotoxic | Known |
| CID5280343 | CXCL2 | Non-hepatotoxic | Known |
| CID5280343 | CXCL10 | Non-hepatotoxic | Known |
| CID5280343 | ACPP | Non-hepatotoxic | Known |
| CID5280343 | CD40LG | Non-hepatotoxic | Known |
| CID5280343 | IRF1 | Non-hepatotoxic | Known |
| CID5280343 | NCF1 | Non-hepatotoxic | Known |
| CID5280343 | TSHR | Non-hepatotoxic | Known |
| CID5280343 | NFKB1 | Hepatotoxic | Known |
| CID5280343 | KMT2A | Non-hepatotoxic | Known |
| CID5280343 | KDR | Hepatotoxic | Known |
| CID5280343 | CCR4 | Non-hepatotoxic | Known |
| CID5280343 | ABCG2 | Hepatotoxic | Known |
| CID5280343 | AKT1 | Hepatotoxic | Known |
| CID5280343 | ALK | Non-hepatotoxic | Known |
| CID5280343 | AURKB | Non-hepatotoxic | Known |
| CID5280343 | CA1 | Non-hepatotoxic | Known |
| CID5280343 | CA2 | Non-hepatotoxic | Known |
| CID5280343 | CA9 | Non-hepatotoxic | Known |
| CID5280343 | CDK1 | Hepatotoxic | Known |
| CID5280343 | CSNK2A1 | Non-hepatotoxic | Known |
| CID5280343 | CXCR1 | Hepatotoxic | Known |
| CID5280343 | DRD4 | Non-hepatotoxic | Known |
| CID5280343 | F2 | Hepatotoxic | Known |
| CID5280343 | FLT3 | Hepatotoxic | Known |
| CID5280343 | GLO1 | Hepatotoxic | Known |
| CID5280343 | HSD17B2 | Non-hepatotoxic | Known |
| CID5280343 | IGF1R | Hepatotoxic | Known |
| CID5280343 | MAOA | Non-hepatotoxic | Known |
| CID5280343 | MET | Hepatotoxic | Known |
| CID5280343 | MMP13 | Hepatotoxic | Known |
| CID5280343 | MPO | Hepatotoxic | Known |
| CID5280343 | NEK2 | Non-hepatotoxic | Known |
| CID5280343 | PIM1 | Non-hepatotoxic | Known |
| CID5280343 | PLK1 | Non-hepatotoxic | Known |
| CID5280343 | PTK2 | Non-hepatotoxic | Known |
| CID5280343 | XDH | Hepatotoxic | Known |
| CID5280343 | AKR1C3 | Non-hepatotoxic | Known |
| CID5280343 | ATP5A1 | Non-hepatotoxic | Known |
| CID5280343 | ATP5C1 | Non-hepatotoxic | Known |
| CID5280343 | MMP2 | Hepatotoxic | Known |
| CID5280343 | NT5E | Non-hepatotoxic | Known |
| CID5280343 | UGT1A9 | Hepatotoxic | Known |
| CID5280343 | GSTP1 | Hepatotoxic | Known |
| CID5280343 | MAPK1 | Non-hepatotoxic | Known |
| CID5280343 | TOP2A | Non-hepatotoxic | Known |
| CID5280343 | ESR2 | Hepatotoxic | Known |
| CID5280343 | PRKCB | Hepatotoxic | Known |
| CID5280343 | PTGS1 | Non-hepatotoxic | Known |
| CID5280343 | TOP1 | Non-hepatotoxic | Known |
| CID5280343 | NOS2 | Hepatotoxic | Known |
| CID5280343 | PLAT | Non-hepatotoxic | Known |
| CID5280343 | PLAU | Hepatotoxic | Known |
| CID5280343 | NR1I2 | Hepatotoxic | Known |
| CID5280343 | NR1I3 | Hepatotoxic | Known |
| CID5280343 | CCNB1 | Non-hepatotoxic | Known |
| CID5280343 | CDKN1A | Non-hepatotoxic | Known |
| CID5280343 | CHEK2 | Non-hepatotoxic | Known |
| CID5280343 | VCAM1 | Non-hepatotoxic | Known |
| CID5280343 | SELE | Hepatotoxic | Known |
| CID5280343 | BAX | Hepatotoxic | Known |
| CID5280343 | ACACA | Hepatotoxic | Known |
| CID5280343 | INSR | Hepatotoxic | Known |
| CID5280343 | SLC2A4 | Hepatotoxic | Known |
| CID5280343 | BCL2L1 | Hepatotoxic | Known |
| CID5280343 | CASP9 | Hepatotoxic | Known |
| CID5280343 | PPARA | Hepatotoxic | Known |
| CID5280343 | PPARD | Hepatotoxic | Known |
| CID5280343 | CAV1 | Hepatotoxic | Known |
| CID5280343 | COL1A1 | Hepatotoxic | Known |
| CID5280343 | CCND1 | Hepatotoxic | Known |
| CID5280343 | IL1A | Hepatotoxic | Known |
| CID5280343 | NOS3 | Hepatotoxic | Known |
| CID5280343 | HSPB1 | Non-hepatotoxic | Known |
| CID5280343 | SERPINE1 | Hepatotoxic | Known |
| CID5280343 | RUNX2 | Non-hepatotoxic | Known |
| CID5280343 | GSK3B | Non-hepatotoxic | Known |
| CID5280343 | COL3A1 | Hepatotoxic | Known |
| CID5280343 | CDKN2A | Hepatotoxic | Known |
| CID5280343 | RASSF1 | Non-hepatotoxic | Known |
| CID5280343 | E2F1 | Hepatotoxic | Known |
| CID5280343 | CTSD | Non-hepatotoxic | Known |
| CID5280343 | THBD | Hepatotoxic | Known |
| CID5280343 | AR | Hepatotoxic | Known |
| CID5280343 | IGFBP3 | Hepatotoxic | Known |
| CID5280343 | IGF2 | Hepatotoxic | Known |
| CID5280343 | SOD3 | Hepatotoxic | Known |
| CID5280343 | CAT | Hepatotoxic | Known |
| CID5280343 | HMOX1 | Hepatotoxic | Known |
| CID5280343 | POR | Non-hepatotoxic | Known |
| CID5280343 | PTEN | Hepatotoxic | Known |
| CID5280343 | PRKCA | Hepatotoxic | Known |
| CID5280343 | ERBB3 | Non-hepatotoxic | Known |
| CID5280343 | PON1 | Hepatotoxic | Known |
| CID5280343 | RAF1 | Hepatotoxic | Known |
| CID5280343 | F3 | Hepatotoxic | Known |
| CID5280343 | EGF | Hepatotoxic | Known |
| CID5280343 | DIO1 | Non-hepatotoxic | Known |
| CID5280343 | ODC1 | Non-hepatotoxic | Known |
| CID5280343 | NQO1 | Hepatotoxic | Known |
| CID5280343 | RASA1 | Non-hepatotoxic | Known |
| CID5280343 | FOS | Non-hepatotoxic | Known |
| CID5280343 | SULT1E1 | Hepatotoxic | Known |
| CID5280343 | GSTM1 | Hepatotoxic | Known |
| CID5280343 | GSTM2 | Non-hepatotoxic | Known |
| CID5280343 | NKX3-1 | Non-hepatotoxic | Known |
| CID5280343 | ABCC1 | Hepatotoxic | Known |
| CID5280343 | AKR1B1 | Non-hepatotoxic | Known |
| CID5280343 | ALOX15 | Non-hepatotoxic | Known |
| CID5280343 | AVPR2 | Non-hepatotoxic | Known |
| CID5280343 | CA14 | Non-hepatotoxic | Known |
| CID5280343 | CA3 | Non-hepatotoxic | Known |
| CID5280343 | CA4 | Non-hepatotoxic | Known |
| CID5280343 | CA5A | Non-hepatotoxic | Known |
| CID5280343 | CA6 | Non-hepatotoxic | Known |
| CID5280343 | CA7 | Non-hepatotoxic | Known |
| CID5280343 | CAMK2B | Non-hepatotoxic | Known |
| CID5280343 | CYP19A1 | Hepatotoxic | Known |
| CID5280343 | CYP1A1 | Hepatotoxic | Known |
| CID5280343 | CYP1A2 | Hepatotoxic | Known |
| CID5280343 | CYP1B1 | Hepatotoxic | Known |
| CID5280343 | GPR35 | Non-hepatotoxic | Known |
| CID5280343 | MAPK10 | Non-hepatotoxic | Known |
| CID5280343 | NEK6 | Non-hepatotoxic | Known |
| CID5280343 | NOX4 | Non-hepatotoxic | Known |
| CID5280343 | NUAK1 | Non-hepatotoxic | Known |
| CID5280343 | PKN1 | Non-hepatotoxic | Known |
| CID5280343 | PLA2G1B | Non-hepatotoxic | Known |
| CID5280343 | ATP5B | Non-hepatotoxic | Known |
| CID5280343 | DECR1 | Non-hepatotoxic | Known |
| CID5280343 | ERN1 | Non-hepatotoxic | Known |
| CID5280343 | ERN2 | Non-hepatotoxic | Known |
| CID5280343 | HIBCH | Non-hepatotoxic | Known |
| CID5280343 | RHOA | Non-hepatotoxic | Known |
| CID5280343 | SLC16A1 | Hepatotoxic | Known |
| CID5280343 | SLC16A7 | Non-hepatotoxic | Known |
| CID5280343 | UGT1A10 | Hepatotoxic | Known |
| CID5280343 | UGT1A7 | Hepatotoxic | Known |
| CID5280343 | UGT1A8 | Hepatotoxic | Known |
| CID5280343 | UGT3A1 | Non-hepatotoxic | Known |
| CID5280343 | gyrB | Non-hepatotoxic | Known |
| CID5280343 | HSF1 | Non-hepatotoxic | Known |
| CID5280343 | PRXC1A | Non-hepatotoxic | Known |
| CID5280343 | CXCL11 | Non-hepatotoxic | Known |
| CID5280343 | DCAF5 | Non-hepatotoxic | Known |
| CID5280343 | HSPA5 | Hepatotoxic | Known |
| CID5280343 | CXCL8 | Non-hepatotoxic | Known |
| CID5280343 | CLDN4 | Non-hepatotoxic | Known |
| CID5280343 | CYP3A4 | Hepatotoxic | Known |
| CID5280343 | DUOX2 | Non-hepatotoxic | Known |
| CID5280343 | EIF6 | Non-hepatotoxic | Known |
| CID5280343 | AHSA1 | Non-hepatotoxic | Known |
| CID5280343 | BMP2 | Non-hepatotoxic | Known |
| CID5280343 | RUNX1T1 | Non-hepatotoxic | Known |
| CID5280343 | HERC5 | Non-hepatotoxic | Known |
| CID5280343 | E2F2 | Non-hepatotoxic | Known |
| CID5280343 | MGAM | Non-hepatotoxic | Known |
| CID5280343 | PSMD3 | Non-hepatotoxic | Known |
| CID5280343 | ELK1 | Non-hepatotoxic | Known |
| CID5280343 | PCOLCE | Non-hepatotoxic | Known |
| CID5280343 | NPEPPS | Non-hepatotoxic | Known |
| CID5280343 | HK2 | Non-hepatotoxic | Known |
| CID5280343 | HAS2 | Non-hepatotoxic | Known |
| CID5280343 | KDM4E | Non-hepatotoxic | Known |
| CID5280343 | POLB | Non-hepatotoxic | Known |
| CID5280343 | MAPT | Non-hepatotoxic | Known |
| CID5280343 | CYP2C9 | Hepatotoxic | Known |
| CID5280343 | HPGD | Non-hepatotoxic | Known |
| CID5280343 | RECQL | Non-hepatotoxic | Known |
| CID5280343 | NPSR1 | Non-hepatotoxic | Known |
| CID5280343 | HSD17B10 | Non-hepatotoxic | Known |
| CID5280343 | PYGL | Non-hepatotoxic | Known |
| CID5280343 | MMP3 | Hepatotoxic | Known |
| CID5280343 | CYP2C8 | Hepatotoxic | Known |
| CID5280343 | SRC | Non-hepatotoxic | Known |
| CID5280343 | BACE1 | Non-hepatotoxic | Known |
| CID5280343 | CA5B | Non-hepatotoxic | Predicted |
| CID5280343 | HDAC10 | Hepatotoxic | Predicted |
| CID5280343 | TDP1 | Non-hepatotoxic | Predicted |
| CID5280343 | CYP2C19 | Hepatotoxic | Predicted |
| CID5280343 | MMP12 | Hepatotoxic | Predicted |
| CID5280343 | MMP1 | Hepatotoxic | Known |
| CID5280343 | CA13 | Non-hepatotoxic | Predicted |
| CID5280343 | CDK5 | Non-hepatotoxic | Predicted |
| CID5280343 | ALDH1A1 | Non-hepatotoxic | Predicted |
| CID5280343 | LMNA | Non-hepatotoxic | Predicted |
| CID5280343 | APEX1 | Non-hepatotoxic | Predicted |
| CID5280343 | HDAC3 | Non-hepatotoxic | Predicted |
| CID5280343 | HDAC1 | Hepatotoxic | Predicted |
| CID5280343 | PLK4 | Non-hepatotoxic | Predicted |
| CID5280343 | AGL | Non-hepatotoxic | Predicted |
| CID5280343 | MMP8 | Hepatotoxic | Predicted |
| CID5280343 | ERBB2 | Hepatotoxic | Known |
| CID5280343 | HDAC8 | Non-hepatotoxic | Predicted |
| CID5280343 | THPO | Non-hepatotoxic | Predicted |
| CID5280343 | HDAC2 | Non-hepatotoxic | Predicted |
| CID5280378 | JUN | Hepatotoxic | Known |
| CID5280378 | IL4 | Hepatotoxic | Known |
| CID5280378 | ESR1 | Hepatotoxic | Known |
| CID5280378 | PPARG | Hepatotoxic | Known |
| CID5280378 | TP53 | Hepatotoxic | Known |
| CID5280378 | IL2 | Hepatotoxic | Known |
| CID5280378 | ESR2 | Hepatotoxic | Known |
| CID5280378 | HSD3B2 | Non-hepatotoxic | Known |
| CID5280378 | HSD3B1 | Non-hepatotoxic | Known |
| CID5280378 | MT-ND6 | Non-hepatotoxic | Known |
| CID5280378 | ATP5B | Non-hepatotoxic | Known |
| CID5280378 | SIRT1 | Non-hepatotoxic | Known |
| CID5280378 | CYP2D6 | Hepatotoxic | Known |
| CID5280378 | MAPT | Non-hepatotoxic | Known |
| CID5280378 | CYP2C9 | Hepatotoxic | Known |
| CID5280378 | CYP2C19 | Hepatotoxic | Known |
| CID5280378 | CYP3A4 | Hepatotoxic | Predicted |
| CID5280378 | TNF | Hepatotoxic | Predicted |
| CID5280378 | ALDH2 | Hepatotoxic | Predicted |
| CID5280378 | NPC1 | Non-hepatotoxic | Predicted |
| CID5280378 | RAB9A | Non-hepatotoxic | Predicted |
| CID5280378 | HIF1A | Hepatotoxic | Predicted |
| CID5280378 | LMNA | Non-hepatotoxic | Predicted |
| CID5280378 | TSHR | Non-hepatotoxic | Predicted |
| CID5280378 | HSD17B10 | Non-hepatotoxic | Predicted |
| CID5280378 | ALDH1A1 | Non-hepatotoxic | Predicted |
| CID5280378 | HPGD | Non-hepatotoxic | Predicted |
| CID5280378 | ALOX15 | Non-hepatotoxic | Predicted |
| CID5280378 | SMN1 | Non-hepatotoxic | Predicted |
| CID5280378 | KMT2A | Non-hepatotoxic | Predicted |
| CID5280378 | TDP1 | Non-hepatotoxic | Predicted |
| CID5280378 | NFKB1 | Hepatotoxic | Predicted |
| CID5280378 | THPO | Non-hepatotoxic | Predicted |
| CID5280378 | KDM4E | Non-hepatotoxic | Predicted |
| CID5280378 | MAPK1 | Non-hepatotoxic | Predicted |
| CID5280378 | MAPK10 | Non-hepatotoxic | Predicted |
| CID5280442 | RELA | Hepatotoxic | Known |
| CID5280442 | PTGS2 | Hepatotoxic | Known |
| CID5280442 | FASLG | Hepatotoxic | Known |
| CID5280442 | BCL2 | Non-hepatotoxic | Known |
| CID5280442 | CASP3 | Hepatotoxic | Known |
| CID5280442 | CASP8 | Hepatotoxic | Known |
| CID5280442 | NR3C1 | Hepatotoxic | Known |
| CID5280442 | HIF1A | Hepatotoxic | Known |
| CID5280442 | NOS2 | Hepatotoxic | Known |
| CID5280442 | FASN | Non-hepatotoxic | Known |
| CID5280442 | BAX | Hepatotoxic | Known |
| CID5280442 | CDKN1A | Non-hepatotoxic | Known |
| CID5280442 | CYP1A1 | Hepatotoxic | Known |
| CID5280442 | CYP1A2 | Hepatotoxic | Known |
| CID5280442 | CYP1B1 | Hepatotoxic | Known |
| CID5280442 | MAPK10 | Non-hepatotoxic | Known |
| CID5280442 | NPC1 | Non-hepatotoxic | Known |
| CID5280442 | ALDH1A1 | Non-hepatotoxic | Known |
| CID5280442 | CYP3A4 | Hepatotoxic | Known |
| CID5280442 | AR | Hepatotoxic | Known |
| CID5280442 | MAPT | Non-hepatotoxic | Known |
| CID5280442 | CYP2C9 | Hepatotoxic | Known |
| CID5280442 | CYP2C19 | Hepatotoxic | Known |
| CID5280442 | RAB9A | Non-hepatotoxic | Known |
| CID5280442 | ALDH2 | Hepatotoxic | Predicted |
| CID5280442 | CALM1 | Non-hepatotoxic | Predicted |
| CID5280442 | TP53 | Hepatotoxic | Known |
| CID5280442 | CYP2D6 | Hepatotoxic | Predicted |
| CID5280442 | XDH | Hepatotoxic | Predicted |
| CID5280442 | ABCG2 | Hepatotoxic | Predicted |
| CID5280442 | LMNA | Non-hepatotoxic | Predicted |
| CID5280442 | HPGD | Non-hepatotoxic | Predicted |
| CID5280442 | HSD17B10 | Non-hepatotoxic | Predicted |
| CID5280442 | KMT2A | Non-hepatotoxic | Predicted |
| CID5280442 | ABCB1 | Hepatotoxic | Predicted |
| CID5280442 | CYP19A1 | Hepatotoxic | Known |
| CID5280442 | HSD17B1 | Non-hepatotoxic | Predicted |
| CID5280442 | ALOX15 | Non-hepatotoxic | Predicted |
| CID5280442 | ABCC1 | Hepatotoxic | Predicted |
| CID5280442 | NOX4 | Non-hepatotoxic | Predicted |
| CID5280442 | SMN1 | Non-hepatotoxic | Predicted |
| CID5280442 | KDM4E | Non-hepatotoxic | Predicted |
| CID5280442 | PLG | Hepatotoxic | Predicted |
| CID5280442 | TSHR | Non-hepatotoxic | Predicted |
| CID5280443 | IL4 | Hepatotoxic | Known |
| CID5280443 | ICAM1 | Hepatotoxic | Known |
| CID5280443 | JUN | Hepatotoxic | Known |
| CID5280443 | IFNG | Hepatotoxic | Known |
| CID5280443 | ABCB1 | Hepatotoxic | Known |
| CID5280443 | CDK5 | Non-hepatotoxic | Known |
| CID5280443 | ESR1 | Hepatotoxic | Known |
| CID5280443 | PTGS2 | Hepatotoxic | Known |
| CID5280443 | RELA | Hepatotoxic | Known |
| CID5280443 | XIAP | Non-hepatotoxic | Known |
| CID5280443 | NFKBIA | Hepatotoxic | Known |
| CID5280443 | HIF1A | Hepatotoxic | Known |
| CID5280443 | VEGFA | Hepatotoxic | Known |
| CID5280443 | TP53 | Hepatotoxic | Known |
| CID5280443 | BCL2 | Non-hepatotoxic | Known |
| CID5280443 | INS | Hepatotoxic | Known |
| CID5280443 | IL13 | Hepatotoxic | Known |
| CID5280443 | RB1 | Hepatotoxic | Known |
| CID5280443 | CASP3 | Hepatotoxic | Known |
| CID5280443 | MMP9 | Hepatotoxic | Known |
| CID5280443 | TNF | Hepatotoxic | Known |
| CID5280443 | IL2 | Hepatotoxic | Known |
| CID5280443 | TNKS2 | Non-hepatotoxic | Known |
| CID5280443 | CD40LG | Non-hepatotoxic | Known |
| CID5280443 | PSME3 | Non-hepatotoxic | Known |
| CID5280443 | IKBKG | Hepatotoxic | Known |
| CID5280443 | NR3C1 | Hepatotoxic | Known |
| CID5280443 | TSHR | Non-hepatotoxic | Known |
| CID5280443 | KMT2A | Non-hepatotoxic | Known |
| CID5280443 | SYK | Non-hepatotoxic | Known |
| CID5280443 | ABCG2 | Hepatotoxic | Known |
| CID5280443 | AR | Hepatotoxic | Known |
| CID5280443 | CDK6 | Non-hepatotoxic | Known |
| CID5280443 | CSNK2A1 | Non-hepatotoxic | Known |
| CID5280443 | ESR2 | Hepatotoxic | Known |
| CID5280443 | FLT3 | Hepatotoxic | Known |
| CID5280443 | HSD17B1 | Non-hepatotoxic | Known |
| CID5280443 | MAOA | Non-hepatotoxic | Known |
| CID5280443 | PIM1 | Non-hepatotoxic | Known |
| CID5280443 | TNKS | Non-hepatotoxic | Known |
| CID5280443 | XDH | Hepatotoxic | Known |
| CID5280443 | BCL2L1 | Hepatotoxic | Known |
| CID5280443 | MCL1 | Hepatotoxic | Known |
| CID5280443 | AKT1 | Hepatotoxic | Known |
| CID5280443 | BAD | Non-hepatotoxic | Known |
| CID5280443 | IGF1R | Hepatotoxic | Known |
| CID5280443 | CASP9 | Hepatotoxic | Known |
| CID5280443 | GSK3B | Non-hepatotoxic | Known |
| CID5280443 | NOS2 | Hepatotoxic | Known |
| CID5280443 | CDKN2A | Hepatotoxic | Known |
| CID5280443 | CDK1 | Hepatotoxic | Known |
| CID5280443 | CCNA2 | Non-hepatotoxic | Known |
| CID5280443 | CCNB1 | Non-hepatotoxic | Known |
| CID5280443 | CDKN1A | Non-hepatotoxic | Known |
| CID5280443 | BAX | Hepatotoxic | Known |
| CID5280443 | MDM2 | Hepatotoxic | Known |
| CID5280443 | APC | Hepatotoxic | Known |
| CID5280443 | HMOX1 | Hepatotoxic | Known |
| CID5280443 | FCER2 | Non-hepatotoxic | Known |
| CID5280443 | ACACA | Hepatotoxic | Known |
| CID5280443 | AKR1C3 | Non-hepatotoxic | Known |
| CID5280443 | CCND2 | Non-hepatotoxic | Known |
| CID5280443 | CDK2 | Non-hepatotoxic | Known |
| CID5280443 | CDK4 | Non-hepatotoxic | Known |
| CID5280443 | ODC1 | Non-hepatotoxic | Known |
| CID5280443 | SLC5A5 | Non-hepatotoxic | Known |
| CID5280443 | PLAU | Hepatotoxic | Known |
| CID5280443 | SERPINE1 | Hepatotoxic | Known |
| CID5280443 | FOS | Non-hepatotoxic | Known |
| CID5280443 | MMP1 | Hepatotoxic | Known |
| CID5280443 | INSR | Hepatotoxic | Known |
| CID5280443 | SLC2A4 | Hepatotoxic | Known |
| CID5280443 | ABCC1 | Hepatotoxic | Known |
| CID5280443 | CYP19A1 | Hepatotoxic | Known |
| CID5280443 | CYP1A1 | Hepatotoxic | Known |
| CID5280443 | CYP1A2 | Hepatotoxic | Known |
| CID5280443 | CYP1B1 | Hepatotoxic | Known |
| CID5280443 | KARS | Non-hepatotoxic | Known |
| CID5280443 | MAPK10 | Non-hepatotoxic | Known |
| CID5280443 | NOX4 | Non-hepatotoxic | Known |
| CID5280443 | ALG5 | Non-hepatotoxic | Known |
| CID5280443 | CFLAR | Non-hepatotoxic | Known |
| CID5280443 | ALPI | Non-hepatotoxic | Known |
| CID5280443 | EIF6 | Non-hepatotoxic | Known |
| CID5280443 | RUNX1T1 | Non-hepatotoxic | Known |
| CID5280443 | HERC5 | Non-hepatotoxic | Known |
| CID5280443 | CYCS | Non-hepatotoxic | Known |
| CID5280443 | AHSA1 | Non-hepatotoxic | Known |
| CID5280443 | G6PC | Non-hepatotoxic | Known |
| CID5280443 | MS4A2 | Hepatotoxic | Known |
| CID5280443 | TRPM2 | Non-hepatotoxic | Known |
| CID5280443 | EEF1E1 | Non-hepatotoxic | Known |
| CID5280443 | PSMD3 | Non-hepatotoxic | Known |
| CID5280443 | FXYD2 | Non-hepatotoxic | Known |
| CID5280443 | AAGAB | Non-hepatotoxic | Known |
| CID5280443 | NPC1 | Non-hepatotoxic | Known |
| CID5280443 | USP2 | Non-hepatotoxic | Known |
| CID5280443 | ALDH1A1 | Non-hepatotoxic | Known |
| CID5280443 | CYP3A4 | Hepatotoxic | Known |
| CID5280443 | MAPT | Non-hepatotoxic | Known |
| CID5280443 | CYP2C9 | Hepatotoxic | Known |
| CID5280443 | PKM | Non-hepatotoxic | Known |
| CID5280443 | HPGD | Non-hepatotoxic | Known |
| CID5280443 | ALOX15 | Non-hepatotoxic | Known |
| CID5280443 | CYP2C19 | Hepatotoxic | Known |
| CID5280443 | OPRM1 | Hepatotoxic | Known |
| CID5280443 | THPO | Non-hepatotoxic | Known |
| CID5280443 | OPRD1 | Non-hepatotoxic | Known |
| CID5280443 | OPRK1 | Non-hepatotoxic | Known |
| CID5280443 | HSD17B10 | Non-hepatotoxic | Known |
| CID5280443 | TDP1 | Non-hepatotoxic | Known |
| CID5280443 | CFTR | Hepatotoxic | Known |
| CID5280443 | ACHE | Non-hepatotoxic | Known |
| CID5280443 | CSNK2B | Non-hepatotoxic | Known |
| CID5280443 | PARP1 | Hepatotoxic | Predicted |
| CID5280443 | PTP4A3 | Non-hepatotoxic | Predicted |
| CID5280443 | RAB9A | Non-hepatotoxic | Predicted |
| CID5280443 | LMNA | Non-hepatotoxic | Predicted |
| CID5280443 | KDM4E | Non-hepatotoxic | Predicted |
| CID5280443 | ALOX12 | Non-hepatotoxic | Predicted |
| CID5280443 | RECQL | Non-hepatotoxic | Predicted |
| CID5280443 | LCK | Non-hepatotoxic | Predicted |
| CID5280443 | MAPK1 | Non-hepatotoxic | Predicted |
| CID5280443 | CBR1 | Non-hepatotoxic | Predicted |
| CID5280443 | ALOX5 | Non-hepatotoxic | Predicted |
| CID5280443 | MAPK14 | Hepatotoxic | Predicted |
| CID5280443 | PLG | Hepatotoxic | Predicted |
| CID5280443 | CCND1 | Hepatotoxic | Known |
| CID5280443 | POLB | Non-hepatotoxic | Predicted |
| CID5280443 | SMN1 | Non-hepatotoxic | Predicted |
| CID5280443 | CYP2D6 | Hepatotoxic | Predicted |
| CID5280443 | BLM | Non-hepatotoxic | Predicted |
| CID5280443 | CDK5R1 | Non-hepatotoxic | Predicted |
| CID5280443 | NFKB1 | Hepatotoxic | Predicted |
| CID5280961 | ICAM1 | Hepatotoxic | Known |
| CID5280961 | JUN | Hepatotoxic | Known |
| CID5280961 | BIRC5 | Non-hepatotoxic | Known |
| CID5280961 | EGFR | Hepatotoxic | Known |
| CID5280961 | ESR1 | Hepatotoxic | Known |
| CID5280961 | VEGFA | Hepatotoxic | Known |
| CID5280961 | STAT1 | Hepatotoxic | Known |
| CID5280961 | RELA | Hepatotoxic | Known |
| CID5280961 | ATM | Hepatotoxic | Known |
| CID5280961 | PPARG | Hepatotoxic | Known |
| CID5280961 | LDLR | Hepatotoxic | Known |
| CID5280961 | TNF | Hepatotoxic | Known |
| CID5280961 | IL1B | Hepatotoxic | Known |
| CID5280961 | CCL2 | Hepatotoxic | Known |
| CID5280961 | PTGS2 | Hepatotoxic | Known |
| CID5280961 | INS | Hepatotoxic | Known |
| CID5280961 | TP53 | Hepatotoxic | Known |
| CID5280961 | CDC20 | Non-hepatotoxic | Known |
| CID5280961 | FN1 | Hepatotoxic | Known |
| CID5280961 | BCL2 | Non-hepatotoxic | Known |
| CID5280961 | CASP3 | Hepatotoxic | Known |
| CID5280961 | TGFB1 | Hepatotoxic | Known |
| CID5280961 | HIF1A | Hepatotoxic | Known |
| CID5280961 | STAT3 | Hepatotoxic | Known |
| CID5280961 | ERBB2 | Hepatotoxic | Known |
| CID5280961 | PML | Non-hepatotoxic | Known |
| CID5280961 | MMP9 | Hepatotoxic | Known |
| CID5280961 | BTK | Non-hepatotoxic | Known |
| CID5280961 | SYK | Non-hepatotoxic | Known |
| CID5280961 | CRYAB | Non-hepatotoxic | Known |
| CID5280961 | MDC1 | Non-hepatotoxic | Known |
| CID5280961 | SAFB | Non-hepatotoxic | Known |
| CID5280961 | GFAP | Non-hepatotoxic | Known |
| CID5280961 | NR3C1 | Hepatotoxic | Known |
| CID5280961 | TSHR | Non-hepatotoxic | Known |
| CID5280961 | BLM | Non-hepatotoxic | Known |
| CID5280961 | ABCG2 | Hepatotoxic | Known |
| CID5280961 | ABL1 | Hepatotoxic | Known |
| CID5280961 | ESR2 | Hepatotoxic | Known |
| CID5280961 | ESRRA | Non-hepatotoxic | Known |
| CID5280961 | ESRRG | Non-hepatotoxic | Known |
| CID5280961 | HSD17B1 | Non-hepatotoxic | Known |
| CID5280961 | SLCO1B1 | Hepatotoxic | Known |
| CID5280961 | SLCO1B3 | Hepatotoxic | Known |
| CID5280961 | CFTR | Hepatotoxic | Known |
| CID5280961 | PTEN | Hepatotoxic | Known |
| CID5280961 | NOS2 | Hepatotoxic | Known |
| CID5280961 | UGT1A1 | Hepatotoxic | Known |
| CID5280961 | CDK1 | Hepatotoxic | Known |
| CID5280961 | RAF1 | Hepatotoxic | Known |
| CID5280961 | CDKN1A | Non-hepatotoxic | Known |
| CID5280961 | CCNB1 | Non-hepatotoxic | Known |
| CID5280961 | CHEK2 | Non-hepatotoxic | Known |
| CID5280961 | PPARA | Hepatotoxic | Known |
| CID5280961 | HMGCR | Hepatotoxic | Known |
| CID5280961 | VCAM1 | Non-hepatotoxic | Known |
| CID5280961 | CCNA2 | Non-hepatotoxic | Known |
| CID5280961 | AKT1 | Hepatotoxic | Known |
| CID5280961 | AR | Hepatotoxic | Known |
| CID5280961 | KLK3 | Non-hepatotoxic | Known |
| CID5280961 | CDC25C | Non-hepatotoxic | Known |
| CID5280961 | FOS | Non-hepatotoxic | Known |
| CID5280961 | HDAC6 | Non-hepatotoxic | Known |
| CID5280961 | BAX | Hepatotoxic | Known |
| CID5280961 | APOA1 | Hepatotoxic | Known |
| CID5280961 | CDKN2A | Hepatotoxic | Known |
| CID5280961 | SOD2 | Hepatotoxic | Known |
| CID5280961 | NOS3 | Hepatotoxic | Known |
| CID5280961 | PLK1 | Non-hepatotoxic | Known |
| CID5280961 | TEP1 | Non-hepatotoxic | Known |
| CID5280961 | CASP9 | Hepatotoxic | Known |
| CID5280961 | KCNJ11 | Non-hepatotoxic | Known |
| CID5280961 | PTGER2 | Non-hepatotoxic | Known |
| CID5280961 | MDM2 | Hepatotoxic | Known |
| CID5280961 | IGF1R | Hepatotoxic | Known |
| CID5280961 | MAPK3 | Non-hepatotoxic | Known |
| CID5280961 | MAPK1 | Non-hepatotoxic | Known |
| CID5280961 | SELE | Hepatotoxic | Known |
| CID5280961 | AOX1 | Non-hepatotoxic | Known |
| CID5280961 | MGAM | Non-hepatotoxic | Known |
| CID5280961 | TTR | Non-hepatotoxic | Known |
| CID5280961 | PCOLCE | Non-hepatotoxic | Known |
| CID5280961 | CXCL8 | Non-hepatotoxic | Known |
| CID5280961 | AHSA1 | Non-hepatotoxic | Known |
| CID5280961 | PBK | Non-hepatotoxic | Known |
| CID5280961 | BUB1 | Non-hepatotoxic | Known |
| CID5280961 | AKR1B1 | Non-hepatotoxic | Known |
| CID5280961 | TGFB2 | Non-hepatotoxic | Known |
| CID5280961 | RASGRF2 | Non-hepatotoxic | Known |
| CID5280961 | CDC37 | Non-hepatotoxic | Known |
| CID5280961 | MAP2K5 | Non-hepatotoxic | Known |
| CID5280961 | MAPK12 | Non-hepatotoxic | Known |
| CID5280961 | CCNB1IP1 | Non-hepatotoxic | Known |
| CID5280961 | PLK2 | Non-hepatotoxic | Known |
| CID5280961 | NCOR1 | Non-hepatotoxic | Known |
| CID5280961 | GCK | Hepatotoxic | Known |
| CID5280961 | CYP24A1 | Hepatotoxic | Known |
| CID5280961 | PTGFR | Non-hepatotoxic | Known |
| CID5280961 | GLB1 | Non-hepatotoxic | Known |
| CID5280961 | MSLN | Non-hepatotoxic | Known |
| CID5280961 | TIMP1 | Non-hepatotoxic | Known |
| CID5280961 | NPC1 | Non-hepatotoxic | Known |
| CID5280961 | ESRRB | Non-hepatotoxic | Known |
| CID5280961 | CYP3A4 | Hepatotoxic | Known |
| CID5280961 | MAPT | Non-hepatotoxic | Known |
| CID5280961 | CYP2C9 | Hepatotoxic | Known |
| CID5280961 | TBXAS1 | Non-hepatotoxic | Known |
| CID5280961 | CYP2C19 | Hepatotoxic | Known |
| CID5280961 | THPO | Non-hepatotoxic | Known |
| CID5280961 | RAB9A | Non-hepatotoxic | Known |
| CID5280961 | TDP1 | Non-hepatotoxic | Known |
| CID5280961 | LMNA | Non-hepatotoxic | Predicted |
| CID5280961 | SULT1A1 | Hepatotoxic | Predicted |
| CID5280961 | SULT1E1 | Hepatotoxic | Known |
| CID5280961 | GPER1 | Non-hepatotoxic | Predicted |
| CID5280961 | BCR | Non-hepatotoxic | Predicted |
| CID5280961 | CYP1A2 | Hepatotoxic | Predicted |
| CID5280961 | HSD17B10 | Non-hepatotoxic | Predicted |
| CID5280961 | CYP1A1 | Hepatotoxic | Predicted |
| CID5280961 | SLC22A1 | Hepatotoxic | Predicted |
| CID5280961 | ALDH2 | Hepatotoxic | Predicted |
| CID5280961 | CYP2B6 | Hepatotoxic | Predicted |
| CID5280961 | CYP2D6 | Hepatotoxic | Predicted |
| CID5280961 | HPGD | Non-hepatotoxic | Known |
| CID5280961 | SLC22A3 | Hepatotoxic | Predicted |
| CID5280961 | CYP1B1 | Hepatotoxic | Predicted |
| CID5280961 | NFKB1 | Hepatotoxic | Predicted |
| CID5280961 | SMN1 | Non-hepatotoxic | Predicted |
| CID5280961 | ALOX15 | Non-hepatotoxic | Predicted |
| CID5280961 | ALDH1A1 | Non-hepatotoxic | Predicted |
| CID5280961 | KMT2A | Non-hepatotoxic | Predicted |
| CID5281051 | RELA | Hepatotoxic | Known |
| CID5281051 | NFKB1 | Hepatotoxic | Known |
| CID5281051 | NFKBIA | Hepatotoxic | Known |
| CID5281051 | CRHR1 | Non-hepatotoxic | Known |
| CID5281051 | KMT2A | Non-hepatotoxic | Known |
| CID5281051 | DRD3 | Non-hepatotoxic | Known |
| CID5281051 | GSR | Non-hepatotoxic | Known |
| CID5281051 | MMP1 | Hepatotoxic | Known |
| CID5281051 | CYP3A4 | Hepatotoxic | Known |
| CID5281051 | CRTC3 | Non-hepatotoxic | Known |
| CID5281051 | THRB | Non-hepatotoxic | Known |
| CID5281051 | HPGD | Non-hepatotoxic | Known |
| CID5281051 | RECQL | Non-hepatotoxic | Known |
| CID5281051 | MAPT | Non-hepatotoxic | Predicted |
| CID5281051 | DRD2 | Hepatotoxic | Predicted |
| CID5281051 | HSD17B10 | Non-hepatotoxic | Predicted |
| CID5281051 | KDM4E | Non-hepatotoxic | Predicted |
| CID5281051 | ALOX15 | Non-hepatotoxic | Predicted |
| CID5281051 | ALDH1A1 | Non-hepatotoxic | Predicted |
| CID5281051 | TDP1 | Non-hepatotoxic | Predicted |
| CID5281051 | BLM | Non-hepatotoxic | Predicted |
| CID5281051 | TP53 | Hepatotoxic | Predicted |
| CID5281051 | DRD4 | Non-hepatotoxic | Predicted |
| CID5281051 | APEX1 | Non-hepatotoxic | Predicted |
| CID5281051 | LMNA | Non-hepatotoxic | Predicted |
| CID5281051 | CYP2C19 | Hepatotoxic | Predicted |
| CID5281051 | DRD5 | Non-hepatotoxic | Predicted |
| CID5281051 | MAPK1 | Non-hepatotoxic | Predicted |
| CID5281051 | CYP2D6 | Hepatotoxic | Predicted |
| CID5281051 | SMN1 | Non-hepatotoxic | Predicted |
| CID5281051 | CYP2C9 | Hepatotoxic | Predicted |
| CID5281051 | HIF1A | Hepatotoxic | Predicted |
| CID5281051 | USP2 | Non-hepatotoxic | Predicted |
| CID5281426 | CA12 | Non-hepatotoxic | Known |
| CID5281426 | CA9 | Non-hepatotoxic | Known |
| CID5281426 | CA7 | Non-hepatotoxic | Predicted |
| CID5281426 | CA1 | Non-hepatotoxic | Predicted |
| CID5281426 | CA14 | Non-hepatotoxic | Predicted |
| CID5281426 | CA2 | Non-hepatotoxic | Predicted |
| CID5281426 | CA13 | Non-hepatotoxic | Predicted |
| CID5281426 | CA3 | Non-hepatotoxic | Predicted |
| CID5281426 | CA4 | Non-hepatotoxic | Predicted |
| CID5281426 | CA6 | Non-hepatotoxic | Predicted |
| CID5281426 | CA5A | Non-hepatotoxic | Predicted |
| CID5281426 | CA5B | Non-hepatotoxic | Predicted |
| CID5281426 | HSD17B3 | Non-hepatotoxic | Predicted |
| CID5281426 | GLA | Non-hepatotoxic | Predicted |
| CID5281426 | HSD17B10 | Non-hepatotoxic | Predicted |
| CID5281426 | LMNA | Non-hepatotoxic | Predicted |
| CID5281426 | AR | Hepatotoxic | Predicted |
| CID5281426 | ABCB1 | Hepatotoxic | Predicted |
| CID5281426 | KMT2A | Non-hepatotoxic | Predicted |
| CID5281426 | KDM4E | Non-hepatotoxic | Predicted |
| CID5281426 | ESR2 | Hepatotoxic | Predicted |
| CID5281426 | HPGD | Non-hepatotoxic | Predicted |
| CID5281702 | PTGS2 | Hepatotoxic | Known |
| CID5281702 | GSTP1 | Hepatotoxic | Known |
| CID5281702 | SULT1E1 | Hepatotoxic | Known |
| CID5281702 | PTGS1 | Non-hepatotoxic | Known |
| CID5281702 | AKR1B1 | Non-hepatotoxic | Known |
| CID5281702 | AKR1B10 | Non-hepatotoxic | Predicted |
| CID5281702 | GBA | Non-hepatotoxic | Predicted |
| CID5281702 | RECQL | Non-hepatotoxic | Predicted |
| CID5281702 | ALOX12 | Non-hepatotoxic | Predicted |
| CID5281702 | MAPT | Non-hepatotoxic | Predicted |
| CID5281702 | APAF1 | Non-hepatotoxic | Predicted |
| CID5281702 | GAA | Non-hepatotoxic | Predicted |
| CID5281702 | ALPI | Non-hepatotoxic | Predicted |
| CID5281702 | FYN | Non-hepatotoxic | Predicted |
| CID5281702 | ALOX15 | Non-hepatotoxic | Predicted |
| CID5281702 | POLB | Non-hepatotoxic | Predicted |
| CID5281702 | TTR | Non-hepatotoxic | Predicted |
| CID5281702 | AKR1C2 | Hepatotoxic | Predicted |
| CID5281702 | AKR1C4 | Non-hepatotoxic | Predicted |
| CID5281702 | AKR1A1 | Non-hepatotoxic | Predicted |
| CID5281702 | CYP2C9 | Hepatotoxic | Predicted |
| CID5281702 | PTPN6 | Non-hepatotoxic | Predicted |
| CID5281702 | CYP2C19 | Hepatotoxic | Predicted |
| CID5281702 | PTPN11 | Non-hepatotoxic | Predicted |
| CID5281702 | OPRD1 | Non-hepatotoxic | Predicted |
| CID5318214 | PLG | Hepatotoxic | Known |
| CID5318214 | NOX4 | Non-hepatotoxic | Predicted |
| CID5318214 | MAPK10 | Non-hepatotoxic | Predicted |
| CID5318214 | F11 | Non-hepatotoxic | Predicted |
| CID5318214 | MAPT | Non-hepatotoxic | Predicted |
| CID5318214 | CYP3A4 | Hepatotoxic | Predicted |
| CID5318214 | ABCG2 | Hepatotoxic | Predicted |
| CID5318214 | CYP19A1 | Hepatotoxic | Predicted |
| CID5318214 | TDP1 | Non-hepatotoxic | Predicted |
| CID5318214 | ALDH1A1 | Non-hepatotoxic | Predicted |
| CID5318214 | KDM4E | Non-hepatotoxic | Predicted |
| CID5318214 | RAB9A | Non-hepatotoxic | Predicted |
| CID5318214 | LMNA | Non-hepatotoxic | Predicted |
| CID5318214 | ALOX15 | Non-hepatotoxic | Predicted |
| CID5318214 | NPC1 | Non-hepatotoxic | Predicted |
| CID5318214 | TP53 | Hepatotoxic | Predicted |
| CID5318214 | MAOA | Non-hepatotoxic | Predicted |
| CID5318214 | HSD17B10 | Non-hepatotoxic | Predicted |
| CID5318214 | CA9 | Non-hepatotoxic | Predicted |
| CID5318214 | CA12 | Non-hepatotoxic | Predicted |
| CID5318214 | TSHR | Non-hepatotoxic | Predicted |
| CID5319562 | CA12 | Non-hepatotoxic | Known |
| CID5319562 | CA1 | Non-hepatotoxic | Known |
| CID5319562 | CA2 | Non-hepatotoxic | Known |
| CID5319562 | CA9 | Non-hepatotoxic | Known |
| CID5319562 | CA14 | Non-hepatotoxic | Known |
| CID5319562 | CA7 | Non-hepatotoxic | Known |
| CID5319562 | CA13 | Non-hepatotoxic | Predicted |
| CID5319562 | CA6 | Non-hepatotoxic | Predicted |
| CID5319562 | CA4 | Non-hepatotoxic | Predicted |
| CID5319562 | CA5A | Non-hepatotoxic | Predicted |
| CID5319562 | CA5B | Non-hepatotoxic | Predicted |
| CID5319562 | CA3 | Non-hepatotoxic | Predicted |
| CID5319562 | HSD17B10 | Non-hepatotoxic | Predicted |
| CID5319562 | HSD17B3 | Non-hepatotoxic | Predicted |
| CID5319562 | GLA | Non-hepatotoxic | Predicted |
| CID5319562 | MB | Non-hepatotoxic | Predicted |
| CID5319562 | ESR2 | Hepatotoxic | Predicted |
| CID5319562 | LMNA | Non-hepatotoxic | Predicted |
| CID5319562 | HPGD | Non-hepatotoxic | Predicted |
| CID5319562 | GAA | Non-hepatotoxic | Predicted |
| CID5319562 | MAPT | Non-hepatotoxic | Predicted |
| CID5319562 | TDP1 | Non-hepatotoxic | Predicted |
| CID5319562 | SERPINE1 | Hepatotoxic | Predicted |
| CID5319562 | KMT2A | Non-hepatotoxic | Predicted |
| CID5319562 | CYP3A4 | Hepatotoxic | Predicted |
| CID5319562 | KDM4E | Non-hepatotoxic | Predicted |
| CID5352005 | ABCG2 | Hepatotoxic | Known |
| CID5352005 | TNFRSF1A | Hepatotoxic | Predicted |
| CID5352005 | ABCB1 | Hepatotoxic | Predicted |
| CID5352005 | NR3C1 | Hepatotoxic | Predicted |
| CID5352005 | AR | Hepatotoxic | Predicted |
| CID5352005 | CYP3A4 | Hepatotoxic | Predicted |
| CID5352005 | NPC1 | Non-hepatotoxic | Predicted |
| CID5352005 | RAB9A | Non-hepatotoxic | Predicted |
| CID5352005 | PRKDC | Hepatotoxic | Predicted |
| CID5352005 | ESR2 | Hepatotoxic | Predicted |
| CID5352005 | KMT2A | Non-hepatotoxic | Predicted |
| CID5352005 | ESR1 | Hepatotoxic | Predicted |
| CID5352005 | MAPT | Non-hepatotoxic | Predicted |
| CID5352005 | CYP2C19 | Hepatotoxic | Predicted |
| CID5352005 | MAPK1 | Non-hepatotoxic | Predicted |
| CID5352005 | HSD17B1 | Non-hepatotoxic | Predicted |
| CID5352005 | TBXAS1 | Non-hepatotoxic | Predicted |
| CID5352005 | TDP1 | Non-hepatotoxic | Predicted |
| CID5352005 | CYP1A1 | Hepatotoxic | Predicted |
| CID5352005 | PDE5A | Hepatotoxic | Predicted |
| CID5352005 | CYP1B1 | Hepatotoxic | Predicted |
| CID5353955 | SPHK1 | Non-hepatotoxic | Known |
| CID5353955 | SPHK2 | Non-hepatotoxic | Known |
| CID5379096 | CASP3 | Hepatotoxic | Known |
| CID5460221 | FABP3 | Non-hepatotoxic | Known |
| CID5460221 | FABP4 | Non-hepatotoxic | Predicted |
| CID5460221 | FABP2 | Non-hepatotoxic | Predicted |
| CID5460221 | FFAR1 | Non-hepatotoxic | Predicted |
| CID5460221 | FFAR4 | Non-hepatotoxic | Predicted |
| CID5460221 | TLR2 | Hepatotoxic | Predicted |
| CID5460221 | FABP5 | Non-hepatotoxic | Predicted |
| CID5460221 | PPARD | Hepatotoxic | Predicted |
| CID5460221 | PPARA | Hepatotoxic | Predicted |
| CID5460221 | PPARG | Hepatotoxic | Predicted |
| CID5460221 | HPGD | Non-hepatotoxic | Predicted |
| CID5460221 | ALOX15 | Non-hepatotoxic | Predicted |
| CID5460221 | LMNA | Non-hepatotoxic | Predicted |
| CID5460221 | TSHR | Non-hepatotoxic | Predicted |
| CID5460221 | HMGCR | Hepatotoxic | Predicted |
| CID5460221 | MPEG1 | Non-hepatotoxic | Predicted |
| CID5460221 | FAAH | Non-hepatotoxic | Predicted |
| CID5460221 | ALOX5 | Non-hepatotoxic | Predicted |
| CID5460221 | TRPV1 | Non-hepatotoxic | Predicted |
| CID5460221 | MAPT | Non-hepatotoxic | Predicted |
| CID5460221 | PTPN1 | Non-hepatotoxic | Predicted |
| CID5460332 | FABP3 | Non-hepatotoxic | Known |
| CID5460332 | FABP4 | Non-hepatotoxic | Predicted |
| CID5460332 | FABP2 | Non-hepatotoxic | Predicted |
| CID5460332 | FFAR1 | Non-hepatotoxic | Predicted |
| CID5460332 | FFAR4 | Non-hepatotoxic | Predicted |
| CID5460332 | TLR2 | Hepatotoxic | Predicted |
| CID5460332 | FABP5 | Non-hepatotoxic | Predicted |
| CID5460332 | PPARD | Hepatotoxic | Predicted |
| CID5460332 | PPARA | Hepatotoxic | Predicted |
| CID5460332 | PPARG | Hepatotoxic | Predicted |
| CID5460332 | HPGD | Non-hepatotoxic | Predicted |
| CID5460332 | ALOX15 | Non-hepatotoxic | Predicted |
| CID5460332 | LMNA | Non-hepatotoxic | Predicted |
| CID5460332 | TSHR | Non-hepatotoxic | Predicted |
| CID5460332 | HMGCR | Hepatotoxic | Predicted |
| CID5460332 | FAAH | Non-hepatotoxic | Predicted |
| CID5460332 | MPEG1 | Non-hepatotoxic | Predicted |
| CID5460332 | TRPV1 | Non-hepatotoxic | Predicted |
| CID5460332 | MAPT | Non-hepatotoxic | Predicted |
| CID5460332 | PTPN1 | Non-hepatotoxic | Predicted |
| CID5460332 | ALOX5 | Non-hepatotoxic | Predicted |
| CID5469424 | PPARG | Hepatotoxic | Known |
| CID5469424 | ABCB1 | Hepatotoxic | Known |
| CID6371716 | TSHR | Non-hepatotoxic | Known |
| CID6371716 | ALDH1A1 | Non-hepatotoxic | Known |
| CID6371716 | CYP3A4 | Hepatotoxic | Known |
| CID6371716 | ALOX15 | Non-hepatotoxic | Known |
| CID6371716 | HPGD | Non-hepatotoxic | Predicted |
| CID6371716 | MAPT | Non-hepatotoxic | Predicted |
| CID6371716 | HSD17B10 | Non-hepatotoxic | Predicted |
| CID6371716 | LMNA | Non-hepatotoxic | Predicted |
| CID6371716 | TP53 | Hepatotoxic | Predicted |
| CID6371716 | KDM4E | Non-hepatotoxic | Predicted |
| CID6371716 | TDP1 | Non-hepatotoxic | Predicted |
| CID6371716 | ALOX12 | Non-hepatotoxic | Predicted |
| CID6371716 | MAPK1 | Non-hepatotoxic | Predicted |
| CID6371716 | CYP2C19 | Hepatotoxic | Predicted |
| CID6371716 | HIF1A | Hepatotoxic | Predicted |
| CID6371716 | CYP2C9 | Hepatotoxic | Predicted |
| CID6371716 | CYP2D6 | Hepatotoxic | Predicted |
| CID6371716 | RECQL | Non-hepatotoxic | Predicted |
| CID6371716 | TRPA1 | Non-hepatotoxic | Predicted |
| CID6371716 | KMT2A | Non-hepatotoxic | Predicted |
| CID6371716 | THPO | Non-hepatotoxic | Predicted |
| CID6371716 | USP2 | Non-hepatotoxic | Predicted |
| CID6371716 | NFKB1 | Hepatotoxic | Predicted |
| CID6371716 | SMN1 | Non-hepatotoxic | Predicted |
| CID12004528 | KDM4E | Non-hepatotoxic | Known |
| CID12004528 | POLB | Non-hepatotoxic | Known |
| CID12004528 | ALPI | Non-hepatotoxic | Known |
| CID12004528 | GAA | Non-hepatotoxic | Known |
| CID14137704 | MAPT | Non-hepatotoxic | Known |
| CID14137704 | NPSR1 | Non-hepatotoxic | Known |
| CID14704104 | NPC1 | Non-hepatotoxic | Known |
| CID14704104 | MAPT | Non-hepatotoxic | Known |
| CID14704104 | RAB9A | Non-hepatotoxic | Known |
| CID14704104 | SMN1 | Non-hepatotoxic | Known |
| CID14704104 | TP53 | Hepatotoxic | Predicted |
| CID14704104 | LMNA | Non-hepatotoxic | Predicted |
| CID14704104 | CYP3A4 | Hepatotoxic | Predicted |
| CID14704104 | KMT2A | Non-hepatotoxic | Predicted |
| CID14704104 | RELA | Hepatotoxic | Predicted |
| CID14704104 | KDM4E | Non-hepatotoxic | Predicted |
| CID14704104 | ALDH1A1 | Non-hepatotoxic | Predicted |
| CID14704104 | NFKB1 | Hepatotoxic | Predicted |
| CID14704104 | RECQL | Non-hepatotoxic | Predicted |
| CID14704104 | MITF | Non-hepatotoxic | Predicted |
| CID14704104 | XBP1 | Non-hepatotoxic | Predicted |
| CID14704104 | MAPK1 | Non-hepatotoxic | Predicted |
| CID14704104 | CYP2C19 | Hepatotoxic | Predicted |
| CID14704104 | TNKS | Non-hepatotoxic | Predicted |
| CID14704104 | HPGD | Non-hepatotoxic | Predicted |
| CID14704104 | CYP2C9 | Hepatotoxic | Predicted |
| CID14704104 | NPSR1 | Non-hepatotoxic | Predicted |
| CID14704104 | HTT | Non-hepatotoxic | Predicted |
| CID14704104 | ABCG2 | Hepatotoxic | Predicted |
| CID14704104 | HSD17B10 | Non-hepatotoxic | Predicted |
| CID54675830 | CA1 | Non-hepatotoxic | Known |
| CID54675830 | CA2 | Non-hepatotoxic | Known |

**Table S6.** Detail information for the 6 hepatotoxic compounds validated by literatures.

| **CID** | **Name** | **PMID** | **Summaried** **effect** |
| --- | --- | --- | --- |
| CID5460332 | Linoleic acid | 28827690;  26240766 | contributed to the pathogenesis of alcohol-induced liver injury. |
| CID1183 | Vanillin | 10478828 | potentiates the hepatotoxic potential of paracetamol and carbon tetrachloride (CCl4). |
| CID31404 | 2,6-Di-tert-butyl-4-methylphenol | 3798451 | the hepatotoxic effect is caused by inadequate rates of detoxification of the reactive metabolite. |
| CID5460221 | Oleic Acid | 26674288 | induced a damage of the cytoplasmic membrane in rat primary hepatocytes. |
| CID31423 | Pyrene | 26160115 | activate human constitutive androstane receptor(CAR) which mediates pyrene-induced hepatotoxicity. |
| CID305 | Choline | 27316781;  4018729 | caused liver steatosis and impaired liver and vascular endothelium. |

**Figure S1.** Chemical space analysis of training set and test set.

**Figure S2.** Chemical space analysis of compounds in TCMSP.
